# Genetically predicted inflammatory cytokines and bone health outcomes: A 2-sample Mendelian randomization study

**DOI:** 10.1097/MD.0000000000047939

**Published:** 2026-03-06

**Authors:** Yujia Zhong, Fang Yang, Weiyin Chen

**Affiliations:** a Department of Rehabilitation, Chengdu Jinniu Hospital of Traditional Chinese Medicine, Chengdu, China; b Department of Neurology, Hospital of Chengdu University of Traditional Chinese Medicine, Chengdu, China.

**Keywords:** inflammation, Mendelian randomization, osteoarthritis, osteoporosis, rheumatoid arthritis

## Abstract

Circulating inflammatory factors have been implicated in various bone health outcomes, but the causal relationships remain unclear. We applied 2-sample Mendelian randomization to investigate the effects of 41 inflammatory factors on 5 bone health outcomes: osteoarthritis (OA), knee osteoarthritis (KOA), hip osteoarthritis, osteoporosis (OP), and rheumatoid arthritis (RA). We used publicly available summary statistics from genome-wide association studies of inflammatory factors and bone health outcomes. We selected genetic instruments for each inflammatory factor based on the largest available study, and performed inverse-variance weighted (IVW), Mendelian randomization-Egger, and weighted median analyses to estimate the causal effects. We also performed sensitivity analyses to assess the potential impact of horizontal pleiotropy, heterogeneity, and linkage disequilibrium. Genetically predicted IP-10 was associated with a higher odds of OA, and GROA was associated with a slightly higher odds of OP; 5 other cytokines showed inverse associations with selected outcomes in the IVW analysis (IP-10-OA, odds ratio [OR]: 1.09, 95% confidence interval [CI]: 1.01–1.17, *P* = .037; G-CSF-KOA, OR: 0.91, 95% CI: 0.86–0.96, *P* = .0006; SCF-HOA, OR: 0.90, 95% CI: 0.83–0.97, *P* = .004; GROA-OP,OR: 1.002,95% CI: 1.000–1.004,*P* = .014; MIP-1β-OP, OR: 0.999, 95% CI: 0.998–1.000, *P* = .031; IL-5-RA, OR: 0.91, 95% CI: 0.83–0.98, *P* = .019; PDGF-BB-RA, OR: 0.89, 95% CI: 0.80–0.98, *P* = .022). Across sensitivity analyses, we found no strong evidence of directional pleiotropy for these associations. This 2-sample Mendelian randomization study suggests several genetically predicted associations between inflammatory cytokines and bone health outcomes; however, given the small effect sizes and the limited sample sizes of the exposure genome-wide association studies, larger cytokine genome-wide association studies and independent validation are needed before clinical translation.

## 1. Introduction

An aging population will bring with it more chronic diseases and economic burdens on society and families.^[1]^ Bone health is a major concern for the aging population, as it affects the risk of developing various musculoskeletal disorders, such as osteoarthritis (OA),^[2]^ osteoporosis (OP),^[3,4]^ and rheumatoid arthritis (RA).^[5]^ These disorders are associated with reduced quality of life, increased morbidity and mortality, and substantial economic burden.^[6]^ According to the Global Burden of Disease Study 2017, OA was the 11th leading cause of years lived with disability worldwide, affecting 303 million people.^[7]^ OP was estimated to affect 200 million women globally, and caused about 9 million fractures annually.^[8]^ RA was the 42nd leading cause of years lived with disability worldwide, affecting 24.5 million people.^[7]^ Therefore, identifying the modifiable risk factors and potential preventive strategies for bone health is of great importance.

One of the emerging risk factors for bone health is chronic low-grade inflammation, which is characterized by elevated levels of circulating inflammatory markers, such as C-reactive protein (CRP), interleukin-6 (IL-6), and tumor necrosis factor-alpha (TNF-α).^[9]^ Chronic inflammation can impair bone formation and enhance bone resorption, leading to bone loss and increased fracture risk.^[10]^ Moreover, chronic inflammation can also trigger the activation of immune cells and cytokines that mediate joint damage and cartilage degradation in osteoarthritis and rheumatoid arthritis.^[11]^ However, the causal relationship between chronic inflammation and bone health remains unclear, as observational studies are prone to confounding and reverse causation.

Mendelian randomization (MR) is a genetic epidemiological method that uses genetic variants as IVs to infer the causal effect of an exposure on an outcome, independent of confounding or reverse causation.^[12]^ MR has been widely applied to investigate the causal role of various biomarkers and lifestyle factors on bone health.^[13-16]^ Previous MR studies have examined selected inflammatory pathways in relation to musculoskeletal phenotypes, such as CRP- and IL-6-related signaling with bone mineral density (BMD) and fracture risk, but most of these analyses have focused on only a few markers and specific skeletal sites and have often reported null or inconsistent causal estimates.^[17,18]^ More recently, MR studies using cytokine genome-wide association stud (GWAS) have started to evaluate panels of circulating inflammatory proteins in relation to BMD, OP and osteoporotic fractures, generally identifying robust associations for only a small subset of cytokines and largely site-specific or suggestive effects for the remainder.^[19-21]^ Parallel MR work on OA has begun to incorporate circulating cytokines or immune-related biomarkers into OA risk models, but these studies have also been restricted to a limited number of markers or OA phenotypes.^[22,23]^ Taken together, the existing evidence highlights the need for a broader and more systematic evaluation of circulating cytokines across multiple bone health endpoints using a unified analytical framework.

In this study, we aimed to evaluate the causal effect of 41 circulating inflammatory factors on bone health, including OA, OP, and RA, using a 2-sample MR approach. We obtained the summary statistics of GWAS for the inflammatory factors from a large-scale meta-analysis, and for the bone health outcomes from the online databases. We applied several robust MR methods to account for potential pleiotropy and heterogeneity. Our findings may provide novel insights into the role of chronic inflammation in bone health and suggest potential targets for prevention and treatment.

## 2. Materials and methods

To be a valid instrument for causal inference in MR studies, genetic variation should meet 3 key assumptions. First, genetic variation must be robustly associated with exposure (circulating inflammatory factors). Second, genetic variation must be independent of exposure-outcome confounders. Third, genetic variation must affect outcome (bone health) only through exposure (circulating inflammatory factors), with no other pleiotropic effects. We performed this MR study using a previously published, publicly accessible, large-scale GWAS summary dataset. All participants gave written informed consent in the original GWAS.

### 2.1. Data sources

The 41 circulating inflammatory factor data we used contain summary statistics from a meta-analysis of GWAS of inflammatory cytokines conducted in 3 Finnish cohorts.^[24]^ The study design 8293 European participants (Table 1).

**Table 1:** Information about circulating inflammatory factors used in this study.

Cytokines	Abbreviation	Sample size	SNPs
Beta nerve growth factor	B-NGF	3531	96,04,287
Cutaneous T-cell attracting (CCL27)	CTACK	3631	96,29,449
Eotaxin (CCL11)	EOTAXIN	8153	1,00,35,792
Basic fibroblast growth factor	FGF-BASIC	7565	1,00,31,426
Granulocyte colony-stimulating factor	G-CSF	7904	1,00,30,668
Growth-regulated oncogene-α (CXCL1)	GROA	3505	95,88,632
Hepatocyte growth factor	HGF	8292	1,00,60,102
Interferon γ	IFN-G	7701	1,00,25,322
Interleukin-10	IL-10	7681	1,00,40,760
Interleukin-12p70	IL-12-P70	8270	1,00,55,506
Interleukin-13	IL-13	3557	96,03,999
Interleukin-16	IL-16	3483	96,13,894
Interleukin-17	IL-17	7760	1,00,24,147
Interleukin-18	IL-18	3636	96,34,251
Interleukin-1-beta	IL-1B	3309	95,00,544
Interleukin-1 receptor antagonist	IL-1RA	3638	96,30,185
Interleukin-2	IL-2	3475	95,78,108
Interleukin-2 receptor, α subunit	IL-2RA	3677	96,43,655
Interleukin-4	IL-4	8124	1,00,28,095
Interleukin-5	IL-5	3364	95,13,957
Interleukin-6	IL-6	8189	1,00,40,617
Interleukin-7	IL-7	3409	95,55,295
Interleukin-8 (CXCL8)	IL-8	3526	95,82,336
Interleukin-9	IL-9	3634	96,33,899
Interferon γ-induced protein 10 (CXCL10)	IP-10	3685	96,38,000
Macrophage colony-stimulating factor	M-CSF	840	91,79,322
Monocyte chemotactic protein-1 (CCL2)	MCP-1-MCAF	8 293	1,00,57,790
Monocyte specific chemokine 3 (CCL7)	MCP-3	843	77,78,670
Macrophage migration inhibitory factor (glycosylation-inhibiting factor)	MIF	3494	95,97,221
Monokine induced by interferon γ (CXCL9)	MIG	3685	96,41,045
Macrophage inflammatory protein-1α (CCL3)	MIP-1A	3522	95,84,103
Macrophage inflammatory protein-1β (CCL4)	MIP-1B	8243	1,00,53,121
Platelet-derived growth factor-BB	PDGF-BB	8293	1,00,56,150
Regulated on activation, normal T cell expressed and secreted (CCL5)	RANTES	3421	95,90,306
Stem cell factor	SCF	8290	1,00,60,571
Stem cell growth factor beta	SCGF-B	3682	96,40,823
Stromal cell-derived factor-1α (CXCL12)	SDF-1A	5998	99,81,044
Tumor necrosis factor-α	TNF-A	3454	95,64,932
Tumor necrosis factor beta	TNF-B	1559	64,98 ,356
TNF-related apoptosis inducing ligand	TRAIL	8186	1,00,55,736
Vascular endothelial growth factor	VEGF	7118	1,00,32,332

SNP = single nucleotide polymorphism, TNF = tumor necrosis factor.

Bone health outcomes include OA, OP, and RA, and data on knee osteoarthritis (KOA) and hip osteoarthritis (HOA) have been added to the OA data. OA was defined based on self-reported status and association with hospital episode statistics, as well as the joint specificity of the disease (knee and/or hip). This OA data contains 63,556 sample sizes, all in European populations, involving more than 1500 single nucleotide polymorphisms (SNPs).^[25]^ The KOA and HOA data we used are the most recent GWAS summary data, with the deepest sequencing depth, involving close to 30 million SNPs.^[26]^ Using the UK Biobank and the arcOGEN resource, Tachmazidou et al analyzed the data of up to 4,55, 221 individuals with approximately 17.5 million single nucleotide variants in a genome-wide meta-analysis of OA. OP is defined as physician-confirmed self-reported OP or osteopenia, or BMD measurements in the heel, spine, or hip that are below the thresholds for OP set by the World Health Organization.^[26]^ It contains a sample size of 4,62,933, including both male and female European populations, with more than 9 million SNPs. Finally, we used RA data derived from retrieving RA association summary statistics from previous GWAS in Korean, Japanese, and European populations.^[27]^ To avoid population heterogeneity due to ethnic differences, we used the European population portion of this data, which included 14,361 cases and 43,923 controls, totaling more than 13 million SNPs.^[27]^

### 2.2. Selection of genetic instruments

We selected genetic variants that were strongly associated with each cytokine level at a genome-wide significance threshold of *P* < 5 × 10^−6^. We only included variants that were present in both GWAS datasets and had consistent effect directions and allele frequencies. To avoid bias due to linkage disequilibrium, we only included SNPs significantly associated with the exposure that had *r*^2^ < 0.01 and KB > 5000 in the final analysis. To minimize confounding and horizontal pleiotropy, we additionally examined whether the cytokine-associated SNPs had been reported to associate with major lifestyle and metabolic traits that might influence both inflammation and bone health. Each SNP was queried in the MRC IEU OpenGWAS project database using a threshold of *P* < 5E^−6^. Based on published GWAS, we identified genome-wide significant associations with traits such as body mass index, smoking, alcohol consumption, type 2 diabetes, lipid traits, and BMD, and excluded these SNPs from the final instrument set. We also used the *F*-statistic to quantify the strength of the genetic instrument and excluded SNPs with an *F*-statistic below 10.^[28]^

### 2.3. Mendelian randomization analysis

Statistical analysis was performed using the R programming language (version 4.1.2; R Foundation for Statistical Computing, Vienna, Austria). MR analysis was based on the “TwoSampleMR” package (version 0.5.6). And the “MRPRESSO” package (version 1.0) is used to apply MR-PRESSO analysis to identify SNPs with abnormal values and to delete abnormal SNPs for rerunning MR analysis.

We performed a 2-sample MR analysis using genetic instruments to estimate the causal effect of each cytokine level on the risk of OA (including KOA and HOA), OP, and RA. We used the inverse-variance weighted (IVW) method as the main analysis, which assumes that all genetic variants are valid instruments and provides a consistent estimate of the causal effect if this assumption holds.^[29]^ We also used the weighted median method, which only requires that at least 50% of the weight in the genetic instrument comes from valid variants.^[30,31]^ Additionally, we used the MR-Egger (Mendelian randomization-Egger) method, which allows for some degree of directional pleiotropy as long as it is independent of the variant’s strength as an instrument.^[30,32]^ The results are more convincing when the 3 methods are tested in agreement.

### 2.4. Sensitivity analysis

We performed several sensitivity analyses to test the robustness of our results. IVW and MR-Egger regression were used to test for heterogeneity, and *Q* statistics were produced to quantify it.^[32]^ If there was heterogeneity, we conducted the study using IVW with random effects. Horizontal pleiotropy is essential for our study because being affected by horizontal pleiotropy may lead to unstable effect estimates. The MR-Egger intercept method calculates the intercept term available after linear regression analysis to determine the likelihood of horizontal pleiotropy.^[33]^ We also performed a leave-one-out analysis to assess the influence of each variant on the overall estimate. The MR-PRESSO examination assesses the heterogeneity of studies and examines abnormal SNPs that may be heterogeneous.^[31]^ We used a software program to increase the number of distributions in the MR-PRESSO analysis to 5000, and then performed a global test to determine whether heterogeneity existed in the studies. We further assessed the robustness of the results of the MR analysis by comparing the effects before and after the exclusion of abnormal SNPs.^[34]^ If outliers were found, the MR analysis was performed again after culling the outliers.

## 3. Results

### 3.1. Selection of instrumental variables

In the end, 3 to 67 SNPs were identified as IVs, as detailed in Table S1, Supplemental Digital Content, https://links.lww.com/MD/R499. For the IVs used in the final analysis, all F statistics were >10 (range: 20.77–531.56). This suggests that these IVs are robust to the strong correlation assumption of MR.

### 3.2. Causal relationship between circulating inflammatory factors and the risk of osteoarthritis

**Figure 1.**
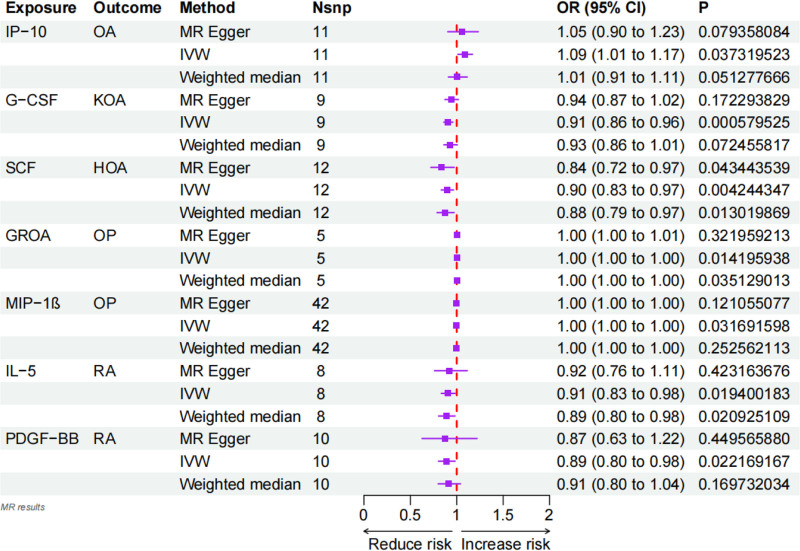
Positive results for the causal relationship between circulating inflammatory factors and. bone health. An OR value > 1 suggests that the exposure indicator is a risk factor, while the opposite is a protective factor. CI = confidence interval, HOA = hip osteoarthritis, IVW = inverse-variance weighted, KOA = knee osteoarthritis, MR = Mendelian randomization, OA = osteoarthritis, OP = osteoporosis, OR = odds ratio, RA = rheumatoid arthritis, SNP = single nucleotide polymorphism.

**Figure 2.**
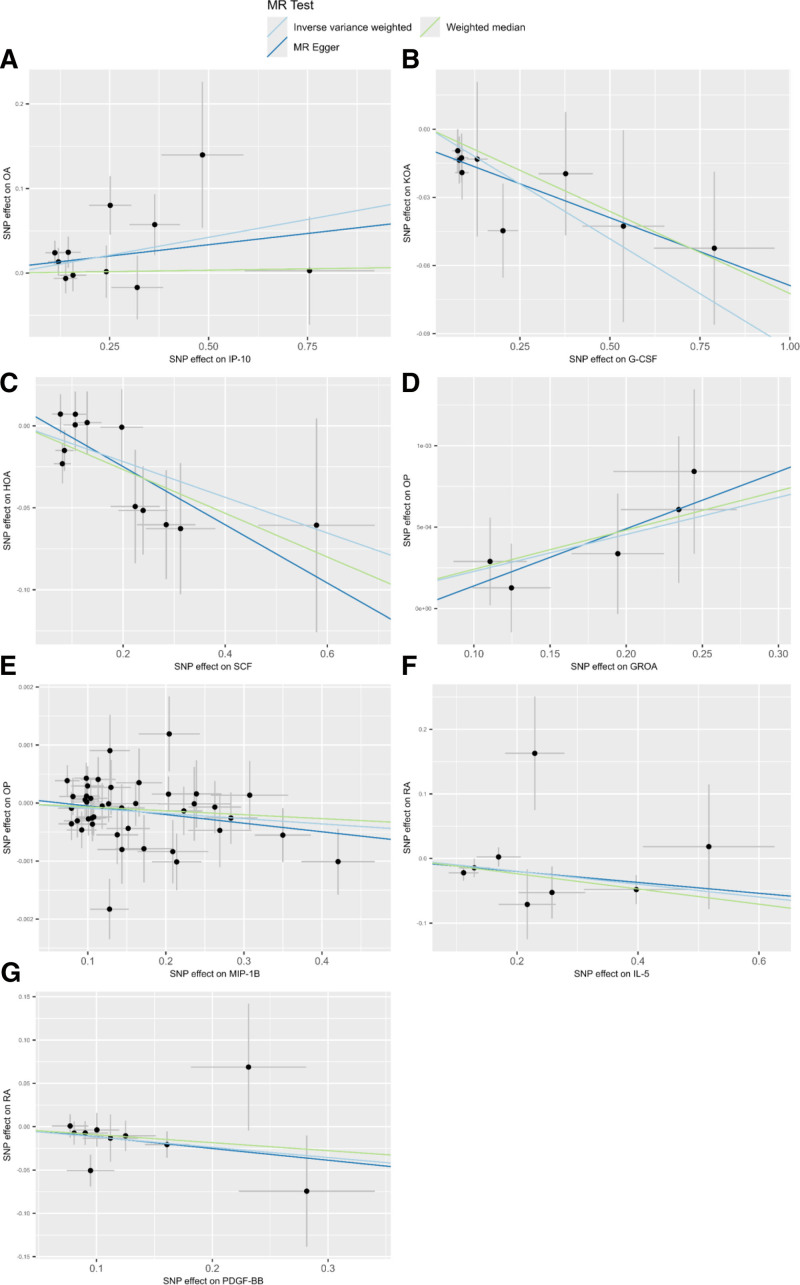
Scatter plot of genetic correlation between inflammatory factors and bone health by different MR analysis methods. HOA = hip osteoarthritis, KOA = knee osteoarthritis, OA = osteoarthritis, OP = osteoporosis, RA = rheumatoid arthritis, SNP = single nucleotide polymorphism. Figures A–G show the causal associations between IP-10, G-CSF, SCF, GROA, MIP-1B, IL-5, and PDGF-BB and bone health, respectively.

**Figure 3.**
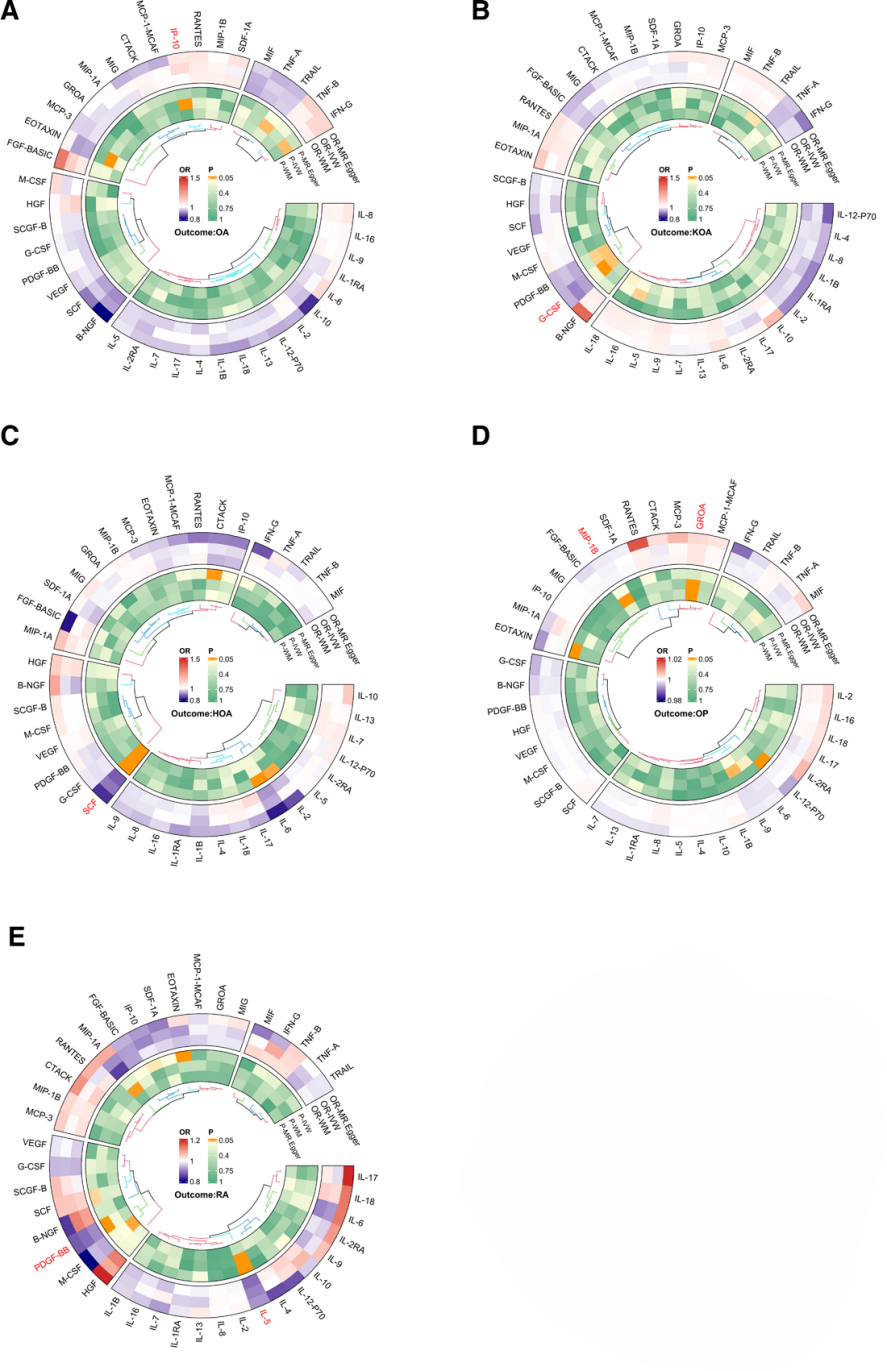
Circumplex plot of the causal relationship between circulating inflammatory factors and bone health. IVW = inverse-variance weighted, HOA = hip osteoarthritis, KOA = knee osteoarthritis, OA = osteoarthritis, OP = osteoporosis, RA = rheumatoid arthritis, WM = weighted median.

Under the MR assumptions, our study found that Interferon γ-induced protein 10 (IP-10) was significantly associated with OA and was identified as an OA-associated pathogenic cytokine based on the IVW method (IP-10-OA, odds ratio [OR]: 1.09, 95% confidence interval [CI]: 1.01–1.17, *P* = .037). In the IVW analysis, genetically predicted higher IP-10 levels were associated with an increased risk of OA (IP-10-OA, OR: 1.09, 95% CI: 1.01–1.17, *P* = .037; Fig. 1). The weighted median and MR-Egger estimates showed a similar direction of effect (Fig. 2). However, no causal relationship was found among other inflammatory cytokines (Fig. 3 and Table S2, Supplemental Digital Content, https://links.lww.com/MD/R499).

**Figure 4.**
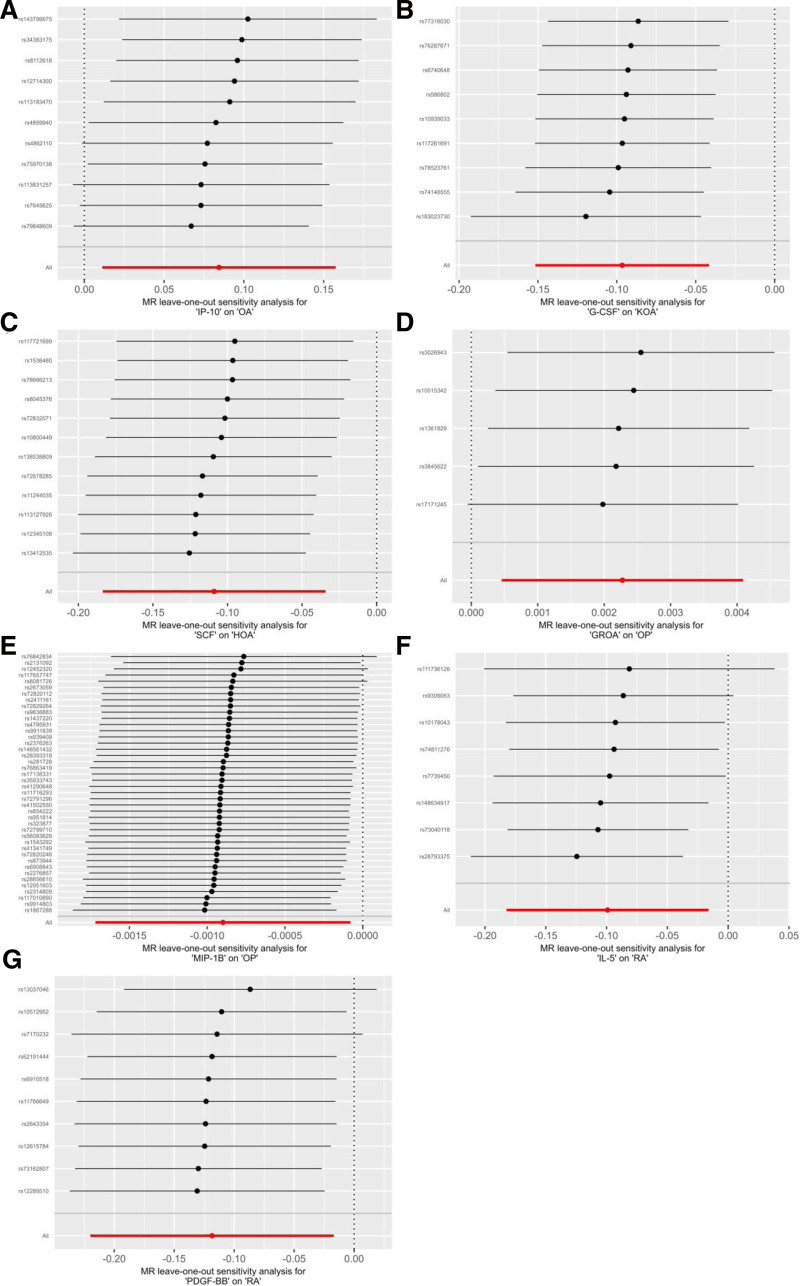
Leave-one-out sensitivity analysis between inflammatory factors and bone health. Red lines represent estimates from IVW tests. Figures A–G show the leave-one-out sensitivity analysis between IP-10, G-CSF, SCF, GROA, MIP-1B, IL-5, and PDGF-BB and bone health, respectively. IVW = inverse-variance weighted.

MR-Egger regression and IVW analysis were used to detect heterogeneity. For our positive results, MR-Egger regression (IP-10: Cochran’s *Q* = 10.37, *P* = .32) and IVW (IP-10: Cochran’s *Q* = 10.60, *P* = .39) did not show heterogeneity (Table S3, Supplemental Digital Content, https://links.lww.com/MD/R499). Similarly, the MR-Egger intercept did not show horizontal pleiotropy (IP-10: Intercept = 0.01, *P* = .66; Table S3, Supplemental Digital Content, https://links.lww.com/MD/R499). We used the leave-one-out method to remove SNPs 1 by 1 to determine whether the causal association was due to a single IV, and the final results showed that the MR analysis results were robust (Fig. 4).

### 3.3. Causal relationship between circulating inflammatory factors and the risk of knee osteoarthritis

In the KOA analysis, genetically predicted higher granulocyte colony-stimulating factor (G-CSF) levels were associated with a reduced risk of KOA in the IVW model (G-CSF-KOA, OR: 0.91, 95% CI: 0.86–0.96, *P* = .0006; Fig. 1). And Cochran’s *Q* statistic for MR-Egger and IVW showed no heterogeneity (Table S3, Supplemental Digital Content, https://links.lww.com/MD/R499). In the MR-Egger regression, the intercept term indicated that horizontal pleiotropy had no effect on the results (Intercept = −0.01, *P* = .22; Table S3, Supplemental Digital Content, https://links.lww.com/MD/R499). Leave-one-out analysis showed that the overall risk estimate was not significantly affected by any single SNP (Fig. 4).

### 3.4. Causal relationship between circulating inflammatory factors and the risk of hip osteoarthritis

In the analysis of HOA, the IVW model suggested that genetically predicted higher stem cell factor (SCF) levels were associated with a reduced risk of HOA (SCF-HOA, OR: 0.90, 95% CI: 0.83–0.97, *P* = .004; Fig. 1). The weighted median and MR-Egger estimates were directionally consistent. And the Cochran’s *Q* statistic for MR-Egger and IVW showed no heterogeneity (Table S3, Supplemental Digital Content, https://links.lww.com/MD/R499). In the MR-Egger regression, the intercept term indicated that horizontal pleiotropy also had no effect on the results (Intercept = 0.01, *P* = .27; Table S3, Supplemental Digital Content, https://links.lww.com/MD/R499). Leave-one-out analyses indicated that either individual SNP would not have a substantial effect on the overall risk estimate (Fig. 4).

### 3.5. Causal relationship between circulating inflammatory factors and the risk of osteoporosis

Genetically predicted growth-regulated oncogene-α (GROA) levels were associated with a slightly increased risk of OP, and macrophage inflammatory protein-1β (MIP-1β) levels were associated with a slightly decreased risk of OP (GROA-OP, OR: 1.002, 95% CI: 1.000–1.004, *P* = .014; MIP-1β-OP, OR: 0.999, 95% CI: 0.998–1.000, *P* = .031; Fig. 1). These ORs were very close to the null. We found little statistical evidence of directional pleiotropy or heterogeneity based on the MR-Egger intercept and Cochran’s *Q* statistics (Table S3, Supplemental Digital Content, https://links.lww.com/MD/R499), but given the small effect sizes and modest instrument strength, these associations should be interpreted cautiously.

### 3.6. Causal relationship between circulating inflammatory factors and the risk of rheumatoid arthritis

In random-effects IVW analyses, genetically predicted higher interleukin-5 (IL-5) and platelet-derived growth factor-BB (PDGF-BB) levels were associated with a reduced risk of RA (IL-5-RA, OR: 0.91, 95% CI: 0.83–0.98, *P* = .019; PDGF-BB-RA, OR: 0.89, 95% CI: 0.80–0.98, *P* = .022). We did not observe strong statistical evidence of directional pleiotropy or heterogeneity, and leave-one-out analyses did not identify single SNPs that fully drove the associations.

## 4. Discussion

In this study, we used a 2-sample MR approach to investigate the causal effects of 41 circulating inflammatory factors on bone health, including OA, OP, and RA. We used summary statistics from large-scale GWAS on inflammatory factors and bone health outcomes. We found several significant associations between some inflammatory factors and different aspects of bone health, which may provide novel insights into the role of chronic inflammation in bone health and suggest potential targets for prevention and treatment.

There is a strong relationship between inflammatory factors and bone health. In OA, the release of early inflammatory cytokines induces the activation of inflammatory signaling pathways.^[35]^ For example, interleukin-1β (IL-1β) causes inflammatory factors to accumulate through activation of the phosphatidylinositol 3-kinase/protein kinase B (PI3K/AKT) pathway, leading to altered physiological function of the joint.^[11]^ It has been shown that pro-inflammatory cytokines can alter bone formation by controlling transcription factors that up-regulate or down-regulate miRNA expression, leading to a balance of osteoblast genesis and/or osteoclast genesis.^[36]^ However, there are many inflammatory cytokines and a systematic analysis between inflammatory factors and bone health is not currently done. Therefore, we systematically assessed the causal association between inflammatory factors and bone health using MR methods.

The causal mechanisms by which these cytokines increase the risk of bone-related diseases are not fully understood, but some possible explanations can be suggested. We found that IP-10 was positively associated with OA, indicating that IP-10 may be a risk factor for OA. IP-10, also known as CXCL10, is a chemokine that attracts activated T cells and monocytes to sites of inflammation.^[37]^ Previous studies have shown that IP-10 is elevated in the serum and synovial fluid of patients with OA, and correlates with disease severity and progression.^[38,39]^ Moreover, IP-10 has been shown to induce cartilage degradation and chondrocyte apoptosis in vitro.^[40]^ Therefore, our finding is consistent with the existing evidence and supports the hypothesis that IP-10 may play a causal role in OA pathogenesis.

Another interesting finding is that G-CSF negatively correlates with KOA and SCF negatively correlates with HOA.G-CSF is a cytokine that stimulates the production and mobilization of bone marrow neutrophils.^[41]^ One study found a new therapeutic modality for arthritis, G-CSF receptor blockade improved arthritis pain.^[42]^ However, we also found that G-CSF has a pro-angiogenic capacity and has therapeutic effects on tissue repair and regeneration. This may be the key role it plays in KOA.^[43]^ SCF has an active role in hematopoiesis, which can lead to increased blood flow around the hip joint and promote the metabolism of inflammatory substances, which in turn is a protective factor.^[44]^

In terms of OP, we found a positive correlation between GROA and OP, and conversely, MIP-1β was a protective factor for OP. GROA, also known as CXCL1 or IL-8, is a chemokine that attracts neutrophils and other leukocytes to sites of inflammation.^[45]^ It has been shown that GROA can be able to activate and differentiate osteoclasts through the Wnt/DKK1/sclerostin pathway, leading to bone loss.^[46]^ Therefore, our findings are consistent with previous evidence and support the hypothesis that GROA may be involved in the pathogenesis of OP. MIP-1β, also known as CCL4, is a chemokine that is involved in osteoclast activity and pathological progression of bone metabolism diseases.^[47]^ And it has been suggested that CCL4 may be a key factor in the progression of postmenopausal female patients with OP.^[48]^ However, our results showed that MIP-1β is a protective factor in OP. CCL4 enhances immunity by recruiting cells such as macrophages, which may play a positive role in the development of OP.^[49]^ More studies on different mechanisms are needed in the future.

Regarding RA, we identified IL-5 and PDGF-BB as possible protective factors for RA. IL-5 is a cytokine that stimulates the production and activation of eosinophils, which are involved in allergic.^[50]^ PDGF-BB is a growth factor that promotes the proliferation and migration of fibroblasts, smooth muscle cells, and endothelial cells, and is involved in wound healing and angiogenesis.^[51]^ Previous studies have reported that both IL-5 and PDGF-BB are elevated in the serum and synovial fluid of RA patients, and that they have pro-inflammatory and tissue-destructive effects on RA pathogenesis.^[52]^ However, our results suggest that these factors may have a beneficial role in preventing or ameliorating RA.

One possible explanation for this paradoxical finding is that IL-5 and PDGF-BB may have dual functions in RA, depending on the stage and severity of the disease. IL-5 may have anti-inflammatory effects by inhibiting the production of pro-inflammatory cytokines such as TNF-α, IL-1beta, and IL-6, and by inducing the differentiation of regulatory B cells, which can suppress the activation of autoreactive T cells and produce anti-inflammatory cytokines such as IL-10 and TGF-β.^[53,54]^ PDGF-BB may have tissue-protective effects by stimulating the synthesis of extracellular matrix proteins such as collagen and proteoglycans, and by enhancing the survival and function of chondrocytes, which are the main cells responsible for maintaining the integrity of articular cartilage.^[51]^ Therefore, IL-5 and PDGF-BB may have a compensatory or regulatory role in RA, especially in the early or mild stages of the disease, when the inflammatory and destructive processes are not overwhelming.

Our study has several strengths and limitations. The main strength is the use of a 2-sample MR approach, which allows us to infer causality from observational data by using genetic variants as IVs. This approach reduces the confounding and reverse causation problems that plague conventional epidemiological studies. Moreover, we used summary statistics from large-scale GWAS for both the inflammatory factors and the bone health outcomes, which increased the statistical power and the generalizability of our results. Furthermore, we applied several robust MR methods to account for potential pleiotropy and heterogeneity among the genetic instruments. Our study could provide theoretical support for exploring the use of inflammatory markers to predict disease progression and treatment outcomes.

The main limitation of our study is that we assumed a linear relationship between cytokine levels and skeletal disease risk, which may not reflect the complex dose-response curves or threshold effects that may exist in reality. In addition, we did not consider possible interactions or correlations between cytokines or other mediators that may affect skeletal disease risk. Third, all subjects in the GWAS data were of European descent, and further research is needed to determine whether the findings can be generalized to populations of European descent. Another important limitation is the potential for weak instrument bias. Several cytokines were instrumented by only a few SNPs derived from GWAS with relatively small sample sizes, and many effect estimates were close to 1 with wide confidence intervals. Under these circumstances, MR-Egger and weighted median estimates are inherently unstable, and our findings for such cytokines should be interpreted with particular caution. Finally, because several *P* values were close to the conventional significance threshold and many tests were conducted, it is likely that some nominally significant associations would not remain significant after multiple-testing adjustment and should be interpreted with caution.

## 5. Conclusion

In this study, we applied a 2-sample MR approach to explore the potential causal role of 41 circulating inflammatory factors in bone health. We identified several associations between genetically predicted cytokine levels and different skeletal outcomes, including IP-10 with OA, G-CSF with KOA, SCF with HOA, GROA and MIP-1β with OP, and IL-5 and PDGF-BB with rheumatoid arthritis. These findings suggest that chronic inflammatory pathways may be involved in the development of bone and joint diseases and highlight candidate cytokines for further investigation. However, further studies are needed to validate our results and to elucidate the underlying mechanisms.

## Acknowledgments

Our data is taken from the publicly available GWAS database. We thank the genetics consortiums for publicly making the GWAS summary data available.

## Author contributions

**Conceptualization:** Yujia Zhong.

**Investigation:** Fang Yang.

**Methodology:** Yujia Zhong, Fang Yang.

**Project administration:** Weiyin Chen.

**Resources:** Yujia Zhong.

**Supervision:** Weiyin Chen.

**Visualization:** Fang Yang.

**Writing – original draft:** Yujia Zhong.

**Writing – review & editing:** Yujia Zhong.

**Funding Statement:** There was no additional external funding received for this study.

## Supplementary Material


