# Supplementary material for: Genetically predicted inflammatory cytokines and bone health outcomes: A 2-sample Mendelian randomization study
Source: Medicine (Baltimore). 2026 Mar 6;105(10):e47939. doi: 10.1097/MD.0000000000047939 (PMC12975193; doi:10.1097/MD.0000000000047939)
Supplement: Supplementary file 1 [file medi-105-e47939-s001.docx]

**Supplementary Table 1. Instrumental variables for inflammatory factors and OA.**

| **Exposure** | **SNP** | | **Chr** | **EA** | **OA** | **Beta** | **EAF** | **SE** | **P** | **F** | |
| --- | --- | --- | --- | --- | --- | --- | --- | --- | --- | --- | --- |
| B-NGF | rs73472576 | | 18 | T | C | -0.1146 | 0.457 | 0.0251 | 4.98E-06 | 20.85 | |
| B-NGF | rs4767014 | | 12 | T | C | -0.1211 | 0.6731 | 0.0264 | 4.49E-06 | 21.04 | |
| B-NGF | rs71641308 | | 1 | T | C | 0.1969 | 0.0995 | 0.0429 | 4.44E-06 | 21.07 | |
| B-NGF | rs28637706 | | 19 | T | G | -0.1554 | 0.314 | 0.0261 | 2.62E-09 | 35.45 | |
| CTACK | rs57338032 | | 15 | A | G | 0.1443 | 0.8287 | 0.0316 | 4.96E-06 | 20.85 | |
| CTACK | rs62578137 | | 9 | T | C | -0.1311 | 0.2421 | 0.0286 | 4.56E-06 | 21.01 | |
| CTACK | rs60247384 | | 3 | T | C | 0.1128 | 0.3512 | 0.0245 | 4.14E-06 | 21.20 | |
| CTACK | rs118084576 | | 9 | A | G | 0.5675 | 0.0112 | 0.1226 | 3.68E-06 | 21.43 | |
| CTACK | rs57789542 | | 3 | T | C | -0.7687 | 0.9951 | 0.1659 | 3.60E-06 | 21.47 | |
| CTACK | rs116303454 | | 3 | A | G | 0.3754 | 0.0218 | 0.081 | 3.58E-06 | 21.48 | |
| CTACK | rs117932939 | | 9 | T | C | 0.1969 | 0.09 | 0.0422 | 3.07E-06 | 21.77 | |
| CTACK | rs72729450 | | 9 | T | C | -0.5123 | 0.0123 | 0.1094 | 2.83E-06 | 21.93 | |
| CTACK | rs116943377 | | 13 | A | G | 0.2878 | 0.0394 | 0.0611 | 2.47E-06 | 22.19 | |
| CTACK | rs184329319 | | 9 | T | G | -0.3069 | 0.035 | 0.0648 | 2.18E-06 | 22.43 | |
| CTACK | rs76395525 | | 15 | A | G | 0.5193 | 0.0118 | 0.1081 | 1.56E-06 | 23.08 | |
| CTACK | rs10854859 | | 22 | A | G | -0.1498 | 0.2464 | 0.0293 | 3.18E-07 | 26.14 | |
| CTACK | rs2233872 | | 9 | A | G | -0.1495 | 0.7699 | 0.0282 | 1.15E-07 | 28.11 | |
| CTACK | rs55764737 | | 15 | T | C | 0.5424 | 0.9846 | 0.0967 | 2.03E-08 | 31.46 | |
| CTACK | rs144072067 | | 9 | A | G | 0.4451 | 0.9574 | 0.0579 | 1.50E-14 | 59.10 | |
| EOTAXIN | rs9975149 | | 21 | A | T | 0.0749 | 0.3743 | 0.0164 | 4.95E-06 | 20.86 | |
| EOTAXIN | rs2097947 | | 7 | A | T | -0.0769 | 0.6836 | 0.0168 | 4.71E-06 | 20.95 | |
| EOTAXIN | rs2027855 | | 22 | T | C | 0.0743 | 0.6153 | 0.0162 | 4.51E-06 | 21.04 | |
| EOTAXIN | rs57723662 | | 17 | C | G | -0.0982 | 0.1657 | 0.0213 | 4.02E-06 | 21.26 | |
| EOTAXIN | rs745331 | | 15 | A | G | -0.0821 | 0.6825 | 0.0176 | 3.09E-06 | 21.76 | |
| EOTAXIN | rs7231030 | | 18 | A | C | 0.0903 | 0.7919 | 0.0193 | 2.89E-06 | 21.89 | |
| EOTAXIN | rs4683182 | | 3 | A | G | 0.0886 | 0.2201 | 0.0189 | 2.76E-06 | 21.98 | |
| EOTAXIN | rs75426604 | | 14 | A | C | -0.1371 | 0.0876 | 0.0291 | 2.46E-06 | 22.20 | |
| EOTAXIN | rs1677588 | | 1 | T | G | 0.1181 | 0.886 | 0.025 | 2.31E-06 | 22.32 | |
| EOTAXIN | rs60075014 | | 5 | T | C | -0.1688 | 0.0604 | 0.0356 | 2.12E-06 | 22.48 | |
| EOTAXIN | rs2040143 | | 21 | A | G | -0.0858 | 0.7234 | 0.0178 | 1.43E-06 | 23.23 | |
| EOTAXIN | rs147287945 | | 6 | A | G | -0.1512 | 0.0741 | 0.0313 | 1.36E-06 | 23.34 | |
| EOTAXIN | rs7550207 | | 1 | T | C | -0.0904 | 0.7785 | 0.0187 | 1.34E-06 | 23.37 | |
| EOTAXIN | rs2828756 | | 21 | T | C | 0.0904 | 0.2423 | 0.0184 | 8.97E-07 | 24.14 | |
| EOTAXIN | rs5754733 | | 22 | A | C | -0.105 | 0.8306 | 0.0213 | 8.24E-07 | 24.30 | |
| EOTAXIN | rs9317045 | | 13 | A | C | 0.1172 | 0.8615 | 0.0236 | 6.83E-07 | 24.66 | |
| EOTAXIN | rs2249581 | | 1 | T | C | -0.0899 | 0.748 | 0.018 | 5.90E-07 | 24.94 | |
| EOTAXIN | rs34004101 | | 3 | T | C | -0.1775 | 0.065 | 0.0341 | 1.94E-07 | 27.09 | |
| EOTAXIN | rs2024050 | | 7 | A | G | 0.164 | 0.0739 | 0.0302 | 5.62E-08 | 29.49 | |
| EOTAXIN | rs73065695 | | 3 | A | G | 0.1532 | 0.0932 | 0.0281 | 4.98E-08 | 29.72 | |
| EOTAXIN | rs9833459 | | 3 | T | C | -0.1149 | 0.3033 | 0.0173 | 3.10E-11 | 44.11 | |
| EOTAXIN | rs11920996 | | 3 | T | C | 0.2979 | 0.0512 | 0.0377 | 2.75E-15 | 62.44 | |
| EOTAXIN | rs2229593 | | 3 | T | C | 0.3647 | 0.0387 | 0.0406 | 2.64E-19 | 80.69 | |
| FGF-BASIC | rs78873483 | | 17 | A | G | 0.1286 | 0.1025 | 0.0282 | 5.11E-06 | 20.80 | |
| FGF-BASIC | rs17094040 | | 14 | T | C | 0.1051 | 0.1522 | 0.0229 | 4.44E-06 | 21.06 | |
| FGF-BASIC | rs76253061 | | 5 | T | C | -0.4811 | 0.9925 | 0.1041 | 3.81E-06 | 21.36 | |
| FGF-BASIC | rs4795091 | | 17 | A | G | 0.1239 | 0.8926 | 0.0266 | 3.19E-06 | 21.70 | |
| FGF-BASIC | rs147409637 | | 6 | T | C | 0.201 | 0.0369 | 0.0431 | 3.11E-06 | 21.75 | |
| FGF-BASIC | rs2849358 | | 18 | A | G | 0.0911 | 0.2551 | 0.0193 | 2.36E-06 | 22.28 | |
| FGF-BASIC | rs13412535 | | 2 | A | G | -0.1129 | 0.1856 | 0.0224 | 4.65E-07 | 25.40 | |
| G-CSF | rs117261691 | | 19 | T | C | 0.1318 | 0.0894 | 0.0288 | 4.73E-06 | 20.94 | |
| G-CSF | rs183023730 | | 9 | T | G | 0.7898 | 0.0034 | 0.1677 | 2.48E-06 | 22.18 | |
| G-CSF | rs586802 | | 11 | A | G | 0.0882 | 0.758 | 0.0187 | 2.40E-06 | 22.25 | |
| G-CSF | rs78523761 | | 5 | A | G | 0.5374 | 0.0077 | 0.1139 | 2.38E-06 | 22.26 | |
| G-CSF | rs76287671 | | 19 | T | C | 0.0894 | 0.2333 | 0.0189 | 2.24E-06 | 22.37 | |
| G-CSF | rs10939033 | | 4 | A | G | -0.0775 | 0.6038 | 0.0163 | 1.99E-06 | 22.61 | |
| G-CSF | rs6740648 | | 2 | T | C | 0.0818 | 0.6752 | 0.0172 | 1.98E-06 | 22.62 | |
| G-CSF | rs74148555 | | 10 | T | C | -0.3771 | 0.0144 | 0.0753 | 5.50E-07 | 25.08 | |
| GROA | rs62024303 | | 15 | A | G | -0.3013 | 0.9617 | 0.066 | 4.99E-06 | 20.84 | |
| GROA | rs1361829 | | 1 | A | G | -0.1106 | 0.4293 | 0.0241 | 4.45E-06 | 21.06 | |
| GROA | rs76390238 | | 2 | C | G | 0.6223 | 0.0095 | 0.1352 | 4.17E-06 | 21.19 | |
| GROA | rs17171245 | | 7 | T | G | 0.2446 | 0.0536 | 0.053 | 3.93E-06 | 21.30 | |
| GROA | rs150194856 | | 1 | T | C | -0.4223 | 0.0177 | 0.0914 | 3.83E-06 | 21.35 | |
| GROA | rs188345231 | | 8 | T | C | 0.6177 | 0.0102 | 0.1322 | 2.98E-06 | 21.83 | |
| GROA | rs76215157 | | 20 | C | G | -0.7398 | 0.0074 | 0.1564 | 2.24E-06 | 22.37 | |
| GROA | rs140734053 | | 10 | A | G | 0.7333 | 0.0066 | 0.1545 | 2.07E-06 | 22.53 | |
| GROA | rs73020704 | | 1 | A | G | 0.2607 | 0.9484 | 0.0545 | 1.72E-06 | 22.88 | |
| GROA | rs3026943 | | 1 | A | C | -0.1246 | 0.3311 | 0.0256 | 1.13E-06 | 23.69 | |
| GROA | rs185768063 | | 6 | A | G | 0.4038 | 0.9735 | 0.076 | 1.08E-07 | 28.23 | |
| GROA | rs115214168 | | 4 | T | C | 0.4528 | 0.0242 | 0.0828 | 4.54E-08 | 29.91 | |
| GROA | rs3845622 | | 1 | A | C | -0.2345 | 0.1171 | 0.0381 | 7.51E-10 | 37.88 | |
| GROA | rs10015342 | | 4 | A | T | 0.1945 | 0.1909 | 0.0304 | 1.57E-10 | 40.93 | |
| HGF | rs11129909 | | 3 | T | C | -0.0738 | 0.623 | 0.0161 | 4.56E-06 | 21.01 | |
| HGF | rs362307 | | 4 | T | C | 0.1511 | 0.0646 | 0.0328 | 4.09E-06 | 21.22 | |
| HGF | rs11060254 | | 12 | A | G | -0.0765 | 0.3306 | 0.0166 | 4.06E-06 | 21.24 | |
| HGF | rs2003620 | | 7 | T | C | 0.2277 | 0.027 | 0.0487 | 2.93E-06 | 21.86 | |
| HGF | rs4245058 | | 11 | T | C | -0.1552 | 0.0619 | 0.0331 | 2.75E-06 | 21.99 | |
| HGF | rs57146176 | | 12 | A | G | -0.0987 | 0.7998 | 0.0208 | 2.08E-06 | 22.52 | |
| HGF | rs80051150 | | 21 | T | C | 0.198 | 0.9595 | 0.0413 | 1.63E-06 | 22.98 | |
| HGF | rs13412535 | | 2 | A | G | -0.1043 | 0.1892 | 0.0213 | 9.75E-07 | 23.98 | |
| HGF | rs2699434 | | 4 | T | C | 0.0863 | 0.3287 | 0.0171 | 4.49E-07 | 25.47 | |
| HGF | rs5745687 | | 7 | T | C | -0.3008 | 0.0375 | 0.0404 | 9.65E-14 | 55.44 | |
| IFN-G | rs11843756 | | 13 | T | G | 0.1812 | 0.9542 | 0.0391 | 3.58E-06 | 21.48 | |
| IFN-G | rs113399544 | | 7 | A | G | -0.0849 | 0.2768 | 0.0183 | 3.50E-06 | 21.52 | |
| IFN-G | rs117046255 | | 7 | T | C | -0.0968 | 0.1922 | 0.0207 | 2.92E-06 | 21.87 | |
| IFN-G | rs73479333 | | 6 | C | G | -0.1123 | 0.1354 | 0.024 | 2.88E-06 | 21.89 | |
| IFN-G | rs1867282 | | 9 | T | C | 0.0781 | 0.4154 | 0.0166 | 2.54E-06 | 22.14 | |
| IFN-G | rs12420286 | | 11 | T | C | 0.2357 | 0.9699 | 0.05 | 2.43E-06 | 22.22 | |
| IFN-G | rs7088799 | | 10 | T | G | -0.0805 | 0.6182 | 0.0166 | 1.24E-06 | 23.52 | |
| IFN-G | rs115729819 | | 4 | A | G | 0.2511 | 0.9716 | 0.0514 | 1.03E-06 | 23.87 | |
| IFN-G | rs2073438 | | 17 | A | G | 0.092 | 0.2506 | 0.0188 | 9.90E-07 | 23.95 | |
| IFN-G | rs74148555 | | 10 | T | C | -0.3771 | 0.0144 | 0.077 | 9.71E-07 | 23.98 | |
| IFN-G | rs113600793 | | 17 | A | C | 0.1871 | 0.0631 | 0.0371 | 4.58E-07 | 25.43 | |
| IFN-G | rs147378920 | | 1 | A | G | -0.384 | 0.9871 | 0.0751 | 3.17E-07 | 26.14 | |
| IL-10 | rs3002131 | | 1 | C | G | 0.1191 | 0.1195 | 0.026 | 4.63E-06 | 20.98 | |
| IL-10 | rs41282660 | | 6 | A | G | -0.1169 | 0.8646 | 0.0254 | 4.18E-06 | 21.18 | |
| IL-10 | rs73192842 | | 3 | A | G | 0.0949 | 0.1911 | 0.0206 | 4.09E-06 | 21.22 | |
| IL-10 | rs465757 | | 20 | A | G | 0.0806 | 0.6657 | 0.0174 | 3.62E-06 | 21.46 | |
| IL-10 | rs4741748 | | 9 | A | G | -0.0788 | 0.6081 | 0.0169 | 3.12E-06 | 21.74 | |
| IL-10 | rs383684 | | 6 | A | G | 0.092 | 0.7555 | 0.0197 | 3.01E-06 | 21.81 | |
| IL-10 | rs6054847 | | 20 | T | C | 0.0971 | 0.8091 | 0.0207 | 2.72E-06 | 22.00 | |
| IL-10 | rs339203 | | 2 | T | C | 0.0954 | 0.8359 | 0.0203 | 2.61E-06 | 22.09 | |
| IL-10 | rs2086656 | | 4 | T | C | -0.08 | 0.6621 | 0.017 | 2.53E-06 | 22.15 | |
| IL-10 | rs3025021 | | 6 | T | C | 0.0913 | 0.3459 | 0.0194 | 2.52E-06 | 22.15 | |
| IL-10 | rs1530455 | | 3 | T | C | 0.082 | 0.3523 | 0.0174 | 2.45E-06 | 22.21 | |
| IL-10 | rs6680918 | | 1 | T | C | -0.1202 | 0.8791 | 0.025 | 1.52E-06 | 23.12 | |
| IL-10 | rs10493718 | | 1 | A | C | -0.1081 | 0.1603 | 0.0222 | 1.12E-06 | 23.71 | |
| IL-10 | rs143072171 | | 6 | T | C | 0.2089 | 0.0413 | 0.0429 | 1.12E-06 | 23.71 | |
| IL-10 | rs865585 | | 6 | A | C | -0.119 | 0.8478 | 0.0244 | 1.08E-06 | 23.79 | |
| IL-10 | rs7088799 | | 10 | T | G | -0.0815 | 0.6191 | 0.0166 | 9.12E-07 | 24.10 | |
| IL-10 | rs10457128 | | 6 | A | G | -0.0854 | 0.639 | 0.0172 | 6.87E-07 | 24.65 | |
| IL-10 | rs910604 | | 6 | A | G | -0.0986 | 0.2383 | 0.0196 | 4.89E-07 | 25.31 | |
| IL-10 | rs181031888 | | 6 | A | T | 0.2577 | 0.961 | 0.0472 | 4.77E-08 | 29.81 | |
| IL-10 | rs7747448 | | 6 | A | G | -0.1061 | 0.2594 | 0.0189 | 1.98E-08 | 31.51 | |
| IL-10 | rs13412535 | | 2 | A | G | -0.1347 | 0.1856 | 0.0224 | 1.82E-09 | 36.16 | |
| IL-12-P70 | rs12154194 | | 6 | T | C | 0.0717 | 0.4615 | 0.0157 | 4.95E-06 | 20.86 | |
| IL-12-P70 | rs6532374 | | 4 | T | C | -0.1033 | 0.8525 | 0.0226 | 4.86E-06 | 20.89 | |
| IL-12-P70 | rs12969892 | | 18 | T | C | 0.1227 | 0.9031 | 0.0267 | 4.32E-06 | 21.12 | |
| IL-12-P70 | rs2123852 | | 19 | T | C | 0.0942 | 0.2039 | 0.0204 | 3.88E-06 | 21.32 | |
| IL-12-P70 | rs41282644 | | 6 | A | G | 0.1401 | 0.0849 | 0.0303 | 3.77E-06 | 21.38 | |
| IL-12-P70 | rs6458375 | | 6 | T | C | 0.0884 | 0.2407 | 0.0191 | 3.69E-06 | 21.42 | |
| IL-12-P70 | rs9472175 | | 6 | T | C | -0.1104 | 0.1542 | 0.0238 | 3.51E-06 | 21.52 | |
| IL-12-P70 | rs34826779 | | 8 | T | G | -0.0884 | 0.2157 | 0.019 | 3.28E-06 | 21.65 | |
| IL-12-P70 | rs282258 | | 2 | T | C | 0.0726 | 0.4279 | 0.0156 | 3.26E-06 | 21.66 | |
| IL-12-P70 | rs273702 | | 18 | A | G | -0.127 | 0.9015 | 0.027 | 2.55E-06 | 22.12 | |
| IL-12-P70 | rs2495005 | | 10 | A | G | -0.075 | 0.4416 | 0.0159 | 2.39E-06 | 22.25 | |
| IL-12-P70 | rs7765264 | | 6 | A | G | 0.1428 | 0.0767 | 0.0301 | 2.09E-06 | 22.51 | |
| IL-12-P70 | rs34322762 | | 9 | T | C | 0.0953 | 0.648 | 0.0199 | 1.68E-06 | 22.93 | |
| IL-12-P70 | rs9381249 | | 6 | T | C | -0.1788 | 0.9466 | 0.0367 | 1.11E-06 | 23.74 | |
| IL-12-P70 | rs4741748 | | 9 | A | G | -0.0799 | 0.6076 | 0.0163 | 9.49E-07 | 24.03 | |
| IL-12-P70 | rs782111 | | 12 | A | C | -0.0765 | 0.5292 | 0.0156 | 9.40E-07 | 24.05 | |
| IL-12-P70 | rs4714698 | | 6 | A | G | -0.1189 | 0.8721 | 0.0238 | 5.86E-07 | 24.96 | |
| IL-12-P70 | rs113600793 | | 17 | A | C | 0.1832 | 0.0623 | 0.0359 | 3.34E-07 | 26.04 | |
| IL-12-P70 | rs148449807 | | 6 | A | G | -0.4217 | 0.0164 | 0.0793 | 1.05E-07 | 28.28 | |
| IL-12-P70 | rs13190738 | | 6 | T | C | 0.1019 | 0.3609 | 0.019 | 8.18E-08 | 28.76 | |
| IL-12-P70 | rs9472153 | | 6 | A | G | 0.0855 | 0.5141 | 0.0159 | 7.56E-08 | 28.92 | |
| IL-12-P70 | rs7763358 | | 6 | T | C | 0.1525 | 0.0885 | 0.0274 | 2.61E-08 | 30.98 | |
| IL-12-P70 | rs181031888 | | 6 | A | T | 0.2861 | 0.9606 | 0.0456 | 3.52E-10 | 39.36 | |
| IL-12-P70 | rs865585 | | 6 | A | C | -0.1654 | 0.8484 | 0.0237 | 2.97E-12 | 48.71 | |
| IL-13 | rs76975337 | | 3 | T | C | -0.1211 | 0.7286 | 0.0265 | 4.88E-06 | 20.88 | |
| IL-13 | rs7073807 | | 10 | T | C | 0.1618 | 0.1351 | 0.0354 | 4.86E-06 | 20.89 | |
| IL-13 | rs27949 | | 5 | T | C | -0.1144 | 0.667 | 0.025 | 4.74E-06 | 20.94 | |
| IL-13 | rs10995604 | | 10 | A | G | -0.1571 | 0.8587 | 0.0343 | 4.65E-06 | 20.98 | |
| IL-13 | rs150836197 | | 6 | T | C | 0.3283 | 0.0367 | 0.0713 | 4.13E-06 | 21.20 | |
| IL-13 | rs12199215 | | 6 | T | C | 0.1309 | 0.247 | 0.0284 | 4.04E-06 | 21.24 | |
| IL-13 | rs12623722 | | 2 | A | G | -0.1189 | 0.3054 | 0.0257 | 3.72E-06 | 21.40 | |
| IL-13 | rs75383097 | | 1 | C | G | -0.5369 | 0.0132 | 0.116 | 3.68E-06 | 21.42 | |
| IL-13 | rs139083458 | | 5 | T | C | 0.9995 | 0.0034 | 0.211 | 2.17E-06 | 22.44 | |
| IL-13 | rs145023524 | | 6 | A | G | 0.2815 | 0.0435 | 0.0588 | 1.69E-06 | 22.92 | |
| IL-13 | rs28442067 | | 3 | A | G | -0.1379 | 0.7832 | 0.0286 | 1.42E-06 | 23.25 | |
| IL-13 | rs76339001 | | 21 | A | T | -0.4375 | 0.9767 | 0.0886 | 7.90E-07 | 24.38 | |
| IL-13 | rs13209117 | | 6 | A | G | 0.1409 | 0.245 | 0.0284 | 7.00E-07 | 24.61 | |
| IL-13 | rs117795020 | | 9 | A | G | -0.3584 | 0.0304 | 0.0716 | 5.57E-07 | 25.06 | |
| IL-13 | rs138854806 | | 6 | A | G | -0.4204 | 0.0247 | 0.0839 | 5.42E-07 | 25.11 | |
| IL-13 | rs7747448 | | 6 | A | G | -0.1393 | 0.2585 | 0.0278 | 5.42E-07 | 25.11 | |
| IL-13 | rs77955971 | | 6 | A | C | 0.4408 | 0.0247 | 0.0868 | 3.81E-07 | 25.79 | |
| IL-13 | rs7757246 | | 6 | T | C | 0.2147 | 0.9092 | 0.0422 | 3.62E-07 | 25.88 | |
| IL-13 | rs9296421 | | 6 | T | G | 0.1814 | 0.842 | 0.0348 | 1.86E-07 | 27.17 | |
| IL-13 | rs13206012 | | 6 | A | G | -0.3719 | 0.3489 | 0.0263 | 2.13E-45 | 199.96 | |
| IL-16 | rs12577604 | | 11 | T | C | 0.4335 | 0.9818 | 0.0941 | 4.09E-06 | 21.22 | |
| IL-16 | rs117217798 | | 17 | T | C | -0.2064 | 0.0889 | 0.044 | 2.72E-06 | 22.00 | |
| IL-16 | rs142034902 | | 12 | A | G | -0.4367 | 0.9827 | 0.0925 | 2.35E-06 | 22.29 | |
| IL-16 | rs78042619 | | 9 | A | G | 0.55 | 0.9883 | 0.1158 | 2.04E-06 | 22.56 | |
| IL-16 | rs4976691 | | 5 | C | G | 0.1254 | 0.3268 | 0.026 | 1.41E-06 | 23.26 | |
| IL-16 | rs7097884 | | 10 | T | C | -0.1193 | 0.5647 | 0.0243 | 9.13E-07 | 24.10 | |
| IL-16 | rs35834666 | | 4 | T | C | -0.1729 | 0.8596 | 0.0348 | 6.75E-07 | 24.68 | |
| IL-16 | rs144691581 | | 15 | A | G | 0.4929 | 0.0197 | 0.0958 | 2.67E-07 | 26.47 | |
| IL-16 | rs142332135 | | 15 | A | G | -0.7646 | 0.0164 | 0.1082 | 1.59E-12 | 49.94 | |
| IL-16 | rs4778640 | | 15 | A | G | 0.7189 | 0.9848 | 0.0983 | 2.61E-13 | 53.48 | |
| IL-17 | rs78296352 | | 1 | T | G | 0.2949 | 0.0186 | 0.0645 | 4.83E-06 | 20.90 | |
| IL-17 | rs12735700 | | 1 | T | G | -0.0943 | 0.1925 | 0.0206 | 4.70E-06 | 20.96 | |
| IL-17 | rs9568764 | | 13 | C | G | 0.0825 | 0.2756 | 0.018 | 4.58E-06 | 21.01 | |
| IL-17 | rs11985957 | | 8 | A | G | 0.1511 | 0.0642 | 0.0329 | 4.38E-06 | 21.09 | |
| IL-17 | rs9519328 | | 13 | A | G | 0.5256 | 0.9845 | 0.1101 | 1.81E-06 | 22.79 | |
| IL-17 | rs77341831 | | 12 | T | C | 0.2263 | 0.9696 | 0.0473 | 1.72E-06 | 22.89 | |
| IL-17 | rs11640734 | | 16 | C | G | -0.115 | 0.8673 | 0.024 | 1.65E-06 | 22.96 | |
| IL-17 | rs61990749 | | 14 | C | G | 0.1124 | 0.1533 | 0.0226 | 6.58E-07 | 24.74 | |
| IL-17 | rs148562661 | | 6 | C | G | 0.2161 | 0.9605 | 0.0434 | 6.38E-07 | 24.79 | |
| IL-17 | rs17282552 | | 2 | T | C | -0.2026 | 0.952 | 0.0403 | 4.97E-07 | 25.27 | |
| IL-17 | rs3804749 | | 3 | T | C | -0.0923 | 0.6236 | 0.0167 | 3.26E-08 | 30.55 | |
| IL-18 | rs150005227 | | 5 | T | C | 0.4062 | 0.0185 | 0.0886 | 4.55E-06 | 21.02 | |
| IL-18 | rs764078 | | 11 | A | T | 0.1283 | 0.2239 | 0.0278 | 3.93E-06 | 21.30 | |
| IL-18 | rs7599125 | | 2 | A | G | 0.1109 | 0.569 | 0.0239 | 3.48E-06 | 21.53 | |
| IL-18 | rs12419156 | | 11 | T | C | 0.1556 | 0.8064 | 0.0335 | 3.40E-06 | 21.57 | |
| IL-18 | rs117371668 | | 16 | T | G | 0.3712 | 0.0254 | 0.0799 | 3.39E-06 | 21.58 | |
| IL-18 | rs77187209 | | 5 | T | C | -0.4859 | 0.9866 | 0.1041 | 3.05E-06 | 21.79 | |
| IL-18 | rs78716465 | | 20 | A | G | 0.3173 | 0.033 | 0.0679 | 2.97E-06 | 21.84 | |
| IL-18 | rs113214367 | | 11 | A | G | -0.278 | 0.0565 | 0.0593 | 2.76E-06 | 21.98 | |
| IL-18 | rs139468359 | | 5 | T | C | 0.5101 | 0.9866 | 0.1088 | 2.75E-06 | 21.98 | |
| IL-18 | rs76138275 | | 5 | T | C | 0.1227 | 0.2858 | 0.026 | 2.37E-06 | 22.27 | |
| IL-18 | rs139727649 | | 5 | T | C | -0.356 | 0.9745 | 0.0751 | 2.13E-06 | 22.47 | |
| IL-18 | rs4952239 | | 2 | A | T | -0.1156 | 0.3555 | 0.0242 | 1.78E-06 | 22.82 | |
| IL-18 | rs117266781 | | 7 | T | C | 0.7051 | 0.0073 | 0.1436 | 9.10E-07 | 24.11 | |
| IL-18 | rs1979967 | | 15 | T | C | 0.14 | 0.2157 | 0.0285 | 9.00E-07 | 24.13 | |
| IL-18 | rs78623212 | | 7 | T | C | 0.8322 | 0.0053 | 0.1676 | 6.86E-07 | 24.66 | |
| IL-18 | rs62312914 | | 4 | T | C | -0.1265 | 0.3726 | 0.025 | 4.19E-07 | 25.60 | |
| IL-18 | rs58701153 | | 6 | A | T | -0.1265 | 0.6586 | 0.0242 | 1.72E-07 | 27.32 | |
| IL-1B | rs61335305 | | 15 | A | C | 0.4333 | 0.0189 | 0.0928 | 3.02E-06 | 21.80 | |
| IL-1B | rs143319329 | | 7 | T | C | 0.4357 | 0.0209 | 0.093 | 2.80E-06 | 21.95 | |
| IL-1B | rs4786740 | | 16 | A | C | 0.1264 | 0.3913 | 0.0265 | 1.84E-06 | 22.75 | |
| IL-1B | rs62015704 | | 16 | A | G | 0.1786 | 0.8688 | 0.0372 | 1.58E-06 | 23.05 | |
| IL-1B | rs115242021 | | 1 | A | C | 0.2795 | 0.0601 | 0.0553 | 4.32E-07 | 25.55 | |
| IL-1RA | rs9985296 | | 3 | T | C | 0.1053 | 0.4758 | 0.0231 | 5.15E-06 | 20.78 | |
| IL-1RA | rs9623661 | | 22 | T | C | -0.1948 | 0.0903 | 0.0424 | 4.34E-06 | 21.11 | |
| IL-1RA | rs6699436 | | 1 | A | G | -0.1858 | 0.0995 | 0.0404 | 4.25E-06 | 21.15 | |
| IL-1RA | rs117181659 | | 22 | A | G | -0.2204 | 0.064 | 0.0478 | 4.01E-06 | 21.26 | |
| IL-1RA | rs35590641 | | 14 | C | G | -0.1167 | 0.3321 | 0.025 | 3.04E-06 | 21.79 | |
| IL-1RA | rs56134659 | | 3 | A | G | -0.1109 | 0.4835 | 0.0236 | 2.61E-06 | 22.08 | |
| IL-1RA | rs61335305 | | 15 | A | C | 0.4315 | 0.0182 | 0.0904 | 1.81E-06 | 22.78 | |
| IL-1RA | rs11869294 | | 17 | C | G | -0.2286 | 0.9205 | 0.047 | 1.15E-06 | 23.66 | |
| IL-1RA | rs1054402 | | 9 | T | C | 0.1325 | 0.2506 | 0.0269 | 8.41E-07 | 24.26 | |
| IL-2 | rs16836080 | | 3 | A | G | 0.1158 | 0.3355 | 0.0253 | 4.72E-06 | 20.95 | |
| IL-2 | rs4479767 | | 4 | A | G | 0.1821 | 0.8977 | 0.0392 | 3.39E-06 | 21.58 | |
| IL-2 | rs4634519 | | 7 | A | G | -0.1249 | 0.7379 | 0.0268 | 3.16E-06 | 21.72 | |
| IL-2 | rs62124990 | | 2 | T | G | -0.7013 | 0.0083 | 0.149 | 2.52E-06 | 22.15 | |
| IL-2 | rs2690020 | | 1 | A | G | 0.1158 | 0.5177 | 0.0245 | 2.28E-06 | 22.34 | |
| IL-2 | rs7615304 | | 3 | A | G | -0.1139 | 0.4531 | 0.024 | 2.08E-06 | 22.52 | |
| IL-2 | rs61335305 | | 15 | A | C | 0.4439 | 0.0184 | 0.0913 | 1.16E-06 | 23.64 | |
| IL-2 | rs13412535 | | 2 | A | G | 0.174 | 0.1886 | 0.0331 | 1.47E-07 | 27.63 | |
| IL-2RA | rs12789243 | | 11 | T | C | 0.1263 | 0.7571 | 0.0276 | 4.74E-06 | 20.94 | |
| IL-2RA | rs17624670 | | 8 | A | G | -0.125 | 0.2531 | 0.0273 | 4.68E-06 | 20.96 | |
| IL-2RA | rs56213152 | | 7 | T | C | 0.1269 | 0.7688 | 0.0271 | 2.83E-06 | 21.93 | |
| IL-2RA | rs11241559 | | 5 | T | G | -0.124 | 0.2643 | 0.0264 | 2.64E-06 | 22.06 | |
| IL-2RA | rs115360066 | | 5 | A | G | 0.1776 | 0.8882 | 0.0377 | 2.47E-06 | 22.19 | |
| IL-2RA | rs34353319 | | 10 | A | T | -0.1581 | 0.8176 | 0.0335 | 2.37E-06 | 22.27 | |
| IL-2RA | rs79100208 | | 3 | C | G | 0.8345 | 0.995 | 0.1758 | 2.07E-06 | 22.53 | |
| IL-2RA | rs9423654 | | 10 | C | G | -0.135 | 0.6609 | 0.0283 | 1.84E-06 | 22.76 | |
| IL-2RA | rs117244812 | | 17 | A | G | -0.7187 | 0.0079 | 0.1493 | 1.48E-06 | 23.17 | |
| IL-2RA | rs34037190 | | 10 | A | G | 0.4784 | 0.0217 | 0.0935 | 3.11E-07 | 26.18 | |
| IL-2RA | rs7078614 | | 10 | T | G | -0.1543 | 0.3549 | 0.0241 | 1.53E-10 | 40.99 | |
| IL-4 | rs117146485 | | 9 | T | C | -0.2856 | 0.9837 | 0.0625 | 4.89E-06 | 20.88 | |
| IL-4 | rs9506111 | | 13 | A | G | -0.1446 | 0.9326 | 0.0314 | 4.12E-06 | 21.21 | |
| IL-4 | rs79597994 | | 1 | T | C | -0.5855 | 0.0049 | 0.1271 | 4.09E-06 | 21.22 | |
| IL-4 | rs2708586 | | 7 | T | C | -0.0767 | 0.3411 | 0.0166 | 3.83E-06 | 21.35 | |
| IL-4 | rs2073438 | | 17 | A | G | 0.0847 | 0.2513 | 0.0183 | 3.68E-06 | 21.42 | |
| IL-4 | rs2346020 | | 3 | A | G | 0.079 | 0.6795 | 0.0169 | 2.95E-06 | 21.85 | |
| IL-4 | rs7613691 | | 3 | A | G | 0.1787 | 0.9482 | 0.0382 | 2.90E-06 | 21.88 | |
| IL-4 | rs116705532 | | 1 | T | G | -0.4675 | 0.9925 | 0.0978 | 1.75E-06 | 22.85 | |
| IL-4 | rs12238729 | | 9 | T | C | 0.5271 | 0.0162 | 0.1096 | 1.51E-06 | 23.13 | |
| IL-4 | rs56408830 | | 6 | A | G | -0.1794 | 0.0537 | 0.0365 | 8.88E-07 | 24.16 | |
| IL-4 | rs17713451 | | 7 | A | G | 0.1255 | 0.1149 | 0.0252 | 6.35E-07 | 24.80 | |
| IL-4 | rs1867282 | | 9 | T | C | 0.0808 | 0.4148 | 0.0162 | 6.11E-07 | 24.88 | |
| IL-4 | rs9941733 | | 20 | A | G | 0.1156 | 0.8355 | 0.0229 | 4.46E-07 | 25.48 | |
| IL-4 | rs12640583 | | 4 | T | G | -0.1104 | 0.1631 | 0.0214 | 2.48E-07 | 26.61 | |
| IL-5 | rs9309063 | | 2 | T | G | -0.1119 | 0.4763 | 0.0245 | 4.94E-06 | 20.86 | |
| IL-5 | rs111736126 | | 2 | C | G | -0.3973 | 0.9792 | 0.0867 | 4.60E-06 | 21.00 | |
| IL-5 | rs74811276 | | 14 | A | G | 0.217 | 0.0761 | 0.0471 | 4.08E-06 | 21.23 | |
| IL-5 | rs10178043 | | 2 | T | G | 0.2579 | 0.949 | 0.0553 | 3.11E-06 | 21.75 | |
| IL-5 | rs73040118 | | 19 | T | C | 0.2294 | 0.9285 | 0.049 | 2.85E-06 | 21.92 | |
| IL-5 | rs28793375 | | 8 | T | C | 0.1697 | 0.1298 | 0.0362 | 2.76E-06 | 21.98 | |
| IL-5 | rs148634917 | | 1 | A | G | -0.517 | 0.986 | 0.1087 | 1.97E-06 | 22.62 | |
| IL-5 | rs72831687 | | 6 | A | G | -0.5337 | 0.0137 | 0.1104 | 1.34E-06 | 23.37 | |
| IL-5 | rs7739450 | | 6 | A | G | -0.1295 | 0.5158 | 0.0256 | 4.22E-07 | 25.59 | |
| IL-6 | rs10982193 | | 9 | A | G | -0.0793 | 0.2822 | 0.0174 | 5.18E-06 | 20.77 | |
| IL-6 | rs113098456 | | 2 | A | G | -0.1553 | 0.0725 | 0.0339 | 4.62E-06 | 20.99 | |
| IL-6 | rs10910395 | | 1 | A | T | -0.108 | 0.8729 | 0.0235 | 4.31E-06 | 21.12 | |
| IL-6 | rs4684700 | | 3 | T | C | -0.0747 | 0.5142 | 0.0162 | 4.01E-06 | 21.26 | |
| IL-6 | rs11732981 | | 4 | A | C | 0.0722 | 0.5119 | 0.0156 | 3.69E-06 | 21.42 | |
| IL-6 | rs114373846 | | 3 | T | C | 0.4196 | 0.0085 | 0.0905 | 3.54E-06 | 21.50 | |
| IL-6 | rs1333040 | | 9 | T | C | 0.0747 | 0.4508 | 0.0157 | 1.96E-06 | 22.64 | |
| IL-6 | rs76856708 | | 16 | T | C | 0.336 | 0.9856 | 0.0697 | 1.43E-06 | 23.24 | |
| IL-6 | rs113600793 | | 17 | A | C | 0.1736 | 0.0623 | 0.0359 | 1.33E-06 | 23.38 | |
| IL-6 | rs73273528 | | 20 | T | C | 0.268 | 0.0211 | 0.0553 | 1.26E-06 | 23.49 | |
| IL-6 | rs13412535 | | 2 | A | G | -0.1186 | 0.189 | 0.0214 | 2.99E-08 | 30.71 | |
| IL-7 | rs28793375 | | 8 | T | C | 0.1644 | 0.1305 | 0.036 | 4.96E-06 | 20.85 | |
| IL-7 | rs115215018 | | 4 | T | C | 0.5985 | 0.0101 | 0.1308 | 4.75E-06 | 20.94 | |
| IL-7 | rs7155170 | | 14 | A | T | -0.1236 | 0.2744 | 0.027 | 4.70E-06 | 20.96 | |
| IL-7 | rs78346957 | | 10 | A | G | 0.4632 | 0.0161 | 0.1008 | 4.32E-06 | 21.12 | |
| IL-7 | rs142397827 | | 5 | A | C | 0.4592 | 0.0178 | 0.0994 | 3.84E-06 | 21.34 | |
| IL-7 | rs218238 | | 4 | A | T | 0.1319 | 0.7729 | 0.0284 | 3.41E-06 | 21.57 | |
| IL-7 | rs1374279 | | 2 | A | T | 0.1625 | 0.143 | 0.0347 | 2.83E-06 | 21.93 | |
| IL-7 | rs117509142 | | 8 | T | C | -0.3213 | 0.9569 | 0.0684 | 2.64E-06 | 22.07 | |
| IL-7 | rs11757972 | | 6 | T | C | 0.121 | 0.4776 | 0.0257 | 2.50E-06 | 22.17 | |
| IL-7 | rs77981494 | | 16 | T | C | -0.5201 | 0.984 | 0.1055 | 8.23E-07 | 24.30 | |
| IL-7 | rs62006410 | | 14 | T | C | -0.1492 | 0.245 | 0.0302 | 7.80E-07 | 24.41 | |
| IL-7 | rs17091524 | | 14 | T | C | 0.5092 | 0.9849 | 0.1015 | 5.26E-07 | 25.17 | |
| IL-7 | rs7739450 | | 6 | A | G | -0.2907 | 0.5167 | 0.0252 | 8.72E-31 | 133.07 | |
| IL-8 | rs75840288 | | 16 | A | C | 0.5125 | 0.9839 | 0.1121 | 4.84E-06 | 20.90 | |
| IL-8 | rs116726256 | | 2 | T | C | -0.2247 | 0.9343 | 0.0489 | 4.33E-06 | 21.11 | |
| IL-8 | rs183628733 | | 1 | T | C | 0.6547 | 0.9917 | 0.1417 | 3.83E-06 | 21.35 | |
| IL-8 | rs2673604 | | 8 | A | C | -0.118 | 0.6829 | 0.0254 | 3.39E-06 | 21.58 | |
| IL-8 | rs12912642 | | 15 | A | G | 0.1168 | 0.3302 | 0.0251 | 3.27E-06 | 21.65 | |
| IL-8 | rs113487695 | | 7 | A | C | -0.6129 | 0.9914 | 0.1292 | 2.10E-06 | 22.50 | |
| IL-8 | rs3786107 | | 17 | A | G | 0.2463 | 0.9287 | 0.0517 | 1.90E-06 | 22.70 | |
| IL-8 | rs12075 | | 1 | A | G | 0.1148 | 0.5353 | 0.0235 | 1.03E-06 | 23.86 | |
| IL-9 | rs3736858 | | 13 | C | G | -0.1351 | 0.7976 | 0.0291 | 3.44E-06 | 21.55 | |
| IL-9 | rs41294750 | | 1 | T | C | 0.3442 | 0.0308 | 0.0736 | 2.92E-06 | 21.87 | |
| IL-9 | rs1259728 | | 12 | A | G | -0.2381 | 0.0554 | 0.0507 | 2.65E-06 | 22.05 | |
| IL-9 | rs73443903 | | 6 | A | C | 0.2162 | 0.0685 | 0.046 | 2.60E-06 | 22.09 | |
| IL-9 | rs117807175 | | 14 | C | G | -0.5225 | 0.0121 | 0.1106 | 2.31E-06 | 22.32 | |
| IL-9 | rs4880409 | | 10 | T | C | -0.3552 | 0.9664 | 0.0716 | 7.02E-07 | 24.61 | |
| IP-10 | rs4862110 | | 4 | T | C | -0.1453 | 0.771 | 0.0318 | 4.90E-06 | 20.88 | |
| IP-10 | rs113183470 | | 6 | A | T | -0.2414 | 0.946 | 0.0524 | 4.09E-06 | 21.22 | |
| IP-10 | rs143799975 | | 4 | A | G | -0.7551 | 0.9949 | 0.1638 | 4.03E-06 | 21.25 | |
| IP-10 | rs12714300 | | 2 | A | T | -0.1573 | 0.1378 | 0.0338 | 3.26E-06 | 21.66 | |
| IP-10 | rs4859940 | | 4 | C | G | -0.1204 | 0.295 | 0.0258 | 3.06E-06 | 21.78 | |
| IP-10 | rs75970138 | | 9 | A | G | -0.4845 | 0.0139 | 0.1037 | 2.98E-06 | 21.83 | |
| IP-10 | rs8112618 | | 19 | A | G | 0.1388 | 0.1841 | 0.0297 | 2.96E-06 | 21.84 | |
| IP-10 | rs79848609 | | 15 | A | C | 0.2514 | 0.9449 | 0.0535 | 2.61E-06 | 22.08 | |
| IP-10 | rs7645625 | | 3 | T | G | -0.1116 | 0.5724 | 0.0236 | 2.26E-06 | 22.36 | |
| IP-10 | rs34383175 | | 8 | T | C | -0.3196 | 0.035 | 0.0653 | 9.86E-07 | 23.95 | |
| IP-10 | rs113831257 | | 4 | A | G | 0.3639 | 0.0417 | 0.0641 | 1.37E-08 | 32.23 | |
| MCP-1-MCAF | | rs143815843 | 2 | A | G | -0.2049 | 0.0315 | 0.0447 | 4.56E-06 | 21.01 | |
| MCP-1-MCAF | | rs188998783 | 19 | T | C | -0.5659 | 0.0066 | 0.1233 | 4.44E-06 | 21.06 | |
| MCP-1-MCAF | | rs145155829 | 1 | T | C | -0.2125 | 0.037 | 0.0461 | 4.04E-06 | 21.25 | |
| MCP-1-MCAF | | rs12062235 | 1 | T | G | 0.1477 | 0.9377 | 0.032 | 3.92E-06 | 21.30 | |
| MCP-1-MCAF | | rs111995966 | 2 | T | G | 0.1428 | 0.9297 | 0.0309 | 3.81E-06 | 21.36 | |
| MCP-1-MCAF | | rs16837903 | 1 | A | G | -0.1104 | 0.1237 | 0.0238 | 3.51E-06 | 21.52 | |
| MCP-1-MCAF | | rs72705803 | 9 | A | G | -0.2188 | 0.9694 | 0.047 | 3.23E-06 | 21.67 | |
| MCP-1-MCAF | | rs7978037 | 12 | A | T | 0.0746 | 0.3863 | 0.016 | 3.12E-06 | 21.74 | |
| MCP-1-MCAF | | rs2820126 | 1 | T | G | 0.0907 | 0.7898 | 0.0193 | 2.61E-06 | 22.09 | |
| MCP-1-MCAF | | rs7197349 | 16 | A | G | 0.0971 | 0.8124 | 0.0206 | 2.43E-06 | 22.22 | |
| MCP-1-MCAF | | rs11920996 | 3 | T | C | 0.1805 | 0.051 | 0.0376 | 1.58E-06 | 23.05 | |
| MCP-1-MCAF | | rs34190208 | 3 | T | C | 0.1052 | 0.1511 | 0.0219 | 1.56E-06 | 23.08 | |
| MCP-1-MCAF | | rs56212190 | 1 | T | C | 0.1799 | 0.0519 | 0.0372 | 1.32E-06 | 23.39 | |
| MCP-1-MCAF | | rs9317045 | 13 | A | C | 0.1157 | 0.8618 | 0.0235 | 8.51E-07 | 24.24 | |
| MCP-1-MCAF | | rs77116118 | 3 | T | C | -0.4239 | 0.9892 | 0.0824 | 2.68E-07 | 26.47 | |
| MCP-1-MCAF | | rs112313229 | 3 | A | G | -0.1652 | 0.0708 | 0.0312 | 1.19E-07 | 28.04 | |
| MCP-1-MCAF | | rs3026968 | 1 | T | C | -0.0896 | 0.3195 | 0.0169 | 1.15E-07 | 28.11 | |
| MCP-1-MCAF | | rs12493953 | 3 | A | G | -0.0948 | 0.3224 | 0.0172 | 3.56E-08 | 30.38 | |
| MCP-1-MCAF | | rs79939301 | 3 | A | G | 0.1449 | 0.1081 | 0.0255 | 1.33E-08 | 32.29 | |
| MCP-1-MCAF | | rs80108502 | 3 | T | C | 0.2463 | 0.0391 | 0.0409 | 1.72E-09 | 36.26 | |
| MCP-1-MCAF | | rs2229593 | 3 | T | C | 0.2624 | 0.0385 | 0.0405 | 9.23E-11 | 41.98 | |
| MCP-1-MCAF | | rs863002 | 1 | T | C | 0.1135 | 0.4077 | 0.0158 | 6.79E-13 | 51.60 | |
| MCP-3 | rs3129806 | | 9 | T | C | -0.1975 | 0.5729 | 0.0433 | 5.09E-06 | 20.80 | |
| MCP-3 | rs6993671 | | 8 | T | C | 0.2041 | 0.5588 | 0.0443 | 4.08E-06 | 21.23 | |
| MCP-3 | rs7275485 | | 21 | T | C | -0.2218 | 0.2711 | 0.0481 | 4.00E-06 | 21.26 | |
| MCP-3 | rs28394764 | | 4 | A | T | 0.597 | 0.9696 | 0.1282 | 3.21E-06 | 21.69 | |
| MCP-3 | rs117286643 | | 8 | A | G | 0.6934 | 0.0225 | 0.1474 | 2.55E-06 | 22.13 | |
| MCP-3 | rs10892381 | | 11 | T | C | 0.2432 | 0.6629 | 0.0473 | 2.72E-07 | 26.44 | |
| M-CSF | rs11963606 | | 6 | C | G | -0.5353 | 0.9826 | 0.117 | 4.76E-06 | 20.93 | |
| M-CSF | rs116887628 | | 8 | A | G | -0.2741 | 0.0611 | 0.0598 | 4.57E-06 | 21.01 | |
| M-CSF | rs72723242 | | 5 | T | G | -0.4969 | 0.0177 | 0.1083 | 4.47E-06 | 21.05 | |
| M-CSF | rs12962919 | | 18 | T | C | 0.3025 | 0.0644 | 0.0659 | 4.43E-06 | 21.07 | |
| M-CSF | rs9626985 | | 22 | T | C | 0.2277 | 0.0936 | 0.0496 | 4.42E-06 | 21.07 | |
| M-CSF | rs147378920 | | 1 | A | G | -0.6064 | 0.9871 | 0.1318 | 4.21E-06 | 21.17 | |
| M-CSF | rs9387100 | | 6 | T | C | -0.135 | 0.4291 | 0.029 | 3.24E-06 | 21.67 | |
| M-CSF | rs139457375 | | 8 | A | C | -0.4047 | 0.9668 | 0.0854 | 2.15E-06 | 22.46 | |
| M-CSF | rs34089869 | | 2 | T | C | 0.2194 | 0.1039 | 0.0462 | 2.05E-06 | 22.55 | |
| M-CSF | rs116274860 | | 3 | T | G | 0.8262 | 0.9908 | 0.1739 | 2.02E-06 | 22.57 | |
| M-CSF | rs117867915 | | 18 | T | C | 0.5224 | 0.9793 | 0.1096 | 1.88E-06 | 22.72 | |
| M-CSF | rs62294910 | | 3 | A | G | 0.3472 | 0.0504 | 0.0687 | 4.33E-07 | 25.54 | |
| MIF | rs35890933 | | 19 | T | G | 0.1676 | 0.8474 | 0.0365 | 4.39E-06 | 21.08 | |
| MIF | rs11551183 | | 16 | C | G | 0.3666 | 0.9756 | 0.0795 | 4.00E-06 | 21.26 | |
| MIF | rs3814097 | | 7 | A | G | -0.1163 | 0.5533 | 0.0251 | 3.60E-06 | 21.47 | |
| MIF | rs2294689 | | 6 | C | G | -0.1338 | 0.3018 | 0.0287 | 3.13E-06 | 21.73 | |
| MIF | rs113218956 | | 22 | A | G | -0.8789 | 0.0044 | 0.1876 | 2.80E-06 | 21.95 | |
| MIF | rs141009259 | | 2 | T | C | -0.6194 | 0.9886 | 0.1285 | 1.43E-06 | 23.23 | |
| MIF | rs35792361 | | 4 | A | G | -0.2586 | 0.0591 | 0.0527 | 9.25E-07 | 24.08 | |
| MIF | rs12594190 | | 15 | A | G | 0.1321 | 0.7008 | 0.0266 | 6.83E-07 | 24.66 | |
| MIF | rs78098071 | | 5 | T | C | -0.4583 | 0.9809 | 0.0915 | 5.48E-07 | 25.09 | |
| MIF | rs1007888 | | 22 | T | C | -0.1275 | 0.6128 | 0.0245 | 1.95E-07 | 27.08 | |
| MIG | rs113302091 | | 14 | T | C | 0.2537 | 0.0468 | 0.0553 | 4.48E-06 | 21.05 | |
| MIG | rs139010077 | | 3 | T | C | 0.4337 | 0.0169 | 0.0943 | 4.24E-06 | 21.15 | |
| MIG | rs9456663 | | 6 | T | C | -0.1186 | 0.6702 | 0.0255 | 3.30E-06 | 21.63 | |
| MIG | rs111607343 | | 19 | A | G | -0.5235 | 0.0134 | 0.1119 | 2.89E-06 | 21.89 | |
| MIG | rs13143163 | | 4 | C | G | 0.2735 | 0.0512 | 0.0582 | 2.61E-06 | 22.08 | |
| MIG | rs11177248 | | 12 | A | G | 0.3157 | 0.0351 | 0.0667 | 2.21E-06 | 22.40 | |
| MIG | rs192433162 | | 10 | A | G | -0.8045 | 0.0061 | 0.1676 | 1.59E-06 | 23.04 | |
| MIG | rs8127917 | | 21 | T | G | 0.2382 | 0.0608 | 0.0492 | 1.29E-06 | 23.44 | |
| MIG | rs816960 | | 13 | T | C | -0.1179 | 0.3692 | 0.0242 | 1.11E-06 | 23.74 | |
| MIG | rs3733233 | | 4 | T | C | 0.1223 | 0.6588 | 0.025 | 9.98E-07 | 23.93 | |
| MIG | rs62562991 | | 9 | A | G | 0.6239 | 0.0097 | 0.1259 | 7.21E-07 | 24.56 | |
| MIG | rs6679677 | | 1 | A | C | 0.1628 | 0.1458 | 0.0327 | 6.40E-07 | 24.79 | |
| MIG | rs10266753 | | 7 | T | C | -0.2016 | 0.9109 | 0.0397 | 3.81E-07 | 25.79 | |
| MIG | rs191555775 | | 6 | A | T | 0.2279 | 0.909 | 0.0412 | 3.17E-08 | 30.60 | |
| MIP-1A | rs6956239 | | 7 | T | C | 0.119 | 0.2927 | 0.026 | 4.72E-06 | 20.95 | |
| MIP-1A | rs117506943 | | 11 | T | C | 0.3128 | 0.0323 | 0.0682 | 4.51E-06 | 21.04 | |
| MIP-1A | rs116615337 | | 1 | A | G | 0.1286 | 0.4232 | 0.0278 | 3.73E-06 | 21.40 | |
| MIP-1A | rs12159394 | | 22 | A | G | -0.1708 | 0.1183 | 0.0366 | 3.06E-06 | 21.78 | |
| MIP-1A | rs6900267 | | 6 | A | C | -0.2472 | 0.9308 | 0.0515 | 1.59E-06 | 23.04 | |
| MIP-1A | rs57786342 | | 14 | A | G | 0.139 | 0.2336 | 0.0283 | 9.03E-07 | 24.12 | |
| MIP-1B | rs1437220 | | 17 | T | C | 0.1437 | 0.9207 | 0.0315 | 5.07E-06 | 20.81 | |
| MIP-1B | rs854222 | | 3 | A | C | 0.0809 | 0.2696 | 0.0177 | 4.86E-06 | 20.89 | |
| MIP-1B | rs28393318 | | 4 | A | G | -0.1076 | 0.8758 | 0.0235 | 4.68E-06 | 20.96 | |
| MIP-1B | rs145526037 | | 3 | T | G | -0.1863 | 0.9604 | 0.0406 | 4.46E-06 | 21.06 | |
| MIP-1B | rs112337896 | | 17 | A | G | 0.2891 | 0.0288 | 0.063 | 4.46E-06 | 21.06 | |
| MIP-1B | rs117657747 | | 18 | A | G | 0.2089 | 0.0526 | 0.0453 | 4.00E-06 | 21.27 | |
| MIP-1B | rs281728 | | 8 | A | C | -0.079 | 0.7058 | 0.0171 | 3.84E-06 | 21.34 | |
| MIP-1B | rs141793738 | | 17 | A | G | 0.1815 | 0.0432 | 0.0389 | 3.07E-06 | 21.77 | |
| MIP-1B | rs2314809 | | 17 | T | C | -0.0735 | 0.5305 | 0.0157 | 2.85E-06 | 21.92 | |
| MIP-1B | rs116237296 | | 1 | A | G | 0.5284 | 0.0054 | 0.1115 | 2.15E-06 | 22.46 | |
| MIP-1B | rs9793308 | | 1 | A | G | 0.0842 | 0.6036 | 0.0177 | 1.96E-06 | 22.63 | |
| MIP-1B | rs9838883 | | 3 | T | C | 0.079 | 0.3399 | 0.0166 | 1.95E-06 | 22.65 | |
| MIP-1B | rs6908843 | | 6 | A | G | 0.0997 | 0.1684 | 0.0209 | 1.84E-06 | 22.76 | |
| MIP-1B | rs72799710 | | 5 | T | C | -0.1037 | 0.1514 | 0.0217 | 1.76E-06 | 22.84 | |
| MIP-1B | rs2673059 | | 3 | T | C | 0.0921 | 0.7787 | 0.0192 | 1.61E-06 | 23.01 | |
| MIP-1B | rs76582507 | | 9 | A | G | 0.3259 | 0.0156 | 0.0676 | 1.43E-06 | 23.24 | |
| MIP-1B | rs4796110 | | 17 | A | G | 0.1244 | 0.8879 | 0.0256 | 1.18E-06 | 23.61 | |
| MIP-1B | rs159309 | | 17 | T | C | 0.1254 | 0.1102 | 0.0258 | 1.17E-06 | 23.62 | |
| MIP-1B | rs17138331 | | 7 | A | G | -0.1434 | 0.9092 | 0.0295 | 1.17E-06 | 23.63 | |
| MIP-1B | rs117503347 | | 17 | T | C | 0.3039 | 0.98 | 0.062 | 9.51E-07 | 24.03 | |
| MIP-1B | rs35933743 | | 17 | T | G | -0.1183 | 0.8438 | 0.0238 | 6.68E-07 | 24.71 | |
| MIP-1B | rs2276857 | | 3 | T | C | -0.1283 | 0.1356 | 0.0257 | 5.97E-07 | 24.92 | |
| MIP-1B | rs4795931 | | 17 | A | G | -0.0865 | 0.3193 | 0.0173 | 5.73E-07 | 25.00 | |
| MIP-1B | rs12951603 | | 17 | A | G | -0.1132 | 0.1492 | 0.0225 | 4.88E-07 | 25.31 | |
| MIP-1B | rs80322601 | | 17 | T | C | -0.1915 | 0.9468 | 0.038 | 4.67E-07 | 25.40 | |
| MIP-1B | rs56083628 | | 17 | T | C | -0.1297 | 0.1054 | 0.0257 | 4.50E-07 | 25.47 | |
| MIP-1B | rs72791296 | | 5 | T | C | 0.2364 | 0.0349 | 0.0466 | 3.92E-07 | 25.73 | |
| MIP-1B | rs9911839 | | 17 | T | G | -0.1377 | 0.9011 | 0.027 | 3.40E-07 | 26.01 | |
| MIP-1B | rs951814 | | 17 | A | G | 0.1614 | 0.07 | 0.0315 | 2.99E-07 | 26.25 | |
| MIP-1B | rs146565944 | | 17 | T | C | 0.2863 | 0.0235 | 0.0558 | 2.88E-07 | 26.33 | |
| MIP-1B | rs2131092 | | 3 | A | G | -0.1278 | 0.1103 | 0.0248 | 2.56E-07 | 26.56 | |
| MIP-1B | rs74979864 | | 7 | A | T | -0.3184 | 0.0169 | 0.0613 | 2.06E-07 | 26.98 | |
| MIP-1B | rs117010890 | | 17 | T | C | -0.2043 | 0.9347 | 0.0393 | 2.01E-07 | 27.02 | |
| MIP-1B | rs11716293 | | 3 | C | G | 0.0986 | 0.7698 | 0.0189 | 1.82E-07 | 27.22 | |
| MIP-1B | rs117139712 | | 17 | T | C | -0.2669 | 0.0277 | 0.05 | 9.40E-08 | 28.49 | |
| MIP-1B | rs34437725 | | 17 | T | C | -0.2571 | 0.9697 | 0.0481 | 9.04E-08 | 28.57 | |
| MIP-1B | rs2373048 | | 3 | A | T | -0.116 | 0.1789 | 0.0215 | 6.84E-08 | 29.11 | |
| MIP-1B | rs117394484 | | 17 | T | C | -0.4266 | 0.0126 | 0.0786 | 5.72E-08 | 29.46 | |
| MIP-1B | rs72829264 | | 17 | A | G | -0.1517 | 0.8833 | 0.0277 | 4.34E-08 | 29.99 | |
| MIP-1B | rs79088462 | | 17 | T | C | -0.3214 | 0.9782 | 0.0584 | 3.72E-08 | 30.29 | |
| MIP-1B | rs323877 | | 3 | C | G | -0.0965 | 0.6443 | 0.0172 | 2.02E-08 | 31.48 | |
| MIP-1B | rs2376263 | | 17 | A | G | 0.1053 | 0.7795 | 0.0187 | 1.79E-08 | 31.71 | |
| MIP-1B | rs41341749 | | 17 | A | G | -0.1656 | 0.9203 | 0.0294 | 1.77E-08 | 31.73 | |
| MIP-1B | rs117084209 | | 17 | C | G | 0.2445 | 0.0365 | 0.0428 | 1.11E-08 | 32.63 | |
| MIP-1B | rs72825991 | | 17 | A | G | -0.179 | 0.0709 | 0.0312 | 9.63E-09 | 32.92 | |
| MIP-1B | rs41502550 | | 3 | T | C | 0.1266 | 0.8499 | 0.022 | 8.69E-09 | 33.11 | |
| MIP-1B | rs72820112 | | 17 | T | C | 0.1059 | 0.7412 | 0.0183 | 7.17E-09 | 33.49 | |
| MIP-1B | rs72820246 | | 17 | T | G | -0.0983 | 0.4163 | 0.0167 | 3.95E-09 | 34.65 | |
| MIP-1B | rs191600590 | | 3 | A | T | 0.1449 | 0.1326 | 0.0246 | 3.86E-09 | 34.69 | |
| MIP-1B | rs62079535 | | 17 | A | G | 0.2309 | 0.9572 | 0.0389 | 2.93E-09 | 35.23 | |
| MIP-1B | rs28856610 | | 17 | T | C | -0.3074 | 0.031 | 0.0495 | 5.30E-10 | 38.57 | |
| MIP-1B | rs939408 | | 3 | A | C | -0.1007 | 0.3938 | 0.016 | 3.10E-10 | 39.61 | |
| MIP-1B | rs148561432 | | 17 | A | G | -0.2691 | 0.0454 | 0.0407 | 3.80E-11 | 43.72 | |
| MIP-1B | rs12452320 | | 17 | A | C | 0.2139 | 0.0716 | 0.0319 | 2.01E-11 | 44.96 | |
| MIP-1B | rs71381491 | | 17 | A | C | 0.3777 | 0.9746 | 0.0561 | 1.67E-11 | 45.33 | |
| MIP-1B | rs873944 | | 17 | T | C | 0.2392 | 0.0726 | 0.0322 | 1.10E-13 | 55.18 | |
| MIP-1B | rs111942332 | | 17 | T | G | -0.4711 | 0.9782 | 0.0571 | 1.58E-16 | 68.07 | |
| MIP-1B | rs76863419 | | 17 | T | G | -0.2833 | 0.073 | 0.0341 | 9.74E-17 | 69.02 | |
| MIP-1B | rs76842834 | | 17 | T | C | -0.4207 | 0.0371 | 0.0471 | 4.18E-19 | 79.78 | |
| MIP-1B | rs41290648 | | 3 | A | G | 0.2231 | 0.1266 | 0.0239 | 1.01E-20 | 87.14 | |
| MIP-1B | rs117453826 | | 17 | A | G | -0.5907 | 0.981 | 0.0591 | 1.60E-23 | 99.90 | |
| MIP-1B | rs114933663 | | 3 | T | C | 0.3178 | 0.0694 | 0.0307 | 4.11E-25 | 107.16 | |
| PDGF-BB | rs35859699 | | 4 | A | G | -0.3854 | 0.0109 | 0.0838 | 4.24E-06 | 21.15 | |
| PDGF-BB | rs13037046 | | 20 | A | T | -0.0948 | 0.1871 | 0.0206 | 4.19E-06 | 21.18 | |
| PDGF-BB | rs11766649 | | 7 | A | G | 0.0902 | 0.8018 | 0.0196 | 4.18E-06 | 21.18 | |
| PDGF-BB | rs192743385 | | 15 | T | C | 0.1807 | 0.0518 | 0.0392 | 4.03E-06 | 21.25 | |
| PDGF-BB | rs73162807 | | 3 | A | C | -0.2313 | 0.0259 | 0.0499 | 3.56E-06 | 21.49 | |
| PDGF-BB | rs62191444 | | 20 | T | G | -0.112 | 0.1492 | 0.0239 | 2.78E-06 | 21.96 | |
| PDGF-BB | rs9924851 | | 16 | C | G | 0.0767 | 0.3564 | 0.0163 | 2.53E-06 | 22.14 | |
| PDGF-BB | rs72958564 | | 2 | A | T | -0.4097 | 0.9901 | 0.0864 | 2.12E-06 | 22.49 | |
| PDGF-BB | rs2643354 | | 15 | A | G | 0.1251 | 0.8921 | 0.0261 | 1.64E-06 | 22.97 | |
| PDGF-BB | rs10512952 | | 5 | T | C | -0.2816 | 0.9819 | 0.0587 | 1.61E-06 | 23.01 | |
| PDGF-BB | rs116154010 | | 2 | T | C | 0.3225 | 0.0179 | 0.0662 | 1.11E-06 | 23.73 | |
| PDGF-BB | rs12289510 | | 11 | A | G | -0.0772 | 0.4767 | 0.0158 | 1.03E-06 | 23.87 | |
| PDGF-BB | rs72972467 | | 2 | C | G | -0.1616 | 0.0623 | 0.0328 | 8.36E-07 | 24.27 | |
| PDGF-BB | rs6910518 | | 6 | T | G | 0.0806 | 0.5924 | 0.0162 | 6.51E-07 | 24.75 | |
| PDGF-BB | rs34131731 | | 2 | A | T | 0.2083 | 0.9594 | 0.0411 | 4.02E-07 | 25.69 | |
| PDGF-BB | rs12615784 | | 2 | T | C | -0.1003 | 0.7849 | 0.0193 | 2.03E-07 | 27.01 | |
| PDGF-BB | rs147862316 | | 2 | T | C | 0.2279 | 0.0371 | 0.0411 | 2.94E-08 | 30.75 | |
| PDGF-BB | rs111996132 | | 2 | A | C | -0.3127 | 0.9789 | 0.0561 | 2.49E-08 | 31.07 | |
| PDGF-BB | rs7170232 | | 15 | T | C | 0.1609 | 0.219 | 0.0189 | 1.69E-17 | 72.48 | |
| RANTES | rs2731672 | | 5 | T | C | -0.1242 | 0.267 | 0.0272 | 4.97E-06 | 20.85 | |
| RANTES | rs148526102 | | 19 | T | C | -0.3798 | 0.0215 | 0.083 | 4.74E-06 | 20.94 | |
| RANTES | rs62438851 | | 6 | A | G | -0.1904 | 0.8961 | 0.0413 | 4.02E-06 | 21.25 | |
| RANTES | rs11873385 | | 18 | A | G | -0.2582 | 0.9466 | 0.0552 | 2.90E-06 | 21.88 | |
| RANTES | rs7170339 | | 15 | C | G | -0.4283 | 0.0197 | 0.0904 | 2.16E-06 | 22.45 | |
| RANTES | rs118096511 | | 13 | T | C | -0.3374 | 0.9696 | 0.0709 | 1.95E-06 | 22.65 | |
| RANTES | rs4795087 | | 17 | C | G | 0.1494 | 0.8188 | 0.0312 | 1.68E-06 | 22.93 | |
| RANTES | rs78050316 | | 2 | A | C | 0.4202 | 0.0204 | 0.0859 | 1.00E-06 | 23.93 | |
| RANTES | rs72793342 | | 16 | A | G | -0.1505 | 0.2003 | 0.0307 | 9.47E-07 | 24.03 | |
| RANTES | rs10505135 | | 8 | T | C | 0.1315 | 0.3704 | 0.0252 | 1.81E-07 | 27.23 | |
| SCF | rs8045376 | | 16 | A | G | -0.3126 | 0.0144 | 0.068 | 4.28E-06 | 21.13 | |
| SCF | rs72678285 | | 14 | A | T | 0.1062 | 0.8512 | 0.0231 | 4.28E-06 | 21.14 | |
| SCF | rs12345108 | | 9 | T | C | -0.0772 | 0.3395 | 0.0167 | 3.79E-06 | 21.37 | |
| SCF | rs72832071 | | 16 | A | G | 0.2238 | 0.9732 | 0.0482 | 3.43E-06 | 21.56 | |
| SCF | rs113127926 | | 14 | A | C | 0.1974 | 0.0418 | 0.0418 | 2.33E-06 | 22.30 | |
| SCF | rs10800449 | | 1 | A | C | 0.0851 | 0.2859 | 0.0179 | 1.99E-06 | 22.60 | |
| SCF | rs1536480 | | 9 | T | C | 0.081 | 0.3204 | 0.0167 | 1.23E-06 | 23.53 | |
| SCF | rs117721699 | | 9 | C | G | -0.2392 | 0.0265 | 0.0484 | 7.73E-07 | 24.42 | |
| SCF | rs78666213 | | 4 | T | G | -0.2845 | 0.9797 | 0.0574 | 7.18E-07 | 24.57 | |
| SCF | rs13412535 | | 2 | A | G | -0.1065 | 0.1892 | 0.0213 | 5.73E-07 | 25.00 | |
| SCF | rs138538809 | | 8 | T | C | -0.5788 | 0.0052 | 0.1139 | 3.74E-07 | 25.82 | |
| SCGF-B | rs1149926 | | 10 | T | C | -0.3458 | 0.0239 | 0.0749 | 3.90E-06 | 21.32 | |
| SCGF-B | rs112346514 | | 19 | T | C | -0.3261 | 0.0302 | 0.0703 | 3.51E-06 | 21.52 | |
| SCGF-B | rs118003677 | | 12 | T | C | -0.3654 | 0.9775 | 0.0786 | 3.34E-06 | 21.61 | |
| SCGF-B | rs12118918 | | 1 | A | G | -0.1631 | 0.8669 | 0.035 | 3.16E-06 | 21.72 | |
| SCGF-B | rs12480722 | | 20 | T | C | 0.1654 | 0.8752 | 0.0353 | 2.79E-06 | 21.95 | |
| SCGF-B | rs13287050 | | 9 | A | T | -0.121 | 0.7202 | 0.0263 | 4.21E-06 | 21.17 | |
| SCGF-B | rs13866 | | 19 | T | C | -0.1647 | 0.2622 | 0.028 | 4.05E-09 | 34.60 | |
| SCGF-B | rs139413256 | | 7 | A | G | -0.5174 | 0.0139 | 0.1076 | 1.52E-06 | 23.12 | |
| SCGF-B | rs143829871 | | 3 | T | C | -0.1866 | 0.9047 | 0.0399 | 2.92E-06 | 21.87 | |
| SCGF-B | rs144724875 | | 19 | T | C | 0.5381 | 0.0274 | 0.0829 | 8.53E-11 | 42.13 | |
| SCGF-B | rs149009264 | | 10 | A | G | 0.4551 | 0.9848 | 0.0985 | 3.83E-06 | 21.35 | |
| SCGF-B | rs150733161 | | 13 | T | C | -0.5255 | 0.0142 | 0.112 | 2.71E-06 | 22.01 | |
| SCGF-B | rs151194174 | | 7 | A | G | 0.4536 | 0.0187 | 0.0941 | 1.43E-06 | 23.24 | |
| SCGF-B | rs264157 | | 18 | A | G | 0.1079 | 0.4743 | 0.0233 | 3.64E-06 | 21.45 | |
| SCGF-B | rs34911860 | | 1 | A | G | -0.3674 | 0.0291 | 0.0787 | 3.04E-06 | 21.79 | |
| SCGF-B | rs3817303 | | 12 | T | G | 0.1362 | 0.194 | 0.0294 | 3.61E-06 | 21.46 | |
| SCGF-B | rs4737731 | | 8 | T | C | 0.1146 | 0.3019 | 0.0251 | 4.98E-06 | 20.85 | |
| SCGF-B | rs4976691 | | 5 | C | G | -0.1484 | 0.3257 | 0.0253 | 4.47E-09 | 34.41 | |
| SCGF-B | rs5742627 | | 12 | T | C | 0.2625 | 0.045 | 0.0574 | 4.80E-06 | 20.91 | |
| SCGF-B | rs77247938 | | 12 | A | G | 0.2862 | 0.0655 | 0.0475 | 1.69E-09 | 36.30 | |
| SCGF-B | rs77954165 | | 9 | T | C | 0.2631 | 0.9528 | 0.0562 | 2.85E-06 | 21.92 | |
| SCGF-B | rs7802293 | | 7 | T | C | -0.1145 | 0.3779 | 0.0247 | 3.56E-06 | 21.49 | |
| SCGF-B | rs78217154 | | 8 | T | C | 0.3942 | 0.9796 | 0.0861 | 4.69E-06 | 20.96 | |
| SDF-1A | rs10474392 | | 5 | A | G | 0.0934 | 0.334 | 0.0177 | 1.31E-07 | 27.84 | |
| SDF-1A | rs10516368 | | 4 | A | C | -0.4268 | 0.0105 | 0.0883 | 1.34E-06 | 23.36 | |
| SDF-1A | rs12141941 | | 1 | T | C | -0.0881 | 0.7493 | 0.0186 | 2.17E-06 | 22.43 | |
| SDF-1A | rs149893336 | | 4 | A | G | -0.494 | 0.993 | 0.1082 | 4.98E-06 | 20.84 | |
| SDF-1A | rs1600396 | | 4 | A | G | -0.0933 | 0.8096 | 0.0204 | 4.80E-06 | 20.92 | |
| SDF-1A | rs62194946 | | 2 | T | G | -0.0849 | 0.2628 | 0.0185 | 4.45E-06 | 21.06 | |
| SDF-1A | rs6586903 | | 8 | T | C | -0.1264 | 0.1037 | 0.0268 | 2.40E-06 | 22.24 | |
| SDF-1A | rs76766406 | | 4 | A | G | 0.4642 | 0.9935 | 0.1012 | 4.50E-06 | 21.04 | |
| SDF-1A | rs78037609 | | 21 | A | G | -0.6261 | 0.0104 | 0.1334 | 2.69E-06 | 22.03 | |
| SDF-1A | rs78883416 | | 10 | C | G | -0.0871 | 0.308 | 0.0182 | 1.70E-06 | 22.90 | |
| TNF-A | rs10767536 | | 11 | A | G | 0.118 | 0.3274 | 0.0253 | 3.10E-06 | 21.75 | |
| TNF-A | rs115018697 | | 4 | C | G | -0.9542 | 0.9962 | 0.197 | 1.27E-06 | 23.46 | |
| TNF-A | rs116736594 | | 5 | T | C | 0.3407 | 0.0312 | 0.0702 | 1.21E-06 | 23.55 | |
| TNF-A | rs79105320 | | 8 | A | G | 0.5573 | 0.0112 | 0.1177 | 2.19E-06 | 22.42 | |
| TNF-B | rs10925040 | | 1 | T | C | 0.1738 | 0.371 | 0.0372 | 2.98E-06 | 21.83 | |
| TNF-B | rs143259067 | | 1 | T | C | -0.6923 | 0.9595 | 0.1003 | 5.12E-12 | 47.64 | |
| TNF-B | rs2420873 | | 19 | T | G | 0.1673 | 0.3897 | 0.0365 | 4.57E-06 | 21.01 | |
| TNF-B | rs62284710 | | 3 | A | G | 0.3702 | 0.9443 | 0.0782 | 2.20E-06 | 22.41 | |
| TNF-B | rs75240021 | | 8 | C | G | 0.3713 | 0.071 | 0.0772 | 1.51E-06 | 23.13 | |
| TNF-B | rs76225863 | | 1 | A | G | 0.7534 | 0.0293 | 0.1217 | 5.99E-10 | 38.32 | |
| TRAIL | rs10084050 | | 18 | A | G | -0.1101 | 0.8647 | 0.023 | 1.69E-06 | 22.91 | |
| TRAIL | rs10164260 | | 18 | A | G | 0.1003 | 0.1741 | 0.0211 | 2.00E-06 | 22.60 | |
| TRAIL | rs11081739 | | 18 | A | G | 0.1395 | 0.2048 | 0.0202 | 4.99E-12 | 47.69 | |
| TRAIL | rs112821861 | | 18 | T | G | -0.8566 | 0.9717 | 0.0494 | 2.34E-67 | 300.68 | |
| TRAIL | rs113057689 | | 3 | A | G | -0.2625 | 0.033 | 0.0489 | 7.96E-08 | 28.82 | |
| TRAIL | rs11875481 | | 18 | T | C | -0.0969 | 0.835 | 0.0211 | 4.38E-06 | 21.09 | |
| TRAIL | rs13115587 | | 4 | A | C | 0.101 | 0.1558 | 0.0217 | 3.25E-06 | 21.66 | |
| TRAIL | rs13278062 | | 8 | T | G | 0.08 | 0.5589 | 0.0157 | 3.48E-07 | 25.96 | |
| TRAIL | rs139958028 | | 11 | A | G | 0.1803 | 0.0501 | 0.0395 | 5.01E-06 | 20.84 | |
| TRAIL | rs141032096 | | 18 | T | G | -0.2125 | 0.962 | 0.0455 | 3.01E-06 | 21.81 | |
| TRAIL | rs146827832 | | 3 | T | C | 0.1341 | 0.9038 | 0.0291 | 4.06E-06 | 21.24 | |
| TRAIL | rs148051545 | | 19 | T | C | -0.4211 | 0.0114 | 0.0843 | 5.88E-07 | 24.95 | |
| TRAIL | rs17535790 | | 3 | A | G | -0.1125 | 0.1498 | 0.0218 | 2.46E-07 | 26.63 | |
| TRAIL | rs183815186 | | 18 | A | T | -0.3499 | 0.9803 | 0.0602 | 6.16E-09 | 33.78 | |
| TRAIL | rs558572 | | 3 | T | C | 0.1351 | 0.0949 | 0.0265 | 3.43E-07 | 25.99 | |
| TRAIL | rs57396456 | | 18 | T | C | -0.5641 | 0.9755 | 0.0516 | 8.09E-28 | 119.51 | |
| TRAIL | rs62093482 | | 18 | T | C | 0.9827 | 0.0237 | 0.0529 | 4.97E-77 | 345.09 | |
| TRAIL | rs7233927 | | 18 | A | G | 0.0905 | 0.6648 | 0.0164 | 3.42E-08 | 30.45 | |
| TRAIL | rs73039026 | | 3 | A | C | -0.3098 | 0.9821 | 0.0634 | 1.03E-06 | 23.88 | |
| TRAIL | rs73408359 | | 18 | T | C | 0.4153 | 0.049 | 0.0364 | 3.76E-30 | 130.17 | |
| TRAIL | rs74488044 | | 18 | A | G | 0.3473 | 0.0583 | 0.0334 | 2.53E-25 | 108.12 | |
| TRAIL | rs747324 | | 14 | T | C | -0.0826 | 0.2828 | 0.0178 | 3.48E-06 | 21.53 | |
| TRAIL | rs74778900 | | 18 | T | C | 0.5791 | 0.0238 | 0.0531 | 1.08E-27 | 118.94 | |
| TRAIL | rs75473890 | | 18 | T | C | -0.1349 | 0.9136 | 0.028 | 1.45E-06 | 23.21 | |
| TRAIL | rs75489499 | | 3 | T | C | -0.2006 | 0.0677 | 0.0347 | 7.43E-09 | 33.42 | |
| TRAIL | rs75928541 | | 4 | A | G | 0.2784 | 0.0188 | 0.0591 | 2.47E-06 | 22.19 | |
| TRAIL | rs7599203 | | 2 | T | C | 0.0918 | 0.8034 | 0.02 | 4.43E-06 | 21.07 | |
| TRAIL | rs79085506 | | 18 | A | G | 0.6961 | 0.0154 | 0.0737 | 3.55E-21 | 89.21 | |
| TRAIL | rs9946486 | | 18 | A | G | 0.1856 | 0.1159 | 0.0245 | 3.58E-14 | 57.39 | |
| VEGF | rs10411345 | | 19 | C | G | -0.1041 | 0.7823 | 0.0218 | 1.80E-06 | 22.80 | |
| VEGF | rs10757514 | | 9 | C | G | -0.1024 | 0.1715 | 0.0222 | 3.98E-06 | 21.28 | |
| VEGF | rs10822118 | | 10 | T | C | -0.0797 | 0.4984 | 0.0168 | 2.09E-06 | 22.51 | |
| VEGF | rs10934631 | | 3 | T | C | -0.1132 | 0.8517 | 0.0244 | 3.50E-06 | 21.52 | |
| VEGF | rs10967183 | | 9 | T | C | -0.0887 | 0.4262 | 0.0169 | 1.53E-07 | 27.55 | |
| VEGF | rs111950052 | | 6 | A | G | -0.1763 | 0.0577 | 0.0383 | 4.16E-06 | 21.19 | |
| VEGF | rs114773511 | | 6 | T | C | 0.2187 | 0.0463 | 0.0441 | 7.08E-07 | 24.59 | |
| VEGF | rs12156533 | | 9 | A | T | 0.0915 | 0.6655 | 0.0186 | 8.68E-07 | 24.20 | |
| VEGF | rs12456390 | | 18 | T | C | -0.0818 | 0.6772 | 0.0179 | 4.88E-06 | 20.88 | |
| VEGF | rs13190738 | | 6 | T | C | 0.1111 | 0.3644 | 0.0231 | 1.51E-06 | 23.13 | |
| VEGF | rs1730969 | | 16 | C | G | -0.7811 | 0.0039 | 0.1696 | 4.11E-06 | 21.21 | |
| VEGF | rs3025020 | | 6 | T | C | -0.124 | 0.2786 | 0.0253 | 9.53E-07 | 24.02 | |
| VEGF | rs4573079 | | 6 | A | C | 0.1522 | 0.8439 | 0.0256 | 2.76E-09 | 35.35 | |
| VEGF | rs56071907 | | 16 | T | C | 0.126 | 0.1319 | 0.027 | 3.06E-06 | 21.78 | |
| VEGF | rs58078557 | | 9 | A | T | -0.1168 | 0.1473 | 0.024 | 1.13E-06 | 23.68 | |
| VEGF | rs60013354 | | 10 | A | G | -0.2497 | 0.0266 | 0.0521 | 1.65E-06 | 22.97 | |
| VEGF | rs60987108 | | 6 | A | G | 0.1812 | 0.9459 | 0.039 | 3.38E-06 | 21.59 | |
| VEGF | rs6496613 | | 15 | A | C | -0.2359 | 0.9677 | 0.0515 | 4.64E-06 | 20.98 | |
| VEGF | rs73872715 | | 3 | T | C | -0.6079 | 0.0045 | 0.1299 | 2.87E-06 | 21.90 | |
| VEGF | rs748227 | | 6 | T | C | 0.2377 | 0.0654 | 0.0364 | 6.57E-11 | | 42.64 |
| VEGF | rs7739450 | | 6 | A | G | -0.415 | 0.5059 | 0.018 | 1.29E-117 | | 531.56 |
| VEGF | rs7754905 | | 6 | A | G | -0.1303 | 0.7682 | 0.0204 | 1.69E-10 | 40.80 | |
| VEGF | rs7757024 | | 6 | T | C | 0.1443 | 0.0973 | 0.0291 | 7.09E-07 | 24.59 | |
| VEGF | rs77961527 | | 3 | A | G | 0.2289 | 0.9606 | 0.0457 | 5.48E-07 | 25.09 | |
| VEGF | rs9369440 | | 6 | T | C | -0.0872 | 0.2934 | 0.0186 | 2.76E-06 | 21.98 | |
| VEGF | rs9381249 | | 6 | T | C | -0.2414 | 0.9461 | 0.0396 | 1.09E-09 | 37.16 | |
| VEGF | rs9472153 | | 6 | A | G | 0.106 | 0.5187 | 0.0174 | 1.12E-09 | 37.11 | |

2.Instrumental variables for inflammatory factors and KOA

| **Exposure** | **SNP** | **Chr** | **EA** | **OA** | **Beta** | **EAF** | **SE** | **P** | **F** |
| --- | --- | --- | --- | --- | --- | --- | --- | --- | --- |
| B-NGF | rs28637706 | 19 | T | G | -0.1554 | 0.314 | 0.0261 | 2.62E-09 | 35.45 |
| B-NGF | rs4767014 | 12 | T | C | -0.1211 | 0.6731 | 0.0264 | 4.49E-06 | 21.04 |
| B-NGF | rs71641308 | 1 | T | C | 0.1969 | 0.0995 | 0.0429 | 4.44E-06 | 21.07 |
| B-NGF | rs73472576 | 18 | T | C | -0.1146 | 0.457 | 0.0251 | 4.98E-06 | 20.85 |
| CTACK | rs10854859 | 22 | A | G | -0.1498 | 0.2464 | 0.0293 | 3.18E-07 | 26.14 |
| CTACK | rs116303454 | 3 | A | G | 0.3754 | 0.0218 | 0.081 | 3.58E-06 | 21.48 |
| CTACK | rs116871507 | 9 | A | T | -0.2086 | 0.0791 | 0.0448 | 3.22E-06 | 21.68 |
| CTACK | rs116943377 | 13 | A | G | 0.2878 | 0.0394 | 0.0611 | 2.47E-06 | 22.19 |
| CTACK | rs117932939 | 9 | T | C | 0.1969 | 0.09 | 0.0422 | 3.07E-06 | 21.77 |
| CTACK | rs118084576 | 9 | A | G | 0.5675 | 0.0112 | 0.1226 | 3.68E-06 | 21.43 |
| CTACK | rs144072067 | 9 | A | G | 0.4451 | 0.9574 | 0.0579 | 1.50E-14 | 59.10 |
| CTACK | rs184329319 | 9 | T | G | -0.3069 | 0.035 | 0.0648 | 2.18E-06 | 22.43 |
| CTACK | rs2233872 | 9 | A | G | -0.1495 | 0.7699 | 0.0282 | 1.15E-07 | 28.11 |
| CTACK | rs55764737 | 15 | T | C | 0.5424 | 0.9846 | 0.0967 | 2.03E-08 | 31.46 |
| CTACK | rs57338032 | 15 | A | G | 0.1443 | 0.8287 | 0.0316 | 4.96E-06 | 20.85 |
| CTACK | rs57789542 | 3 | T | C | -0.7687 | 0.9951 | 0.1659 | 3.60E-06 | 21.47 |
| CTACK | rs60247384 | 3 | T | C | 0.1128 | 0.3512 | 0.0245 | 4.14E-06 | 21.20 |
| CTACK | rs62578137 | 9 | T | C | -0.1311 | 0.2421 | 0.0286 | 4.56E-06 | 21.01 |
| CTACK | rs72729450 | 9 | T | C | -0.5123 | 0.0123 | 0.1094 | 2.83E-06 | 21.93 |
| CTACK | rs76395525 | 15 | A | G | 0.5193 | 0.0118 | 0.1081 | 1.56E-06 | 23.08 |
| EOTAXIN | rs11920996 | 3 | T | C | 0.2979 | 0.0512 | 0.0377 | 2.75E-15 | 62.44 |
| EOTAXIN | rs147287945 | 6 | A | G | -0.1512 | 0.0741 | 0.0313 | 1.36E-06 | 23.34 |
| EOTAXIN | rs1677588 | 1 | T | G | 0.1181 | 0.886 | 0.025 | 2.31E-06 | 22.32 |
| EOTAXIN | rs2024050 | 7 | A | G | 0.164 | 0.0739 | 0.0302 | 5.62E-08 | 29.49 |
| EOTAXIN | rs2040143 | 21 | A | G | -0.0858 | 0.7234 | 0.0178 | 1.43E-06 | 23.23 |
| EOTAXIN | rs2097947 | 7 | A | T | -0.0769 | 0.6836 | 0.0168 | 4.71E-06 | 20.95 |
| EOTAXIN | rs2229593 | 3 | T | C | 0.3647 | 0.0387 | 0.0406 | 2.64E-19 | 80.69 |
| EOTAXIN | rs2249581 | 1 | T | C | -0.0899 | 0.748 | 0.018 | 5.90E-07 | 24.94 |
| EOTAXIN | rs2828756 | 21 | T | C | 0.0904 | 0.2423 | 0.0184 | 8.97E-07 | 24.14 |
| EOTAXIN | rs34004101 | 3 | T | C | -0.1775 | 0.065 | 0.0341 | 1.94E-07 | 27.09 |
| EOTAXIN | rs4683182 | 3 | A | G | 0.0886 | 0.2201 | 0.0189 | 2.76E-06 | 21.98 |
| EOTAXIN | rs5754733 | 22 | A | C | -0.105 | 0.8306 | 0.0213 | 8.24E-07 | 24.30 |
| EOTAXIN | rs57723662 | 17 | C | G | -0.0982 | 0.1657 | 0.0213 | 4.02E-06 | 21.26 |
| EOTAXIN | rs60075014 | 5 | T | C | -0.1688 | 0.0604 | 0.0356 | 2.12E-06 | 22.48 |
| EOTAXIN | rs7231030 | 18 | A | C | 0.0903 | 0.7919 | 0.0193 | 2.89E-06 | 21.89 |
| EOTAXIN | rs73065695 | 3 | A | G | 0.1532 | 0.0932 | 0.0281 | 4.98E-08 | 29.72 |
| EOTAXIN | rs745331 | 15 | A | G | -0.0821 | 0.6825 | 0.0176 | 3.09E-06 | 21.76 |
| EOTAXIN | rs75426604 | 14 | A | C | -0.1371 | 0.0876 | 0.0291 | 2.46E-06 | 22.20 |
| EOTAXIN | rs7550207 | 1 | T | C | -0.0904 | 0.7785 | 0.0187 | 1.34E-06 | 23.37 |
| EOTAXIN | rs9317045 | 13 | A | C | 0.1172 | 0.8615 | 0.0236 | 6.83E-07 | 24.66 |
| EOTAXIN | rs9833459 | 3 | T | C | -0.1149 | 0.3033 | 0.0173 | 3.10E-11 | 44.11 |
| EOTAXIN | rs9975149 | 21 | A | T | 0.0749 | 0.3743 | 0.0164 | 4.95E-06 | 20.86 |
| FGF-BASIC | rs13412535 | 2 | A | G | -0.1129 | 0.1856 | 0.0224 | 4.65E-07 | 25.40 |
| FGF-BASIC | rs147409637 | 6 | T | C | 0.201 | 0.0369 | 0.0431 | 3.11E-06 | 21.75 |
| FGF-BASIC | rs17094040 | 14 | T | C | 0.1051 | 0.1522 | 0.0229 | 4.44E-06 | 21.06 |
| FGF-BASIC | rs2849358 | 18 | A | G | 0.0911 | 0.2551 | 0.0193 | 2.36E-06 | 22.28 |
| FGF-BASIC | rs4795091 | 17 | A | G | 0.1239 | 0.8926 | 0.0266 | 3.19E-06 | 21.70 |
| FGF-BASIC | rs76253061 | 5 | T | C | -0.4811 | 0.9925 | 0.1041 | 3.81E-06 | 21.36 |
| FGF-BASIC | rs78873483 | 17 | A | G | 0.1286 | 0.1025 | 0.0282 | 5.11E-06 | 20.80 |
| G-CSF | rs10939033 | 4 | A | G | -0.0775 | 0.6038 | 0.0163 | 1.99E-06 | 22.61 |
| G-CSF | rs117261691 | 19 | T | C | 0.1318 | 0.0894 | 0.0288 | 4.73E-06 | 20.94 |
| G-CSF | rs183023730 | 9 | T | G | 0.7898 | 0.0034 | 0.1677 | 2.48E-06 | 22.18 |
| G-CSF | rs586802 | 11 | A | G | 0.0882 | 0.758 | 0.0187 | 2.40E-06 | 22.25 |
| G-CSF | rs6740648 | 2 | T | C | 0.0818 | 0.6752 | 0.0172 | 1.98E-06 | 22.62 |
| G-CSF | rs74148555 | 10 | T | C | -0.3771 | 0.0144 | 0.0753 | 5.50E-07 | 25.08 |
| G-CSF | rs76287671 | 19 | T | C | 0.0894 | 0.2333 | 0.0189 | 2.24E-06 | 22.37 |
| G-CSF | rs77318030 | 19 | T | C | -0.2031 | 0.9563 | 0.0427 | 1.97E-06 | 22.62 |
| G-CSF | rs78523761 | 5 | A | G | 0.5374 | 0.0077 | 0.1139 | 2.38E-06 | 22.26 |
| GROA | rs10015342 | 4 | A | T | 0.1945 | 0.1909 | 0.0304 | 1.57E-10 | 40.93 |
| GROA | rs114991247 | 6 | T | C | -0.2202 | 0.9226 | 0.0463 | 1.98E-06 | 22.62 |
| GROA | rs115214168 | 4 | T | C | 0.4528 | 0.0242 | 0.0828 | 4.54E-08 | 29.91 |
| GROA | rs1361829 | 1 | A | G | -0.1106 | 0.4293 | 0.0241 | 4.45E-06 | 21.06 |
| GROA | rs140734053 | 10 | A | G | 0.7333 | 0.0066 | 0.1545 | 2.07E-06 | 22.53 |
| GROA | rs150194856 | 1 | T | C | -0.4223 | 0.0177 | 0.0914 | 3.83E-06 | 21.35 |
| GROA | rs17171245 | 7 | T | G | 0.2446 | 0.0536 | 0.053 | 3.93E-06 | 21.30 |
| GROA | rs185768063 | 6 | A | G | 0.4038 | 0.9735 | 0.076 | 1.08E-07 | 28.23 |
| GROA | rs188345231 | 8 | T | C | 0.6177 | 0.0102 | 0.1322 | 2.98E-06 | 21.83 |
| GROA | rs3026943 | 1 | A | C | -0.1246 | 0.3311 | 0.0256 | 1.13E-06 | 23.69 |
| GROA | rs3845622 | 1 | A | C | -0.2345 | 0.1171 | 0.0381 | 7.51E-10 | 37.88 |
| GROA | rs62024303 | 15 | A | G | -0.3013 | 0.9617 | 0.066 | 4.99E-06 | 20.84 |
| GROA | rs73020704 | 1 | A | G | 0.2607 | 0.9484 | 0.0545 | 1.72E-06 | 22.88 |
| GROA | rs76215157 | 20 | C | G | -0.7398 | 0.0074 | 0.1564 | 2.24E-06 | 22.37 |
| GROA | rs76390238 | 2 | C | G | 0.6223 | 0.0095 | 0.1352 | 4.17E-06 | 21.19 |
| HGF | rs11060254 | 12 | A | G | -0.0765 | 0.3306 | 0.0166 | 4.06E-06 | 21.24 |
| HGF | rs11129909 | 3 | T | C | -0.0738 | 0.623 | 0.0161 | 4.56E-06 | 21.01 |
| HGF | rs13412535 | 2 | A | G | -0.1043 | 0.1892 | 0.0213 | 9.75E-07 | 23.98 |
| HGF | rs2003620 | 7 | T | C | 0.2277 | 0.027 | 0.0487 | 2.93E-06 | 21.86 |
| HGF | rs2699434 | 4 | T | C | 0.0863 | 0.3287 | 0.0171 | 4.49E-07 | 25.47 |
| HGF | rs362307 | 4 | T | C | 0.1511 | 0.0646 | 0.0328 | 4.09E-06 | 21.22 |
| HGF | rs4245058 | 11 | T | C | -0.1552 | 0.0619 | 0.0331 | 2.75E-06 | 21.99 |
| HGF | rs57146176 | 12 | A | G | -0.0987 | 0.7998 | 0.0208 | 2.08E-06 | 22.52 |
| HGF | rs5745687 | 7 | T | C | -0.3008 | 0.0375 | 0.0404 | 9.65E-14 | 55.44 |
| HGF | rs80051150 | 21 | T | C | 0.198 | 0.9595 | 0.0413 | 1.63E-06 | 22.98 |
| IFN-G | rs113399544 | 7 | A | G | -0.0849 | 0.2768 | 0.0183 | 3.50E-06 | 21.52 |
| IFN-G | rs113600793 | 17 | A | C | 0.1871 | 0.0631 | 0.0371 | 4.58E-07 | 25.43 |
| IFN-G | rs115729819 | 4 | A | G | 0.2511 | 0.9716 | 0.0514 | 1.03E-06 | 23.87 |
| IFN-G | rs117046255 | 7 | T | C | -0.0968 | 0.1922 | 0.0207 | 2.92E-06 | 21.87 |
| IFN-G | rs11843756 | 13 | T | G | 0.1812 | 0.9542 | 0.0391 | 3.58E-06 | 21.48 |
| IFN-G | rs12420286 | 11 | T | C | 0.2357 | 0.9699 | 0.05 | 2.43E-06 | 22.22 |
| IFN-G | rs147378920 | 1 | A | G | -0.384 | 0.9871 | 0.0751 | 3.17E-07 | 26.14 |
| IFN-G | rs1867282 | 9 | T | C | 0.0781 | 0.4154 | 0.0166 | 2.54E-06 | 22.14 |
| IFN-G | rs2073438 | 17 | A | G | 0.092 | 0.2506 | 0.0188 | 9.90E-07 | 23.95 |
| IFN-G | rs7088799 | 10 | T | G | -0.0805 | 0.6182 | 0.0166 | 1.24E-06 | 23.52 |
| IFN-G | rs73479333 | 6 | C | G | -0.1123 | 0.1354 | 0.024 | 2.88E-06 | 21.89 |
| IFN-G | rs74148555 | 10 | T | C | -0.3771 | 0.0144 | 0.077 | 9.71E-07 | 23.98 |
| IL-10 | rs10457128 | 6 | A | G | -0.0854 | 0.639 | 0.0172 | 6.87E-07 | 24.65 |
| IL-10 | rs10493718 | 1 | A | C | -0.1081 | 0.1603 | 0.0222 | 1.12E-06 | 23.71 |
| IL-10 | rs13412535 | 2 | A | G | -0.1347 | 0.1856 | 0.0224 | 1.82E-09 | 36.16 |
| IL-10 | rs143072171 | 6 | T | C | 0.2089 | 0.0413 | 0.0429 | 1.12E-06 | 23.71 |
| IL-10 | rs1530455 | 3 | T | C | 0.082 | 0.3523 | 0.0174 | 2.45E-06 | 22.21 |
| IL-10 | rs181031888 | 6 | A | T | 0.2577 | 0.961 | 0.0472 | 4.77E-08 | 29.81 |
| IL-10 | rs2086656 | 4 | T | C | -0.08 | 0.6621 | 0.017 | 2.53E-06 | 22.15 |
| IL-10 | rs3002131 | 1 | C | G | 0.1191 | 0.1195 | 0.026 | 4.63E-06 | 20.98 |
| IL-10 | rs3025021 | 6 | T | C | 0.0913 | 0.3459 | 0.0194 | 2.52E-06 | 22.15 |
| IL-10 | rs339203 | 2 | T | C | 0.0954 | 0.8359 | 0.0203 | 2.61E-06 | 22.09 |
| IL-10 | rs383684 | 6 | A | G | 0.092 | 0.7555 | 0.0197 | 3.01E-06 | 21.81 |
| IL-10 | rs41282660 | 6 | A | G | -0.1169 | 0.8646 | 0.0254 | 4.18E-06 | 21.18 |
| IL-10 | rs465757 | 20 | A | G | 0.0806 | 0.6657 | 0.0174 | 3.62E-06 | 21.46 |
| IL-10 | rs4741748 | 9 | A | G | -0.0788 | 0.6081 | 0.0169 | 3.12E-06 | 21.74 |
| IL-10 | rs6054847 | 20 | T | C | 0.0971 | 0.8091 | 0.0207 | 2.72E-06 | 22.00 |
| IL-10 | rs6680918 | 1 | T | C | -0.1202 | 0.8791 | 0.025 | 1.52E-06 | 23.12 |
| IL-10 | rs7088799 | 10 | T | G | -0.0815 | 0.6191 | 0.0166 | 9.12E-07 | 24.10 |
| IL-10 | rs73192842 | 3 | A | G | 0.0949 | 0.1911 | 0.0206 | 4.09E-06 | 21.22 |
| IL-10 | rs7747448 | 6 | A | G | -0.1061 | 0.2594 | 0.0189 | 1.98E-08 | 31.51 |
| IL-10 | rs865585 | 6 | A | C | -0.119 | 0.8478 | 0.0244 | 1.08E-06 | 23.79 |
| IL-10 | rs910604 | 6 | A | G | -0.0986 | 0.2383 | 0.0196 | 4.89E-07 | 25.31 |
| IL-12-P70 | rs113600793 | 17 | A | C | 0.1832 | 0.0623 | 0.0359 | 3.34E-07 | 26.04 |
| IL-12-P70 | rs12154194 | 6 | T | C | 0.0717 | 0.4615 | 0.0157 | 4.95E-06 | 20.86 |
| IL-12-P70 | rs12969892 | 18 | T | C | 0.1227 | 0.9031 | 0.0267 | 4.32E-06 | 21.12 |
| IL-12-P70 | rs13190738 | 6 | T | C | 0.1019 | 0.3609 | 0.019 | 8.18E-08 | 28.76 |
| IL-12-P70 | rs148449807 | 6 | A | G | -0.4217 | 0.0164 | 0.0793 | 1.05E-07 | 28.28 |
| IL-12-P70 | rs181031888 | 6 | A | T | 0.2861 | 0.9606 | 0.0456 | 3.52E-10 | 39.36 |
| IL-12-P70 | rs2123852 | 19 | T | C | 0.0942 | 0.2039 | 0.0204 | 3.88E-06 | 21.32 |
| IL-12-P70 | rs2495005 | 10 | A | G | -0.075 | 0.4416 | 0.0159 | 2.39E-06 | 22.25 |
| IL-12-P70 | rs273702 | 18 | A | G | -0.127 | 0.9015 | 0.027 | 2.55E-06 | 22.12 |
| IL-12-P70 | rs282258 | 2 | T | C | 0.0726 | 0.4279 | 0.0156 | 3.26E-06 | 21.66 |
| IL-12-P70 | rs34322762 | 9 | T | C | 0.0953 | 0.648 | 0.0199 | 1.68E-06 | 22.93 |
| IL-12-P70 | rs34826779 | 8 | T | G | -0.0884 | 0.2157 | 0.019 | 3.28E-06 | 21.65 |
| IL-12-P70 | rs41282644 | 6 | A | G | 0.1401 | 0.0849 | 0.0303 | 3.77E-06 | 21.38 |
| IL-12-P70 | rs4714698 | 6 | A | G | -0.1189 | 0.8721 | 0.0238 | 5.86E-07 | 24.96 |
| IL-12-P70 | rs4741748 | 9 | A | G | -0.0799 | 0.6076 | 0.0163 | 9.49E-07 | 24.03 |
| IL-12-P70 | rs6532374 | 4 | T | C | -0.1033 | 0.8525 | 0.0226 | 4.86E-06 | 20.89 |
| IL-12-P70 | rs7763358 | 6 | T | C | 0.1525 | 0.0885 | 0.0274 | 2.61E-08 | 30.98 |
| IL-12-P70 | rs7765264 | 6 | A | G | 0.1428 | 0.0767 | 0.0301 | 2.09E-06 | 22.51 |
| IL-12-P70 | rs782111 | 12 | A | C | -0.0765 | 0.5292 | 0.0156 | 9.40E-07 | 24.05 |
| IL-12-P70 | rs865585 | 6 | A | C | -0.1654 | 0.8484 | 0.0237 | 2.97E-12 | 48.71 |
| IL-12-P70 | rs9381249 | 6 | T | C | -0.1788 | 0.9466 | 0.0367 | 1.11E-06 | 23.74 |
| IL-12-P70 | rs9472153 | 6 | A | G | 0.0855 | 0.5141 | 0.0159 | 7.56E-08 | 28.92 |
| IL-12-P70 | rs9472175 | 6 | T | C | -0.1104 | 0.1542 | 0.0238 | 3.51E-06 | 21.52 |
| IL-13 | rs10995604 | 10 | A | G | -0.1571 | 0.8587 | 0.0343 | 4.65E-06 | 20.98 |
| IL-13 | rs117795020 | 9 | A | G | -0.3584 | 0.0304 | 0.0716 | 5.57E-07 | 25.06 |
| IL-13 | rs12199215 | 6 | T | C | 0.1309 | 0.247 | 0.0284 | 4.04E-06 | 21.24 |
| IL-13 | rs12623722 | 2 | A | G | -0.1189 | 0.3054 | 0.0257 | 3.72E-06 | 21.40 |
| IL-13 | rs13206012 | 6 | A | G | -0.3719 | 0.3489 | 0.0263 | 2.13E-45 | 199.96 |
| IL-13 | rs13209117 | 6 | A | G | 0.1409 | 0.245 | 0.0284 | 7.00E-07 | 24.61 |
| IL-13 | rs138854806 | 6 | A | G | -0.4204 | 0.0247 | 0.0839 | 5.42E-07 | 25.11 |
| IL-13 | rs139083458 | 5 | T | C | 0.9995 | 0.0034 | 0.211 | 2.17E-06 | 22.44 |
| IL-13 | rs145023524 | 6 | A | G | 0.2815 | 0.0435 | 0.0588 | 1.69E-06 | 22.92 |
| IL-13 | rs147747784 | 19 | C | G | 0.369 | 0.0285 | 0.0765 | 1.41E-06 | 23.27 |
| IL-13 | rs150836197 | 6 | T | C | 0.3283 | 0.0367 | 0.0713 | 4.13E-06 | 21.20 |
| IL-13 | rs27949 | 5 | T | C | -0.1144 | 0.667 | 0.025 | 4.74E-06 | 20.94 |
| IL-13 | rs28442067 | 3 | A | G | -0.1379 | 0.7832 | 0.0286 | 1.42E-06 | 23.25 |
| IL-13 | rs7073807 | 10 | T | C | 0.1618 | 0.1351 | 0.0354 | 4.86E-06 | 20.89 |
| IL-13 | rs75383097 | 1 | C | G | -0.5369 | 0.0132 | 0.116 | 3.68E-06 | 21.42 |
| IL-13 | rs76339001 | 21 | A | T | -0.4375 | 0.9767 | 0.0886 | 7.90E-07 | 24.38 |
| IL-13 | rs76975337 | 3 | T | C | -0.1211 | 0.7286 | 0.0265 | 4.88E-06 | 20.88 |
| IL-13 | rs7747448 | 6 | A | G | -0.1393 | 0.2585 | 0.0278 | 5.42E-07 | 25.11 |
| IL-13 | rs7757246 | 6 | T | C | 0.2147 | 0.9092 | 0.0422 | 3.62E-07 | 25.88 |
| IL-13 | rs77955971 | 6 | A | C | 0.4408 | 0.0247 | 0.0868 | 3.81E-07 | 25.79 |
| IL-13 | rs9296421 | 6 | T | G | 0.1814 | 0.842 | 0.0348 | 1.86E-07 | 27.17 |
| IL-16 | rs117217798 | 17 | T | C | -0.2064 | 0.0889 | 0.044 | 2.72E-06 | 22.00 |
| IL-16 | rs12577604 | 11 | T | C | 0.4335 | 0.9818 | 0.0941 | 4.09E-06 | 21.22 |
| IL-16 | rs142034902 | 12 | A | G | -0.4367 | 0.9827 | 0.0925 | 2.35E-06 | 22.29 |
| IL-16 | rs142332135 | 15 | A | G | -0.7646 | 0.0164 | 0.1082 | 1.59E-12 | 49.94 |
| IL-16 | rs144691581 | 15 | A | G | 0.4929 | 0.0197 | 0.0958 | 2.67E-07 | 26.47 |
| IL-16 | rs35834666 | 4 | T | C | -0.1729 | 0.8596 | 0.0348 | 6.75E-07 | 24.68 |
| IL-16 | rs4778640 | 15 | A | G | 0.7189 | 0.9848 | 0.0983 | 2.61E-13 | 53.48 |
| IL-16 | rs4976691 | 5 | C | G | 0.1254 | 0.3268 | 0.026 | 1.41E-06 | 23.26 |
| IL-16 | rs7097884 | 10 | T | C | -0.1193 | 0.5647 | 0.0243 | 9.13E-07 | 24.10 |
| IL-16 | rs78042619 | 9 | A | G | 0.55 | 0.9883 | 0.1158 | 2.04E-06 | 22.56 |
| IL-17 | rs11640734 | 16 | C | G | -0.115 | 0.8673 | 0.024 | 1.65E-06 | 22.96 |
| IL-17 | rs11985957 | 8 | A | G | 0.1511 | 0.0642 | 0.0329 | 4.38E-06 | 21.09 |
| IL-17 | rs12735700 | 1 | T | G | -0.0943 | 0.1925 | 0.0206 | 4.70E-06 | 20.96 |
| IL-17 | rs148562661 | 6 | C | G | 0.2161 | 0.9605 | 0.0434 | 6.38E-07 | 24.79 |
| IL-17 | rs17282552 | 2 | T | C | -0.2026 | 0.952 | 0.0403 | 4.97E-07 | 25.27 |
| IL-17 | rs3804749 | 3 | T | C | -0.0923 | 0.6236 | 0.0167 | 3.26E-08 | 30.55 |
| IL-17 | rs61990749 | 14 | C | G | 0.1124 | 0.1533 | 0.0226 | 6.58E-07 | 24.74 |
| IL-17 | rs77341831 | 12 | T | C | 0.2263 | 0.9696 | 0.0473 | 1.72E-06 | 22.89 |
| IL-17 | rs78296352 | 1 | T | G | 0.2949 | 0.0186 | 0.0645 | 4.83E-06 | 20.90 |
| IL-17 | rs9519328 | 13 | A | G | 0.5256 | 0.9845 | 0.1101 | 1.81E-06 | 22.79 |
| IL-17 | rs9568764 | 13 | C | G | 0.0825 | 0.2756 | 0.018 | 4.58E-06 | 21.01 |
| IL-18 | rs10409850 | 19 | A | G | 0.1791 | 0.8704 | 0.0347 | 2.45E-07 | 26.64 |
| IL-18 | rs113214367 | 11 | A | G | -0.278 | 0.0565 | 0.0593 | 2.76E-06 | 21.98 |
| IL-18 | rs117266781 | 7 | T | C | 0.7051 | 0.0073 | 0.1436 | 9.10E-07 | 24.11 |
| IL-18 | rs117371668 | 16 | T | G | 0.3712 | 0.0254 | 0.0799 | 3.39E-06 | 21.58 |
| IL-18 | rs12419156 | 11 | T | C | 0.1556 | 0.8064 | 0.0335 | 3.40E-06 | 21.57 |
| IL-18 | rs139468359 | 5 | T | C | 0.5101 | 0.9866 | 0.1088 | 2.75E-06 | 21.98 |
| IL-18 | rs139727649 | 5 | T | C | -0.356 | 0.9745 | 0.0751 | 2.13E-06 | 22.47 |
| IL-18 | rs150005227 | 5 | T | C | 0.4062 | 0.0185 | 0.0886 | 4.55E-06 | 21.02 |
| IL-18 | rs1979967 | 15 | T | C | 0.14 | 0.2157 | 0.0285 | 9.00E-07 | 24.13 |
| IL-18 | rs4952239 | 2 | A | T | -0.1156 | 0.3555 | 0.0242 | 1.78E-06 | 22.82 |
| IL-18 | rs58701153 | 6 | A | T | -0.1265 | 0.6586 | 0.0242 | 1.72E-07 | 27.32 |
| IL-18 | rs62312914 | 4 | T | C | -0.1265 | 0.3726 | 0.025 | 4.19E-07 | 25.60 |
| IL-18 | rs7599125 | 2 | A | G | 0.1109 | 0.569 | 0.0239 | 3.48E-06 | 21.53 |
| IL-18 | rs76138275 | 5 | T | C | 0.1227 | 0.2858 | 0.026 | 2.37E-06 | 22.27 |
| IL-18 | rs764078 | 11 | A | T | 0.1283 | 0.2239 | 0.0278 | 3.93E-06 | 21.30 |
| IL-18 | rs77187209 | 5 | T | C | -0.4859 | 0.9866 | 0.1041 | 3.05E-06 | 21.79 |
| IL-18 | rs78623212 | 7 | T | C | 0.8322 | 0.0053 | 0.1676 | 6.86E-07 | 24.66 |
| IL-18 | rs78716465 | 20 | A | G | 0.3173 | 0.033 | 0.0679 | 2.97E-06 | 21.84 |
| IL-1B | rs143319329 | 7 | T | C | 0.4357 | 0.0209 | 0.093 | 2.80E-06 | 21.95 |
| IL-1B | rs4786740 | 16 | A | C | 0.1264 | 0.3913 | 0.0265 | 1.84E-06 | 22.75 |
| IL-1B | rs61335305 | 15 | A | C | 0.4333 | 0.0189 | 0.0928 | 3.02E-06 | 21.80 |
| IL-1B | rs62015704 | 16 | A | G | 0.1786 | 0.8688 | 0.0372 | 1.58E-06 | 23.05 |
| IL-1RA | rs1054402 | 9 | T | C | 0.1325 | 0.2506 | 0.0269 | 8.41E-07 | 24.26 |
| IL-1RA | rs117181659 | 22 | A | G | -0.2204 | 0.064 | 0.0478 | 4.01E-06 | 21.26 |
| IL-1RA | rs11869294 | 17 | C | G | -0.2286 | 0.9205 | 0.047 | 1.15E-06 | 23.66 |
| IL-1RA | rs13343438 | 19 | A | G | 0.2771 | 0.0434 | 0.0607 | 4.99E-06 | 20.84 |
| IL-1RA | rs35590641 | 14 | C | G | -0.1167 | 0.3321 | 0.025 | 3.04E-06 | 21.79 |
| IL-1RA | rs3876037 | 22 | A | G | 0.1234 | 0.6783 | 0.027 | 4.87E-06 | 20.89 |
| IL-1RA | rs56134659 | 3 | A | G | -0.1109 | 0.4835 | 0.0236 | 2.61E-06 | 22.08 |
| IL-1RA | rs61335305 | 15 | A | C | 0.4315 | 0.0182 | 0.0904 | 1.81E-06 | 22.78 |
| IL-1RA | rs6699436 | 1 | A | G | -0.1858 | 0.0995 | 0.0404 | 4.25E-06 | 21.15 |
| IL-1RA | rs9623661 | 22 | T | C | -0.1948 | 0.0903 | 0.0424 | 4.34E-06 | 21.11 |
| IL-1RA | rs9985296 | 3 | T | C | 0.1053 | 0.4758 | 0.0231 | 5.15E-06 | 20.78 |
| IL-2 | rs13412535 | 2 | A | G | 0.174 | 0.1886 | 0.0331 | 1.47E-07 | 27.63 |
| IL-2 | rs16836080 | 3 | A | G | 0.1158 | 0.3355 | 0.0253 | 4.72E-06 | 20.95 |
| IL-2 | rs2690020 | 1 | A | G | 0.1158 | 0.5177 | 0.0245 | 2.28E-06 | 22.34 |
| IL-2 | rs4479767 | 4 | A | G | 0.1821 | 0.8977 | 0.0392 | 3.39E-06 | 21.58 |
| IL-2 | rs4634519 | 7 | A | G | -0.1249 | 0.7379 | 0.0268 | 3.16E-06 | 21.72 |
| IL-2 | rs61335305 | 15 | A | C | 0.4439 | 0.0184 | 0.0913 | 1.16E-06 | 23.64 |
| IL-2 | rs62124990 | 2 | T | G | -0.7013 | 0.0083 | 0.149 | 2.52E-06 | 22.15 |
| IL-2 | rs7615304 | 3 | A | G | -0.1139 | 0.4531 | 0.024 | 2.08E-06 | 22.52 |
| IL-2RA | rs11241559 | 5 | T | G | -0.124 | 0.2643 | 0.0264 | 2.64E-06 | 22.06 |
| IL-2RA | rs115360066 | 5 | A | G | 0.1776 | 0.8882 | 0.0377 | 2.47E-06 | 22.19 |
| IL-2RA | rs117244812 | 17 | A | G | -0.7187 | 0.0079 | 0.1493 | 1.48E-06 | 23.17 |
| IL-2RA | rs12789243 | 11 | T | C | 0.1263 | 0.7571 | 0.0276 | 4.74E-06 | 20.94 |
| IL-2RA | rs17624670 | 8 | A | G | -0.125 | 0.2531 | 0.0273 | 4.68E-06 | 20.96 |
| IL-2RA | rs34037190 | 10 | A | G | 0.4784 | 0.0217 | 0.0935 | 3.11E-07 | 26.18 |
| IL-2RA | rs34353319 | 10 | A | T | -0.1581 | 0.8176 | 0.0335 | 2.37E-06 | 22.27 |
| IL-2RA | rs56213152 | 7 | T | C | 0.1269 | 0.7688 | 0.0271 | 2.83E-06 | 21.93 |
| IL-2RA | rs7078614 | 10 | T | G | -0.1543 | 0.3549 | 0.0241 | 1.53E-10 | 40.99 |
| IL-2RA | rs79100208 | 3 | C | G | 0.8345 | 0.995 | 0.1758 | 2.07E-06 | 22.53 |
| IL-2RA | rs9423654 | 10 | C | G | -0.135 | 0.6609 | 0.0283 | 1.84E-06 | 22.76 |
| IL-4 | rs116705532 | 1 | T | G | -0.4675 | 0.9925 | 0.0978 | 1.75E-06 | 22.85 |
| IL-4 | rs117146485 | 9 | T | C | -0.2856 | 0.9837 | 0.0625 | 4.89E-06 | 20.88 |
| IL-4 | rs12238729 | 9 | T | C | 0.5271 | 0.0162 | 0.1096 | 1.51E-06 | 23.13 |
| IL-4 | rs12640583 | 4 | T | G | -0.1104 | 0.1631 | 0.0214 | 2.48E-07 | 26.61 |
| IL-4 | rs17713451 | 7 | A | G | 0.1255 | 0.1149 | 0.0252 | 6.35E-07 | 24.80 |
| IL-4 | rs1867282 | 9 | T | C | 0.0808 | 0.4148 | 0.0162 | 6.11E-07 | 24.88 |
| IL-4 | rs2073438 | 17 | A | G | 0.0847 | 0.2513 | 0.0183 | 3.68E-06 | 21.42 |
| IL-4 | rs2346020 | 3 | A | G | 0.079 | 0.6795 | 0.0169 | 2.95E-06 | 21.85 |
| IL-4 | rs2708586 | 7 | T | C | -0.0767 | 0.3411 | 0.0166 | 3.83E-06 | 21.35 |
| IL-4 | rs56408830 | 6 | A | G | -0.1794 | 0.0537 | 0.0365 | 8.88E-07 | 24.16 |
| IL-4 | rs7613691 | 3 | A | G | 0.1787 | 0.9482 | 0.0382 | 2.90E-06 | 21.88 |
| IL-4 | rs79597994 | 1 | T | C | -0.5855 | 0.0049 | 0.1271 | 4.09E-06 | 21.22 |
| IL-4 | rs9506111 | 13 | A | G | -0.1446 | 0.9326 | 0.0314 | 4.12E-06 | 21.21 |
| IL-4 | rs9941733 | 20 | A | G | 0.1156 | 0.8355 | 0.0229 | 4.46E-07 | 25.48 |
| IL-5 | rs10178043 | 2 | T | G | 0.2579 | 0.949 | 0.0553 | 3.11E-06 | 21.75 |
| IL-5 | rs111736126 | 2 | C | G | -0.3973 | 0.9792 | 0.0867 | 4.60E-06 | 21.00 |
| IL-5 | rs148634917 | 1 | A | G | -0.517 | 0.986 | 0.1087 | 1.97E-06 | 22.62 |
| IL-5 | rs28793375 | 8 | T | C | 0.1697 | 0.1298 | 0.0362 | 2.76E-06 | 21.98 |
| IL-5 | rs72831687 | 6 | A | G | -0.5337 | 0.0137 | 0.1104 | 1.34E-06 | 23.37 |
| IL-5 | rs73040118 | 19 | T | C | 0.2294 | 0.9285 | 0.049 | 2.85E-06 | 21.92 |
| IL-5 | rs74811276 | 14 | A | G | 0.217 | 0.0761 | 0.0471 | 4.08E-06 | 21.23 |
| IL-5 | rs7739450 | 6 | A | G | -0.1295 | 0.5158 | 0.0256 | 4.22E-07 | 25.59 |
| IL-5 | rs9309063 | 2 | T | G | -0.1119 | 0.4763 | 0.0245 | 4.94E-06 | 20.86 |
| IL-6 | rs10910395 | 1 | A | T | -0.108 | 0.8729 | 0.0235 | 4.31E-06 | 21.12 |
| IL-6 | rs10982193 | 9 | A | G | -0.0793 | 0.2822 | 0.0174 | 5.18E-06 | 20.77 |
| IL-6 | rs113098456 | 2 | A | G | -0.1553 | 0.0725 | 0.0339 | 4.62E-06 | 20.99 |
| IL-6 | rs113600793 | 17 | A | C | 0.1736 | 0.0623 | 0.0359 | 1.33E-06 | 23.38 |
| IL-6 | rs114373846 | 3 | T | C | 0.4196 | 0.0085 | 0.0905 | 3.54E-06 | 21.50 |
| IL-6 | rs11732981 | 4 | A | C | 0.0722 | 0.5119 | 0.0156 | 3.69E-06 | 21.42 |
| IL-6 | rs1333040 | 9 | T | C | 0.0747 | 0.4508 | 0.0157 | 1.96E-06 | 22.64 |
| IL-6 | rs13412535 | 2 | A | G | -0.1186 | 0.189 | 0.0214 | 2.99E-08 | 30.71 |
| IL-6 | rs4684700 | 3 | T | C | -0.0747 | 0.5142 | 0.0162 | 4.01E-06 | 21.26 |
| IL-6 | rs73273528 | 20 | T | C | 0.268 | 0.0211 | 0.0553 | 1.26E-06 | 23.49 |
| IL-6 | rs76856708 | 16 | T | C | 0.336 | 0.9856 | 0.0697 | 1.43E-06 | 23.24 |
| IL-7 | rs115215018 | 4 | T | C | 0.5985 | 0.0101 | 0.1308 | 4.75E-06 | 20.94 |
| IL-7 | rs117509142 | 8 | T | C | -0.3213 | 0.9569 | 0.0684 | 2.64E-06 | 22.07 |
| IL-7 | rs11757972 | 6 | T | C | 0.121 | 0.4776 | 0.0257 | 2.50E-06 | 22.17 |
| IL-7 | rs1374279 | 2 | A | T | 0.1625 | 0.143 | 0.0347 | 2.83E-06 | 21.93 |
| IL-7 | rs142397827 | 5 | A | C | 0.4592 | 0.0178 | 0.0994 | 3.84E-06 | 21.34 |
| IL-7 | rs17091524 | 14 | T | C | 0.5092 | 0.9849 | 0.1015 | 5.26E-07 | 25.17 |
| IL-7 | rs218238 | 4 | A | T | 0.1319 | 0.7729 | 0.0284 | 3.41E-06 | 21.57 |
| IL-7 | rs28793375 | 8 | T | C | 0.1644 | 0.1305 | 0.036 | 4.96E-06 | 20.85 |
| IL-7 | rs62006410 | 14 | T | C | -0.1492 | 0.245 | 0.0302 | 7.80E-07 | 24.41 |
| IL-7 | rs7155170 | 14 | A | T | -0.1236 | 0.2744 | 0.027 | 4.70E-06 | 20.96 |
| IL-7 | rs77318030 | 19 | T | C | -0.2966 | 0.9574 | 0.0631 | 2.60E-06 | 22.09 |
| IL-7 | rs7739450 | 6 | A | G | -0.2907 | 0.5167 | 0.0252 | 8.72E-31 | 133.07 |
| IL-7 | rs77981494 | 16 | T | C | -0.5201 | 0.984 | 0.1055 | 8.23E-07 | 24.30 |
| IL-7 | rs78346957 | 10 | A | G | 0.4632 | 0.0161 | 0.1008 | 4.32E-06 | 21.12 |
| IL-8 | rs113487695 | 7 | A | C | -0.6129 | 0.9914 | 0.1292 | 2.10E-06 | 22.50 |
| IL-8 | rs116726256 | 2 | T | C | -0.2247 | 0.9343 | 0.0489 | 4.33E-06 | 21.11 |
| IL-8 | rs12075 | 1 | A | G | 0.1148 | 0.5353 | 0.0235 | 1.03E-06 | 23.86 |
| IL-8 | rs12912642 | 15 | A | G | 0.1168 | 0.3302 | 0.0251 | 3.27E-06 | 21.65 |
| IL-8 | rs183628733 | 1 | T | C | 0.6547 | 0.9917 | 0.1417 | 3.83E-06 | 21.35 |
| IL-8 | rs2673604 | 8 | A | C | -0.118 | 0.6829 | 0.0254 | 3.39E-06 | 21.58 |
| IL-8 | rs3786107 | 17 | A | G | 0.2463 | 0.9287 | 0.0517 | 1.90E-06 | 22.70 |
| IL-8 | rs75840288 | 16 | A | C | 0.5125 | 0.9839 | 0.1121 | 4.84E-06 | 20.90 |
| IL-9 | rs117807175 | 14 | C | G | -0.5225 | 0.0121 | 0.1106 | 2.31E-06 | 22.32 |
| IL-9 | rs1259728 | 12 | A | G | -0.2381 | 0.0554 | 0.0507 | 2.65E-06 | 22.05 |
| IL-9 | rs3736858 | 13 | C | G | -0.1351 | 0.7976 | 0.0291 | 3.44E-06 | 21.55 |
| IL-9 | rs41294750 | 1 | T | C | 0.3442 | 0.0308 | 0.0736 | 2.92E-06 | 21.87 |
| IL-9 | rs4880409 | 10 | T | C | -0.3552 | 0.9664 | 0.0716 | 7.02E-07 | 24.61 |
| IL-9 | rs73443903 | 6 | A | C | 0.2162 | 0.0685 | 0.046 | 2.60E-06 | 22.09 |
| IP-10 | rs113183470 | 6 | A | T | -0.2414 | 0.946 | 0.0524 | 4.09E-06 | 21.22 |
| IP-10 | rs113831257 | 4 | A | G | 0.3639 | 0.0417 | 0.0641 | 1.37E-08 | 32.23 |
| IP-10 | rs12714300 | 2 | A | T | -0.1573 | 0.1378 | 0.0338 | 3.26E-06 | 21.66 |
| IP-10 | rs143799975 | 4 | A | G | -0.7551 | 0.9949 | 0.1638 | 4.03E-06 | 21.25 |
| IP-10 | rs34383175 | 8 | T | C | -0.3196 | 0.035 | 0.0653 | 9.86E-07 | 23.95 |
| IP-10 | rs397816 | 22 | T | C | 0.1211 | 0.5891 | 0.0248 | 1.04E-06 | 23.84 |
| IP-10 | rs4859940 | 4 | C | G | -0.1204 | 0.295 | 0.0258 | 3.06E-06 | 21.78 |
| IP-10 | rs4862110 | 4 | T | C | -0.1453 | 0.771 | 0.0318 | 4.90E-06 | 20.88 |
| IP-10 | rs75970138 | 9 | A | G | -0.4845 | 0.0139 | 0.1037 | 2.98E-06 | 21.83 |
| IP-10 | rs7645625 | 3 | T | G | -0.1116 | 0.5724 | 0.0236 | 2.26E-06 | 22.36 |
| IP-10 | rs79848609 | 15 | A | C | 0.2514 | 0.9449 | 0.0535 | 2.61E-06 | 22.08 |
| IP-10 | rs8112618 | 19 | A | G | 0.1388 | 0.1841 | 0.0297 | 2.96E-06 | 21.84 |
| M-CSF | rs116274860 | 3 | T | G | 0.8262 | 0.9908 | 0.1739 | 2.02E-06 | 22.57 |
| M-CSF | rs116887628 | 8 | A | G | -0.2741 | 0.0611 | 0.0598 | 4.57E-06 | 21.01 |
| M-CSF | rs117867915 | 18 | T | C | 0.5224 | 0.9793 | 0.1096 | 1.88E-06 | 22.72 |
| M-CSF | rs11963606 | 6 | C | G | -0.5353 | 0.9826 | 0.117 | 4.76E-06 | 20.93 |
| M-CSF | rs12962919 | 18 | T | C | 0.3025 | 0.0644 | 0.0659 | 4.43E-06 | 21.07 |
| M-CSF | rs139457375 | 8 | A | C | -0.4047 | 0.9668 | 0.0854 | 2.15E-06 | 22.46 |
| M-CSF | rs147378920 | 1 | A | G | -0.6064 | 0.9871 | 0.1318 | 4.21E-06 | 21.17 |
| M-CSF | rs34089869 | 2 | T | C | 0.2194 | 0.1039 | 0.0462 | 2.05E-06 | 22.55 |
| M-CSF | rs62294910 | 3 | A | G | 0.3472 | 0.0504 | 0.0687 | 4.33E-07 | 25.54 |
| M-CSF | rs72723242 | 5 | T | G | -0.4969 | 0.0177 | 0.1083 | 4.47E-06 | 21.05 |
| M-CSF | rs9387100 | 6 | T | C | -0.135 | 0.4291 | 0.029 | 3.24E-06 | 21.67 |
| M-CSF | rs9626985 | 22 | T | C | 0.2277 | 0.0936 | 0.0496 | 4.42E-06 | 21.07 |
| MCP-1-MCAF | rs111995966 | 2 | T | G | 0.1428 | 0.9297 | 0.0309 | 3.81E-06 | 21.36 |
| MCP-1-MCAF | rs112313229 | 3 | A | G | -0.1652 | 0.0708 | 0.0312 | 1.19E-07 | 28.04 |
| MCP-1-MCAF | rs11920996 | 3 | T | C | 0.1805 | 0.051 | 0.0376 | 1.58E-06 | 23.05 |
| MCP-1-MCAF | rs12062235 | 1 | T | G | 0.1477 | 0.9377 | 0.032 | 3.92E-06 | 21.30 |
| MCP-1-MCAF | rs12493953 | 3 | A | G | -0.0948 | 0.3224 | 0.0172 | 3.56E-08 | 30.38 |
| MCP-1-MCAF | rs143815843 | 2 | A | G | -0.2049 | 0.0315 | 0.0447 | 4.56E-06 | 21.01 |
| MCP-1-MCAF | rs145155829 | 1 | T | C | -0.2125 | 0.037 | 0.0461 | 4.04E-06 | 21.25 |
| MCP-1-MCAF | rs16837903 | 1 | A | G | -0.1104 | 0.1237 | 0.0238 | 3.51E-06 | 21.52 |
| MCP-1-MCAF | rs188998783 | 19 | T | C | -0.5659 | 0.0066 | 0.1233 | 4.44E-06 | 21.06 |
| MCP-1-MCAF | rs2229593 | 3 | T | C | 0.2624 | 0.0385 | 0.0405 | 9.23E-11 | 41.98 |
| MCP-1-MCAF | rs2820126 | 1 | T | G | 0.0907 | 0.7898 | 0.0193 | 2.61E-06 | 22.09 |
| MCP-1-MCAF | rs3026968 | 1 | T | C | -0.0896 | 0.3195 | 0.0169 | 1.15E-07 | 28.11 |
| MCP-1-MCAF | rs34190208 | 3 | T | C | 0.1052 | 0.1511 | 0.0219 | 1.56E-06 | 23.08 |
| MCP-1-MCAF | rs56212190 | 1 | T | C | 0.1799 | 0.0519 | 0.0372 | 1.32E-06 | 23.39 |
| MCP-1-MCAF | rs7197349 | 16 | A | G | 0.0971 | 0.8124 | 0.0206 | 2.43E-06 | 22.22 |
| MCP-1-MCAF | rs72705803 | 9 | A | G | -0.2188 | 0.9694 | 0.047 | 3.23E-06 | 21.67 |
| MCP-1-MCAF | rs77116118 | 3 | T | C | -0.4239 | 0.9892 | 0.0824 | 2.68E-07 | 26.47 |
| MCP-1-MCAF | rs7978037 | 12 | A | T | 0.0746 | 0.3863 | 0.016 | 3.12E-06 | 21.74 |
| MCP-1-MCAF | rs79939301 | 3 | A | G | 0.1449 | 0.1081 | 0.0255 | 1.33E-08 | 32.29 |
| MCP-1-MCAF | rs80108502 | 3 | T | C | 0.2463 | 0.0391 | 0.0409 | 1.72E-09 | 36.26 |
| MCP-1-MCAF | rs863002 | 1 | T | C | 0.1135 | 0.4077 | 0.0158 | 6.79E-13 | 51.60 |
| MCP-1-MCAF | rs9317045 | 13 | A | C | 0.1157 | 0.8618 | 0.0235 | 8.51E-07 | 24.24 |
| MCP-3 | rs10892381 | 11 | T | C | 0.2432 | 0.6629 | 0.0473 | 2.72E-07 | 26.44 |
| MCP-3 | rs117286643 | 8 | A | G | 0.6934 | 0.0225 | 0.1474 | 2.55E-06 | 22.13 |
| MCP-3 | rs28394764 | 4 | A | T | 0.597 | 0.9696 | 0.1282 | 3.21E-06 | 21.69 |
| MCP-3 | rs3129806 | 9 | T | C | -0.1975 | 0.5729 | 0.0433 | 5.09E-06 | 20.80 |
| MCP-3 | rs6993671 | 8 | T | C | 0.2041 | 0.5588 | 0.0443 | 4.08E-06 | 21.23 |
| MCP-3 | rs7275485 | 21 | T | C | -0.2218 | 0.2711 | 0.0481 | 4.00E-06 | 21.26 |
| MIF | rs1007888 | 22 | T | C | -0.1275 | 0.6128 | 0.0245 | 1.95E-07 | 27.08 |
| MIF | rs113218956 | 22 | A | G | -0.8789 | 0.0044 | 0.1876 | 2.80E-06 | 21.95 |
| MIF | rs11551183 | 16 | C | G | 0.3666 | 0.9756 | 0.0795 | 4.00E-06 | 21.26 |
| MIF | rs12594190 | 15 | A | G | 0.1321 | 0.7008 | 0.0266 | 6.83E-07 | 24.66 |
| MIF | rs141009259 | 2 | T | C | -0.6194 | 0.9886 | 0.1285 | 1.43E-06 | 23.23 |
| MIF | rs2294689 | 6 | C | G | -0.1338 | 0.3018 | 0.0287 | 3.13E-06 | 21.73 |
| MIF | rs35792361 | 4 | A | G | -0.2586 | 0.0591 | 0.0527 | 9.25E-07 | 24.08 |
| MIF | rs35890933 | 19 | T | G | 0.1676 | 0.8474 | 0.0365 | 4.39E-06 | 21.08 |
| MIF | rs3814097 | 7 | A | G | -0.1163 | 0.5533 | 0.0251 | 3.60E-06 | 21.47 |
| MIF | rs78098071 | 5 | T | C | -0.4583 | 0.9809 | 0.0915 | 5.48E-07 | 25.09 |
| MIG | rs10266753 | 7 | T | C | -0.2016 | 0.9109 | 0.0397 | 3.81E-07 | 25.79 |
| MIG | rs111607343 | 19 | A | G | -0.5235 | 0.0134 | 0.1119 | 2.89E-06 | 21.89 |
| MIG | rs11177248 | 12 | A | G | 0.3157 | 0.0351 | 0.0667 | 2.21E-06 | 22.40 |
| MIG | rs113302091 | 14 | T | C | 0.2537 | 0.0468 | 0.0553 | 4.48E-06 | 21.05 |
| MIG | rs13143163 | 4 | C | G | 0.2735 | 0.0512 | 0.0582 | 2.61E-06 | 22.08 |
| MIG | rs139010077 | 3 | T | C | 0.4337 | 0.0169 | 0.0943 | 4.24E-06 | 21.15 |
| MIG | rs191555775 | 6 | A | T | 0.2279 | 0.909 | 0.0412 | 3.17E-08 | 30.60 |
| MIG | rs192433162 | 10 | A | G | -0.8045 | 0.0061 | 0.1676 | 1.59E-06 | 23.04 |
| MIG | rs3733233 | 4 | T | C | 0.1223 | 0.6588 | 0.025 | 9.98E-07 | 23.93 |
| MIG | rs62562991 | 9 | A | G | 0.6239 | 0.0097 | 0.1259 | 7.21E-07 | 24.56 |
| MIG | rs6679677 | 1 | A | C | 0.1628 | 0.1458 | 0.0327 | 6.40E-07 | 24.79 |
| MIG | rs8127917 | 21 | T | G | 0.2382 | 0.0608 | 0.0492 | 1.29E-06 | 23.44 |
| MIG | rs816960 | 13 | T | C | -0.1179 | 0.3692 | 0.0242 | 1.11E-06 | 23.74 |
| MIG | rs9456663 | 6 | T | C | -0.1186 | 0.6702 | 0.0255 | 3.30E-06 | 21.63 |
| MIP-1A | rs116615337 | 1 | A | G | 0.1286 | 0.4232 | 0.0278 | 3.73E-06 | 21.40 |
| MIP-1A | rs117506943 | 11 | T | C | 0.3128 | 0.0323 | 0.0682 | 4.51E-06 | 21.04 |
| MIP-1A | rs12159394 | 22 | A | G | -0.1708 | 0.1183 | 0.0366 | 3.06E-06 | 21.78 |
| MIP-1A | rs57786342 | 14 | A | G | 0.139 | 0.2336 | 0.0283 | 9.03E-07 | 24.12 |
| MIP-1A | rs6900267 | 6 | A | C | -0.2472 | 0.9308 | 0.0515 | 1.59E-06 | 23.04 |
| MIP-1A | rs6956239 | 7 | T | C | 0.119 | 0.2927 | 0.026 | 4.72E-06 | 20.95 |
| MIP-1B | rs111942332 | 17 | T | G | -0.4711 | 0.9782 | 0.0571 | 1.58E-16 | 68.07 |
| MIP-1B | rs112337896 | 17 | A | G | 0.2891 | 0.0288 | 0.063 | 4.46E-06 | 21.06 |
| MIP-1B | rs114933663 | 3 | T | C | 0.3178 | 0.0694 | 0.0307 | 4.11E-25 | 107.16 |
| MIP-1B | rs116237296 | 1 | A | G | 0.5284 | 0.0054 | 0.1115 | 2.15E-06 | 22.46 |
| MIP-1B | rs117010890 | 17 | T | C | -0.2043 | 0.9347 | 0.0393 | 2.01E-07 | 27.02 |
| MIP-1B | rs117084209 | 17 | C | G | 0.2445 | 0.0365 | 0.0428 | 1.11E-08 | 32.63 |
| MIP-1B | rs117139712 | 17 | T | C | -0.2669 | 0.0277 | 0.05 | 9.40E-08 | 28.49 |
| MIP-1B | rs11716293 | 3 | C | G | 0.0986 | 0.7698 | 0.0189 | 1.82E-07 | 27.22 |
| MIP-1B | rs117394484 | 17 | T | C | -0.4266 | 0.0126 | 0.0786 | 5.72E-08 | 29.46 |
| MIP-1B | rs117453826 | 17 | A | G | -0.5907 | 0.981 | 0.0591 | 1.60E-23 | 99.90 |
| MIP-1B | rs117503347 | 17 | T | C | 0.3039 | 0.98 | 0.062 | 9.51E-07 | 24.03 |
| MIP-1B | rs117657747 | 18 | A | G | 0.2089 | 0.0526 | 0.0453 | 4.00E-06 | 21.27 |
| MIP-1B | rs12452320 | 17 | A | C | 0.2139 | 0.0716 | 0.0319 | 2.01E-11 | 44.96 |
| MIP-1B | rs12951603 | 17 | A | G | -0.1132 | 0.1492 | 0.0225 | 4.88E-07 | 25.31 |
| MIP-1B | rs141793738 | 17 | A | G | 0.1815 | 0.0432 | 0.0389 | 3.07E-06 | 21.77 |
| MIP-1B | rs1437220 | 17 | T | C | 0.1437 | 0.9207 | 0.0315 | 5.07E-06 | 20.81 |
| MIP-1B | rs145526037 | 3 | T | G | -0.1863 | 0.9604 | 0.0406 | 4.46E-06 | 21.06 |
| MIP-1B | rs146565944 | 17 | T | C | 0.2863 | 0.0235 | 0.0558 | 2.88E-07 | 26.33 |
| MIP-1B | rs148561432 | 17 | A | G | -0.2691 | 0.0454 | 0.0407 | 3.80E-11 | 43.72 |
| MIP-1B | rs1543292 | 17 | A | G | 0.2627 | 0.0632 | 0.0338 | 7.71E-15 | 60.41 |
| MIP-1B | rs159309 | 17 | T | C | 0.1254 | 0.1102 | 0.0258 | 1.17E-06 | 23.62 |
| MIP-1B | rs17138331 | 7 | A | G | -0.1434 | 0.9092 | 0.0295 | 1.17E-06 | 23.63 |
| MIP-1B | rs1867288 | 17 | C | G | 0.2034 | 0.2288 | 0.0215 | 3.07E-21 | 89.50 |
| MIP-1B | rs191600590 | 3 | A | T | 0.1449 | 0.1326 | 0.0246 | 3.86E-09 | 34.69 |
| MIP-1B | rs2131092 | 3 | A | G | -0.1278 | 0.1103 | 0.0248 | 2.56E-07 | 26.56 |
| MIP-1B | rs2276857 | 3 | T | C | -0.1283 | 0.1356 | 0.0257 | 5.97E-07 | 24.92 |
| MIP-1B | rs2314809 | 17 | T | C | -0.0735 | 0.5305 | 0.0157 | 2.85E-06 | 21.92 |
| MIP-1B | rs2373048 | 3 | A | T | -0.116 | 0.1789 | 0.0215 | 6.84E-08 | 29.11 |
| MIP-1B | rs2376263 | 17 | A | G | 0.1053 | 0.7795 | 0.0187 | 1.79E-08 | 31.71 |
| MIP-1B | rs2411161 | 17 | T | C | 0.1719 | 0.9508 | 0.0365 | 2.48E-06 | 22.18 |
| MIP-1B | rs2673059 | 3 | T | C | 0.0921 | 0.7787 | 0.0192 | 1.61E-06 | 23.01 |
| MIP-1B | rs281728 | 8 | A | C | -0.079 | 0.7058 | 0.0171 | 3.84E-06 | 21.34 |
| MIP-1B | rs28393318 | 4 | A | G | -0.1076 | 0.8758 | 0.0235 | 4.68E-06 | 20.96 |
| MIP-1B | rs28856610 | 17 | T | C | -0.3074 | 0.031 | 0.0495 | 5.30E-10 | 38.57 |
| MIP-1B | rs323877 | 3 | C | G | -0.0965 | 0.6443 | 0.0172 | 2.02E-08 | 31.48 |
| MIP-1B | rs34437725 | 17 | T | C | -0.2571 | 0.9697 | 0.0481 | 9.04E-08 | 28.57 |
| MIP-1B | rs35933743 | 17 | T | G | -0.1183 | 0.8438 | 0.0238 | 6.68E-07 | 24.71 |
| MIP-1B | rs41290648 | 3 | A | G | 0.2231 | 0.1266 | 0.0239 | 1.01E-20 | 87.14 |
| MIP-1B | rs41341749 | 17 | A | G | -0.1656 | 0.9203 | 0.0294 | 1.77E-08 | 31.73 |
| MIP-1B | rs41502550 | 3 | T | C | 0.1266 | 0.8499 | 0.022 | 8.69E-09 | 33.11 |
| MIP-1B | rs4795931 | 17 | A | G | -0.0865 | 0.3193 | 0.0173 | 5.73E-07 | 25.00 |
| MIP-1B | rs4796110 | 17 | A | G | 0.1244 | 0.8879 | 0.0256 | 1.18E-06 | 23.61 |
| MIP-1B | rs56083628 | 17 | T | C | -0.1297 | 0.1054 | 0.0257 | 4.50E-07 | 25.47 |
| MIP-1B | rs62079535 | 17 | A | G | 0.2309 | 0.9572 | 0.0389 | 2.93E-09 | 35.23 |
| MIP-1B | rs6908843 | 6 | A | G | 0.0997 | 0.1684 | 0.0209 | 1.84E-06 | 22.76 |
| MIP-1B | rs71381491 | 17 | A | C | 0.3777 | 0.9746 | 0.0561 | 1.67E-11 | 45.33 |
| MIP-1B | rs72791296 | 5 | T | C | 0.2364 | 0.0349 | 0.0466 | 3.92E-07 | 25.73 |
| MIP-1B | rs72799710 | 5 | T | C | -0.1037 | 0.1514 | 0.0217 | 1.76E-06 | 22.84 |
| MIP-1B | rs72820112 | 17 | T | C | 0.1059 | 0.7412 | 0.0183 | 7.17E-09 | 33.49 |
| MIP-1B | rs72820246 | 17 | T | G | -0.0983 | 0.4163 | 0.0167 | 3.95E-09 | 34.65 |
| MIP-1B | rs72825991 | 17 | A | G | -0.179 | 0.0709 | 0.0312 | 9.63E-09 | 32.92 |
| MIP-1B | rs72829264 | 17 | A | G | -0.1517 | 0.8833 | 0.0277 | 4.34E-08 | 29.99 |
| MIP-1B | rs74979864 | 7 | A | T | -0.3184 | 0.0169 | 0.0613 | 2.06E-07 | 26.98 |
| MIP-1B | rs76582507 | 9 | A | G | 0.3259 | 0.0156 | 0.0676 | 1.43E-06 | 23.24 |
| MIP-1B | rs76842834 | 17 | T | C | -0.4207 | 0.0371 | 0.0471 | 4.18E-19 | 79.78 |
| MIP-1B | rs76863419 | 17 | T | G | -0.2833 | 0.073 | 0.0341 | 9.74E-17 | 69.02 |
| MIP-1B | rs79088462 | 17 | T | C | -0.3214 | 0.9782 | 0.0584 | 3.72E-08 | 30.29 |
| MIP-1B | rs80322601 | 17 | T | C | -0.1915 | 0.9468 | 0.038 | 4.67E-07 | 25.40 |
| MIP-1B | rs8081726 | 17 | T | C | -0.3499 | 0.0491 | 0.036 | 2.49E-22 | 94.47 |
| MIP-1B | rs854222 | 3 | A | C | 0.0809 | 0.2696 | 0.0177 | 4.86E-06 | 20.89 |
| MIP-1B | rs873944 | 17 | T | C | 0.2392 | 0.0726 | 0.0322 | 1.10E-13 | 55.18 |
| MIP-1B | rs939408 | 3 | A | C | -0.1007 | 0.3938 | 0.016 | 3.10E-10 | 39.61 |
| MIP-1B | rs951814 | 17 | A | G | 0.1614 | 0.07 | 0.0315 | 2.99E-07 | 26.25 |
| MIP-1B | rs9793308 | 1 | A | G | 0.0842 | 0.6036 | 0.0177 | 1.96E-06 | 22.63 |
| MIP-1B | rs9838883 | 3 | T | C | 0.079 | 0.3399 | 0.0166 | 1.95E-06 | 22.65 |
| MIP-1B | rs9911839 | 17 | T | G | -0.1377 | 0.9011 | 0.027 | 3.40E-07 | 26.01 |
| MIP-1B | rs9914803 | 17 | T | C | 0.098 | 0.5189 | 0.0156 | 3.34E-10 | 39.46 |
| PDGF-BB | rs10512952 | 5 | T | C | -0.2816 | 0.9819 | 0.0587 | 1.61E-06 | 23.01 |
| PDGF-BB | rs111996132 | 2 | A | C | -0.3127 | 0.9789 | 0.0561 | 2.49E-08 | 31.07 |
| PDGF-BB | rs116154010 | 2 | T | C | 0.3225 | 0.0179 | 0.0662 | 1.11E-06 | 23.73 |
| PDGF-BB | rs11766649 | 7 | A | G | 0.0902 | 0.8018 | 0.0196 | 4.18E-06 | 21.18 |
| PDGF-BB | rs12289510 | 11 | A | G | -0.0772 | 0.4767 | 0.0158 | 1.03E-06 | 23.87 |
| PDGF-BB | rs12615784 | 2 | T | C | -0.1003 | 0.7849 | 0.0193 | 2.03E-07 | 27.01 |
| PDGF-BB | rs13037046 | 20 | A | T | -0.0948 | 0.1871 | 0.0206 | 4.19E-06 | 21.18 |
| PDGF-BB | rs147862316 | 2 | T | C | 0.2279 | 0.0371 | 0.0411 | 2.94E-08 | 30.75 |
| PDGF-BB | rs192743385 | 15 | T | C | 0.1807 | 0.0518 | 0.0392 | 4.03E-06 | 21.25 |
| PDGF-BB | rs2643354 | 15 | A | G | 0.1251 | 0.8921 | 0.0261 | 1.64E-06 | 22.97 |
| PDGF-BB | rs34131731 | 2 | A | T | 0.2083 | 0.9594 | 0.0411 | 4.02E-07 | 25.69 |
| PDGF-BB | rs35859699 | 4 | A | G | -0.3854 | 0.0109 | 0.0838 | 4.24E-06 | 21.15 |
| PDGF-BB | rs62191444 | 20 | T | G | -0.112 | 0.1492 | 0.0239 | 2.78E-06 | 21.96 |
| PDGF-BB | rs6910518 | 6 | T | G | 0.0806 | 0.5924 | 0.0162 | 6.51E-07 | 24.75 |
| PDGF-BB | rs7170232 | 15 | T | C | 0.1609 | 0.219 | 0.0189 | 1.69E-17 | 72.48 |
| PDGF-BB | rs72958564 | 2 | A | T | -0.4097 | 0.9901 | 0.0864 | 2.12E-06 | 22.49 |
| PDGF-BB | rs72972467 | 2 | C | G | -0.1616 | 0.0623 | 0.0328 | 8.36E-07 | 24.27 |
| PDGF-BB | rs73162807 | 3 | A | C | -0.2313 | 0.0259 | 0.0499 | 3.56E-06 | 21.49 |
| PDGF-BB | rs9924851 | 16 | C | G | 0.0767 | 0.3564 | 0.0163 | 2.53E-06 | 22.14 |
| RANTES | rs10505135 | 8 | T | C | 0.1315 | 0.3704 | 0.0252 | 1.81E-07 | 27.23 |
| RANTES | rs118096511 | 13 | T | C | -0.3374 | 0.9696 | 0.0709 | 1.95E-06 | 22.65 |
| RANTES | rs11873385 | 18 | A | G | -0.2582 | 0.9466 | 0.0552 | 2.90E-06 | 21.88 |
| RANTES | rs148526102 | 19 | T | C | -0.3798 | 0.0215 | 0.083 | 4.74E-06 | 20.94 |
| RANTES | rs2731672 | 5 | T | C | -0.1242 | 0.267 | 0.0272 | 4.97E-06 | 20.85 |
| RANTES | rs4795087 | 17 | C | G | 0.1494 | 0.8188 | 0.0312 | 1.68E-06 | 22.93 |
| RANTES | rs62438851 | 6 | A | G | -0.1904 | 0.8961 | 0.0413 | 4.02E-06 | 21.25 |
| RANTES | rs7170339 | 15 | C | G | -0.4283 | 0.0197 | 0.0904 | 2.16E-06 | 22.45 |
| RANTES | rs72793342 | 16 | A | G | -0.1505 | 0.2003 | 0.0307 | 9.47E-07 | 24.03 |
| RANTES | rs78050316 | 2 | A | C | 0.4202 | 0.0204 | 0.0859 | 1.00E-06 | 23.93 |
| SCF | rs10800449 | 1 | A | C | 0.0851 | 0.2859 | 0.0179 | 1.99E-06 | 22.60 |
| SCF | rs11244035 | 9 | T | C | -0.1296 | 0.0944 | 0.0279 | 3.40E-06 | 21.58 |
| SCF | rs113127926 | 14 | A | C | 0.1974 | 0.0418 | 0.0418 | 2.33E-06 | 22.30 |
| SCF | rs117721699 | 9 | C | G | -0.2392 | 0.0265 | 0.0484 | 7.73E-07 | 24.42 |
| SCF | rs12345108 | 9 | T | C | -0.0772 | 0.3395 | 0.0167 | 3.79E-06 | 21.37 |
| SCF | rs13412535 | 2 | A | G | -0.1065 | 0.1892 | 0.0213 | 5.73E-07 | 25.00 |
| SCF | rs138538809 | 8 | T | C | -0.5788 | 0.0052 | 0.1139 | 3.74E-07 | 25.82 |
| SCF | rs1536480 | 9 | T | C | 0.081 | 0.3204 | 0.0167 | 1.23E-06 | 23.53 |
| SCF | rs72678285 | 14 | A | T | 0.1062 | 0.8512 | 0.0231 | 4.28E-06 | 21.14 |
| SCF | rs72832071 | 16 | A | G | 0.2238 | 0.9732 | 0.0482 | 3.43E-06 | 21.56 |
| SCF | rs78666213 | 4 | T | G | -0.2845 | 0.9797 | 0.0574 | 7.18E-07 | 24.57 |
| SCF | rs8045376 | 16 | A | G | -0.3126 | 0.0144 | 0.068 | 4.28E-06 | 21.13 |
| SCGF-B | rs112346514 | 19 | T | C | -0.3261 | 0.0302 | 0.0703 | 3.51E-06 | 21.52 |
| SCGF-B | rs1149926 | 10 | T | C | -0.3458 | 0.0239 | 0.0749 | 3.90E-06 | 21.32 |
| SCGF-B | rs118003677 | 12 | T | C | -0.3654 | 0.9775 | 0.0786 | 3.34E-06 | 21.61 |
| SCGF-B | rs12118918 | 1 | A | G | -0.1631 | 0.8669 | 0.035 | 3.16E-06 | 21.72 |
| SCGF-B | rs12480722 | 20 | T | C | 0.1654 | 0.8752 | 0.0353 | 2.79E-06 | 21.95 |
| SCGF-B | rs13287050 | 9 | A | T | -0.121 | 0.7202 | 0.0263 | 4.21E-06 | 21.17 |
| SCGF-B | rs13866 | 19 | T | C | -0.1647 | 0.2622 | 0.028 | 4.05E-09 | 34.60 |
| SCGF-B | rs139413256 | 7 | A | G | -0.5174 | 0.0139 | 0.1076 | 1.52E-06 | 23.12 |
| SCGF-B | rs143829871 | 3 | T | C | -0.1866 | 0.9047 | 0.0399 | 2.92E-06 | 21.87 |
| SCGF-B | rs144724875 | 19 | T | C | 0.5381 | 0.0274 | 0.0829 | 8.53E-11 | 42.13 |
| SCGF-B | rs149009264 | 10 | A | G | 0.4551 | 0.9848 | 0.0985 | 3.83E-06 | 21.35 |
| SCGF-B | rs150733161 | 13 | T | C | -0.5255 | 0.0142 | 0.112 | 2.71E-06 | 22.01 |
| SCGF-B | rs151194174 | 7 | A | G | 0.4536 | 0.0187 | 0.0941 | 1.43E-06 | 23.24 |
| SCGF-B | rs264157 | 18 | A | G | 0.1079 | 0.4743 | 0.0233 | 3.64E-06 | 21.45 |
| SCGF-B | rs34911860 | 1 | A | G | -0.3674 | 0.0291 | 0.0787 | 3.04E-06 | 21.79 |
| SCGF-B | rs3817303 | 12 | T | G | 0.1362 | 0.194 | 0.0294 | 3.61E-06 | 21.46 |
| SCGF-B | rs4737731 | 8 | T | C | 0.1146 | 0.3019 | 0.0251 | 4.98E-06 | 20.85 |
| SCGF-B | rs4976691 | 5 | C | G | -0.1484 | 0.3257 | 0.0253 | 4.47E-09 | 34.41 |
| SCGF-B | rs5742627 | 12 | T | C | 0.2625 | 0.045 | 0.0574 | 4.80E-06 | 20.91 |
| SCGF-B | rs77247938 | 12 | A | G | 0.2862 | 0.0655 | 0.0475 | 1.69E-09 | 36.30 |
| SCGF-B | rs77954165 | 9 | T | C | 0.2631 | 0.9528 | 0.0562 | 2.85E-06 | 21.92 |
| SCGF-B | rs7802293 | 7 | T | C | -0.1145 | 0.3779 | 0.0247 | 3.56E-06 | 21.49 |
| SCGF-B | rs78217154 | 8 | T | C | 0.3942 | 0.9796 | 0.0861 | 4.69E-06 | 20.96 |
| SDF-1A | rs10474392 | 5 | A | G | 0.0934 | 0.334 | 0.0177 | 1.31E-07 | 27.84 |
| SDF-1A | rs10516368 | 4 | A | C | -0.4268 | 0.0105 | 0.0883 | 1.34E-06 | 23.36 |
| SDF-1A | rs12141941 | 1 | T | C | -0.0881 | 0.7493 | 0.0186 | 2.17E-06 | 22.43 |
| SDF-1A | rs149893336 | 4 | A | G | -0.494 | 0.993 | 0.1082 | 4.98E-06 | 20.84 |
| SDF-1A | rs1600396 | 4 | A | G | -0.0933 | 0.8096 | 0.0204 | 4.80E-06 | 20.92 |
| SDF-1A | rs62194946 | 2 | T | G | -0.0849 | 0.2628 | 0.0185 | 4.45E-06 | 21.06 |
| SDF-1A | rs6586903 | 8 | T | C | -0.1264 | 0.1037 | 0.0268 | 2.40E-06 | 22.24 |
| SDF-1A | rs76766406 | 4 | A | G | 0.4642 | 0.9935 | 0.1012 | 4.50E-06 | 21.04 |
| SDF-1A | rs78037609 | 21 | A | G | -0.6261 | 0.0104 | 0.1334 | 2.69E-06 | 22.03 |
| SDF-1A | rs78883416 | 10 | C | G | -0.0871 | 0.308 | 0.0182 | 1.70E-06 | 22.90 |
| TNF-A | rs10767536 | 11 | A | G | 0.118 | 0.3274 | 0.0253 | 3.10E-06 | 21.75 |
| TNF-A | rs115018697 | 4 | C | G | -0.9542 | 0.9962 | 0.197 | 1.27E-06 | 23.46 |
| TNF-A | rs116736594 | 5 | T | C | 0.3407 | 0.0312 | 0.0702 | 1.21E-06 | 23.55 |
| TNF-A | rs79105320 | 8 | A | G | 0.5573 | 0.0112 | 0.1177 | 2.19E-06 | 22.42 |
| TNF-B | rs10925040 | 1 | T | C | 0.1738 | 0.371 | 0.0372 | 2.98E-06 | 21.83 |
| TNF-B | rs143259067 | 1 | T | C | -0.6923 | 0.9595 | 0.1003 | 5.12E-12 | 47.64 |
| TNF-B | rs2420873 | 19 | T | G | 0.1673 | 0.3897 | 0.0365 | 4.57E-06 | 21.01 |
| TNF-B | rs62284710 | 3 | A | G | 0.3702 | 0.9443 | 0.0782 | 2.20E-06 | 22.41 |
| TNF-B | rs75240021 | 8 | C | G | 0.3713 | 0.071 | 0.0772 | 1.51E-06 | 23.13 |
| TNF-B | rs76225863 | 1 | A | G | 0.7534 | 0.0293 | 0.1217 | 5.99E-10 | 38.32 |
| TRAIL | rs10084050 | 18 | A | G | -0.1101 | 0.8647 | 0.023 | 1.69E-06 | 22.91 |
| TRAIL | rs10164260 | 18 | A | G | 0.1003 | 0.1741 | 0.0211 | 2.00E-06 | 22.60 |
| TRAIL | rs11081739 | 18 | A | G | 0.1395 | 0.2048 | 0.0202 | 4.99E-12 | 47.69 |
| TRAIL | rs112821861 | 18 | T | G | -0.8566 | 0.9717 | 0.0494 | 2.34E-67 | 300.68 |
| TRAIL | rs113057689 | 3 | A | G | -0.2625 | 0.033 | 0.0489 | 7.96E-08 | 28.82 |
| TRAIL | rs11875481 | 18 | T | C | -0.0969 | 0.835 | 0.0211 | 4.38E-06 | 21.09 |
| TRAIL | rs13115587 | 4 | A | C | 0.101 | 0.1558 | 0.0217 | 3.25E-06 | 21.66 |
| TRAIL | rs13278062 | 8 | T | G | 0.08 | 0.5589 | 0.0157 | 3.48E-07 | 25.96 |
| TRAIL | rs139958028 | 11 | A | G | 0.1803 | 0.0501 | 0.0395 | 5.01E-06 | 20.84 |
| TRAIL | rs141032096 | 18 | T | G | -0.2125 | 0.962 | 0.0455 | 3.01E-06 | 21.81 |
| TRAIL | rs146827832 | 3 | T | C | 0.1341 | 0.9038 | 0.0291 | 4.06E-06 | 21.24 |
| TRAIL | rs148051545 | 19 | T | C | -0.4211 | 0.0114 | 0.0843 | 5.88E-07 | 24.95 |
| TRAIL | rs17535790 | 3 | A | G | -0.1125 | 0.1498 | 0.0218 | 2.46E-07 | 26.63 |
| TRAIL | rs183815186 | 18 | A | T | -0.3499 | 0.9803 | 0.0602 | 6.16E-09 | 33.78 |
| TRAIL | rs550057 | 9 | T | C | -0.0783 | 0.3049 | 0.0169 | 3.60E-06 | 21.47 |
| TRAIL | rs558572 | 3 | T | C | 0.1351 | 0.0949 | 0.0265 | 3.43E-07 | 25.99 |
| TRAIL | rs57396456 | 18 | T | C | -0.5641 | 0.9755 | 0.0516 | 8.09E-28 | 119.51 |
| TRAIL | rs62093482 | 18 | T | C | 0.9827 | 0.0237 | 0.0529 | 4.97E-77 | 345.09 |
| TRAIL | rs7233927 | 18 | A | G | 0.0905 | 0.6648 | 0.0164 | 3.42E-08 | 30.45 |
| TRAIL | rs73039026 | 3 | A | C | -0.3098 | 0.9821 | 0.0634 | 1.03E-06 | 23.88 |
| TRAIL | rs73408359 | 18 | T | C | 0.4153 | 0.049 | 0.0364 | 3.76E-30 | 130.17 |
| TRAIL | rs74488044 | 18 | A | G | 0.3473 | 0.0583 | 0.0334 | 2.53E-25 | 108.12 |
| TRAIL | rs747324 | 14 | T | C | -0.0826 | 0.2828 | 0.0178 | 3.48E-06 | 21.53 |
| TRAIL | rs74778900 | 18 | T | C | 0.5791 | 0.0238 | 0.0531 | 1.08E-27 | 118.94 |
| TRAIL | rs75473890 | 18 | T | C | -0.1349 | 0.9136 | 0.028 | 1.45E-06 | 23.21 |
| TRAIL | rs75489499 | 3 | T | C | -0.2006 | 0.0677 | 0.0347 | 7.43E-09 | 33.42 |
| TRAIL | rs75928541 | 4 | A | G | 0.2784 | 0.0188 | 0.0591 | 2.47E-06 | 22.19 |
| TRAIL | rs7599203 | 2 | T | C | 0.0918 | 0.8034 | 0.02 | 4.43E-06 | 21.07 |
| TRAIL | rs79085506 | 18 | A | G | 0.6961 | 0.0154 | 0.0737 | 3.55E-21 | 89.21 |
| TRAIL | rs9946486 | 18 | A | G | 0.1856 | 0.1159 | 0.0245 | 3.58E-14 | 57.39 |
| VEGF | rs10411345 | 19 | C | G | -0.1041 | 0.7823 | 0.0218 | 1.80E-06 | 22.80 |
| VEGF | rs10757514 | 9 | C | G | -0.1024 | 0.1715 | 0.0222 | 3.98E-06 | 21.28 |
| VEGF | rs10822118 | 10 | T | C | -0.0797 | 0.4984 | 0.0168 | 2.09E-06 | 22.51 |
| VEGF | rs10934631 | 3 | T | C | -0.1132 | 0.8517 | 0.0244 | 3.50E-06 | 21.52 |
| VEGF | rs10967183 | 9 | T | C | -0.0887 | 0.4262 | 0.0169 | 1.53E-07 | 27.55 |
| VEGF | rs111950052 | 6 | A | G | -0.1763 | 0.0577 | 0.0383 | 4.16E-06 | 21.19 |
| VEGF | rs114773511 | 6 | T | C | 0.2187 | 0.0463 | 0.0441 | 7.08E-07 | 24.59 |
| VEGF | rs12156533 | 9 | A | T | 0.0915 | 0.6655 | 0.0186 | 8.68E-07 | 24.20 |
| VEGF | rs12456390 | 18 | T | C | -0.0818 | 0.6772 | 0.0179 | 4.88E-06 | 20.88 |
| VEGF | rs13190738 | 6 | T | C | 0.1111 | 0.3644 | 0.0231 | 1.51E-06 | 23.13 |
| VEGF | rs1730969 | 16 | C | G | -0.7811 | 0.0039 | 0.1696 | 4.11E-06 | 21.21 |
| VEGF | rs3025020 | 6 | T | C | -0.124 | 0.2786 | 0.0253 | 9.53E-07 | 24.02 |
| VEGF | rs4573079 | 6 | A | C | 0.1522 | 0.8439 | 0.0256 | 2.76E-09 | 35.35 |
| VEGF | rs56071907 | 16 | T | C | 0.126 | 0.1319 | 0.027 | 3.06E-06 | 21.78 |
| VEGF | rs58078557 | 9 | A | T | -0.1168 | 0.1473 | 0.024 | 1.13E-06 | 23.68 |
| VEGF | rs60013354 | 10 | A | G | -0.2497 | 0.0266 | 0.0521 | 1.65E-06 | 22.97 |
| VEGF | rs60987108 | 6 | A | G | 0.1812 | 0.9459 | 0.039 | 3.38E-06 | 21.59 |
| VEGF | rs6496613 | 15 | A | C | -0.2359 | 0.9677 | 0.0515 | 4.64E-06 | 20.98 |
| VEGF | rs73872715 | 3 | T | C | -0.6079 | 0.0045 | 0.1299 | 2.87E-06 | 21.90 |
| VEGF | rs748227 | 6 | T | C | 0.2377 | 0.0654 | 0.0364 | 6.57E-11 | 42.64 |
| VEGF | rs7739450 | 6 | A | G | -0.415 | 0.5059 | 0.018 | 1.29E-117 | 531.56 |
| VEGF | rs7754905 | 6 | A | G | -0.1303 | 0.7682 | 0.0204 | 1.69E-10 | 40.80 |
| VEGF | rs7757024 | 6 | T | C | 0.1443 | 0.0973 | 0.0291 | 7.09E-07 | 24.59 |
| VEGF | rs77961527 | 3 | A | G | 0.2289 | 0.9606 | 0.0457 | 5.48E-07 | 25.09 |
| VEGF | rs9369440 | 6 | T | C | -0.0872 | 0.2934 | 0.0186 | 2.76E-06 | 21.98 |
| VEGF | rs9381249 | 6 | T | C | -0.2414 | 0.9461 | 0.0396 | 1.09E-09 | 37.16 |
| VEGF | rs9472153 | 6 | A | G | 0.106 | 0.5187 | 0.0174 | 1.12E-09 | 37.11 |

3.Instrumental variables for inflammatory factors and HOA

| **Exposure** | **SNP** | **Chr** | **EA** | **OA** | **Beta** | **EAF** | **SE** | **P** | **F** | |
| --- | --- | --- | --- | --- | --- | --- | --- | --- | --- | --- |
| B-NGF | rs28637706 | 19 | T | G | -0.1554 | 0.314 | 0.0261 | 2.62E-09 | 35.45 | |
| B-NGF | rs4767014 | 12 | T | C | -0.1211 | 0.6731 | 0.0264 | 4.49E-06 | 21.04 | |
| B-NGF | rs71641308 | 1 | T | C | 0.1969 | 0.0995 | 0.0429 | 4.44E-06 | 21.07 | |
| B-NGF | rs73472576 | 18 | T | C | -0.1146 | 0.457 | 0.0251 | 4.98E-06 | 20.85 | |
| CTACK | rs10854859 | 22 | A | G | -0.1498 | 0.2464 | 0.0293 | 3.18E-07 | 26.14 | |
| CTACK | rs116303454 | 3 | A | G | 0.3754 | 0.0218 | 0.081 | 3.58E-06 | 21.48 | |
| CTACK | rs116871507 | 9 | A | T | -0.2086 | 0.0791 | 0.0448 | 3.22E-06 | 21.68 | |
| CTACK | rs116943377 | 13 | A | G | 0.2878 | 0.0394 | 0.0611 | 2.47E-06 | 22.19 | |
| CTACK | rs117932939 | 9 | T | C | 0.1969 | 0.09 | 0.0422 | 3.07E-06 | 21.77 | |
| CTACK | rs118084576 | 9 | A | G | 0.5675 | 0.0112 | 0.1226 | 3.68E-06 | 21.43 | |
| CTACK | rs144072067 | 9 | A | G | 0.4451 | 0.9574 | 0.0579 | 1.50E-14 | 59.10 | |
| CTACK | rs184329319 | 9 | T | G | -0.3069 | 0.035 | 0.0648 | 2.18E-06 | 22.43 | |
| CTACK | rs2233872 | 9 | A | G | -0.1495 | 0.7699 | 0.0282 | 1.15E-07 | 28.11 | |
| CTACK | rs55764737 | 15 | T | C | 0.5424 | 0.9846 | 0.0967 | 2.03E-08 | 31.46 | |
| CTACK | rs57338032 | 15 | A | G | 0.1443 | 0.8287 | 0.0316 | 4.96E-06 | 20.85 | |
| CTACK | rs57789542 | 3 | T | C | -0.7687 | 0.9951 | 0.1659 | 3.60E-06 | 21.47 | |
| CTACK | rs60247384 | 3 | T | C | 0.1128 | 0.3512 | 0.0245 | 4.14E-06 | 21.20 | |
| CTACK | rs62578137 | 9 | T | C | -0.1311 | 0.2421 | 0.0286 | 4.56E-06 | 21.01 | |
| CTACK | rs72729450 | 9 | T | C | -0.5123 | 0.0123 | 0.1094 | 2.83E-06 | 21.93 | |
| CTACK | rs76395525 | 15 | A | G | 0.5193 | 0.0118 | 0.1081 | 1.56E-06 | 23.08 | |
| EOTAXIN | rs11920996 | 3 | T | C | 0.2979 | 0.0512 | 0.0377 | 2.75E-15 | 62.44 | |
| EOTAXIN | rs147287945 | 6 | A | G | -0.1512 | 0.0741 | 0.0313 | 1.36E-06 | 23.34 | |
| EOTAXIN | rs1677588 | 1 | T | G | 0.1181 | 0.886 | 0.025 | 2.31E-06 | 22.32 | |
| EOTAXIN | rs2024050 | 7 | A | G | 0.164 | 0.0739 | 0.0302 | 5.62E-08 | 29.49 | |
| EOTAXIN | rs2027855 | 22 | T | C | 0.0743 | 0.6153 | 0.0162 | 4.51E-06 | 21.04 | |
| EOTAXIN | rs2040143 | 21 | A | G | -0.0858 | 0.7234 | 0.0178 | 1.43E-06 | 23.23 | |
| EOTAXIN | rs2097947 | 7 | A | T | -0.0769 | 0.6836 | 0.0168 | 4.71E-06 | 20.95 | |
| EOTAXIN | rs2229593 | 3 | T | C | 0.3647 | 0.0387 | 0.0406 | 2.64E-19 | 80.69 | |
| EOTAXIN | rs2249581 | 1 | T | C | -0.0899 | 0.748 | 0.018 | 5.90E-07 | 24.94 | |
| EOTAXIN | rs2828756 | 21 | T | C | 0.0904 | 0.2423 | 0.0184 | 8.97E-07 | 24.14 | |
| EOTAXIN | rs34004101 | 3 | T | C | -0.1775 | 0.065 | 0.0341 | 1.94E-07 | 27.09 | |
| EOTAXIN | rs4683182 | 3 | A | G | 0.0886 | 0.2201 | 0.0189 | 2.76E-06 | 21.98 | |
| EOTAXIN | rs5754733 | 22 | A | C | -0.105 | 0.8306 | 0.0213 | 8.24E-07 | 24.30 | |
| EOTAXIN | rs57723662 | 17 | C | G | -0.0982 | 0.1657 | 0.0213 | 4.02E-06 | 21.26 | |
| EOTAXIN | rs60075014 | 5 | T | C | -0.1688 | 0.0604 | 0.0356 | 2.12E-06 | 22.48 | |
| EOTAXIN | rs7231030 | 18 | A | C | 0.0903 | 0.7919 | 0.0193 | 2.89E-06 | 21.89 | |
| EOTAXIN | rs73065695 | 3 | A | G | 0.1532 | 0.0932 | 0.0281 | 4.98E-08 | 29.72 | |
| EOTAXIN | rs745331 | 15 | A | G | -0.0821 | 0.6825 | 0.0176 | 3.09E-06 | 21.76 | |
| EOTAXIN | rs75426604 | 14 | A | C | -0.1371 | 0.0876 | 0.0291 | 2.46E-06 | 22.20 | |
| EOTAXIN | rs7550207 | 1 | T | C | -0.0904 | 0.7785 | 0.0187 | 1.34E-06 | 23.37 | |
| EOTAXIN | rs9317045 | 13 | A | C | 0.1172 | 0.8615 | 0.0236 | 6.83E-07 | 24.66 | |
| EOTAXIN | rs9833459 | 3 | T | C | -0.1149 | 0.3033 | 0.0173 | 3.10E-11 | 44.11 | |
| EOTAXIN | rs9975149 | 21 | A | T | 0.0749 | 0.3743 | 0.0164 | 4.95E-06 | 20.86 | |
| FGF-BASIC | rs13412535 | 2 | A | G | -0.1129 | 0.1856 | 0.0224 | 4.65E-07 | 25.40 | |
| FGF-BASIC | rs147409637 | 6 | T | C | 0.201 | 0.0369 | 0.0431 | 3.11E-06 | 21.75 | |
| FGF-BASIC | rs17094040 | 14 | T | C | 0.1051 | 0.1522 | 0.0229 | 4.44E-06 | 21.06 | |
| FGF-BASIC | rs2849358 | 18 | A | G | 0.0911 | 0.2551 | 0.0193 | 2.36E-06 | 22.28 | |
| FGF-BASIC | rs4795091 | 17 | A | G | 0.1239 | 0.8926 | 0.0266 | 3.19E-06 | 21.70 | |
| FGF-BASIC | rs76253061 | 5 | T | C | -0.4811 | 0.9925 | 0.1041 | 3.81E-06 | 21.36 | |
| FGF-BASIC | rs78873483 | 17 | A | G | 0.1286 | 0.1025 | 0.0282 | 5.11E-06 | 20.80 | |
| G-CSF | rs10939033 | 4 | A | G | -0.0775 | 0.6038 | 0.0163 | 1.99E-06 | 22.61 | |
| G-CSF | rs117261691 | 19 | T | C | 0.1318 | 0.0894 | 0.0288 | 4.73E-06 | 20.94 | |
| G-CSF | rs183023730 | 9 | T | G | 0.7898 | 0.0034 | 0.1677 | 2.48E-06 | 22.18 | |
| G-CSF | rs586802 | 11 | A | G | 0.0882 | 0.758 | 0.0187 | 2.40E-06 | 22.25 | |
| G-CSF | rs6740648 | 2 | T | C | 0.0818 | 0.6752 | 0.0172 | 1.98E-06 | 22.62 | |
| G-CSF | rs74148555 | 10 | T | C | -0.3771 | 0.0144 | 0.0753 | 5.50E-07 | 25.08 | |
| G-CSF | rs76287671 | 19 | T | C | 0.0894 | 0.2333 | 0.0189 | 2.24E-06 | 22.37 | |
| G-CSF | rs77318030 | 19 | T | C | -0.2031 | 0.9563 | 0.0427 | 1.97E-06 | 22.62 | |
| G-CSF | rs78523761 | 5 | A | G | 0.5374 | 0.0077 | 0.1139 | 2.38E-06 | 22.26 | |
| GROA | rs10015342 | 4 | A | T | 0.1945 | 0.1909 | 0.0304 | 1.57E-10 | 40.93 | |
| GROA | rs114991247 | 6 | T | C | -0.2202 | 0.9226 | 0.0463 | 1.98E-06 | 22.62 | |
| GROA | rs115214168 | 4 | T | C | 0.4528 | 0.0242 | 0.0828 | 4.54E-08 | 29.91 | |
| GROA | rs1361829 | 1 | A | G | -0.1106 | 0.4293 | 0.0241 | 4.45E-06 | 21.06 | |
| GROA | rs140734053 | 10 | A | G | 0.7333 | 0.0066 | 0.1545 | 2.07E-06 | 22.53 | |
| GROA | rs150194856 | 1 | T | C | -0.4223 | 0.0177 | 0.0914 | 3.83E-06 | 21.35 | |
| GROA | rs17171245 | 7 | T | G | 0.2446 | 0.0536 | 0.053 | 3.93E-06 | 21.30 | |
| GROA | rs185768063 | 6 | A | G | 0.4038 | 0.9735 | 0.076 | 1.08E-07 | 28.23 | |
| GROA | rs188345231 | 8 | T | C | 0.6177 | 0.0102 | 0.1322 | 2.98E-06 | 21.83 | |
| GROA | rs3026943 | 1 | A | C | -0.1246 | 0.3311 | 0.0256 | 1.13E-06 | 23.69 | |
| GROA | rs3845622 | 1 | A | C | -0.2345 | 0.1171 | 0.0381 | 7.51E-10 | 37.88 | |
| GROA | rs62024303 | 15 | A | G | -0.3013 | 0.9617 | 0.066 | 4.99E-06 | 20.84 | |
| GROA | rs73020704 | 1 | A | G | 0.2607 | 0.9484 | 0.0545 | 1.72E-06 | 22.88 | |
| GROA | rs76215157 | 20 | C | G | -0.7398 | 0.0074 | 0.1564 | 2.24E-06 | 22.37 | |
| GROA | rs76390238 | 2 | C | G | 0.6223 | 0.0095 | 0.1352 | 4.17E-06 | 21.19 | |
| HGF | rs11060254 | 12 | A | G | -0.0765 | 0.3306 | 0.0166 | 4.06E-06 | 21.24 | |
| HGF | rs11129909 | 3 | T | C | -0.0738 | 0.623 | 0.0161 | 4.56E-06 | 21.01 | |
| HGF | rs13412535 | 2 | A | G | -0.1043 | 0.1892 | 0.0213 | 9.75E-07 | 23.98 | |
| HGF | rs2003620 | 7 | T | C | 0.2277 | 0.027 | 0.0487 | 2.93E-06 | 21.86 | |
| HGF | rs2699434 | 4 | T | C | 0.0863 | 0.3287 | 0.0171 | 4.49E-07 | 25.47 | |
| HGF | rs362307 | 4 | T | C | 0.1511 | 0.0646 | 0.0328 | 4.09E-06 | 21.22 | |
| HGF | rs4245058 | 11 | T | C | -0.1552 | 0.0619 | 0.0331 | 2.75E-06 | 21.99 | |
| HGF | rs57146176 | 12 | A | G | -0.0987 | 0.7998 | 0.0208 | 2.08E-06 | 22.52 | |
| HGF | rs5745687 | 7 | T | C | -0.3008 | 0.0375 | 0.0404 | 9.65E-14 | 55.44 | |
| HGF | rs80051150 | 21 | T | C | 0.198 | 0.9595 | 0.0413 | 1.63E-06 | 22.98 | |
| IFN-G | rs113399544 | 7 | A | G | -0.0849 | 0.2768 | 0.0183 | 3.50E-06 | 21.52 | |
| IFN-G | rs113600793 | 17 | A | C | 0.1871 | 0.0631 | 0.0371 | 4.58E-07 | 25.43 | |
| IFN-G | rs115729819 | 4 | A | G | 0.2511 | 0.9716 | 0.0514 | 1.03E-06 | 23.87 | |
| IFN-G | rs117046255 | 7 | T | C | -0.0968 | 0.1922 | 0.0207 | 2.92E-06 | 21.87 | |
| IFN-G | rs11843756 | 13 | T | G | 0.1812 | 0.9542 | 0.0391 | 3.58E-06 | 21.48 | |
| IFN-G | rs12420286 | 11 | T | C | 0.2357 | 0.9699 | 0.05 | 2.43E-06 | 22.22 | |
| IFN-G | rs147378920 | 1 | A | G | -0.384 | 0.9871 | 0.0751 | 3.17E-07 | 26.14 | |
| IFN-G | rs1867282 | 9 | T | C | 0.0781 | 0.4154 | 0.0166 | 2.54E-06 | 22.14 | |
| IFN-G | rs2073438 | 17 | A | G | 0.092 | 0.2506 | 0.0188 | 9.90E-07 | 23.95 | |
| IFN-G | rs7088799 | 10 | T | G | -0.0805 | 0.6182 | 0.0166 | 1.24E-06 | 23.52 | |
| IFN-G | rs73479333 | 6 | C | G | -0.1123 | 0.1354 | 0.024 | 2.88E-06 | 21.89 | |
| IFN-G | rs74148555 | 10 | T | C | -0.3771 | 0.0144 | 0.077 | 9.71E-07 | 23.98 | |
| IL-10 | rs10457128 | 6 | A | G | -0.0854 | 0.639 | 0.0172 | 6.87E-07 | 24.65 | |
| IL-10 | rs10493718 | 1 | A | C | -0.1081 | 0.1603 | 0.0222 | 1.12E-06 | 23.71 | |
| IL-10 | rs13412535 | 2 | A | G | -0.1347 | 0.1856 | 0.0224 | 1.82E-09 | 36.16 | |
| IL-10 | rs143072171 | 6 | T | C | 0.2089 | 0.0413 | 0.0429 | 1.12E-06 | 23.71 | |
| IL-10 | rs1530455 | 3 | T | C | 0.082 | 0.3523 | 0.0174 | 2.45E-06 | 22.21 | |
| IL-10 | rs181031888 | 6 | A | T | 0.2577 | 0.961 | 0.0472 | 4.77E-08 | 29.81 | |
| IL-10 | rs2086656 | 4 | T | C | -0.08 | 0.6621 | 0.017 | 2.53E-06 | 22.15 | |
| IL-10 | rs3002131 | 1 | C | G | 0.1191 | 0.1195 | 0.026 | 4.63E-06 | 20.98 | |
| IL-10 | rs3025021 | 6 | T | C | 0.0913 | 0.3459 | 0.0194 | 2.52E-06 | 22.15 | |
| IL-10 | rs339203 | 2 | T | C | 0.0954 | 0.8359 | 0.0203 | 2.61E-06 | 22.09 | |
| IL-10 | rs383684 | 6 | A | G | 0.092 | 0.7555 | 0.0197 | 3.01E-06 | 21.81 | |
| IL-10 | rs41282660 | 6 | A | G | -0.1169 | 0.8646 | 0.0254 | 4.18E-06 | 21.18 | |
| IL-10 | rs465757 | 20 | A | G | 0.0806 | 0.6657 | 0.0174 | 3.62E-06 | 21.46 | |
| IL-10 | rs4741748 | 9 | A | G | -0.0788 | 0.6081 | 0.0169 | 3.12E-06 | 21.74 | |
| IL-10 | rs6054847 | 20 | T | C | 0.0971 | 0.8091 | 0.0207 | 2.72E-06 | 22.00 | |
| IL-10 | rs6680918 | 1 | T | C | -0.1202 | 0.8791 | 0.025 | 1.52E-06 | 23.12 | |
| IL-10 | rs7088799 | 10 | T | G | -0.0815 | 0.6191 | 0.0166 | 9.12E-07 | 24.10 | |
| IL-10 | rs73192842 | 3 | A | G | 0.0949 | 0.1911 | 0.0206 | 4.09E-06 | 21.22 | |
| IL-10 | rs7747448 | 6 | A | G | -0.1061 | 0.2594 | 0.0189 | 1.98E-08 | 31.51 | |
| IL-10 | rs865585 | 6 | A | C | -0.119 | 0.8478 | 0.0244 | 1.08E-06 | 23.79 | |
| IL-10 | rs910604 | 6 | A | G | -0.0986 | 0.2383 | 0.0196 | 4.89E-07 | 25.31 | |
| IL-12-P70 | rs113600793 | 17 | A | C | 0.1832 | 0.0623 | 0.0359 | 3.34E-07 | 26.04 | |
| IL-12-P70 | rs12154194 | 6 | T | C | 0.0717 | 0.4615 | 0.0157 | 4.95E-06 | 20.86 | |
| IL-12-P70 | rs12969892 | 18 | T | C | 0.1227 | 0.9031 | 0.0267 | 4.32E-06 | 21.12 | |
| IL-12-P70 | rs13190738 | 6 | T | C | 0.1019 | 0.3609 | 0.019 | 8.18E-08 | 28.76 | |
| IL-12-P70 | rs148449807 | 6 | A | G | -0.4217 | 0.0164 | 0.0793 | 1.05E-07 | 28.28 | |
| IL-12-P70 | rs181031888 | 6 | A | T | 0.2861 | 0.9606 | 0.0456 | 3.52E-10 | 39.36 | |
| IL-12-P70 | rs2123852 | 19 | T | C | 0.0942 | 0.2039 | 0.0204 | 3.88E-06 | 21.32 | |
| IL-12-P70 | rs2495005 | 10 | A | G | -0.075 | 0.4416 | 0.0159 | 2.39E-06 | 22.25 | |
| IL-12-P70 | rs273702 | 18 | A | G | -0.127 | 0.9015 | 0.027 | 2.55E-06 | 22.12 | |
| IL-12-P70 | rs282258 | 2 | T | C | 0.0726 | 0.4279 | 0.0156 | 3.26E-06 | 21.66 | |
| IL-12-P70 | rs34322762 | 9 | T | C | 0.0953 | 0.648 | 0.0199 | 1.68E-06 | 22.93 | |
| IL-12-P70 | rs34826779 | 8 | T | G | -0.0884 | 0.2157 | 0.019 | 3.28E-06 | 21.65 | |
| IL-12-P70 | rs41282644 | 6 | A | G | 0.1401 | 0.0849 | 0.0303 | 3.77E-06 | 21.38 | |
| IL-12-P70 | rs4714698 | 6 | A | G | -0.1189 | 0.8721 | 0.0238 | 5.86E-07 | 24.96 | |
| IL-12-P70 | rs4741748 | 9 | A | G | -0.0799 | 0.6076 | 0.0163 | 9.49E-07 | 24.03 | |
| IL-12-P70 | rs6458375 | 6 | T | C | 0.0884 | 0.2407 | 0.0191 | 3.69E-06 | 21.42 | |
| IL-12-P70 | rs6532374 | 4 | T | C | -0.1033 | 0.8525 | 0.0226 | 4.86E-06 | 20.89 | |
| IL-12-P70 | rs7763358 | 6 | T | C | 0.1525 | 0.0885 | 0.0274 | 2.61E-08 | 30.98 | |
| IL-12-P70 | rs7765264 | 6 | A | G | 0.1428 | 0.0767 | 0.0301 | 2.09E-06 | 22.51 | |
| IL-12-P70 | rs782111 | 12 | A | C | -0.0765 | 0.5292 | 0.0156 | 9.40E-07 | 24.05 | |
| IL-12-P70 | rs865585 | 6 | A | C | -0.1654 | 0.8484 | 0.0237 | 2.97E-12 | 48.71 | |
| IL-12-P70 | rs9381249 | 6 | T | C | -0.1788 | 0.9466 | 0.0367 | 1.11E-06 | 23.74 | |
| IL-12-P70 | rs9472153 | 6 | A | G | 0.0855 | 0.5141 | 0.0159 | 7.56E-08 | 28.92 | |
| IL-12-P70 | rs9472175 | 6 | T | C | -0.1104 | 0.1542 | 0.0238 | 3.51E-06 | 21.52 | |
| IL-13 | rs10995604 | 10 | A | G | -0.1571 | 0.8587 | 0.0343 | 4.65E-06 | 20.98 | |
| IL-13 | rs117795020 | 9 | A | G | -0.3584 | 0.0304 | 0.0716 | 5.57E-07 | 25.06 | |
| IL-13 | rs12199215 | 6 | T | C | 0.1309 | 0.247 | 0.0284 | 4.04E-06 | 21.24 | |
| IL-13 | rs12623722 | 2 | A | G | -0.1189 | 0.3054 | 0.0257 | 3.72E-06 | 21.40 | |
| IL-13 | rs13206012 | 6 | A | G | -0.3719 | 0.3489 | 0.0263 | 2.13E-45 | 199.96 | |
| IL-13 | rs13209117 | 6 | A | G | 0.1409 | 0.245 | 0.0284 | 7.00E-07 | 24.61 | |
| IL-13 | rs138854806 | 6 | A | G | -0.4204 | 0.0247 | 0.0839 | 5.42E-07 | 25.11 | |
| IL-13 | rs139083458 | 5 | T | C | 0.9995 | 0.0034 | 0.211 | 2.17E-06 | 22.44 | |
| IL-13 | rs145023524 | 6 | A | G | 0.2815 | 0.0435 | 0.0588 | 1.69E-06 | 22.92 | |
| IL-13 | rs147747784 | 19 | C | G | 0.369 | 0.0285 | 0.0765 | 1.41E-06 | 23.27 | |
| IL-13 | rs150836197 | 6 | T | C | 0.3283 | 0.0367 | 0.0713 | 4.13E-06 | 21.20 | |
| IL-13 | rs27949 | 5 | T | C | -0.1144 | 0.667 | 0.025 | 4.74E-06 | 20.94 | |
| IL-13 | rs28442067 | 3 | A | G | -0.1379 | 0.7832 | 0.0286 | 1.42E-06 | 23.25 | |
| IL-13 | rs7073807 | 10 | T | C | 0.1618 | 0.1351 | 0.0354 | 4.86E-06 | 20.89 | |
| IL-13 | rs75383097 | 1 | C | G | -0.5369 | 0.0132 | 0.116 | 3.68E-06 | 21.42 | |
| IL-13 | rs76339001 | 21 | A | T | -0.4375 | 0.9767 | 0.0886 | 7.90E-07 | 24.38 | |
| IL-13 | rs76975337 | 3 | T | C | -0.1211 | 0.7286 | 0.0265 | 4.88E-06 | 20.88 | |
| IL-13 | rs7747448 | 6 | A | G | -0.1393 | 0.2585 | 0.0278 | 5.42E-07 | 25.11 | |
| IL-13 | rs7757246 | 6 | T | C | 0.2147 | 0.9092 | 0.0422 | 3.62E-07 | 25.88 | |
| IL-13 | rs77955971 | 6 | A | C | 0.4408 | 0.0247 | 0.0868 | 3.81E-07 | 25.79 | |
| IL-13 | rs9296421 | 6 | T | G | 0.1814 | 0.842 | 0.0348 | 1.86E-07 | 27.17 | |
| IL-16 | rs117217798 | 17 | T | C | -0.2064 | 0.0889 | 0.044 | 2.72E-06 | 22.00 | |
| IL-16 | rs12577604 | 11 | T | C | 0.4335 | 0.9818 | 0.0941 | 4.09E-06 | 21.22 | |
| IL-16 | rs142034902 | 12 | A | G | -0.4367 | 0.9827 | 0.0925 | 2.35E-06 | 22.29 | |
| IL-16 | rs142332135 | 15 | A | G | -0.7646 | 0.0164 | 0.1082 | 1.59E-12 | 49.94 | |
| IL-16 | rs144691581 | 15 | A | G | 0.4929 | 0.0197 | 0.0958 | 2.67E-07 | 26.47 | |
| IL-16 | rs35834666 | 4 | T | C | -0.1729 | 0.8596 | 0.0348 | 6.75E-07 | 24.68 | |
| IL-16 | rs4778640 | 15 | A | G | 0.7189 | 0.9848 | 0.0983 | 2.61E-13 | 53.48 | |
| IL-16 | rs4976691 | 5 | C | G | 0.1254 | 0.3268 | 0.026 | 1.41E-06 | 23.26 | |
| IL-16 | rs7097884 | 10 | T | C | -0.1193 | 0.5647 | 0.0243 | 9.13E-07 | 24.10 | |
| IL-16 | rs78042619 | 9 | A | G | 0.55 | 0.9883 | 0.1158 | 2.04E-06 | 22.56 | |
| IL-17 | rs11640734 | 16 | C | G | -0.115 | 0.8673 | 0.024 | 1.65E-06 | 22.96 | |
| IL-17 | rs11985957 | 8 | A | G | 0.1511 | 0.0642 | 0.0329 | 4.38E-06 | 21.09 | |
| IL-17 | rs12735700 | 1 | T | G | -0.0943 | 0.1925 | 0.0206 | 4.70E-06 | 20.96 | |
| IL-17 | rs148562661 | 6 | C | G | 0.2161 | 0.9605 | 0.0434 | 6.38E-07 | 24.79 | |
| IL-17 | rs17282552 | 2 | T | C | -0.2026 | 0.952 | 0.0403 | 4.97E-07 | 25.27 | |
| IL-17 | rs3804749 | 3 | T | C | -0.0923 | 0.6236 | 0.0167 | 3.26E-08 | 30.55 | |
| IL-17 | rs61990749 | 14 | C | G | 0.1124 | 0.1533 | 0.0226 | 6.58E-07 | 24.74 | |
| IL-17 | rs77341831 | 12 | T | C | 0.2263 | 0.9696 | 0.0473 | 1.72E-06 | 22.89 | |
| IL-17 | rs78296352 | 1 | T | G | 0.2949 | 0.0186 | 0.0645 | 4.83E-06 | 20.90 | |
| IL-17 | rs9519328 | 13 | A | G | 0.5256 | 0.9845 | 0.1101 | 1.81E-06 | 22.79 | |
| IL-17 | rs9568764 | 13 | C | G | 0.0825 | 0.2756 | 0.018 | 4.58E-06 | 21.01 | |
| IL-18 | rs10409850 | 19 | A | G | 0.1791 | 0.8704 | 0.0347 | 2.45E-07 | 26.64 | |
| IL-18 | rs113214367 | 11 | A | G | -0.278 | 0.0565 | 0.0593 | 2.76E-06 | 21.98 | |
| IL-18 | rs117266781 | 7 | T | C | 0.7051 | 0.0073 | 0.1436 | 9.10E-07 | 24.11 | |
| IL-18 | rs117371668 | 16 | T | G | 0.3712 | 0.0254 | 0.0799 | 3.39E-06 | 21.58 | |
| IL-18 | rs12419156 | 11 | T | C | 0.1556 | 0.8064 | 0.0335 | 3.40E-06 | 21.57 | |
| IL-18 | rs139468359 | 5 | T | C | 0.5101 | 0.9866 | 0.1088 | 2.75E-06 | 21.98 | |
| IL-18 | rs139727649 | 5 | T | C | -0.356 | 0.9745 | 0.0751 | 2.13E-06 | 22.47 | |
| IL-18 | rs150005227 | 5 | T | C | 0.4062 | 0.0185 | 0.0886 | 4.55E-06 | 21.02 | |
| IL-18 | rs1979967 | 15 | T | C | 0.14 | 0.2157 | 0.0285 | 9.00E-07 | 24.13 | |
| IL-18 | rs4952239 | 2 | A | T | -0.1156 | 0.3555 | 0.0242 | 1.78E-06 | 22.82 | |
| IL-18 | rs58701153 | 6 | A | T | -0.1265 | 0.6586 | 0.0242 | 1.72E-07 | 27.32 | |
| IL-18 | rs62312914 | 4 | T | C | -0.1265 | 0.3726 | 0.025 | 4.19E-07 | 25.60 | |
| IL-18 | rs7599125 | 2 | A | G | 0.1109 | 0.569 | 0.0239 | 3.48E-06 | 21.53 | |
| IL-18 | rs76138275 | 5 | T | C | 0.1227 | 0.2858 | 0.026 | 2.37E-06 | 22.27 | |
| IL-18 | rs764078 | 11 | A | T | 0.1283 | 0.2239 | 0.0278 | 3.93E-06 | 21.30 | |
| IL-18 | rs77187209 | 5 | T | C | -0.4859 | 0.9866 | 0.1041 | 3.05E-06 | 21.79 | |
| IL-18 | rs78623212 | 7 | T | C | 0.8322 | 0.0053 | 0.1676 | 6.86E-07 | 24.66 | |
| IL-18 | rs78716465 | 20 | A | G | 0.3173 | 0.033 | 0.0679 | 2.97E-06 | 21.84 | |
| IL-1B | rs143319329 | 7 | T | C | 0.4357 | 0.0209 | 0.093 | 2.80E-06 | 21.95 | |
| IL-1B | rs4786740 | 16 | A | C | 0.1264 | 0.3913 | 0.0265 | 1.84E-06 | 22.75 | |
| IL-1B | rs61335305 | 15 | A | C | 0.4333 | 0.0189 | 0.0928 | 3.02E-06 | 21.80 | |
| IL-1B | rs62015704 | 16 | A | G | 0.1786 | 0.8688 | 0.0372 | 1.58E-06 | 23.05 | |
| IL-1RA | rs1054402 | 9 | T | C | 0.1325 | 0.2506 | 0.0269 | 8.41E-07 | 24.26 | |
| IL-1RA | rs117181659 | 22 | A | G | -0.2204 | 0.064 | 0.0478 | 4.01E-06 | 21.26 | |
| IL-1RA | rs11869294 | 17 | C | G | -0.2286 | 0.9205 | 0.047 | 1.15E-06 | 23.66 | |
| IL-1RA | rs13343438 | 19 | A | G | 0.2771 | 0.0434 | 0.0607 | 4.99E-06 | 20.84 | |
| IL-1RA | rs35590641 | 14 | C | G | -0.1167 | 0.3321 | 0.025 | 3.04E-06 | 21.79 | |
| IL-1RA | rs3876037 | 22 | A | G | 0.1234 | 0.6783 | 0.027 | 4.87E-06 | 20.89 | |
| IL-1RA | rs56134659 | 3 | A | G | -0.1109 | 0.4835 | 0.0236 | 2.61E-06 | 22.08 | |
| IL-1RA | rs61335305 | 15 | A | C | 0.4315 | 0.0182 | 0.0904 | 1.81E-06 | 22.78 | |
| IL-1RA | rs6699436 | 1 | A | G | -0.1858 | 0.0995 | 0.0404 | 4.25E-06 | 21.15 | |
| IL-1RA | rs9623661 | 22 | T | C | -0.1948 | 0.0903 | 0.0424 | 4.34E-06 | 21.11 | |
| IL-1RA | rs9985296 | 3 | T | C | 0.1053 | 0.4758 | 0.0231 | 5.15E-06 | 20.78 | |
| IL-2 | rs13412535 | 2 | A | G | 0.174 | 0.1886 | 0.0331 | 1.47E-07 | 27.63 | |
| IL-2 | rs16836080 | 3 | A | G | 0.1158 | 0.3355 | 0.0253 | 4.72E-06 | 20.95 | |
| IL-2 | rs2690020 | 1 | A | G | 0.1158 | 0.5177 | 0.0245 | 2.28E-06 | 22.34 | |
| IL-2 | rs4479767 | 4 | A | G | 0.1821 | 0.8977 | 0.0392 | 3.39E-06 | 21.58 | |
| IL-2 | rs4634519 | 7 | A | G | -0.1249 | 0.7379 | 0.0268 | 3.16E-06 | 21.72 | |
| IL-2 | rs61335305 | 15 | A | C | 0.4439 | 0.0184 | 0.0913 | 1.16E-06 | 23.64 | |
| IL-2 | rs62124990 | 2 | T | G | -0.7013 | 0.0083 | 0.149 | 2.52E-06 | 22.15 | |
| IL-2 | rs7615304 | 3 | A | G | -0.1139 | 0.4531 | 0.024 | 2.08E-06 | 22.52 | |
| IL-2RA | rs11241559 | 5 | T | G | -0.124 | 0.2643 | 0.0264 | 2.64E-06 | 22.06 | |
| IL-2RA | rs115360066 | 5 | A | G | 0.1776 | 0.8882 | 0.0377 | 2.47E-06 | 22.19 | |
| IL-2RA | rs117244812 | 17 | A | G | -0.7187 | 0.0079 | 0.1493 | 1.48E-06 | 23.17 | |
| IL-2RA | rs12789243 | 11 | T | C | 0.1263 | 0.7571 | 0.0276 | 4.74E-06 | 20.94 | |
| IL-2RA | rs17624670 | 8 | A | G | -0.125 | 0.2531 | 0.0273 | 4.68E-06 | 20.96 | |
| IL-2RA | rs34037190 | 10 | A | G | 0.4784 | 0.0217 | 0.0935 | 3.11E-07 | 26.18 | |
| IL-2RA | rs34353319 | 10 | A | T | -0.1581 | 0.8176 | 0.0335 | 2.37E-06 | 22.27 | |
| IL-2RA | rs56213152 | 7 | T | C | 0.1269 | 0.7688 | 0.0271 | 2.83E-06 | 21.93 | |
| IL-2RA | rs7078614 | 10 | T | G | -0.1543 | 0.3549 | 0.0241 | 1.53E-10 | 40.99 | |
| IL-2RA | rs79100208 | 3 | C | G | 0.8345 | 0.995 | 0.1758 | 2.07E-06 | 22.53 | |
| IL-2RA | rs9423654 | 10 | C | G | -0.135 | 0.6609 | 0.0283 | 1.84E-06 | 22.76 | |
| IL-4 | rs116705532 | 1 | T | G | -0.4675 | 0.9925 | 0.0978 | 1.75E-06 | 22.85 | |
| IL-4 | rs117146485 | 9 | T | C | -0.2856 | 0.9837 | 0.0625 | 4.89E-06 | 20.88 | |
| IL-4 | rs12238729 | 9 | T | C | 0.5271 | 0.0162 | 0.1096 | 1.51E-06 | 23.13 | |
| IL-4 | rs12640583 | 4 | T | G | -0.1104 | 0.1631 | 0.0214 | 2.48E-07 | 26.61 | |
| IL-4 | rs17713451 | 7 | A | G | 0.1255 | 0.1149 | 0.0252 | 6.35E-07 | 24.80 | |
| IL-4 | rs1867282 | 9 | T | C | 0.0808 | 0.4148 | 0.0162 | 6.11E-07 | 24.88 | |
| IL-4 | rs2073438 | 17 | A | G | 0.0847 | 0.2513 | 0.0183 | 3.68E-06 | 21.42 | |
| IL-4 | rs2346020 | 3 | A | G | 0.079 | 0.6795 | 0.0169 | 2.95E-06 | 21.85 | |
| IL-4 | rs2708586 | 7 | T | C | -0.0767 | 0.3411 | 0.0166 | 3.83E-06 | 21.35 | |
| IL-4 | rs56408830 | 6 | A | G | -0.1794 | 0.0537 | 0.0365 | 8.88E-07 | 24.16 | |
| IL-4 | rs7613691 | 3 | A | G | 0.1787 | 0.9482 | 0.0382 | 2.90E-06 | 21.88 | |
| IL-4 | rs79597994 | 1 | T | C | -0.5855 | 0.0049 | 0.1271 | 4.09E-06 | 21.22 | |
| IL-4 | rs9941733 | 20 | A | G | 0.1156 | 0.8355 | 0.0229 | 4.46E-07 | 25.48 | |
| IL-5 | rs10178043 | 2 | T | G | 0.2579 | 0.949 | 0.0553 | 3.11E-06 | 21.75 | |
| IL-5 | rs111736126 | 2 | C | G | -0.3973 | 0.9792 | 0.0867 | 4.60E-06 | 21.00 | |
| IL-5 | rs148634917 | 1 | A | G | -0.517 | 0.986 | 0.1087 | 1.97E-06 | 22.62 | |
| IL-5 | rs28793375 | 8 | T | C | 0.1697 | 0.1298 | 0.0362 | 2.76E-06 | 21.98 | |
| IL-5 | rs72831687 | 6 | A | G | -0.5337 | 0.0137 | 0.1104 | 1.34E-06 | 23.37 | |
| IL-5 | rs73040118 | 19 | T | C | 0.2294 | 0.9285 | 0.049 | 2.85E-06 | 21.92 | |
| IL-5 | rs74811276 | 14 | A | G | 0.217 | 0.0761 | 0.0471 | 4.08E-06 | 21.23 | |
| IL-5 | rs7739450 | 6 | A | G | -0.1295 | 0.5158 | 0.0256 | 4.22E-07 | 25.59 | |
| IL-5 | rs9309063 | 2 | T | G | -0.1119 | 0.4763 | 0.0245 | 4.94E-06 | 20.86 | |
| IL-6 | rs10910395 | 1 | A | T | -0.108 | 0.8729 | 0.0235 | 4.31E-06 | 21.12 | |
| IL-6 | rs10982193 | 9 | A | G | -0.0793 | 0.2822 | 0.0174 | 5.18E-06 | 20.77 | |
| IL-6 | rs113098456 | 2 | A | G | -0.1553 | 0.0725 | 0.0339 | 4.62E-06 | 20.99 | |
| IL-6 | rs113600793 | 17 | A | C | 0.1736 | 0.0623 | 0.0359 | 1.33E-06 | 23.38 | |
| IL-6 | rs114373846 | 3 | T | C | 0.4196 | 0.0085 | 0.0905 | 3.54E-06 | 21.50 | |
| IL-6 | rs11732981 | 4 | A | C | 0.0722 | 0.5119 | 0.0156 | 3.69E-06 | 21.42 | |
| IL-6 | rs1333040 | 9 | T | C | 0.0747 | 0.4508 | 0.0157 | 1.96E-06 | 22.64 | |
| IL-6 | rs13412535 | 2 | A | G | -0.1186 | 0.189 | 0.0214 | 2.99E-08 | 30.71 | |
| IL-6 | rs4684700 | 3 | T | C | -0.0747 | 0.5142 | 0.0162 | 4.01E-06 | 21.26 | |
| IL-6 | rs73273528 | 20 | T | C | 0.268 | 0.0211 | 0.0553 | 1.26E-06 | 23.49 | |
| IL-6 | rs76856708 | 16 | T | C | 0.336 | 0.9856 | 0.0697 | 1.43E-06 | 23.24 | |
| IL-7 | rs115215018 | 4 | T | C | 0.5985 | 0.0101 | 0.1308 | 4.75E-06 | 20.94 | |
| IL-7 | rs117509142 | 8 | T | C | -0.3213 | 0.9569 | 0.0684 | 2.64E-06 | 22.07 | |
| IL-7 | rs11757972 | 6 | T | C | 0.121 | 0.4776 | 0.0257 | 2.50E-06 | 22.17 | |
| IL-7 | rs1374279 | 2 | A | T | 0.1625 | 0.143 | 0.0347 | 2.83E-06 | 21.93 | |
| IL-7 | rs142397827 | 5 | A | C | 0.4592 | 0.0178 | 0.0994 | 3.84E-06 | 21.34 | |
| IL-7 | rs17091524 | 14 | T | C | 0.5092 | 0.9849 | 0.1015 | 5.26E-07 | 25.17 | |
| IL-7 | rs218238 | 4 | A | T | 0.1319 | 0.7729 | 0.0284 | 3.41E-06 | 21.57 | |
| IL-7 | rs28793375 | 8 | T | C | 0.1644 | 0.1305 | 0.036 | 4.96E-06 | 20.85 | |
| IL-7 | rs62006410 | 14 | T | C | -0.1492 | 0.245 | 0.0302 | 7.80E-07 | 24.41 | |
| IL-7 | rs7155170 | 14 | A | T | -0.1236 | 0.2744 | 0.027 | 4.70E-06 | 20.96 | |
| IL-7 | rs77318030 | 19 | T | C | -0.2966 | 0.9574 | 0.0631 | 2.60E-06 | 22.09 | |
| IL-7 | rs7739450 | 6 | A | G | -0.2907 | 0.5167 | 0.0252 | 8.72E-31 | 133.07 | |
| IL-7 | rs77981494 | 16 | T | C | -0.5201 | 0.984 | 0.1055 | 8.23E-07 | 24.30 | |
| IL-7 | rs78346957 | 10 | A | G | 0.4632 | 0.0161 | 0.1008 | 4.32E-06 | 21.12 | |
| IL-8 | rs113487695 | 7 | A | C | -0.6129 | 0.9914 | 0.1292 | 2.10E-06 | 22.50 | |
| IL-8 | rs116726256 | 2 | T | C | -0.2247 | 0.9343 | 0.0489 | 4.33E-06 | 21.11 | |
| IL-8 | rs12075 | 1 | A | G | 0.1148 | 0.5353 | 0.0235 | 1.03E-06 | 23.86 | |
| IL-8 | rs12912642 | 15 | A | G | 0.1168 | 0.3302 | 0.0251 | 3.27E-06 | 21.65 | |
| IL-8 | rs183628733 | 1 | T | C | 0.6547 | 0.9917 | 0.1417 | 3.83E-06 | 21.35 | |
| IL-8 | rs2673604 | 8 | A | C | -0.118 | 0.6829 | 0.0254 | 3.39E-06 | 21.58 | |
| IL-8 | rs3786107 | 17 | A | G | 0.2463 | 0.9287 | 0.0517 | 1.90E-06 | 22.70 | |
| IL-8 | rs75840288 | 16 | A | C | 0.5125 | 0.9839 | 0.1121 | 4.84E-06 | 20.90 | |
| IL-9 | rs117807175 | 14 | C | G | -0.5225 | 0.0121 | 0.1106 | 2.31E-06 | 22.32 | |
| IL-9 | rs1259728 | 12 | A | G | -0.2381 | 0.0554 | 0.0507 | 2.65E-06 | 22.05 | |
| IL-9 | rs3736858 | 13 | C | G | -0.1351 | 0.7976 | 0.0291 | 3.44E-06 | 21.55 | |
| IL-9 | rs41294750 | 1 | T | C | 0.3442 | 0.0308 | 0.0736 | 2.92E-06 | 21.87 | |
| IL-9 | rs4880409 | 10 | T | C | -0.3552 | 0.9664 | 0.0716 | 7.02E-07 | 24.61 | |
| IL-9 | rs73443903 | 6 | A | C | 0.2162 | 0.0685 | 0.046 | 2.60E-06 | 22.09 | |
| IP-10 | rs113183470 | 6 | A | T | -0.2414 | 0.946 | 0.0524 | 4.09E-06 | 21.22 | |
| IP-10 | rs113831257 | 4 | A | G | 0.3639 | 0.0417 | 0.0641 | 1.37E-08 | 32.23 | |
| IP-10 | rs12714300 | 2 | A | T | -0.1573 | 0.1378 | 0.0338 | 3.26E-06 | 21.66 | |
| IP-10 | rs143799975 | 4 | A | G | -0.7551 | 0.9949 | 0.1638 | 4.03E-06 | 21.25 | |
| IP-10 | rs34383175 | 8 | T | C | -0.3196 | 0.035 | 0.0653 | 9.86E-07 | 23.95 | |
| IP-10 | rs397816 | 22 | T | C | 0.1211 | 0.5891 | 0.0248 | 1.04E-06 | 23.84 | |
| IP-10 | rs4859940 | 4 | C | G | -0.1204 | 0.295 | 0.0258 | 3.06E-06 | 21.78 | |
| IP-10 | rs4862110 | 4 | T | C | -0.1453 | 0.771 | 0.0318 | 4.90E-06 | 20.88 | |
| IP-10 | rs75970138 | 9 | A | G | -0.4845 | 0.0139 | 0.1037 | 2.98E-06 | 21.83 | |
| IP-10 | rs7645625 | 3 | T | G | -0.1116 | 0.5724 | 0.0236 | 2.26E-06 | 22.36 | |
| IP-10 | rs79848609 | 15 | A | C | 0.2514 | 0.9449 | 0.0535 | 2.61E-06 | 22.08 | |
| IP-10 | rs8112618 | 19 | A | G | 0.1388 | 0.1841 | 0.0297 | 2.96E-06 | 21.84 | |
| M-CSF | rs116274860 | 3 | T | G | 0.8262 | 0.9908 | 0.1739 | 2.02E-06 | 22.57 | |
| M-CSF | rs116887628 | 8 | A | G | -0.2741 | 0.0611 | 0.0598 | 4.57E-06 | 21.01 | |
| M-CSF | rs117867915 | 18 | T | C | 0.5224 | 0.9793 | 0.1096 | 1.88E-06 | 22.72 | |
| M-CSF | rs11963606 | 6 | C | G | -0.5353 | 0.9826 | 0.117 | 4.76E-06 | 20.93 | |
| M-CSF | rs12962919 | 18 | T | C | 0.3025 | 0.0644 | 0.0659 | 4.43E-06 | 21.07 | |
| M-CSF | rs139457375 | 8 | A | C | -0.4047 | 0.9668 | 0.0854 | 2.15E-06 | 22.46 | |
| M-CSF | rs147378920 | 1 | A | G | -0.6064 | 0.9871 | 0.1318 | 4.21E-06 | 21.17 | |
| M-CSF | rs34089869 | 2 | T | C | 0.2194 | 0.1039 | 0.0462 | 2.05E-06 | 22.55 | |
| M-CSF | rs62294910 | 3 | A | G | 0.3472 | 0.0504 | 0.0687 | 4.33E-07 | 25.54 | |
| M-CSF | rs72723242 | 5 | T | G | -0.4969 | 0.0177 | 0.1083 | 4.47E-06 | 21.05 | |
| M-CSF | rs9387100 | 6 | T | C | -0.135 | 0.4291 | 0.029 | 3.24E-06 | 21.67 | |
| M-CSF | rs9626985 | 22 | T | C | 0.2277 | 0.0936 | 0.0496 | 4.42E-06 | 21.07 | |
| MCP-1-MCAF | rs111995966 | 2 | T | G | 0.1428 | 0.9297 | 0.0309 | 3.81E-06 | 21.36 | |
| MCP-1-MCAF | rs112313229 | 3 | A | G | -0.1652 | 0.0708 | 0.0312 | 1.19E-07 | 28.04 | |
| MCP-1-MCAF | rs11920996 | 3 | T | C | 0.1805 | 0.051 | 0.0376 | 1.58E-06 | 23.05 | |
| MCP-1-MCAF | rs12062235 | 1 | T | G | 0.1477 | 0.9377 | 0.032 | 3.92E-06 | 21.30 | |
| MCP-1-MCAF | rs12493953 | 3 | A | G | -0.0948 | 0.3224 | 0.0172 | 3.56E-08 | 30.38 | |
| MCP-1-MCAF | rs143815843 | 2 | A | G | -0.2049 | 0.0315 | 0.0447 | 4.56E-06 | 21.01 | |
| MCP-1-MCAF | rs145155829 | 1 | T | C | -0.2125 | 0.037 | 0.0461 | 4.04E-06 | 21.25 | |
| MCP-1-MCAF | rs16837903 | 1 | A | G | -0.1104 | 0.1237 | 0.0238 | 3.51E-06 | 21.52 | |
| MCP-1-MCAF | rs188998783 | 19 | T | C | -0.5659 | 0.0066 | 0.1233 | 4.44E-06 | 21.06 | |
| MCP-1-MCAF | rs2229593 | 3 | T | C | 0.2624 | 0.0385 | 0.0405 | 9.23E-11 | 41.98 | |
| MCP-1-MCAF | rs2820126 | 1 | T | G | 0.0907 | 0.7898 | 0.0193 | 2.61E-06 | 22.09 | |
| MCP-1-MCAF | rs3026968 | 1 | T | C | -0.0896 | 0.3195 | 0.0169 | 1.15E-07 | 28.11 | |
| MCP-1-MCAF | rs34190208 | 3 | T | C | 0.1052 | 0.1511 | 0.0219 | 1.56E-06 | 23.08 | |
| MCP-1-MCAF | rs56212190 | 1 | T | C | 0.1799 | 0.0519 | 0.0372 | 1.32E-06 | 23.39 | |
| MCP-1-MCAF | rs7197349 | 16 | A | G | 0.0971 | 0.8124 | 0.0206 | 2.43E-06 | 22.22 | |
| MCP-1-MCAF | rs72705803 | 9 | A | G | -0.2188 | 0.9694 | 0.047 | 3.23E-06 | 21.67 | |
| MCP-1-MCAF | rs77116118 | 3 | T | C | -0.4239 | 0.9892 | 0.0824 | 2.68E-07 | 26.47 | |
| MCP-1-MCAF | rs7978037 | 12 | A | T | 0.0746 | 0.3863 | 0.016 | 3.12E-06 | 21.74 | |
| MCP-1-MCAF | rs79939301 | 3 | A | G | 0.1449 | 0.1081 | 0.0255 | 1.33E-08 | 32.29 | |
| MCP-1-MCAF | rs80108502 | 3 | T | C | 0.2463 | 0.0391 | 0.0409 | 1.72E-09 | 36.26 | |
| MCP-1-MCAF | rs863002 | 1 | T | C | 0.1135 | 0.4077 | 0.0158 | 6.79E-13 | 51.60 | |
| MCP-1-MCAF | rs9317045 | 13 | A | C | 0.1157 | 0.8618 | 0.0235 | 8.51E-07 | 24.24 | |
| MCP-3 | rs10892381 | 11 | T | C | 0.2432 | 0.6629 | 0.0473 | 2.72E-07 | 26.44 | |
| MCP-3 | rs117286643 | 8 | A | G | 0.6934 | 0.0225 | 0.1474 | 2.55E-06 | 22.13 | |
| MCP-3 | rs28394764 | 4 | A | T | 0.597 | 0.9696 | 0.1282 | 3.21E-06 | 21.69 | |
| MCP-3 | rs3129806 | 9 | T | C | -0.1975 | 0.5729 | 0.0433 | 5.09E-06 | 20.80 | |
| MCP-3 | rs6993671 | 8 | T | C | 0.2041 | 0.5588 | 0.0443 | 4.08E-06 | 21.23 | |
| MCP-3 | rs7275485 | 21 | T | C | -0.2218 | 0.2711 | 0.0481 | 4.00E-06 | 21.26 | |
| MIF | rs1007888 | 22 | T | C | -0.1275 | 0.6128 | 0.0245 | 1.95E-07 | 27.08 | |
| MIF | rs113218956 | 22 | A | G | -0.8789 | 0.0044 | 0.1876 | 2.80E-06 | 21.95 | |
| MIF | rs11551183 | 16 | C | G | 0.3666 | 0.9756 | 0.0795 | 4.00E-06 | 21.26 | |
| MIF | rs12594190 | 15 | A | G | 0.1321 | 0.7008 | 0.0266 | 6.83E-07 | 24.66 | |
| MIF | rs141009259 | 2 | T | C | -0.6194 | 0.9886 | 0.1285 | 1.43E-06 | 23.23 | |
| MIF | rs2294689 | 6 | C | G | -0.1338 | 0.3018 | 0.0287 | 3.13E-06 | 21.73 | |
| MIF | rs35792361 | 4 | A | G | -0.2586 | 0.0591 | 0.0527 | 9.25E-07 | 24.08 | |
| MIF | rs35890933 | 19 | T | G | 0.1676 | 0.8474 | 0.0365 | 4.39E-06 | 21.08 | |
| MIF | rs3814097 | 7 | A | G | -0.1163 | 0.5533 | 0.0251 | 3.60E-06 | 21.47 | |
| MIF | rs78098071 | 5 | T | C | -0.4583 | 0.9809 | 0.0915 | 5.48E-07 | 25.09 | |
| MIG | rs10266753 | 7 | T | C | -0.2016 | 0.9109 | 0.0397 | 3.81E-07 | 25.79 | |
| MIG | rs111607343 | 19 | A | G | -0.5235 | 0.0134 | 0.1119 | 2.89E-06 | 21.89 | |
| MIG | rs11177248 | 12 | A | G | 0.3157 | 0.0351 | 0.0667 | 2.21E-06 | 22.40 | |
| MIG | rs113302091 | 14 | T | C | 0.2537 | 0.0468 | 0.0553 | 4.48E-06 | 21.05 | |
| MIG | rs13143163 | 4 | C | G | 0.2735 | 0.0512 | 0.0582 | 2.61E-06 | 22.08 | |
| MIG | rs139010077 | 3 | T | C | 0.4337 | 0.0169 | 0.0943 | 4.24E-06 | 21.15 | |
| MIG | rs191555775 | 6 | A | T | 0.2279 | 0.909 | 0.0412 | 3.17E-08 | 30.60 | |
| MIG | rs192433162 | 10 | A | G | -0.8045 | 0.0061 | 0.1676 | 1.59E-06 | 23.04 | |
| MIG | rs3733233 | 4 | T | C | 0.1223 | 0.6588 | 0.025 | 9.98E-07 | 23.93 | |
| MIG | rs62562991 | 9 | A | G | 0.6239 | 0.0097 | 0.1259 | 7.21E-07 | 24.56 | |
| MIG | rs6679677 | 1 | A | C | 0.1628 | 0.1458 | 0.0327 | 6.40E-07 | 24.79 | |
| MIG | rs8127917 | 21 | T | G | 0.2382 | 0.0608 | 0.0492 | 1.29E-06 | 23.44 | |
| MIG | rs816960 | 13 | T | C | -0.1179 | 0.3692 | 0.0242 | 1.11E-06 | 23.74 | |
| MIG | rs9456663 | 6 | T | C | -0.1186 | 0.6702 | 0.0255 | 3.30E-06 | 21.63 | |
| MIP-1A | rs116615337 | 1 | A | G | 0.1286 | 0.4232 | 0.0278 | 3.73E-06 | 21.40 | |
| MIP-1A | rs117506943 | 11 | T | C | 0.3128 | 0.0323 | 0.0682 | 4.51E-06 | 21.04 | |
| MIP-1A | rs12159394 | 22 | A | G | -0.1708 | 0.1183 | 0.0366 | 3.06E-06 | 21.78 | |
| MIP-1A | rs57786342 | 14 | A | G | 0.139 | 0.2336 | 0.0283 | 9.03E-07 | 24.12 | |
| MIP-1A | rs6900267 | 6 | A | C | -0.2472 | 0.9308 | 0.0515 | 1.59E-06 | 23.04 | |
| MIP-1A | rs6956239 | 7 | T | C | 0.119 | 0.2927 | 0.026 | 4.72E-06 | 20.95 | |
| MIP-1B | rs111942332 | 17 | T | G | -0.4711 | 0.9782 | 0.0571 | 1.58E-16 | 68.07 | |
| MIP-1B | rs112337896 | 17 | A | G | 0.2891 | 0.0288 | 0.063 | 4.46E-06 | 21.06 | |
| MIP-1B | rs114933663 | 3 | T | C | 0.3178 | 0.0694 | 0.0307 | 4.11E-25 | 107.16 | |
| MIP-1B | rs116237296 | 1 | A | G | 0.5284 | 0.0054 | 0.1115 | 2.15E-06 | 22.46 | |
| MIP-1B | rs117010890 | 17 | T | C | -0.2043 | 0.9347 | 0.0393 | 2.01E-07 | 27.02 | |
| MIP-1B | rs117084209 | 17 | C | G | 0.2445 | 0.0365 | 0.0428 | 1.11E-08 | 32.63 | |
| MIP-1B | rs117139712 | 17 | T | C | -0.2669 | 0.0277 | 0.05 | 9.40E-08 | 28.49 | |
| MIP-1B | rs11716293 | 3 | C | G | 0.0986 | 0.7698 | 0.0189 | 1.82E-07 | 27.22 | |
| MIP-1B | rs117394484 | 17 | T | C | -0.4266 | 0.0126 | 0.0786 | 5.72E-08 | 29.46 | |
| MIP-1B | rs117453826 | 17 | A | G | -0.5907 | 0.981 | 0.0591 | 1.60E-23 | 99.90 | |
| MIP-1B | rs117503347 | 17 | T | C | 0.3039 | 0.98 | 0.062 | 9.51E-07 | 24.03 | |
| MIP-1B | rs117657747 | 18 | A | G | 0.2089 | 0.0526 | 0.0453 | 4.00E-06 | 21.27 | |
| MIP-1B | rs12452320 | 17 | A | C | 0.2139 | 0.0716 | 0.0319 | 2.01E-11 | 44.96 | |
| MIP-1B | rs12951603 | 17 | A | G | -0.1132 | 0.1492 | 0.0225 | 4.88E-07 | 25.31 | |
| MIP-1B | rs141793738 | 17 | A | G | 0.1815 | 0.0432 | 0.0389 | 3.07E-06 | 21.77 | |
| MIP-1B | rs1437220 | 17 | T | C | 0.1437 | 0.9207 | 0.0315 | 5.07E-06 | 20.81 | |
| MIP-1B | rs145526037 | 3 | T | G | -0.1863 | 0.9604 | 0.0406 | 4.46E-06 | 21.06 | |
| MIP-1B | rs146565944 | 17 | T | C | 0.2863 | 0.0235 | 0.0558 | 2.88E-07 | 26.33 | |
| MIP-1B | rs148561432 | 17 | A | G | -0.2691 | 0.0454 | 0.0407 | 3.80E-11 | 43.72 | |
| MIP-1B | rs1543292 | 17 | A | G | 0.2627 | 0.0632 | 0.0338 | 7.71E-15 | 60.41 | |
| MIP-1B | rs159309 | 17 | T | C | 0.1254 | 0.1102 | 0.0258 | 1.17E-06 | 23.62 | |
| MIP-1B | rs17138331 | 7 | A | G | -0.1434 | 0.9092 | 0.0295 | 1.17E-06 | 23.63 | |
| MIP-1B | rs1867288 | 17 | C | G | 0.2034 | 0.2288 | 0.0215 | 3.07E-21 | 89.50 | |
| MIP-1B | rs191600590 | 3 | A | T | 0.1449 | 0.1326 | 0.0246 | 3.86E-09 | 34.69 | |
| MIP-1B | rs2131092 | 3 | A | G | -0.1278 | 0.1103 | 0.0248 | 2.56E-07 | 26.56 | |
| MIP-1B | rs2276857 | 3 | T | C | -0.1283 | 0.1356 | 0.0257 | 5.97E-07 | 24.92 | |
| MIP-1B | rs2314809 | 17 | T | C | -0.0735 | 0.5305 | 0.0157 | 2.85E-06 | 21.92 | |
| MIP-1B | rs2373048 | 3 | A | T | -0.116 | 0.1789 | 0.0215 | 6.84E-08 | 29.11 | |
| MIP-1B | rs2376263 | 17 | A | G | 0.1053 | 0.7795 | 0.0187 | 1.79E-08 | 31.71 | |
| MIP-1B | rs2411161 | 17 | T | C | 0.1719 | 0.9508 | 0.0365 | 2.48E-06 | 22.18 | |
| MIP-1B | rs2673059 | 3 | T | C | 0.0921 | 0.7787 | 0.0192 | 1.61E-06 | 23.01 | |
| MIP-1B | rs281728 | 8 | A | C | -0.079 | 0.7058 | 0.0171 | 3.84E-06 | 21.34 | |
| MIP-1B | rs28393318 | 4 | A | G | -0.1076 | 0.8758 | 0.0235 | 4.68E-06 | 20.96 | |
| MIP-1B | rs28856610 | 17 | T | C | -0.3074 | 0.031 | 0.0495 | 5.30E-10 | 38.57 | |
| MIP-1B | rs323877 | 3 | C | G | -0.0965 | 0.6443 | 0.0172 | 2.02E-08 | 31.48 | |
| MIP-1B | rs34437725 | 17 | T | C | -0.2571 | 0.9697 | 0.0481 | 9.04E-08 | 28.57 | |
| MIP-1B | rs35933743 | 17 | T | G | -0.1183 | 0.8438 | 0.0238 | 6.68E-07 | 24.71 | |
| MIP-1B | rs41290648 | 3 | A | G | 0.2231 | 0.1266 | 0.0239 | 1.01E-20 | 87.14 | |
| MIP-1B | rs41341749 | 17 | A | G | -0.1656 | 0.9203 | 0.0294 | 1.77E-08 | 31.73 | |
| MIP-1B | rs41502550 | 3 | T | C | 0.1266 | 0.8499 | 0.022 | 8.69E-09 | 33.11 | |
| MIP-1B | rs4795931 | 17 | A | G | -0.0865 | 0.3193 | 0.0173 | 5.73E-07 | 25.00 | |
| MIP-1B | rs4796110 | 17 | A | G | 0.1244 | 0.8879 | 0.0256 | 1.18E-06 | 23.61 | |
| MIP-1B | rs56083628 | 17 | T | C | -0.1297 | 0.1054 | 0.0257 | 4.50E-07 | 25.47 | |
| MIP-1B | rs62079535 | 17 | A | G | 0.2309 | 0.9572 | 0.0389 | 2.93E-09 | 35.23 | |
| MIP-1B | rs6908843 | 6 | A | G | 0.0997 | 0.1684 | 0.0209 | 1.84E-06 | 22.76 | |
| MIP-1B | rs71381491 | 17 | A | C | 0.3777 | 0.9746 | 0.0561 | 1.67E-11 | 45.33 | |
| MIP-1B | rs72791296 | 5 | T | C | 0.2364 | 0.0349 | 0.0466 | 3.92E-07 | 25.73 | |
| MIP-1B | rs72799710 | 5 | T | C | -0.1037 | 0.1514 | 0.0217 | 1.76E-06 | 22.84 | |
| MIP-1B | rs72820112 | 17 | T | C | 0.1059 | 0.7412 | 0.0183 | 7.17E-09 | 33.49 | |
| MIP-1B | rs72820246 | 17 | T | G | -0.0983 | 0.4163 | 0.0167 | 3.95E-09 | 34.65 | |
| MIP-1B | rs72825991 | 17 | A | G | -0.179 | 0.0709 | 0.0312 | 9.63E-09 | 32.92 | |
| MIP-1B | rs72829264 | 17 | A | G | -0.1517 | 0.8833 | 0.0277 | 4.34E-08 | 29.99 | |
| MIP-1B | rs74979864 | 7 | A | T | -0.3184 | 0.0169 | 0.0613 | 2.06E-07 | 26.98 | |
| MIP-1B | rs76582507 | 9 | A | G | 0.3259 | 0.0156 | 0.0676 | 1.43E-06 | 23.24 | |
| MIP-1B | rs76842834 | 17 | T | C | -0.4207 | 0.0371 | 0.0471 | 4.18E-19 | 79.78 | |
| MIP-1B | rs76863419 | 17 | T | G | -0.2833 | 0.073 | 0.0341 | 9.74E-17 | 69.02 | |
| MIP-1B | rs79088462 | 17 | T | C | -0.3214 | 0.9782 | 0.0584 | 3.72E-08 | 30.29 | |
| MIP-1B | rs80322601 | 17 | T | C | -0.1915 | 0.9468 | 0.038 | 4.67E-07 | 25.40 | |
| MIP-1B | rs8081726 | 17 | T | C | -0.3499 | 0.0491 | 0.036 | 2.49E-22 | 94.47 | |
| MIP-1B | rs854222 | 3 | A | C | 0.0809 | 0.2696 | 0.0177 | 4.86E-06 | 20.89 | |
| MIP-1B | rs873944 | 17 | T | C | 0.2392 | 0.0726 | 0.0322 | 1.10E-13 | 55.18 | |
| MIP-1B | rs939408 | 3 | A | C | -0.1007 | 0.3938 | 0.016 | 3.10E-10 | 39.61 | |
| MIP-1B | rs951814 | 17 | A | G | 0.1614 | 0.07 | 0.0315 | 2.99E-07 | 26.25 | |
| MIP-1B | rs9793308 | 1 | A | G | 0.0842 | 0.6036 | 0.0177 | 1.96E-06 | 22.63 | |
| MIP-1B | rs9838883 | 3 | T | C | 0.079 | 0.3399 | 0.0166 | 1.95E-06 | 22.65 | |
| MIP-1B | rs9911839 | 17 | T | G | -0.1377 | 0.9011 | 0.027 | 3.40E-07 | 26.01 | |
| MIP-1B | rs9914803 | 17 | T | C | 0.098 | 0.5189 | 0.0156 | 3.34E-10 | 39.46 | |
| PDGF-BB | rs10512952 | 5 | T | C | -0.2816 | 0.9819 | 0.0587 | 1.61E-06 | 23.01 | |
| PDGF-BB | rs111996132 | 2 | A | C | -0.3127 | 0.9789 | 0.0561 | 2.49E-08 | 31.07 | |
| PDGF-BB | rs116154010 | 2 | T | C | 0.3225 | 0.0179 | 0.0662 | 1.11E-06 | 23.73 | |
| PDGF-BB | rs11766649 | 7 | A | G | 0.0902 | 0.8018 | 0.0196 | 4.18E-06 | 21.18 | |
| PDGF-BB | rs12289510 | 11 | A | G | -0.0772 | 0.4767 | 0.0158 | 1.03E-06 | 23.87 | |
| PDGF-BB | rs12615784 | 2 | T | C | -0.1003 | 0.7849 | 0.0193 | 2.03E-07 | 27.01 | |
| PDGF-BB | rs13037046 | 20 | A | T | -0.0948 | 0.1871 | 0.0206 | 4.19E-06 | 21.18 | |
| PDGF-BB | rs147862316 | 2 | T | C | 0.2279 | 0.0371 | 0.0411 | 2.94E-08 | 30.75 | |
| PDGF-BB | rs192743385 | 15 | T | C | 0.1807 | 0.0518 | 0.0392 | 4.03E-06 | 21.25 | |
| PDGF-BB | rs2643354 | 15 | A | G | 0.1251 | 0.8921 | 0.0261 | 1.64E-06 | 22.97 | |
| PDGF-BB | rs34131731 | 2 | A | T | 0.2083 | 0.9594 | 0.0411 | 4.02E-07 | 25.69 | |
| PDGF-BB | rs35859699 | 4 | A | G | -0.3854 | 0.0109 | 0.0838 | 4.24E-06 | 21.15 | |
| PDGF-BB | rs62191444 | 20 | T | G | -0.112 | 0.1492 | 0.0239 | 2.78E-06 | 21.96 | |
| PDGF-BB | rs6910518 | 6 | T | G | 0.0806 | 0.5924 | 0.0162 | 6.51E-07 | 24.75 | |
| PDGF-BB | rs7170232 | 15 | T | C | 0.1609 | 0.219 | 0.0189 | 1.69E-17 | 72.48 | |
| PDGF-BB | rs72958564 | 2 | A | T | -0.4097 | 0.9901 | 0.0864 | 2.12E-06 | 22.49 | |
| PDGF-BB | rs72972467 | 2 | C | G | -0.1616 | 0.0623 | 0.0328 | 8.36E-07 | 24.27 | |
| PDGF-BB | rs73162807 | 3 | A | C | -0.2313 | 0.0259 | 0.0499 | 3.56E-06 | 21.49 | |
| PDGF-BB | rs9924851 | 16 | C | G | 0.0767 | 0.3564 | 0.0163 | 2.53E-06 | 22.14 | |
| RANTES | rs10505135 | 8 | T | C | 0.1315 | 0.3704 | 0.0252 | 1.81E-07 | 27.23 | |
| RANTES | rs118096511 | 13 | T | C | -0.3374 | 0.9696 | 0.0709 | 1.95E-06 | 22.65 | |
| RANTES | rs11873385 | 18 | A | G | -0.2582 | 0.9466 | 0.0552 | 2.90E-06 | 21.88 | |
| RANTES | rs148526102 | 19 | T | C | -0.3798 | 0.0215 | 0.083 | 4.74E-06 | 20.94 | |
| RANTES | rs2731672 | 5 | T | C | -0.1242 | 0.267 | 0.0272 | 4.97E-06 | 20.85 | |
| RANTES | rs4795087 | 17 | C | G | 0.1494 | 0.8188 | 0.0312 | 1.68E-06 | 22.93 | |
| RANTES | rs62438851 | 6 | A | G | -0.1904 | 0.8961 | 0.0413 | 4.02E-06 | 21.25 | |
| RANTES | rs7170339 | 15 | C | G | -0.4283 | 0.0197 | 0.0904 | 2.16E-06 | 22.45 | |
| RANTES | rs72793342 | 16 | A | G | -0.1505 | 0.2003 | 0.0307 | 9.47E-07 | 24.03 | |
| RANTES | rs78050316 | 2 | A | C | 0.4202 | 0.0204 | 0.0859 | 1.00E-06 | 23.93 | |
| SCF | rs10800449 | 1 | A | C | 0.0851 | 0.2859 | 0.0179 | 1.99E-06 | 22.60 | |
| SCF | rs11244035 | 9 | T | C | -0.1296 | 0.0944 | 0.0279 | 3.40E-06 | 21.58 | |
| SCF | rs113127926 | 14 | A | C | 0.1974 | 0.0418 | 0.0418 | 2.33E-06 | 22.30 | |
| SCF | rs117721699 | 9 | C | G | -0.2392 | 0.0265 | 0.0484 | 7.73E-07 | 24.42 | |
| SCF | rs12345108 | 9 | T | C | -0.0772 | 0.3395 | 0.0167 | 3.79E-06 | 21.37 | |
| SCF | rs13412535 | 2 | A | G | -0.1065 | 0.1892 | 0.0213 | 5.73E-07 | 25.00 | |
| SCF | rs138538809 | 8 | T | C | -0.5788 | 0.0052 | 0.1139 | 3.74E-07 | 25.82 | |
| SCF | rs1536480 | 9 | T | C | 0.081 | 0.3204 | 0.0167 | 1.23E-06 | 23.53 | |
| SCF | rs72678285 | 14 | A | T | 0.1062 | 0.8512 | 0.0231 | 4.28E-06 | 21.14 | |
| SCF | rs72832071 | 16 | A | G | 0.2238 | 0.9732 | 0.0482 | 3.43E-06 | 21.56 | |
| SCF | rs78666213 | 4 | T | G | -0.2845 | 0.9797 | 0.0574 | 7.18E-07 | 24.57 | |
| SCF | rs8045376 | 16 | A | G | -0.3126 | 0.0144 | 0.068 | 4.28E-06 | 21.13 | |
| SCGF-B | rs112346514 | 19 | T | C | -0.3261 | 0.0302 | 0.0703 | 3.51E-06 | 21.52 | |
| SCGF-B | rs1149926 | 10 | T | C | -0.3458 | 0.0239 | 0.0749 | 3.90E-06 | 21.32 | |
| SCGF-B | rs118003677 | 12 | T | C | -0.3654 | 0.9775 | 0.0786 | 3.34E-06 | 21.61 | |
| SCGF-B | rs12118918 | 1 | A | G | -0.1631 | 0.8669 | 0.035 | 3.16E-06 | 21.72 | |
| SCGF-B | rs12480722 | 20 | T | C | 0.1654 | 0.8752 | 0.0353 | 2.79E-06 | 21.95 | |
| SCGF-B | rs13287050 | 9 | A | T | -0.121 | 0.7202 | 0.0263 | 4.21E-06 | 21.17 | |
| SCGF-B | rs13866 | 19 | T | C | -0.1647 | 0.2622 | 0.028 | 4.05E-09 | 34.60 | |
| SCGF-B | rs139413256 | 7 | A | G | -0.5174 | 0.0139 | 0.1076 | 1.52E-06 | 23.12 | |
| SCGF-B | rs143829871 | 3 | T | C | -0.1866 | 0.9047 | 0.0399 | 2.92E-06 | 21.87 | |
| SCGF-B | rs144724875 | 19 | T | C | 0.5381 | 0.0274 | 0.0829 | 8.53E-11 | 42.13 | |
| SCGF-B | rs149009264 | 10 | A | G | 0.4551 | 0.9848 | 0.0985 | 3.83E-06 | 21.35 | |
| SCGF-B | rs150733161 | 13 | T | C | -0.5255 | 0.0142 | 0.112 | 2.71E-06 | 22.01 | |
| SCGF-B | rs151194174 | 7 | A | G | 0.4536 | 0.0187 | 0.0941 | 1.43E-06 | 23.24 | |
| SCGF-B | rs264157 | 18 | A | G | 0.1079 | 0.4743 | 0.0233 | 3.64E-06 | 21.45 | |
| SCGF-B | rs34911860 | 1 | A | G | -0.3674 | 0.0291 | 0.0787 | 3.04E-06 | 21.79 | |
| SCGF-B | rs3817303 | 12 | T | G | 0.1362 | 0.194 | 0.0294 | 3.61E-06 | 21.46 | |
| SCGF-B | rs4737731 | 8 | T | C | 0.1146 | 0.3019 | 0.0251 | 4.98E-06 | 20.85 | |
| SCGF-B | rs4976691 | 5 | C | G | -0.1484 | 0.3257 | 0.0253 | 4.47E-09 | 34.41 | |
| SCGF-B | rs5742627 | 12 | T | C | 0.2625 | 0.045 | 0.0574 | 4.80E-06 | 20.91 | |
| SCGF-B | rs77247938 | 12 | A | G | 0.2862 | 0.0655 | 0.0475 | 1.69E-09 | 36.30 | |
| SCGF-B | rs77954165 | 9 | T | C | 0.2631 | 0.9528 | 0.0562 | 2.85E-06 | 21.92 | |
| SCGF-B | rs7802293 | 7 | T | C | -0.1145 | 0.3779 | 0.0247 | 3.56E-06 | 21.49 | |
| SCGF-B | rs78217154 | 8 | T | C | 0.3942 | 0.9796 | 0.0861 | 4.69E-06 | 20.96 | |
| SDF-1A | rs10474392 | 5 | A | G | 0.0934 | 0.334 | 0.0177 | 1.31E-07 | 27.84 | |
| SDF-1A | rs10516368 | 4 | A | C | -0.4268 | 0.0105 | 0.0883 | 1.34E-06 | 23.36 | |
| SDF-1A | rs12141941 | 1 | T | C | -0.0881 | 0.7493 | 0.0186 | 2.17E-06 | 22.43 | |
| SDF-1A | rs149893336 | 4 | A | G | -0.494 | 0.993 | 0.1082 | 4.98E-06 | 20.84 | |
| SDF-1A | rs1600396 | 4 | A | G | -0.0933 | 0.8096 | 0.0204 | 4.80E-06 | 20.92 | |
| SDF-1A | rs62194946 | 2 | T | G | -0.0849 | 0.2628 | 0.0185 | 4.45E-06 | 21.06 | |
| SDF-1A | rs6586903 | 8 | T | C | -0.1264 | 0.1037 | 0.0268 | 2.40E-06 | 22.24 | |
| SDF-1A | rs76766406 | 4 | A | G | 0.4642 | 0.9935 | 0.1012 | 4.50E-06 | 21.04 | |
| SDF-1A | rs78037609 | 21 | A | G | -0.6261 | 0.0104 | 0.1334 | 2.69E-06 | 22.03 | |
| SDF-1A | rs78883416 | 10 | C | G | -0.0871 | 0.308 | 0.0182 | 1.70E-06 | 22.90 | |
| TNF-A | rs10767536 | 11 | A | G | 0.118 | 0.3274 | 0.0253 | 3.10E-06 | 21.75 | |
| TNF-A | rs115018697 | 4 | C | G | -0.9542 | 0.9962 | 0.197 | 1.27E-06 | 23.46 | |
| TNF-A | rs116736594 | 5 | T | C | 0.3407 | 0.0312 | 0.0702 | 1.21E-06 | 23.55 | |
| TNF-A | rs79105320 | 8 | A | G | 0.5573 | 0.0112 | 0.1177 | 2.19E-06 | 22.42 | |
| TNF-B | rs10925040 | 1 | T | C | 0.1738 | 0.371 | 0.0372 | 2.98E-06 | 21.83 | |
| TNF-B | rs143259067 | 1 | T | C | -0.6923 | 0.9595 | 0.1003 | 5.12E-12 | 47.64 | |
| TNF-B | rs2420873 | 19 | T | G | 0.1673 | 0.3897 | 0.0365 | 4.57E-06 | 21.01 | |
| TNF-B | rs62284710 | 3 | A | G | 0.3702 | 0.9443 | 0.0782 | 2.20E-06 | 22.41 | |
| TNF-B | rs75240021 | 8 | C | G | 0.3713 | 0.071 | 0.0772 | 1.51E-06 | 23.13 | |
| TNF-B | rs76225863 | 1 | A | G | 0.7534 | 0.0293 | 0.1217 | 5.99E-10 | 38.32 | |
| TRAIL | rs10084050 | 18 | A | G | -0.1101 | 0.8647 | 0.023 | 1.69E-06 | 22.91 | |
| TRAIL | rs10164260 | 18 | A | G | 0.1003 | 0.1741 | 0.0211 | 2.00E-06 | 22.60 | |
| TRAIL | rs11081739 | 18 | A | G | 0.1395 | 0.2048 | 0.0202 | 4.99E-12 | 47.69 | |
| TRAIL | rs112821861 | 18 | T | G | -0.8566 | 0.9717 | 0.0494 | 2.34E-67 | 300.68 | |
| TRAIL | rs113057689 | 3 | A | G | -0.2625 | 0.033 | 0.0489 | 7.96E-08 | 28.82 | |
| TRAIL | rs11875481 | 18 | T | C | -0.0969 | 0.835 | 0.0211 | 4.38E-06 | 21.09 | |
| TRAIL | rs13115587 | 4 | A | C | 0.101 | 0.1558 | 0.0217 | 3.25E-06 | 21.66 | |
| TRAIL | rs13278062 | 8 | T | G | 0.08 | 0.5589 | 0.0157 | 3.48E-07 | 25.96 | |
| TRAIL | rs139958028 | 11 | A | G | 0.1803 | 0.0501 | 0.0395 | 5.01E-06 | 20.84 | |
| TRAIL | rs141032096 | 18 | T | G | -0.2125 | 0.962 | 0.0455 | 3.01E-06 | 21.81 | |
| TRAIL | rs146827832 | 3 | T | C | 0.1341 | 0.9038 | 0.0291 | 4.06E-06 | 21.24 | |
| TRAIL | rs148051545 | 19 | T | C | -0.4211 | 0.0114 | 0.0843 | 5.88E-07 | 24.95 | |
| TRAIL | rs17535790 | 3 | A | G | -0.1125 | 0.1498 | 0.0218 | 2.46E-07 | 26.63 | |
| TRAIL | rs183815186 | 18 | A | T | -0.3499 | 0.9803 | 0.0602 | 6.16E-09 | 33.78 | |
| TRAIL | rs550057 | 9 | T | C | -0.0783 | 0.3049 | 0.0169 | 3.60E-06 | 21.47 | |
| TRAIL | rs558572 | 3 | T | C | 0.1351 | 0.0949 | 0.0265 | 3.43E-07 | 25.99 | |
| TRAIL | rs57396456 | 18 | T | C | -0.5641 | 0.9755 | 0.0516 | 8.09E-28 | 119.51 | |
| TRAIL | rs62093482 | 18 | T | C | 0.9827 | 0.0237 | 0.0529 | 4.97E-77 | 345.09 | |
| TRAIL | rs7233927 | 18 | A | G | 0.0905 | 0.6648 | 0.0164 | 3.42E-08 | 30.45 | |
| TRAIL | rs73039026 | 3 | A | C | -0.3098 | 0.9821 | 0.0634 | 1.03E-06 | 23.88 | |
| TRAIL | rs73408359 | 18 | T | C | 0.4153 | 0.049 | 0.0364 | 3.76E-30 | 130.17 | |
| TRAIL | rs74488044 | 18 | A | G | 0.3473 | 0.0583 | 0.0334 | 2.53E-25 | 108.12 | |
| TRAIL | rs747324 | 14 | T | C | -0.0826 | 0.2828 | 0.0178 | 3.48E-06 | 21.53 | |
| TRAIL | rs74778900 | 18 | T | C | 0.5791 | 0.0238 | 0.0531 | 1.08E-27 | 118.94 | |
| TRAIL | rs75473890 | 18 | T | C | -0.1349 | 0.9136 | 0.028 | 1.45E-06 | 23.21 | |
| TRAIL | rs75489499 | 3 | T | C | -0.2006 | 0.0677 | 0.0347 | 7.43E-09 | 33.42 | |
| TRAIL | rs75928541 | 4 | A | G | 0.2784 | 0.0188 | 0.0591 | 2.47E-06 | 22.19 | |
| TRAIL | rs7599203 | 2 | T | C | 0.0918 | 0.8034 | 0.02 | 4.43E-06 | 21.07 | |
| TRAIL | rs79085506 | 18 | A | G | 0.6961 | 0.0154 | 0.0737 | 3.55E-21 | 89.21 | |
| TRAIL | rs9946486 | 18 | A | G | 0.1856 | 0.1159 | 0.0245 | 3.58E-14 | 57.39 | |
| VEGF | rs10411345 | 19 | C | G | -0.1041 | 0.7823 | 0.0218 | 1.80E-06 | 22.80 | |
| VEGF | rs10757514 | 9 | C | G | -0.1024 | 0.1715 | 0.0222 | 3.98E-06 | 21.28 | |
| VEGF | rs10822118 | 10 | T | C | -0.0797 | 0.4984 | 0.0168 | 2.09E-06 | 22.51 | |
| VEGF | rs10934631 | 3 | T | C | -0.1132 | 0.8517 | 0.0244 | 3.50E-06 | 21.52 | |
| VEGF | rs10967183 | 9 | T | C | -0.0887 | 0.4262 | 0.0169 | 1.53E-07 | 27.55 | |
| VEGF | rs111950052 | 6 | A | G | -0.1763 | 0.0577 | 0.0383 | 4.16E-06 | 21.19 | |
| VEGF | rs114773511 | 6 | T | C | 0.2187 | 0.0463 | 0.0441 | 7.08E-07 | 24.59 | |
| VEGF | rs12156533 | 9 | A | T | 0.0915 | 0.6655 | 0.0186 | 8.68E-07 | 24.20 | |
| VEGF | rs12456390 | 18 | T | C | -0.0818 | 0.6772 | 0.0179 | 4.88E-06 | 20.88 | |
| VEGF | rs13190738 | 6 | T | C | 0.1111 | 0.3644 | 0.0231 | 1.51E-06 | 23.13 | |
| VEGF | rs1730969 | 16 | C | G | -0.7811 | 0.0039 | 0.1696 | 4.11E-06 | 21.21 | |
| VEGF | rs3025020 | 6 | T | C | -0.124 | 0.2786 | 0.0253 | 9.53E-07 | 24.02 | |
| VEGF | rs4573079 | 6 | A | C | 0.1522 | 0.8439 | 0.0256 | 2.76E-09 | 35.35 | |
| VEGF | rs56071907 | 16 | T | C | 0.126 | 0.1319 | 0.027 | 3.06E-06 | 21.78 | |
| VEGF | rs58078557 | 9 | A | T | -0.1168 | 0.1473 | 0.024 | 1.13E-06 | 23.68 | |
| VEGF | rs60013354 | 10 | A | G | -0.2497 | 0.0266 | 0.0521 | 1.65E-06 | 22.97 | |
| VEGF | rs60987108 | 6 | A | G | 0.1812 | 0.9459 | 0.039 | 3.38E-06 | 21.59 | |
| VEGF | rs6496613 | 15 | A | C | -0.2359 | 0.9677 | 0.0515 | 4.64E-06 | 20.98 | |
| VEGF | rs73872715 | 3 | T | C | -0.6079 | 0.0045 | 0.1299 | 2.87E-06 | 21.90 | |
| VEGF | rs748227 | 6 | T | C | 0.2377 | 0.0654 | 0.0364 | 6.57E-11 | | 42.64 |
| VEGF | rs7739450 | 6 | A | G | -0.415 | 0.5059 | 0.018 | 1.29E-117 | | 531.56 |
| VEGF | rs7754905 | 6 | A | G | -0.1303 | 0.7682 | 0.0204 | 1.69E-10 | 40.80 | |
| VEGF | rs7757024 | 6 | T | C | 0.1443 | 0.0973 | 0.0291 | 7.09E-07 | 24.59 | |
| VEGF | rs77961527 | 3 | A | G | 0.2289 | 0.9606 | 0.0457 | 5.48E-07 | 25.09 | |
| VEGF | rs9369440 | 6 | T | C | -0.0872 | 0.2934 | 0.0186 | 2.76E-06 | 21.98 | |
| VEGF | rs9381249 | 6 | T | C | -0.2414 | 0.9461 | 0.0396 | 1.09E-09 | 37.16 | |
| VEGF | rs9472153 | 6 | A | G | 0.106 | 0.5187 | 0.0174 | 1.12E-09 | 37.11 | |

4.Instrumental variables for inflammatory factors and OP

| **Exposure** | **SNP** | **Chr** | **EA** | **OA** | **Beta** | **EAF** | **SE** | **P** | **F** | |
| --- | --- | --- | --- | --- | --- | --- | --- | --- | --- | --- |
| B-NGF | rs28637706 | 19 | T | G | -0.1554 | 0.314 | 0.0261 | 2.62E-09 | 35.45 | |
| B-NGF | rs4767014 | 12 | T | C | -0.1211 | 0.6731 | 0.0264 | 4.49E-06 | 21.04 | |
| B-NGF | rs71641308 | 1 | T | C | 0.1969 | 0.0995 | 0.0429 | 4.44E-06 | 21.07 | |
| B-NGF | rs73472576 | 18 | T | C | -0.1146 | 0.457 | 0.0251 | 4.98E-06 | 20.85 | |
| CTACK | rs10854859 | 22 | A | G | -0.1498 | 0.2464 | 0.0293 | 3.18E-07 | 26.14 | |
| CTACK | rs117932939 | 9 | T | C | 0.1969 | 0.09 | 0.0422 | 3.07E-06 | 21.77 | |
| CTACK | rs2233872 | 9 | A | G | -0.1495 | 0.7699 | 0.0282 | 1.15E-07 | 28.11 | |
| CTACK | rs57338032 | 15 | A | G | 0.1443 | 0.8287 | 0.0316 | 4.96E-06 | 20.85 | |
| CTACK | rs60247384 | 3 | T | C | 0.1128 | 0.3512 | 0.0245 | 4.14E-06 | 21.20 | |
| CTACK | rs62578137 | 9 | T | C | -0.1311 | 0.2421 | 0.0286 | 4.56E-06 | 21.01 | |
| EOTAXIN | rs147287945 | 6 | A | G | -0.1512 | 0.0741 | 0.0313 | 1.36E-06 | 23.34 | |
| EOTAXIN | rs1677588 | 1 | T | G | 0.1181 | 0.886 | 0.025 | 2.31E-06 | 22.32 | |
| EOTAXIN | rs2024050 | 7 | A | G | 0.164 | 0.0739 | 0.0302 | 5.62E-08 | 29.49 | |
| EOTAXIN | rs2027855 | 22 | T | C | 0.0743 | 0.6153 | 0.0162 | 4.51E-06 | 21.04 | |
| EOTAXIN | rs2040143 | 21 | A | G | -0.0858 | 0.7234 | 0.0178 | 1.43E-06 | 23.23 | |
| EOTAXIN | rs2097947 | 7 | A | T | -0.0769 | 0.6836 | 0.0168 | 4.71E-06 | 20.95 | |
| EOTAXIN | rs2249581 | 1 | T | C | -0.0899 | 0.748 | 0.018 | 5.90E-07 | 24.94 | |
| EOTAXIN | rs2828756 | 21 | T | C | 0.0904 | 0.2423 | 0.0184 | 8.97E-07 | 24.14 | |
| EOTAXIN | rs34004101 | 3 | T | C | -0.1775 | 0.065 | 0.0341 | 1.94E-07 | 27.09 | |
| EOTAXIN | rs4683182 | 3 | A | G | 0.0886 | 0.2201 | 0.0189 | 2.76E-06 | 21.98 | |
| EOTAXIN | rs5754733 | 22 | A | C | -0.105 | 0.8306 | 0.0213 | 8.24E-07 | 24.30 | |
| EOTAXIN | rs57723662 | 17 | C | G | -0.0982 | 0.1657 | 0.0213 | 4.02E-06 | 21.26 | |
| EOTAXIN | rs60075014 | 5 | T | C | -0.1688 | 0.0604 | 0.0356 | 2.12E-06 | 22.48 | |
| EOTAXIN | rs7231030 | 18 | A | C | 0.0903 | 0.7919 | 0.0193 | 2.89E-06 | 21.89 | |
| EOTAXIN | rs73065695 | 3 | A | G | 0.1532 | 0.0932 | 0.0281 | 4.98E-08 | 29.72 | |
| EOTAXIN | rs745331 | 15 | A | G | -0.0821 | 0.6825 | 0.0176 | 3.09E-06 | 21.76 | |
| EOTAXIN | rs75426604 | 14 | A | C | -0.1371 | 0.0876 | 0.0291 | 2.46E-06 | 22.20 | |
| EOTAXIN | rs7550207 | 1 | T | C | -0.0904 | 0.7785 | 0.0187 | 1.34E-06 | 23.37 | |
| EOTAXIN | rs9317045 | 13 | A | C | 0.1172 | 0.8615 | 0.0236 | 6.83E-07 | 24.66 | |
| EOTAXIN | rs9833459 | 3 | T | C | -0.1149 | 0.3033 | 0.0173 | 3.10E-11 | 44.11 | |
| EOTAXIN | rs9975149 | 21 | A | T | 0.0749 | 0.3743 | 0.0164 | 4.95E-06 | 20.86 | |
| FGF-BASIC | rs13412535 | 2 | A | G | -0.1129 | 0.1856 | 0.0224 | 4.65E-07 | 25.40 | |
| FGF-BASIC | rs17094040 | 14 | T | C | 0.1051 | 0.1522 | 0.0229 | 4.44E-06 | 21.06 | |
| FGF-BASIC | rs2849358 | 18 | A | G | 0.0911 | 0.2551 | 0.0193 | 2.36E-06 | 22.28 | |
| FGF-BASIC | rs4795091 | 17 | A | G | 0.1239 | 0.8926 | 0.0266 | 3.19E-06 | 21.70 | |
| FGF-BASIC | rs78873483 | 17 | A | G | 0.1286 | 0.1025 | 0.0282 | 5.11E-06 | 20.80 | |
| G-CSF | rs10939033 | 4 | A | G | -0.0775 | 0.6038 | 0.0163 | 1.99E-06 | 22.61 | |
| G-CSF | rs586802 | 11 | A | G | 0.0882 | 0.758 | 0.0187 | 2.40E-06 | 22.25 | |
| G-CSF | rs6740648 | 2 | T | C | 0.0818 | 0.6752 | 0.0172 | 1.98E-06 | 22.62 | |
| G-CSF | rs76287671 | 19 | T | C | 0.0894 | 0.2333 | 0.0189 | 2.24E-06 | 22.37 | |
| G-CSF | rs77318030 | 19 | T | C | -0.2031 | 0.9563 | 0.0427 | 1.97E-06 | 22.62 | |
| GROA | rs10015342 | 4 | A | T | 0.1945 | 0.1909 | 0.0304 | 1.57E-10 | 40.93 | |
| GROA | rs1361829 | 1 | A | G | -0.1106 | 0.4293 | 0.0241 | 4.45E-06 | 21.06 | |
| GROA | rs17171245 | 7 | T | G | 0.2446 | 0.0536 | 0.053 | 3.93E-06 | 21.30 | |
| GROA | rs3026943 | 1 | A | C | -0.1246 | 0.3311 | 0.0256 | 1.13E-06 | 23.69 | |
| GROA | rs3845622 | 1 | A | C | -0.2345 | 0.1171 | 0.0381 | 7.51E-10 | 37.88 | |
| HGF | rs11060254 | 12 | A | G | -0.0765 | 0.3306 | 0.0166 | 4.06E-06 | 21.24 | |
| HGF | rs11129909 | 3 | T | C | -0.0738 | 0.623 | 0.0161 | 4.56E-06 | 21.01 | |
| HGF | rs13412535 | 2 | A | G | -0.1043 | 0.1892 | 0.0213 | 9.75E-07 | 23.98 | |
| HGF | rs2003620 | 7 | T | C | 0.2277 | 0.027 | 0.0487 | 2.93E-06 | 21.86 | |
| HGF | rs2699434 | 4 | T | C | 0.0863 | 0.3287 | 0.0171 | 4.49E-07 | 25.47 | |
| HGF | rs362307 | 4 | T | C | 0.1511 | 0.0646 | 0.0328 | 4.09E-06 | 21.22 | |
| HGF | rs4245058 | 11 | T | C | -0.1552 | 0.0619 | 0.0331 | 2.75E-06 | 21.99 | |
| HGF | rs5745687 | 7 | T | C | -0.3008 | 0.0375 | 0.0404 | 9.65E-14 | 55.44 | |
| IFN-G | rs113399544 | 7 | A | G | -0.0849 | 0.2768 | 0.0183 | 3.50E-06 | 21.52 | |
| IFN-G | rs113600793 | 17 | A | C | 0.1871 | 0.0631 | 0.0371 | 4.58E-07 | 25.43 | |
| IFN-G | rs117046255 | 7 | T | C | -0.0968 | 0.1922 | 0.0207 | 2.92E-06 | 21.87 | |
| IFN-G | rs1867282 | 9 | T | C | 0.0781 | 0.4154 | 0.0166 | 2.54E-06 | 22.14 | |
| IFN-G | rs2073438 | 17 | A | G | 0.092 | 0.2506 | 0.0188 | 9.90E-07 | 23.95 | |
| IFN-G | rs7088799 | 10 | T | G | -0.0805 | 0.6182 | 0.0166 | 1.24E-06 | 23.52 | |
| IFN-G | rs73479333 | 6 | C | G | -0.1123 | 0.1354 | 0.024 | 2.88E-06 | 21.89 | |
| IL-10 | rs10457128 | 6 | A | G | -0.0854 | 0.639 | 0.0172 | 6.87E-07 | 24.65 | |
| IL-10 | rs10493718 | 1 | A | C | -0.1081 | 0.1603 | 0.0222 | 1.12E-06 | 23.71 | |
| IL-10 | rs13412535 | 2 | A | G | -0.1347 | 0.1856 | 0.0224 | 1.82E-09 | 36.16 | |
| IL-10 | rs1530455 | 3 | T | C | 0.082 | 0.3523 | 0.0174 | 2.45E-06 | 22.21 | |
| IL-10 | rs2086656 | 4 | T | C | -0.08 | 0.6621 | 0.017 | 2.53E-06 | 22.15 | |
| IL-10 | rs3002131 | 1 | C | G | 0.1191 | 0.1195 | 0.026 | 4.63E-06 | 20.98 | |
| IL-10 | rs3025021 | 6 | T | C | 0.0913 | 0.3459 | 0.0194 | 2.52E-06 | 22.15 | |
| IL-10 | rs339203 | 2 | T | C | 0.0954 | 0.8359 | 0.0203 | 2.61E-06 | 22.09 | |
| IL-10 | rs383684 | 6 | A | G | 0.092 | 0.7555 | 0.0197 | 3.01E-06 | 21.81 | |
| IL-10 | rs41282660 | 6 | A | G | -0.1169 | 0.8646 | 0.0254 | 4.18E-06 | 21.18 | |
| IL-10 | rs465757 | 20 | A | G | 0.0806 | 0.6657 | 0.0174 | 3.62E-06 | 21.46 | |
| IL-10 | rs4741748 | 9 | A | G | -0.0788 | 0.6081 | 0.0169 | 3.12E-06 | 21.74 | |
| IL-10 | rs6054847 | 20 | T | C | 0.0971 | 0.8091 | 0.0207 | 2.72E-06 | 22.00 | |
| IL-10 | rs6680918 | 1 | T | C | -0.1202 | 0.8791 | 0.025 | 1.52E-06 | 23.12 | |
| IL-10 | rs7088799 | 10 | T | G | -0.0815 | 0.6191 | 0.0166 | 9.12E-07 | 24.10 | |
| IL-10 | rs73192842 | 3 | A | G | 0.0949 | 0.1911 | 0.0206 | 4.09E-06 | 21.22 | |
| IL-10 | rs7747448 | 6 | A | G | -0.1061 | 0.2594 | 0.0189 | 1.98E-08 | 31.51 | |
| IL-10 | rs865585 | 6 | A | C | -0.119 | 0.8478 | 0.0244 | 1.08E-06 | 23.79 | |
| IL-10 | rs910604 | 6 | A | G | -0.0986 | 0.2383 | 0.0196 | 4.89E-07 | 25.31 | |
| IL-12-P70 | rs113600793 | 17 | A | C | 0.1832 | 0.0623 | 0.0359 | 3.34E-07 | 26.04 | |
| IL-12-P70 | rs12154194 | 6 | T | C | 0.0717 | 0.4615 | 0.0157 | 4.95E-06 | 20.86 | |
| IL-12-P70 | rs12969892 | 18 | T | C | 0.1227 | 0.9031 | 0.0267 | 4.32E-06 | 21.12 | |
| IL-12-P70 | rs13190738 | 6 | T | C | 0.1019 | 0.3609 | 0.019 | 8.18E-08 | 28.76 | |
| IL-12-P70 | rs2123852 | 19 | T | C | 0.0942 | 0.2039 | 0.0204 | 3.88E-06 | 21.32 | |
| IL-12-P70 | rs273702 | 18 | A | G | -0.127 | 0.9015 | 0.027 | 2.55E-06 | 22.12 | |
| IL-12-P70 | rs282258 | 2 | T | C | 0.0726 | 0.4279 | 0.0156 | 3.26E-06 | 21.66 | |
| IL-12-P70 | rs34322762 | 9 | T | C | 0.0953 | 0.648 | 0.0199 | 1.68E-06 | 22.93 | |
| IL-12-P70 | rs34826779 | 8 | T | G | -0.0884 | 0.2157 | 0.019 | 3.28E-06 | 21.65 | |
| IL-12-P70 | rs41282644 | 6 | A | G | 0.1401 | 0.0849 | 0.0303 | 3.77E-06 | 21.38 | |
| IL-12-P70 | rs4714698 | 6 | A | G | -0.1189 | 0.8721 | 0.0238 | 5.86E-07 | 24.96 | |
| IL-12-P70 | rs4741748 | 9 | A | G | -0.0799 | 0.6076 | 0.0163 | 9.49E-07 | 24.03 | |
| IL-12-P70 | rs6458375 | 6 | T | C | 0.0884 | 0.2407 | 0.0191 | 3.69E-06 | 21.42 | |
| IL-12-P70 | rs6532374 | 4 | T | C | -0.1033 | 0.8525 | 0.0226 | 4.86E-06 | 20.89 | |
| IL-12-P70 | rs7763358 | 6 | T | C | 0.1525 | 0.0885 | 0.0274 | 2.61E-08 | 30.98 | |
| IL-12-P70 | rs782111 | 12 | A | C | -0.0765 | 0.5292 | 0.0156 | 9.40E-07 | 24.05 | |
| IL-12-P70 | rs865585 | 6 | A | C | -0.1654 | 0.8484 | 0.0237 | 2.97E-12 | 48.71 | |
| IL-12-P70 | rs9472153 | 6 | A | G | 0.0855 | 0.5141 | 0.0159 | 7.56E-08 | 28.92 | |
| IL-12-P70 | rs9472175 | 6 | T | C | -0.1104 | 0.1542 | 0.0238 | 3.51E-06 | 21.52 | |
| IL-13 | rs10995604 | 10 | A | G | -0.1571 | 0.8587 | 0.0343 | 4.65E-06 | 20.98 | |
| IL-13 | rs12199215 | 6 | T | C | 0.1309 | 0.247 | 0.0284 | 4.04E-06 | 21.24 | |
| IL-13 | rs12623722 | 2 | A | G | -0.1189 | 0.3054 | 0.0257 | 3.72E-06 | 21.40 | |
| IL-13 | rs13206012 | 6 | A | G | -0.3719 | 0.3489 | 0.0263 | 2.13E-45 | 199.96 | |
| IL-13 | rs13209117 | 6 | A | G | 0.1409 | 0.245 | 0.0284 | 7.00E-07 | 24.61 | |
| IL-13 | rs27949 | 5 | T | C | -0.1144 | 0.667 | 0.025 | 4.74E-06 | 20.94 | |
| IL-13 | rs28442067 | 3 | A | G | -0.1379 | 0.7832 | 0.0286 | 1.42E-06 | 23.25 | |
| IL-13 | rs7073807 | 10 | T | C | 0.1618 | 0.1351 | 0.0354 | 4.86E-06 | 20.89 | |
| IL-13 | rs7747448 | 6 | A | G | -0.1393 | 0.2585 | 0.0278 | 5.42E-07 | 25.11 | |
| IL-13 | rs7757246 | 6 | T | C | 0.2147 | 0.9092 | 0.0422 | 3.62E-07 | 25.88 | |
| IL-13 | rs9296421 | 6 | T | G | 0.1814 | 0.842 | 0.0348 | 1.86E-07 | 27.17 | |
| IL-16 | rs117217798 | 17 | T | C | -0.2064 | 0.0889 | 0.044 | 2.72E-06 | 22.00 | |
| IL-16 | rs35834666 | 4 | T | C | -0.1729 | 0.8596 | 0.0348 | 6.75E-07 | 24.68 | |
| IL-16 | rs4976691 | 5 | C | G | 0.1254 | 0.3268 | 0.026 | 1.41E-06 | 23.26 | |
| IL-16 | rs7097884 | 10 | T | C | -0.1193 | 0.5647 | 0.0243 | 9.13E-07 | 24.10 | |
| IL-17 | rs11640734 | 16 | C | G | -0.115 | 0.8673 | 0.024 | 1.65E-06 | 22.96 | |
| IL-17 | rs11985957 | 8 | A | G | 0.1511 | 0.0642 | 0.0329 | 4.38E-06 | 21.09 | |
| IL-17 | rs12735700 | 1 | T | G | -0.0943 | 0.1925 | 0.0206 | 4.70E-06 | 20.96 | |
| IL-17 | rs3804749 | 3 | T | C | -0.0923 | 0.6236 | 0.0167 | 3.26E-08 | 30.55 | |
| IL-17 | rs61990749 | 14 | C | G | 0.1124 | 0.1533 | 0.0226 | 6.58E-07 | 24.74 | |
| IL-17 | rs77341831 | 12 | T | C | 0.2263 | 0.9696 | 0.0473 | 1.72E-06 | 22.89 | |
| IL-17 | rs9568764 | 13 | C | G | 0.0825 | 0.2756 | 0.018 | 4.58E-06 | 21.01 | |
| IL-18 | rs10409850 | 19 | A | G | 0.1791 | 0.8704 | 0.0347 | 2.45E-07 | 26.64 | |
| IL-18 | rs113214367 | 11 | A | G | -0.278 | 0.0565 | 0.0593 | 2.76E-06 | 21.98 | |
| IL-18 | rs117371668 | 16 | T | G | 0.3712 | 0.0254 | 0.0799 | 3.39E-06 | 21.58 | |
| IL-18 | rs12419156 | 11 | T | C | 0.1556 | 0.8064 | 0.0335 | 3.40E-06 | 21.57 | |
| IL-18 | rs1979967 | 15 | T | C | 0.14 | 0.2157 | 0.0285 | 9.00E-07 | 24.13 | |
| IL-18 | rs4952239 | 2 | A | T | -0.1156 | 0.3555 | 0.0242 | 1.78E-06 | 22.82 | |
| IL-18 | rs58701153 | 6 | A | T | -0.1265 | 0.6586 | 0.0242 | 1.72E-07 | 27.32 | |
| IL-18 | rs62312914 | 4 | T | C | -0.1265 | 0.3726 | 0.025 | 4.19E-07 | 25.60 | |
| IL-18 | rs7599125 | 2 | A | G | 0.1109 | 0.569 | 0.0239 | 3.48E-06 | 21.53 | |
| IL-18 | rs764078 | 11 | A | T | 0.1283 | 0.2239 | 0.0278 | 3.93E-06 | 21.30 | |
| IL-1B | rs115242021 | 1 | A | C | 0.2795 | 0.0601 | 0.0553 | 4.32E-07 | 25.55 | |
| IL-1B | rs4786740 | 16 | A | C | 0.1264 | 0.3913 | 0.0265 | 1.84E-06 | 22.75 | |
| IL-1B | rs62015704 | 16 | A | G | 0.1786 | 0.8688 | 0.0372 | 1.58E-06 | 23.05 | |
| IL-1RA | rs1054402 | 9 | T | C | 0.1325 | 0.2506 | 0.0269 | 8.41E-07 | 24.26 | |
| IL-1RA | rs13343438 | 19 | A | G | 0.2771 | 0.0434 | 0.0607 | 4.99E-06 | 20.84 | |
| IL-1RA | rs35590641 | 14 | C | G | -0.1167 | 0.3321 | 0.025 | 3.04E-06 | 21.79 | |
| IL-1RA | rs6699436 | 1 | A | G | -0.1858 | 0.0995 | 0.0404 | 4.25E-06 | 21.15 | |
| IL-1RA | rs9623661 | 22 | T | C | -0.1948 | 0.0903 | 0.0424 | 4.34E-06 | 21.11 | |
| IL-1RA | rs9985296 | 3 | T | C | 0.1053 | 0.4758 | 0.0231 | 5.15E-06 | 20.78 | |
| IL-2 | rs13412535 | 2 | A | G | 0.174 | 0.1886 | 0.0331 | 1.47E-07 | 27.63 | |
| IL-2 | rs16836080 | 3 | A | G | 0.1158 | 0.3355 | 0.0253 | 4.72E-06 | 20.95 | |
| IL-2 | rs2690020 | 1 | A | G | 0.1158 | 0.5177 | 0.0245 | 2.28E-06 | 22.34 | |
| IL-2 | rs4479767 | 4 | A | G | 0.1821 | 0.8977 | 0.0392 | 3.39E-06 | 21.58 | |
| IL-2 | rs4634519 | 7 | A | G | -0.1249 | 0.7379 | 0.0268 | 3.16E-06 | 21.72 | |
| IL-2 | rs7615304 | 3 | A | G | -0.1139 | 0.4531 | 0.024 | 2.08E-06 | 22.52 | |
| IL-2RA | rs11241559 | 5 | T | G | -0.124 | 0.2643 | 0.0264 | 2.64E-06 | 22.06 | |
| IL-2RA | rs12789243 | 11 | T | C | 0.1263 | 0.7571 | 0.0276 | 4.74E-06 | 20.94 | |
| IL-2RA | rs17624670 | 8 | A | G | -0.125 | 0.2531 | 0.0273 | 4.68E-06 | 20.96 | |
| IL-2RA | rs34353319 | 10 | A | T | -0.1581 | 0.8176 | 0.0335 | 2.37E-06 | 22.27 | |
| IL-2RA | rs56213152 | 7 | T | C | 0.1269 | 0.7688 | 0.0271 | 2.83E-06 | 21.93 | |
| IL-2RA | rs7078614 | 10 | T | G | -0.1543 | 0.3549 | 0.0241 | 1.53E-10 | 40.99 | |
| IL-4 | rs12640583 | 4 | T | G | -0.1104 | 0.1631 | 0.0214 | 2.48E-07 | 26.61 | |
| IL-4 | rs17713451 | 7 | A | G | 0.1255 | 0.1149 | 0.0252 | 6.35E-07 | 24.80 | |
| IL-4 | rs1867282 | 9 | T | C | 0.0808 | 0.4148 | 0.0162 | 6.11E-07 | 24.88 | |
| IL-4 | rs2073438 | 17 | A | G | 0.0847 | 0.2513 | 0.0183 | 3.68E-06 | 21.42 | |
| IL-4 | rs2346020 | 3 | A | G | 0.079 | 0.6795 | 0.0169 | 2.95E-06 | 21.85 | |
| IL-4 | rs2708586 | 7 | T | C | -0.0767 | 0.3411 | 0.0166 | 3.83E-06 | 21.35 | |
| IL-4 | rs7613691 | 3 | A | G | 0.1787 | 0.9482 | 0.0382 | 2.90E-06 | 21.88 | |
| IL-4 | rs9506111 | 13 | A | G | -0.1446 | 0.9326 | 0.0314 | 4.12E-06 | 21.21 | |
| IL-4 | rs9941733 | 20 | A | G | 0.1156 | 0.8355 | 0.0229 | 4.46E-07 | 25.48 | |
| IL-5 | rs10178043 | 2 | T | G | 0.2579 | 0.949 | 0.0553 | 3.11E-06 | 21.75 | |
| IL-5 | rs28793375 | 8 | T | C | 0.1697 | 0.1298 | 0.0362 | 2.76E-06 | 21.98 | |
| IL-5 | rs73040118 | 19 | T | C | 0.2294 | 0.9285 | 0.049 | 2.85E-06 | 21.92 | |
| IL-5 | rs74811276 | 14 | A | G | 0.217 | 0.0761 | 0.0471 | 4.08E-06 | 21.23 | |
| IL-5 | rs7739450 | 6 | A | G | -0.1295 | 0.5158 | 0.0256 | 4.22E-07 | 25.59 | |
| IL-5 | rs9309063 | 2 | T | G | -0.1119 | 0.4763 | 0.0245 | 4.94E-06 | 20.86 | |
| IL-6 | rs10910395 | 1 | A | T | -0.108 | 0.8729 | 0.0235 | 4.31E-06 | 21.12 | |
| IL-6 | rs10982193 | 9 | A | G | -0.0793 | 0.2822 | 0.0174 | 5.18E-06 | 20.77 | |
| IL-6 | rs113098456 | 2 | A | G | -0.1553 | 0.0725 | 0.0339 | 4.62E-06 | 20.99 | |
| IL-6 | rs113600793 | 17 | A | C | 0.1736 | 0.0623 | 0.0359 | 1.33E-06 | 23.38 | |
| IL-6 | rs11732981 | 4 | A | C | 0.0722 | 0.5119 | 0.0156 | 3.69E-06 | 21.42 | |
| IL-6 | rs1333040 | 9 | T | C | 0.0747 | 0.4508 | 0.0157 | 1.96E-06 | 22.64 | |
| IL-6 | rs13412535 | 2 | A | G | -0.1186 | 0.189 | 0.0214 | 2.99E-08 | 30.71 | |
| IL-6 | rs4684700 | 3 | T | C | -0.0747 | 0.5142 | 0.0162 | 4.01E-06 | 21.26 | |
| IL-7 | rs11757972 | 6 | T | C | 0.121 | 0.4776 | 0.0257 | 2.50E-06 | 22.17 | |
| IL-7 | rs1374279 | 2 | A | T | 0.1625 | 0.143 | 0.0347 | 2.83E-06 | 21.93 | |
| IL-7 | rs218238 | 4 | A | T | 0.1319 | 0.7729 | 0.0284 | 3.41E-06 | 21.57 | |
| IL-7 | rs28793375 | 8 | T | C | 0.1644 | 0.1305 | 0.036 | 4.96E-06 | 20.85 | |
| IL-7 | rs62006410 | 14 | T | C | -0.1492 | 0.245 | 0.0302 | 7.80E-07 | 24.41 | |
| IL-7 | rs7155170 | 14 | A | T | -0.1236 | 0.2744 | 0.027 | 4.70E-06 | 20.96 | |
| IL-7 | rs77318030 | 19 | T | C | -0.2966 | 0.9574 | 0.0631 | 2.60E-06 | 22.09 | |
| IL-7 | rs7739450 | 6 | A | G | -0.2907 | 0.5167 | 0.0252 | 8.72E-31 | 133.07 | |
| IL-8 | rs12075 | 1 | A | G | 0.1148 | 0.5353 | 0.0235 | 1.03E-06 | 23.86 | |
| IL-8 | rs12912642 | 15 | A | G | 0.1168 | 0.3302 | 0.0251 | 3.27E-06 | 21.65 | |
| IL-8 | rs2673604 | 8 | A | C | -0.118 | 0.6829 | 0.0254 | 3.39E-06 | 21.58 | |
| IL-8 | rs3786107 | 17 | A | G | 0.2463 | 0.9287 | 0.0517 | 1.90E-06 | 22.70 | |
| IL-9 | rs1259728 | 12 | A | G | -0.2381 | 0.0554 | 0.0507 | 2.65E-06 | 22.05 | |
| IL-9 | rs3736858 | 13 | C | G | -0.1351 | 0.7976 | 0.0291 | 3.44E-06 | 21.55 | |
| IL-9 | rs73443903 | 6 | A | C | 0.2162 | 0.0685 | 0.046 | 2.60E-06 | 22.09 | |
| IP-10 | rs113183470 | 6 | A | T | -0.2414 | 0.946 | 0.0524 | 4.09E-06 | 21.22 | |
| IP-10 | rs12714300 | 2 | A | T | -0.1573 | 0.1378 | 0.0338 | 3.26E-06 | 21.66 | |
| IP-10 | rs397816 | 22 | T | C | 0.1211 | 0.5891 | 0.0248 | 1.04E-06 | 23.84 | |
| IP-10 | rs4859940 | 4 | C | G | -0.1204 | 0.295 | 0.0258 | 3.06E-06 | 21.78 | |
| IP-10 | rs4862110 | 4 | T | C | -0.1453 | 0.771 | 0.0318 | 4.90E-06 | 20.88 | |
| IP-10 | rs7645625 | 3 | T | G | -0.1116 | 0.5724 | 0.0236 | 2.26E-06 | 22.36 | |
| IP-10 | rs79848609 | 15 | A | C | 0.2514 | 0.9449 | 0.0535 | 2.61E-06 | 22.08 | |
| IP-10 | rs8112618 | 19 | A | G | 0.1388 | 0.1841 | 0.0297 | 2.96E-06 | 21.84 | |
| M-CSF | rs116887628 | 8 | A | G | -0.2741 | 0.0611 | 0.0598 | 4.57E-06 | 21.01 | |
| M-CSF | rs12962919 | 18 | T | C | 0.3025 | 0.0644 | 0.0659 | 4.43E-06 | 21.07 | |
| M-CSF | rs34089869 | 2 | T | C | 0.2194 | 0.1039 | 0.0462 | 2.05E-06 | 22.55 | |
| M-CSF | rs62294910 | 3 | A | G | 0.3472 | 0.0504 | 0.0687 | 4.33E-07 | 25.54 | |
| M-CSF | rs72723242 | 5 | T | G | -0.4969 | 0.0177 | 0.1083 | 4.47E-06 | 21.05 | |
| M-CSF | rs9387100 | 6 | T | C | -0.135 | 0.4291 | 0.029 | 3.24E-06 | 21.67 | |
| M-CSF | rs9626985 | 22 | T | C | 0.2277 | 0.0936 | 0.0496 | 4.42E-06 | 21.07 | |
| MCP-1-MCAF | rs12062235 | 1 | T | G | 0.1477 | 0.9377 | 0.032 | 3.92E-06 | 21.30 | |
| MCP-1-MCAF | rs12493953 | 3 | A | G | -0.0948 | 0.3224 | 0.0172 | 3.56E-08 | 30.38 | |
| MCP-1-MCAF | rs16837903 | 1 | A | G | -0.1104 | 0.1237 | 0.0238 | 3.51E-06 | 21.52 | |
| MCP-1-MCAF | rs2820126 | 1 | T | G | 0.0907 | 0.7898 | 0.0193 | 2.61E-06 | 22.09 | |
| MCP-1-MCAF | rs3026968 | 1 | T | C | -0.0896 | 0.3195 | 0.0169 | 1.15E-07 | 28.11 | |
| MCP-1-MCAF | rs34190208 | 3 | T | C | 0.1052 | 0.1511 | 0.0219 | 1.56E-06 | 23.08 | |
| MCP-1-MCAF | rs7197349 | 16 | A | G | 0.0971 | 0.8124 | 0.0206 | 2.43E-06 | 22.22 | |
| MCP-1-MCAF | rs7978037 | 12 | A | T | 0.0746 | 0.3863 | 0.016 | 3.12E-06 | 21.74 | |
| MCP-1-MCAF | rs79939301 | 3 | A | G | 0.1449 | 0.1081 | 0.0255 | 1.33E-08 | 32.29 | |
| MCP-1-MCAF | rs863002 | 1 | T | C | 0.1135 | 0.4077 | 0.0158 | 6.79E-13 | 51.60 | |
| MCP-1-MCAF | rs9317045 | 13 | A | C | 0.1157 | 0.8618 | 0.0235 | 8.51E-07 | 24.24 | |
| MCP-3 | rs10892381 | 11 | T | C | 0.2432 | 0.6629 | 0.0473 | 2.72E-07 | 26.44 | |
| MCP-3 | rs28394764 | 4 | A | T | 0.597 | 0.9696 | 0.1282 | 3.21E-06 | 21.69 | |
| MCP-3 | rs3129806 | 9 | T | C | -0.1975 | 0.5729 | 0.0433 | 5.09E-06 | 20.80 | |
| MCP-3 | rs7275485 | 21 | T | C | -0.2218 | 0.2711 | 0.0481 | 4.00E-06 | 21.26 | |
| MIF | rs1007888 | 22 | T | C | -0.1275 | 0.6128 | 0.0245 | 1.95E-07 | 27.08 | |
| MIF | rs12594190 | 15 | A | G | 0.1321 | 0.7008 | 0.0266 | 6.83E-07 | 24.66 | |
| MIF | rs2294689 | 6 | C | G | -0.1338 | 0.3018 | 0.0287 | 3.13E-06 | 21.73 | |
| MIF | rs35792361 | 4 | A | G | -0.2586 | 0.0591 | 0.0527 | 9.25E-07 | 24.08 | |
| MIF | rs35890933 | 19 | T | G | 0.1676 | 0.8474 | 0.0365 | 4.39E-06 | 21.08 | |
| MIF | rs3814097 | 7 | A | G | -0.1163 | 0.5533 | 0.0251 | 3.60E-06 | 21.47 | |
| MIG | rs10266753 | 7 | T | C | -0.2016 | 0.9109 | 0.0397 | 3.81E-07 | 25.79 | |
| MIG | rs11177248 | 12 | A | G | 0.3157 | 0.0351 | 0.0667 | 2.21E-06 | 22.40 | |
| MIG | rs13143163 | 4 | C | G | 0.2735 | 0.0512 | 0.0582 | 2.61E-06 | 22.08 | |
| MIG | rs3733233 | 4 | T | C | 0.1223 | 0.6588 | 0.025 | 9.98E-07 | 23.93 | |
| MIG | rs6679677 | 1 | A | C | 0.1628 | 0.1458 | 0.0327 | 6.40E-07 | 24.79 | |
| MIG | rs8127917 | 21 | T | G | 0.2382 | 0.0608 | 0.0492 | 1.29E-06 | 23.44 | |
| MIG | rs816960 | 13 | T | C | -0.1179 | 0.3692 | 0.0242 | 1.11E-06 | 23.74 | |
| MIG | rs9456663 | 6 | T | C | -0.1186 | 0.6702 | 0.0255 | 3.30E-06 | 21.63 | |
| MIP-1A | rs12159394 | 22 | A | G | -0.1708 | 0.1183 | 0.0366 | 3.06E-06 | 21.78 | |
| MIP-1A | rs57786342 | 14 | A | G | 0.139 | 0.2336 | 0.0283 | 9.03E-07 | 24.12 | |
| MIP-1A | rs6900267 | 6 | A | C | -0.2472 | 0.9308 | 0.0515 | 1.59E-06 | 23.04 | |
| MIP-1A | rs6956239 | 7 | T | C | 0.119 | 0.2927 | 0.026 | 4.72E-06 | 20.95 | |
| MIP-1B | rs117010890 | 17 | T | C | -0.2043 | 0.9347 | 0.0393 | 2.01E-07 | 27.02 | |
| MIP-1B | rs11716293 | 3 | C | G | 0.0986 | 0.7698 | 0.0189 | 1.82E-07 | 27.22 | |
| MIP-1B | rs117657747 | 18 | A | G | 0.2089 | 0.0526 | 0.0453 | 4.00E-06 | 21.27 | |
| MIP-1B | rs12452320 | 17 | A | C | 0.2139 | 0.0716 | 0.0319 | 2.01E-11 | 44.96 | |
| MIP-1B | rs12951603 | 17 | A | G | -0.1132 | 0.1492 | 0.0225 | 4.88E-07 | 25.31 | |
| MIP-1B | rs1437220 | 17 | T | C | 0.1437 | 0.9207 | 0.0315 | 5.07E-06 | 20.81 | |
| MIP-1B | rs148561432 | 17 | A | G | -0.2691 | 0.0454 | 0.0407 | 3.80E-11 | 43.72 | |
| MIP-1B | rs1543292 | 17 | A | G | 0.2627 | 0.0632 | 0.0338 | 7.71E-15 | 60.41 | |
| MIP-1B | rs17138331 | 7 | A | G | -0.1434 | 0.9092 | 0.0295 | 1.17E-06 | 23.63 | |
| MIP-1B | rs1867288 | 17 | C | G | 0.2034 | 0.2288 | 0.0215 | 3.07E-21 | 89.50 | |
| MIP-1B | rs2131092 | 3 | A | G | -0.1278 | 0.1103 | 0.0248 | 2.56E-07 | 26.56 | |
| MIP-1B | rs2276857 | 3 | T | C | -0.1283 | 0.1356 | 0.0257 | 5.97E-07 | 24.92 | |
| MIP-1B | rs2314809 | 17 | T | C | -0.0735 | 0.5305 | 0.0157 | 2.85E-06 | 21.92 | |
| MIP-1B | rs2376263 | 17 | A | G | 0.1053 | 0.7795 | 0.0187 | 1.79E-08 | 31.71 | |
| MIP-1B | rs2411161 | 17 | T | C | 0.1719 | 0.9508 | 0.0365 | 2.48E-06 | 22.18 | |
| MIP-1B | rs2673059 | 3 | T | C | 0.0921 | 0.7787 | 0.0192 | 1.61E-06 | 23.01 | |
| MIP-1B | rs281728 | 8 | A | C | -0.079 | 0.7058 | 0.0171 | 3.84E-06 | 21.34 | |
| MIP-1B | rs28393318 | 4 | A | G | -0.1076 | 0.8758 | 0.0235 | 4.68E-06 | 20.96 | |
| MIP-1B | rs28856610 | 17 | T | C | -0.3074 | 0.031 | 0.0495 | 5.30E-10 | 38.57 | |
| MIP-1B | rs323877 | 3 | C | G | -0.0965 | 0.6443 | 0.0172 | 2.02E-08 | 31.48 | |
| MIP-1B | rs35933743 | 17 | T | G | -0.1183 | 0.8438 | 0.0238 | 6.68E-07 | 24.71 | |
| MIP-1B | rs41290648 | 3 | A | G | 0.2231 | 0.1266 | 0.0239 | 1.01E-20 | 87.14 | |
| MIP-1B | rs41341749 | 17 | A | G | -0.1656 | 0.9203 | 0.0294 | 1.77E-08 | 31.73 | |
| MIP-1B | rs41502550 | 3 | T | C | 0.1266 | 0.8499 | 0.022 | 8.69E-09 | 33.11 | |
| MIP-1B | rs4795931 | 17 | A | G | -0.0865 | 0.3193 | 0.0173 | 5.73E-07 | 25.00 | |
| MIP-1B | rs56083628 | 17 | T | C | -0.1297 | 0.1054 | 0.0257 | 4.50E-07 | 25.47 | |
| MIP-1B | rs6908843 | 6 | A | G | 0.0997 | 0.1684 | 0.0209 | 1.84E-06 | 22.76 | |
| MIP-1B | rs72791296 | 5 | T | C | 0.2364 | 0.0349 | 0.0466 | 3.92E-07 | 25.73 | |
| MIP-1B | rs72799710 | 5 | T | C | -0.1037 | 0.1514 | 0.0217 | 1.76E-06 | 22.84 | |
| MIP-1B | rs72820112 | 17 | T | C | 0.1059 | 0.7412 | 0.0183 | 7.17E-09 | 33.49 | |
| MIP-1B | rs72820246 | 17 | T | G | -0.0983 | 0.4163 | 0.0167 | 3.95E-09 | 34.65 | |
| MIP-1B | rs72829264 | 17 | A | G | -0.1517 | 0.8833 | 0.0277 | 4.34E-08 | 29.99 | |
| MIP-1B | rs76842834 | 17 | T | C | -0.4207 | 0.0371 | 0.0471 | 4.18E-19 | 79.78 | |
| MIP-1B | rs76863419 | 17 | T | G | -0.2833 | 0.073 | 0.0341 | 9.74E-17 | 69.02 | |
| MIP-1B | rs8081726 | 17 | T | C | -0.3499 | 0.0491 | 0.036 | 2.49E-22 | 94.47 | |
| MIP-1B | rs854222 | 3 | A | C | 0.0809 | 0.2696 | 0.0177 | 4.86E-06 | 20.89 | |
| MIP-1B | rs873944 | 17 | T | C | 0.2392 | 0.0726 | 0.0322 | 1.10E-13 | 55.18 | |
| MIP-1B | rs939408 | 3 | A | C | -0.1007 | 0.3938 | 0.016 | 3.10E-10 | 39.61 | |
| MIP-1B | rs951814 | 17 | A | G | 0.1614 | 0.07 | 0.0315 | 2.99E-07 | 26.25 | |
| MIP-1B | rs9838883 | 3 | T | C | 0.079 | 0.3399 | 0.0166 | 1.95E-06 | 22.65 | |
| MIP-1B | rs9911839 | 17 | T | G | -0.1377 | 0.9011 | 0.027 | 3.40E-07 | 26.01 | |
| MIP-1B | rs9914803 | 17 | T | C | 0.098 | 0.5189 | 0.0156 | 3.34E-10 | 39.46 | |
| PDGF-BB | rs11766649 | 7 | A | G | 0.0902 | 0.8018 | 0.0196 | 4.18E-06 | 21.18 | |
| PDGF-BB | rs12289510 | 11 | A | G | -0.0772 | 0.4767 | 0.0158 | 1.03E-06 | 23.87 | |
| PDGF-BB | rs12615784 | 2 | T | C | -0.1003 | 0.7849 | 0.0193 | 2.03E-07 | 27.01 | |
| PDGF-BB | rs13037046 | 20 | A | T | -0.0948 | 0.1871 | 0.0206 | 4.19E-06 | 21.18 | |
| PDGF-BB | rs2643354 | 15 | A | G | 0.1251 | 0.8921 | 0.0261 | 1.64E-06 | 22.97 | |
| PDGF-BB | rs34131731 | 2 | A | T | 0.2083 | 0.9594 | 0.0411 | 4.02E-07 | 25.69 | |
| PDGF-BB | rs62191444 | 20 | T | G | -0.112 | 0.1492 | 0.0239 | 2.78E-06 | 21.96 | |
| PDGF-BB | rs6910518 | 6 | T | G | 0.0806 | 0.5924 | 0.0162 | 6.51E-07 | 24.75 | |
| PDGF-BB | rs7170232 | 15 | T | C | 0.1609 | 0.219 | 0.0189 | 1.69E-17 | 72.48 | |
| PDGF-BB | rs9924851 | 16 | C | G | 0.0767 | 0.3564 | 0.0163 | 2.53E-06 | 22.14 | |
| RANTES | rs10505135 | 8 | T | C | 0.1315 | 0.3704 | 0.0252 | 1.81E-07 | 27.23 | |
| RANTES | rs2731672 | 5 | T | C | -0.1242 | 0.267 | 0.0272 | 4.97E-06 | 20.85 | |
| RANTES | rs4795087 | 17 | C | G | 0.1494 | 0.8188 | 0.0312 | 1.68E-06 | 22.93 | |
| RANTES | rs62438851 | 6 | A | G | -0.1904 | 0.8961 | 0.0413 | 4.02E-06 | 21.25 | |
| RANTES | rs72793342 | 16 | A | G | -0.1505 | 0.2003 | 0.0307 | 9.47E-07 | 24.03 | |
| SCF | rs10800449 | 1 | A | C | 0.0851 | 0.2859 | 0.0179 | 1.99E-06 | 22.60 | |
| SCF | rs11244035 | 9 | T | C | -0.1296 | 0.0944 | 0.0279 | 3.40E-06 | 21.58 | |
| SCF | rs113127926 | 14 | A | C | 0.1974 | 0.0418 | 0.0418 | 2.33E-06 | 22.30 | |
| SCF | rs117721699 | 9 | C | G | -0.2392 | 0.0265 | 0.0484 | 7.73E-07 | 24.42 | |
| SCF | rs12345108 | 9 | T | C | -0.0772 | 0.3395 | 0.0167 | 3.79E-06 | 21.37 | |
| SCF | rs13412535 | 2 | A | G | -0.1065 | 0.1892 | 0.0213 | 5.73E-07 | 25.00 | |
| SCF | rs1536480 | 9 | T | C | 0.081 | 0.3204 | 0.0167 | 1.23E-06 | 23.53 | |
| SCF | rs72678285 | 14 | A | T | 0.1062 | 0.8512 | 0.0231 | 4.28E-06 | 21.14 | |
| SCGF-B | rs12118918 | 1 | A | G | -0.1631 | 0.8669 | 0.035 | 3.16E-06 | 21.72 | |
| SCGF-B | rs12480722 | 20 | T | C | 0.1654 | 0.8752 | 0.0353 | 2.79E-06 | 21.95 | |
| SCGF-B | rs13287050 | 9 | A | T | -0.121 | 0.7202 | 0.0263 | 4.21E-06 | 21.17 | |
| SCGF-B | rs13866 | 19 | T | C | -0.1647 | 0.2622 | 0.028 | 4.05E-09 | 34.60 | |
| SCGF-B | rs143829871 | 3 | T | C | -0.1866 | 0.9047 | 0.0399 | 2.92E-06 | 21.87 | |
| SCGF-B | rs264157 | 18 | A | G | 0.1079 | 0.4743 | 0.0233 | 3.64E-06 | 21.45 | |
| SCGF-B | rs3817303 | 12 | T | G | 0.1362 | 0.194 | 0.0294 | 3.61E-06 | 21.46 | |
| SCGF-B | rs4737731 | 8 | T | C | 0.1146 | 0.3019 | 0.0251 | 4.98E-06 | 20.85 | |
| SCGF-B | rs4976691 | 5 | C | G | -0.1484 | 0.3257 | 0.0253 | 4.47E-09 | 34.41 | |
| SCGF-B | rs77954165 | 9 | T | C | 0.2631 | 0.9528 | 0.0562 | 2.85E-06 | 21.92 | |
| SCGF-B | rs7802293 | 7 | T | C | -0.1145 | 0.3779 | 0.0247 | 3.56E-06 | 21.49 | |
| SDF-1A | rs1600396 | 4 | A | G | -0.0933 | 0.8096 | 0.0204 | 4.80E-06 | 20.92 | |
| SDF-1A | rs62194946 | 2 | T | G | -0.0849 | 0.2628 | 0.0185 | 4.45E-06 | 21.06 | |
| SDF-1A | rs6586903 | 8 | T | C | -0.1264 | 0.1037 | 0.0268 | 2.40E-06 | 22.24 | |
| SDF-1A | rs78883416 | 10 | C | G | -0.0871 | 0.308 | 0.0182 | 1.70E-06 | 22.90 | |
| TNF-A | rs10767536 | 11 | A | G | 0.118 | 0.3274 | 0.0253 | 3.10E-06 | 21.75 | |
| TNF-A | rs116736594 | 5 | T | C | 0.3407 | 0.0312 | 0.0702 | 1.21E-06 | 23.55 | |
| TNF-A | rs7256693 | 19 | T | C | -0.1841 | 0.8925 | 0.04 | 4.17E-06 | 21.18 | |
| TNF-B | rs10925040 | 1 | T | C | 0.1738 | 0.371 | 0.0372 | 2.98E-06 | 21.83 | |
| TNF-B | rs2420873 | 19 | T | G | 0.1673 | 0.3897 | 0.0365 | 4.57E-06 | 21.01 | |
| TNF-B | rs62284710 | 3 | A | G | 0.3702 | 0.9443 | 0.0782 | 2.20E-06 | 22.41 | |
| TNF-B | rs75240021 | 8 | C | G | 0.3713 | 0.071 | 0.0772 | 1.51E-06 | 23.13 | |
| TRAIL | rs10084050 | 18 | A | G | -0.1101 | 0.8647 | 0.023 | 1.69E-06 | 22.91 | |
| TRAIL | rs10164260 | 18 | A | G | 0.1003 | 0.1741 | 0.0211 | 2.00E-06 | 22.60 | |
| TRAIL | rs11875481 | 18 | T | C | -0.0969 | 0.835 | 0.0211 | 4.38E-06 | 21.09 | |
| TRAIL | rs13115587 | 4 | A | C | 0.101 | 0.1558 | 0.0217 | 3.25E-06 | 21.66 | |
| TRAIL | rs13278062 | 8 | T | G | 0.08 | 0.5589 | 0.0157 | 3.48E-07 | 25.96 | |
| TRAIL | rs146827832 | 3 | T | C | 0.1341 | 0.9038 | 0.0291 | 4.06E-06 | 21.24 | |
| TRAIL | rs17535790 | 3 | A | G | -0.1125 | 0.1498 | 0.0218 | 2.46E-07 | 26.63 | |
| TRAIL | rs550057 | 9 | T | C | -0.0783 | 0.3049 | 0.0169 | 3.60E-06 | 21.47 | |
| TRAIL | rs558572 | 3 | T | C | 0.1351 | 0.0949 | 0.0265 | 3.43E-07 | 25.99 | |
| TRAIL | rs7233927 | 18 | A | G | 0.0905 | 0.6648 | 0.0164 | 3.42E-08 | 30.45 | |
| TRAIL | rs73408359 | 18 | T | C | 0.4153 | 0.049 | 0.0364 | 3.76E-30 | 130.17 | |
| TRAIL | rs74488044 | 18 | A | G | 0.3473 | 0.0583 | 0.0334 | 2.53E-25 | 108.12 | |
| TRAIL | rs747324 | 14 | T | C | -0.0826 | 0.2828 | 0.0178 | 3.48E-06 | 21.53 | |
| TRAIL | rs75473890 | 18 | T | C | -0.1349 | 0.9136 | 0.028 | 1.45E-06 | 23.21 | |
| TRAIL | rs75489499 | 3 | T | C | -0.2006 | 0.0677 | 0.0347 | 7.43E-09 | 33.42 | |
| TRAIL | rs7599203 | 2 | T | C | 0.0918 | 0.8034 | 0.02 | 4.43E-06 | 21.07 | |
| TRAIL | rs9946486 | 18 | A | G | 0.1856 | 0.1159 | 0.0245 | 3.58E-14 | 57.39 | |
| VEGF | rs10411345 | 19 | C | G | -0.1041 | 0.7823 | 0.0218 | 1.80E-06 | 22.80 | |
| VEGF | rs10757514 | 9 | C | G | -0.1024 | 0.1715 | 0.0222 | 3.98E-06 | 21.28 | |
| VEGF | rs10822118 | 10 | T | C | -0.0797 | 0.4984 | 0.0168 | 2.09E-06 | 22.51 | |
| VEGF | rs10934631 | 3 | T | C | -0.1132 | 0.8517 | 0.0244 | 3.50E-06 | 21.52 | |
| VEGF | rs10967183 | 9 | T | C | -0.0887 | 0.4262 | 0.0169 | 1.53E-07 | 27.55 | |
| VEGF | rs12156533 | 9 | A | T | 0.0915 | 0.6655 | 0.0186 | 8.68E-07 | 24.20 | |
| VEGF | rs12456390 | 18 | T | C | -0.0818 | 0.6772 | 0.0179 | 4.88E-06 | 20.88 | |
| VEGF | rs13190738 | 6 | T | C | 0.1111 | 0.3644 | 0.0231 | 1.51E-06 | 23.13 | |
| VEGF | rs3025020 | 6 | T | C | -0.124 | 0.2786 | 0.0253 | 9.53E-07 | 24.02 | |
| VEGF | rs4573079 | 6 | A | C | 0.1522 | 0.8439 | 0.0256 | 2.76E-09 | 35.35 | |
| VEGF | rs56071907 | 16 | T | C | 0.126 | 0.1319 | 0.027 | 3.06E-06 | 21.78 | |
| VEGF | rs58078557 | 9 | A | T | -0.1168 | 0.1473 | 0.024 | 1.13E-06 | 23.68 | |
| VEGF | rs60013354 | 10 | A | G | -0.2497 | 0.0266 | 0.0521 | 1.65E-06 | 22.97 | |
| VEGF | rs60987108 | 6 | A | G | 0.1812 | 0.9459 | 0.039 | 3.38E-06 | 21.59 | |
| VEGF | rs7739450 | 6 | A | G | -0.415 | 0.5059 | 0.018 | 1.29E-117 | | 531.56 |
| VEGF | rs7754905 | 6 | A | G | -0.1303 | 0.7682 | 0.0204 | 1.69E-10 | | 40.80 |
| VEGF | rs7757024 | 6 | T | C | 0.1443 | 0.0973 | 0.0291 | 7.09E-07 | 24.59 | |
| VEGF | rs9369440 | 6 | T | C | -0.0872 | 0.2934 | 0.0186 | 2.76E-06 | 21.98 | |
| VEGF | rs9472153 | 6 | A | G | 0.106 | 0.5187 | 0.0174 | 1.12E-09 | 37.11 | |

5.Instrumental variables for inflammatory factors and RA

| **Exposure** | **SNP** | **Chr** | **EA** | **OA** | **Beta** | **EAF** | **SE** | **P** | **F** | |
| --- | --- | --- | --- | --- | --- | --- | --- | --- | --- | --- |
| B-NGF | rs28637706 | 19 | T | G | -0.1554 | 0.314 | 0.0261 | 2.62E-09 | 35.45 | |
| B-NGF | rs4767014 | 12 | T | C | -0.1211 | 0.6731 | 0.0264 | 4.49E-06 | 21.04 | |
| B-NGF | rs71641308 | 1 | T | C | 0.1969 | 0.0995 | 0.0429 | 4.44E-06 | 21.07 | |
| B-NGF | rs73472576 | 18 | T | C | -0.1146 | 0.457 | 0.0251 | 4.98E-06 | 20.85 | |
| CTACK | rs10854859 | 22 | A | G | -0.1498 | 0.2464 | 0.0293 | 3.18E-07 | 26.14 | |
| CTACK | rs116303454 | 3 | A | G | 0.3754 | 0.0218 | 0.081 | 3.58E-06 | 21.48 | |
| CTACK | rs116871507 | 9 | A | T | -0.2086 | 0.0791 | 0.0448 | 3.22E-06 | 21.68 | |
| CTACK | rs116943377 | 13 | A | G | 0.2878 | 0.0394 | 0.0611 | 2.47E-06 | 22.19 | |
| CTACK | rs117932939 | 9 | T | C | 0.1969 | 0.09 | 0.0422 | 3.07E-06 | 21.77 | |
| CTACK | rs144072067 | 9 | A | G | 0.4451 | 0.9574 | 0.0579 | 1.50E-14 | 59.10 | |
| CTACK | rs184329319 | 9 | T | G | -0.3069 | 0.035 | 0.0648 | 2.18E-06 | 22.43 | |
| CTACK | rs2233872 | 9 | A | G | -0.1495 | 0.7699 | 0.0282 | 1.15E-07 | 28.11 | |
| CTACK | rs55764737 | 15 | T | C | 0.5424 | 0.9846 | 0.0967 | 2.03E-08 | 31.46 | |
| CTACK | rs57338032 | 15 | A | G | 0.1443 | 0.8287 | 0.0316 | 4.96E-06 | 20.85 | |
| CTACK | rs57789542 | 3 | T | C | -0.7687 | 0.9951 | 0.1659 | 3.60E-06 | 21.47 | |
| CTACK | rs60247384 | 3 | T | C | 0.1128 | 0.3512 | 0.0245 | 4.14E-06 | 21.20 | |
| CTACK | rs62578137 | 9 | T | C | -0.1311 | 0.2421 | 0.0286 | 4.56E-06 | 21.01 | |
| CTACK | rs72729450 | 9 | T | C | -0.5123 | 0.0123 | 0.1094 | 2.83E-06 | 21.93 | |
| CTACK | rs76395525 | 15 | A | G | 0.5193 | 0.0118 | 0.1081 | 1.56E-06 | 23.08 | |
| EOTAXIN | rs11920996 | 3 | T | C | 0.2979 | 0.0512 | 0.0377 | 2.75E-15 | 62.44 | |
| EOTAXIN | rs147287945 | 6 | A | G | -0.1512 | 0.0741 | 0.0313 | 1.36E-06 | 23.34 | |
| EOTAXIN | rs1677588 | 1 | T | G | 0.1181 | 0.886 | 0.025 | 2.31E-06 | 22.32 | |
| EOTAXIN | rs2024050 | 7 | A | G | 0.164 | 0.0739 | 0.0302 | 5.62E-08 | 29.49 | |
| EOTAXIN | rs2027855 | 22 | T | C | 0.0743 | 0.6153 | 0.0162 | 4.51E-06 | 21.04 | |
| EOTAXIN | rs2040143 | 21 | A | G | -0.0858 | 0.7234 | 0.0178 | 1.43E-06 | 23.23 | |
| EOTAXIN | rs2229593 | 3 | T | C | 0.3647 | 0.0387 | 0.0406 | 2.64E-19 | 80.69 | |
| EOTAXIN | rs2249581 | 1 | T | C | -0.0899 | 0.748 | 0.018 | 5.90E-07 | 24.94 | |
| EOTAXIN | rs2828756 | 21 | T | C | 0.0904 | 0.2423 | 0.0184 | 8.97E-07 | 24.14 | |
| EOTAXIN | rs34004101 | 3 | T | C | -0.1775 | 0.065 | 0.0341 | 1.94E-07 | 27.09 | |
| EOTAXIN | rs4683182 | 3 | A | G | 0.0886 | 0.2201 | 0.0189 | 2.76E-06 | 21.98 | |
| EOTAXIN | rs5754733 | 22 | A | C | -0.105 | 0.8306 | 0.0213 | 8.24E-07 | 24.30 | |
| EOTAXIN | rs57723662 | 17 | C | G | -0.0982 | 0.1657 | 0.0213 | 4.02E-06 | 21.26 | |
| EOTAXIN | rs60075014 | 5 | T | C | -0.1688 | 0.0604 | 0.0356 | 2.12E-06 | 22.48 | |
| EOTAXIN | rs7231030 | 18 | A | C | 0.0903 | 0.7919 | 0.0193 | 2.89E-06 | 21.89 | |
| EOTAXIN | rs73065695 | 3 | A | G | 0.1532 | 0.0932 | 0.0281 | 4.98E-08 | 29.72 | |
| EOTAXIN | rs745331 | 15 | A | G | -0.0821 | 0.6825 | 0.0176 | 3.09E-06 | 21.76 | |
| EOTAXIN | rs75426604 | 14 | A | C | -0.1371 | 0.0876 | 0.0291 | 2.46E-06 | 22.20 | |
| EOTAXIN | rs7550207 | 1 | T | C | -0.0904 | 0.7785 | 0.0187 | 1.34E-06 | 23.37 | |
| EOTAXIN | rs9317045 | 13 | A | C | 0.1172 | 0.8615 | 0.0236 | 6.83E-07 | 24.66 | |
| EOTAXIN | rs9833459 | 3 | T | C | -0.1149 | 0.3033 | 0.0173 | 3.10E-11 | 44.11 | |
| FGF-BASIC | rs13412535 | 2 | A | G | -0.1129 | 0.1856 | 0.0224 | 4.65E-07 | 25.40 | |
| FGF-BASIC | rs17094040 | 14 | T | C | 0.1051 | 0.1522 | 0.0229 | 4.44E-06 | 21.06 | |
| FGF-BASIC | rs2849358 | 18 | A | G | 0.0911 | 0.2551 | 0.0193 | 2.36E-06 | 22.28 | |
| FGF-BASIC | rs4795091 | 17 | A | G | 0.1239 | 0.8926 | 0.0266 | 3.19E-06 | 21.70 | |
| FGF-BASIC | rs76253061 | 5 | T | C | -0.4811 | 0.9925 | 0.1041 | 3.81E-06 | 21.36 | |
| FGF-BASIC | rs78873483 | 17 | A | G | 0.1286 | 0.1025 | 0.0282 | 5.11E-06 | 20.80 | |
| G-CSF | rs10939033 | 4 | A | G | -0.0775 | 0.6038 | 0.0163 | 1.99E-06 | 22.61 | |
| G-CSF | rs117261691 | 19 | T | C | 0.1318 | 0.0894 | 0.0288 | 4.73E-06 | 20.94 | |
| G-CSF | rs183023730 | 9 | T | G | 0.7898 | 0.0034 | 0.1677 | 2.48E-06 | 22.18 | |
| G-CSF | rs586802 | 11 | A | G | 0.0882 | 0.758 | 0.0187 | 2.40E-06 | 22.25 | |
| G-CSF | rs6740648 | 2 | T | C | 0.0818 | 0.6752 | 0.0172 | 1.98E-06 | 22.62 | |
| G-CSF | rs74148555 | 10 | T | C | -0.3771 | 0.0144 | 0.0753 | 5.50E-07 | 25.08 | |
| G-CSF | rs76287671 | 19 | T | C | 0.0894 | 0.2333 | 0.0189 | 2.24E-06 | 22.37 | |
| G-CSF | rs77318030 | 19 | T | C | -0.2031 | 0.9563 | 0.0427 | 1.97E-06 | 22.62 | |
| G-CSF | rs78523761 | 5 | A | G | 0.5374 | 0.0077 | 0.1139 | 2.38E-06 | 22.26 | |
| GROA | rs10015342 | 4 | A | T | 0.1945 | 0.1909 | 0.0304 | 1.57E-10 | 40.93 | |
| GROA | rs115214168 | 4 | T | C | 0.4528 | 0.0242 | 0.0828 | 4.54E-08 | 29.91 | |
| GROA | rs1361829 | 1 | A | G | -0.1106 | 0.4293 | 0.0241 | 4.45E-06 | 21.06 | |
| GROA | rs140734053 | 10 | A | G | 0.7333 | 0.0066 | 0.1545 | 2.07E-06 | 22.53 | |
| GROA | rs150194856 | 1 | T | C | -0.4223 | 0.0177 | 0.0914 | 3.83E-06 | 21.35 | |
| GROA | rs17171245 | 7 | T | G | 0.2446 | 0.0536 | 0.053 | 3.93E-06 | 21.30 | |
| GROA | rs185768063 | 6 | A | G | 0.4038 | 0.9735 | 0.076 | 1.08E-07 | 28.23 | |
| GROA | rs188345231 | 8 | T | C | 0.6177 | 0.0102 | 0.1322 | 2.98E-06 | 21.83 | |
| GROA | rs3026943 | 1 | A | C | -0.1246 | 0.3311 | 0.0256 | 1.13E-06 | 23.69 | |
| GROA | rs3845622 | 1 | A | C | -0.2345 | 0.1171 | 0.0381 | 7.51E-10 | 37.88 | |
| GROA | rs62024303 | 15 | A | G | -0.3013 | 0.9617 | 0.066 | 4.99E-06 | 20.84 | |
| GROA | rs73020704 | 1 | A | G | 0.2607 | 0.9484 | 0.0545 | 1.72E-06 | 22.88 | |
| GROA | rs76215157 | 20 | C | G | -0.7398 | 0.0074 | 0.1564 | 2.24E-06 | 22.37 | |
| HGF | rs11060254 | 12 | A | G | -0.0765 | 0.3306 | 0.0166 | 4.06E-06 | 21.24 | |
| HGF | rs11129909 | 3 | T | C | -0.0738 | 0.623 | 0.0161 | 4.56E-06 | 21.01 | |
| HGF | rs13412535 | 2 | A | G | -0.1043 | 0.1892 | 0.0213 | 9.75E-07 | 23.98 | |
| HGF | rs2003620 | 7 | T | C | 0.2277 | 0.027 | 0.0487 | 2.93E-06 | 21.86 | |
| HGF | rs2699434 | 4 | T | C | 0.0863 | 0.3287 | 0.0171 | 4.49E-07 | 25.47 | |
| HGF | rs362307 | 4 | T | C | 0.1511 | 0.0646 | 0.0328 | 4.09E-06 | 21.22 | |
| HGF | rs4245058 | 11 | T | C | -0.1552 | 0.0619 | 0.0331 | 2.75E-06 | 21.99 | |
| HGF | rs57146176 | 12 | A | G | -0.0987 | 0.7998 | 0.0208 | 2.08E-06 | 22.52 | |
| HGF | rs5745687 | 7 | T | C | -0.3008 | 0.0375 | 0.0404 | 9.65E-14 | 55.44 | |
| HGF | rs80051150 | 21 | T | C | 0.198 | 0.9595 | 0.0413 | 1.63E-06 | 22.98 | |
| IFN-G | rs113399544 | 7 | A | G | -0.0849 | 0.2768 | 0.0183 | 3.50E-06 | 21.52 | |
| IFN-G | rs117046255 | 7 | T | C | -0.0968 | 0.1922 | 0.0207 | 2.92E-06 | 21.87 | |
| IFN-G | rs11843756 | 13 | T | G | 0.1812 | 0.9542 | 0.0391 | 3.58E-06 | 21.48 | |
| IFN-G | rs12420286 | 11 | T | C | 0.2357 | 0.9699 | 0.05 | 2.43E-06 | 22.22 | |
| IFN-G | rs147378920 | 1 | A | G | -0.384 | 0.9871 | 0.0751 | 3.17E-07 | 26.14 | |
| IFN-G | rs1867282 | 9 | T | C | 0.0781 | 0.4154 | 0.0166 | 2.54E-06 | 22.14 | |
| IFN-G | rs2073438 | 17 | A | G | 0.092 | 0.2506 | 0.0188 | 9.90E-07 | 23.95 | |
| IFN-G | rs7088799 | 10 | T | G | -0.0805 | 0.6182 | 0.0166 | 1.24E-06 | 23.52 | |
| IFN-G | rs73479333 | 6 | C | G | -0.1123 | 0.1354 | 0.024 | 2.88E-06 | 21.89 | |
| IFN-G | rs74148555 | 10 | T | C | -0.3771 | 0.0144 | 0.077 | 9.71E-07 | 23.98 | |
| IL-10 | rs10457128 | 6 | A | G | -0.0854 | 0.639 | 0.0172 | 6.87E-07 | 24.65 | |
| IL-10 | rs10493718 | 1 | A | C | -0.1081 | 0.1603 | 0.0222 | 1.12E-06 | 23.71 | |
| IL-10 | rs13412535 | 2 | A | G | -0.1347 | 0.1856 | 0.0224 | 1.82E-09 | 36.16 | |
| IL-10 | rs1530455 | 3 | T | C | 0.082 | 0.3523 | 0.0174 | 2.45E-06 | 22.21 | |
| IL-10 | rs2086656 | 4 | T | C | -0.08 | 0.6621 | 0.017 | 2.53E-06 | 22.15 | |
| IL-10 | rs3025021 | 6 | T | C | 0.0913 | 0.3459 | 0.0194 | 2.52E-06 | 22.15 | |
| IL-10 | rs339203 | 2 | T | C | 0.0954 | 0.8359 | 0.0203 | 2.61E-06 | 22.09 | |
| IL-10 | rs383684 | 6 | A | G | 0.092 | 0.7555 | 0.0197 | 3.01E-06 | 21.81 | |
| IL-10 | rs41282660 | 6 | A | G | -0.1169 | 0.8646 | 0.0254 | 4.18E-06 | 21.18 | |
| IL-10 | rs465757 | 20 | A | G | 0.0806 | 0.6657 | 0.0174 | 3.62E-06 | 21.46 | |
| IL-10 | rs4741748 | 9 | A | G | -0.0788 | 0.6081 | 0.0169 | 3.12E-06 | 21.74 | |
| IL-10 | rs6054847 | 20 | T | C | 0.0971 | 0.8091 | 0.0207 | 2.72E-06 | 22.00 | |
| IL-10 | rs6680918 | 1 | T | C | -0.1202 | 0.8791 | 0.025 | 1.52E-06 | 23.12 | |
| IL-10 | rs7088799 | 10 | T | G | -0.0815 | 0.6191 | 0.0166 | 9.12E-07 | 24.10 | |
| IL-10 | rs73192842 | 3 | A | G | 0.0949 | 0.1911 | 0.0206 | 4.09E-06 | 21.22 | |
| IL-10 | rs7747448 | 6 | A | G | -0.1061 | 0.2594 | 0.0189 | 1.98E-08 | 31.51 | |
| IL-10 | rs865585 | 6 | A | C | -0.119 | 0.8478 | 0.0244 | 1.08E-06 | 23.79 | |
| IL-10 | rs910604 | 6 | A | G | -0.0986 | 0.2383 | 0.0196 | 4.89E-07 | 25.31 | |
| IL-12-P70 | rs12154194 | 6 | T | C | 0.0717 | 0.4615 | 0.0157 | 4.95E-06 | 20.86 | |
| IL-12-P70 | rs12969892 | 18 | T | C | 0.1227 | 0.9031 | 0.0267 | 4.32E-06 | 21.12 | |
| IL-12-P70 | rs13190738 | 6 | T | C | 0.1019 | 0.3609 | 0.019 | 8.18E-08 | 28.76 | |
| IL-12-P70 | rs2123852 | 19 | T | C | 0.0942 | 0.2039 | 0.0204 | 3.88E-06 | 21.32 | |
| IL-12-P70 | rs2495005 | 10 | A | G | -0.075 | 0.4416 | 0.0159 | 2.39E-06 | 22.25 | |
| IL-12-P70 | rs273702 | 18 | A | G | -0.127 | 0.9015 | 0.027 | 2.55E-06 | 22.12 | |
| IL-12-P70 | rs282258 | 2 | T | C | 0.0726 | 0.4279 | 0.0156 | 3.26E-06 | 21.66 | |
| IL-12-P70 | rs34322762 | 9 | T | C | 0.0953 | 0.648 | 0.0199 | 1.68E-06 | 22.93 | |
| IL-12-P70 | rs34826779 | 8 | T | G | -0.0884 | 0.2157 | 0.019 | 3.28E-06 | 21.65 | |
| IL-12-P70 | rs41282644 | 6 | A | G | 0.1401 | 0.0849 | 0.0303 | 3.77E-06 | 21.38 | |
| IL-12-P70 | rs4714698 | 6 | A | G | -0.1189 | 0.8721 | 0.0238 | 5.86E-07 | 24.96 | |
| IL-12-P70 | rs4741748 | 9 | A | G | -0.0799 | 0.6076 | 0.0163 | 9.49E-07 | 24.03 | |
| IL-12-P70 | rs6458375 | 6 | T | C | 0.0884 | 0.2407 | 0.0191 | 3.69E-06 | 21.42 | |
| IL-12-P70 | rs6532374 | 4 | T | C | -0.1033 | 0.8525 | 0.0226 | 4.86E-06 | 20.89 | |
| IL-12-P70 | rs7763358 | 6 | T | C | 0.1525 | 0.0885 | 0.0274 | 2.61E-08 | 30.98 | |
| IL-12-P70 | rs7765264 | 6 | A | G | 0.1428 | 0.0767 | 0.0301 | 2.09E-06 | 22.51 | |
| IL-12-P70 | rs782111 | 12 | A | C | -0.0765 | 0.5292 | 0.0156 | 9.40E-07 | 24.05 | |
| IL-12-P70 | rs865585 | 6 | A | C | -0.1654 | 0.8484 | 0.0237 | 2.97E-12 | 48.71 | |
| IL-12-P70 | rs9381249 | 6 | T | C | -0.1788 | 0.9466 | 0.0367 | 1.11E-06 | 23.74 | |
| IL-12-P70 | rs9472153 | 6 | A | G | 0.0855 | 0.5141 | 0.0159 | 7.56E-08 | 28.92 | |
| IL-12-P70 | rs9472175 | 6 | T | C | -0.1104 | 0.1542 | 0.0238 | 3.51E-06 | 21.52 | |
| IL-13 | rs10995604 | 10 | A | G | -0.1571 | 0.8587 | 0.0343 | 4.65E-06 | 20.98 | |
| IL-13 | rs117795020 | 9 | A | G | -0.3584 | 0.0304 | 0.0716 | 5.57E-07 | 25.06 | |
| IL-13 | rs12199215 | 6 | T | C | 0.1309 | 0.247 | 0.0284 | 4.04E-06 | 21.24 | |
| IL-13 | rs12623722 | 2 | A | G | -0.1189 | 0.3054 | 0.0257 | 3.72E-06 | 21.40 | |
| IL-13 | rs13206012 | 6 | A | G | -0.3719 | 0.3489 | 0.0263 | 2.13E-45 | 199.96 | |
| IL-13 | rs13209117 | 6 | A | G | 0.1409 | 0.245 | 0.0284 | 7.00E-07 | 24.61 | |
| IL-13 | rs138854806 | 6 | A | G | -0.4204 | 0.0247 | 0.0839 | 5.42E-07 | 25.11 | |
| IL-13 | rs139083458 | 5 | T | C | 0.9995 | 0.0034 | 0.211 | 2.17E-06 | 22.44 | |
| IL-13 | rs145023524 | 6 | A | G | 0.2815 | 0.0435 | 0.0588 | 1.69E-06 | 22.92 | |
| IL-13 | rs27949 | 5 | T | C | -0.1144 | 0.667 | 0.025 | 4.74E-06 | 20.94 | |
| IL-13 | rs28442067 | 3 | A | G | -0.1379 | 0.7832 | 0.0286 | 1.42E-06 | 23.25 | |
| IL-13 | rs7073807 | 10 | T | C | 0.1618 | 0.1351 | 0.0354 | 4.86E-06 | 20.89 | |
| IL-13 | rs76975337 | 3 | T | C | -0.1211 | 0.7286 | 0.0265 | 4.88E-06 | 20.88 | |
| IL-13 | rs7747448 | 6 | A | G | -0.1393 | 0.2585 | 0.0278 | 5.42E-07 | 25.11 | |
| IL-13 | rs7757246 | 6 | T | C | 0.2147 | 0.9092 | 0.0422 | 3.62E-07 | 25.88 | |
| IL-13 | rs77955971 | 6 | A | C | 0.4408 | 0.0247 | 0.0868 | 3.81E-07 | 25.79 | |
| IL-13 | rs9296421 | 6 | T | G | 0.1814 | 0.842 | 0.0348 | 1.86E-07 | 27.17 | |
| IL-16 | rs117217798 | 17 | T | C | -0.2064 | 0.0889 | 0.044 | 2.72E-06 | 22.00 | |
| IL-16 | rs12577604 | 11 | T | C | 0.4335 | 0.9818 | 0.0941 | 4.09E-06 | 21.22 | |
| IL-16 | rs142034902 | 12 | A | G | -0.4367 | 0.9827 | 0.0925 | 2.35E-06 | 22.29 | |
| IL-16 | rs144691581 | 15 | A | G | 0.4929 | 0.0197 | 0.0958 | 2.67E-07 | 26.47 | |
| IL-16 | rs35834666 | 4 | T | C | -0.1729 | 0.8596 | 0.0348 | 6.75E-07 | 24.68 | |
| IL-16 | rs4778640 | 15 | A | G | 0.7189 | 0.9848 | 0.0983 | 2.61E-13 | 53.48 | |
| IL-16 | rs4976691 | 5 | C | G | 0.1254 | 0.3268 | 0.026 | 1.41E-06 | 23.26 | |
| IL-16 | rs7097884 | 10 | T | C | -0.1193 | 0.5647 | 0.0243 | 9.13E-07 | 24.10 | |
| IL-16 | rs78042619 | 9 | A | G | 0.55 | 0.9883 | 0.1158 | 2.04E-06 | 22.56 | |
| IL-17 | rs11640734 | 16 | C | G | -0.115 | 0.8673 | 0.024 | 1.65E-06 | 22.96 | |
| IL-17 | rs11985957 | 8 | A | G | 0.1511 | 0.0642 | 0.0329 | 4.38E-06 | 21.09 | |
| IL-17 | rs12735700 | 1 | T | G | -0.0943 | 0.1925 | 0.0206 | 4.70E-06 | 20.96 | |
| IL-17 | rs148562661 | 6 | C | G | 0.2161 | 0.9605 | 0.0434 | 6.38E-07 | 24.79 | |
| IL-17 | rs17282552 | 2 | T | C | -0.2026 | 0.952 | 0.0403 | 4.97E-07 | 25.27 | |
| IL-17 | rs3804749 | 3 | T | C | -0.0923 | 0.6236 | 0.0167 | 3.26E-08 | 30.55 | |
| IL-17 | rs61990749 | 14 | C | G | 0.1124 | 0.1533 | 0.0226 | 6.58E-07 | 24.74 | |
| IL-17 | rs77341831 | 12 | T | C | 0.2263 | 0.9696 | 0.0473 | 1.72E-06 | 22.89 | |
| IL-17 | rs78296352 | 1 | T | G | 0.2949 | 0.0186 | 0.0645 | 4.83E-06 | 20.90 | |
| IL-18 | rs10409850 | 19 | A | G | 0.1791 | 0.8704 | 0.0347 | 2.45E-07 | 26.64 | |
| IL-18 | rs117266781 | 7 | T | C | 0.7051 | 0.0073 | 0.1436 | 9.10E-07 | 24.11 | |
| IL-18 | rs117371668 | 16 | T | G | 0.3712 | 0.0254 | 0.0799 | 3.39E-06 | 21.58 | |
| IL-18 | rs12419156 | 11 | T | C | 0.1556 | 0.8064 | 0.0335 | 3.40E-06 | 21.57 | |
| IL-18 | rs139727649 | 5 | T | C | -0.356 | 0.9745 | 0.0751 | 2.13E-06 | 22.47 | |
| IL-18 | rs1979967 | 15 | T | C | 0.14 | 0.2157 | 0.0285 | 9.00E-07 | 24.13 | |
| IL-18 | rs4952239 | 2 | A | T | -0.1156 | 0.3555 | 0.0242 | 1.78E-06 | 22.82 | |
| IL-18 | rs62312914 | 4 | T | C | -0.1265 | 0.3726 | 0.025 | 4.19E-07 | 25.60 | |
| IL-18 | rs7599125 | 2 | A | G | 0.1109 | 0.569 | 0.0239 | 3.48E-06 | 21.53 | |
| IL-18 | rs76138275 | 5 | T | C | 0.1227 | 0.2858 | 0.026 | 2.37E-06 | 22.27 | |
| IL-18 | rs764078 | 11 | A | T | 0.1283 | 0.2239 | 0.0278 | 3.93E-06 | 21.30 | |
| IL-18 | rs77187209 | 5 | T | C | -0.4859 | 0.9866 | 0.1041 | 3.05E-06 | 21.79 | |
| IL-18 | rs78623212 | 7 | T | C | 0.8322 | 0.0053 | 0.1676 | 6.86E-07 | 24.66 | |
| IL-18 | rs78716465 | 20 | A | G | 0.3173 | 0.033 | 0.0679 | 2.97E-06 | 21.84 | |
| IL-1B | rs143319329 | 7 | T | C | 0.4357 | 0.0209 | 0.093 | 2.80E-06 | 21.95 | |
| IL-1B | rs4786740 | 16 | A | C | 0.1264 | 0.3913 | 0.0265 | 1.84E-06 | 22.75 | |
| IL-1B | rs61335305 | 15 | A | C | 0.4333 | 0.0189 | 0.0928 | 3.02E-06 | 21.80 | |
| IL-1B | rs62015704 | 16 | A | G | 0.1786 | 0.8688 | 0.0372 | 1.58E-06 | 23.05 | |
| IL-1RA | rs1054402 | 9 | T | C | 0.1325 | 0.2506 | 0.0269 | 8.41E-07 | 24.26 | |
| IL-1RA | rs117181659 | 22 | A | G | -0.2204 | 0.064 | 0.0478 | 4.01E-06 | 21.26 | |
| IL-1RA | rs11869294 | 17 | C | G | -0.2286 | 0.9205 | 0.047 | 1.15E-06 | 23.66 | |
| IL-1RA | rs13343438 | 19 | A | G | 0.2771 | 0.0434 | 0.0607 | 4.99E-06 | 20.84 | |
| IL-1RA | rs3876037 | 22 | A | G | 0.1234 | 0.6783 | 0.027 | 4.87E-06 | 20.89 | |
| IL-1RA | rs56134659 | 3 | A | G | -0.1109 | 0.4835 | 0.0236 | 2.61E-06 | 22.08 | |
| IL-1RA | rs61335305 | 15 | A | C | 0.4315 | 0.0182 | 0.0904 | 1.81E-06 | 22.78 | |
| IL-1RA | rs6699436 | 1 | A | G | -0.1858 | 0.0995 | 0.0404 | 4.25E-06 | 21.15 | |
| IL-1RA | rs9623661 | 22 | T | C | -0.1948 | 0.0903 | 0.0424 | 4.34E-06 | 21.11 | |
| IL-1RA | rs9985296 | 3 | T | C | 0.1053 | 0.4758 | 0.0231 | 5.15E-06 | 20.78 | |
| IL-2 | rs13412535 | 2 | A | G | 0.174 | 0.1886 | 0.0331 | 1.47E-07 | 27.63 | |
| IL-2 | rs16836080 | 3 | A | G | 0.1158 | 0.3355 | 0.0253 | 4.72E-06 | 20.95 | |
| IL-2 | rs2690020 | 1 | A | G | 0.1158 | 0.5177 | 0.0245 | 2.28E-06 | 22.34 | |
| IL-2 | rs4479767 | 4 | A | G | 0.1821 | 0.8977 | 0.0392 | 3.39E-06 | 21.58 | |
| IL-2 | rs4634519 | 7 | A | G | -0.1249 | 0.7379 | 0.0268 | 3.16E-06 | 21.72 | |
| IL-2 | rs61335305 | 15 | A | C | 0.4439 | 0.0184 | 0.0913 | 1.16E-06 | 23.64 | |
| IL-2 | rs62124990 | 2 | T | G | -0.7013 | 0.0083 | 0.149 | 2.52E-06 | 22.15 | |
| IL-2 | rs7615304 | 3 | A | G | -0.1139 | 0.4531 | 0.024 | 2.08E-06 | 22.52 | |
| IL-2RA | rs11241559 | 5 | T | G | -0.124 | 0.2643 | 0.0264 | 2.64E-06 | 22.06 | |
| IL-2RA | rs115360066 | 5 | A | G | 0.1776 | 0.8882 | 0.0377 | 2.47E-06 | 22.19 | |
| IL-2RA | rs117244812 | 17 | A | G | -0.7187 | 0.0079 | 0.1493 | 1.48E-06 | 23.17 | |
| IL-2RA | rs12789243 | 11 | T | C | 0.1263 | 0.7571 | 0.0276 | 4.74E-06 | 20.94 | |
| IL-2RA | rs17624670 | 8 | A | G | -0.125 | 0.2531 | 0.0273 | 4.68E-06 | 20.96 | |
| IL-2RA | rs56213152 | 7 | T | C | 0.1269 | 0.7688 | 0.0271 | 2.83E-06 | 21.93 | |
| IL-2RA | rs7078614 | 10 | T | G | -0.1543 | 0.3549 | 0.0241 | 1.53E-10 | 40.99 | |
| IL-2RA | rs759244 | 4 | A | T | -0.1094 | 0.394 | 0.0238 | 4.29E-06 | 21.13 | |
| IL-2RA | rs79100208 | 3 | C | G | 0.8345 | 0.995 | 0.1758 | 2.07E-06 | 22.53 | |
| IL-2RA | rs9423654 | 10 | C | G | -0.135 | 0.6609 | 0.0283 | 1.84E-06 | 22.76 | |
| IL-4 | rs116705532 | 1 | T | G | -0.4675 | 0.9925 | 0.0978 | 1.75E-06 | 22.85 | |
| IL-4 | rs117146485 | 9 | T | C | -0.2856 | 0.9837 | 0.0625 | 4.89E-06 | 20.88 | |
| IL-4 | rs12640583 | 4 | T | G | -0.1104 | 0.1631 | 0.0214 | 2.48E-07 | 26.61 | |
| IL-4 | rs17713451 | 7 | A | G | 0.1255 | 0.1149 | 0.0252 | 6.35E-07 | 24.80 | |
| IL-4 | rs1867282 | 9 | T | C | 0.0808 | 0.4148 | 0.0162 | 6.11E-07 | 24.88 | |
| IL-4 | rs2073438 | 17 | A | G | 0.0847 | 0.2513 | 0.0183 | 3.68E-06 | 21.42 | |
| IL-4 | rs2346020 | 3 | A | G | 0.079 | 0.6795 | 0.0169 | 2.95E-06 | 21.85 | |
| IL-4 | rs2708586 | 7 | T | C | -0.0767 | 0.3411 | 0.0166 | 3.83E-06 | 21.35 | |
| IL-4 | rs7613691 | 3 | A | G | 0.1787 | 0.9482 | 0.0382 | 2.90E-06 | 21.88 | |
| IL-4 | rs79597994 | 1 | T | C | -0.5855 | 0.0049 | 0.1271 | 4.09E-06 | 21.22 | |
| IL-4 | rs9506111 | 13 | A | G | -0.1446 | 0.9326 | 0.0314 | 4.12E-06 | 21.21 | |
| IL-4 | rs9941733 | 20 | A | G | 0.1156 | 0.8355 | 0.0229 | 4.46E-07 | 25.48 | |
| IL-5 | rs10178043 | 2 | T | G | 0.2579 | 0.949 | 0.0553 | 3.11E-06 | 21.75 | |
| IL-5 | rs111736126 | 2 | C | G | -0.3973 | 0.9792 | 0.0867 | 4.60E-06 | 21.00 | |
| IL-5 | rs148634917 | 1 | A | G | -0.517 | 0.986 | 0.1087 | 1.97E-06 | 22.62 | |
| IL-5 | rs28793375 | 8 | T | C | 0.1697 | 0.1298 | 0.0362 | 2.76E-06 | 21.98 | |
| IL-5 | rs73040118 | 19 | T | C | 0.2294 | 0.9285 | 0.049 | 2.85E-06 | 21.92 | |
| IL-5 | rs74811276 | 14 | A | G | 0.217 | 0.0761 | 0.0471 | 4.08E-06 | 21.23 | |
| IL-5 | rs7739450 | 6 | A | G | -0.1295 | 0.5158 | 0.0256 | 4.22E-07 | 25.59 | |
| IL-5 | rs9309063 | 2 | T | G | -0.1119 | 0.4763 | 0.0245 | 4.94E-06 | 20.86 | |
| IL-6 | rs10910395 | 1 | A | T | -0.108 | 0.8729 | 0.0235 | 4.31E-06 | 21.12 | |
| IL-6 | rs10982193 | 9 | A | G | -0.0793 | 0.2822 | 0.0174 | 5.18E-06 | 20.77 | |
| IL-6 | rs114373846 | 3 | T | C | 0.4196 | 0.0085 | 0.0905 | 3.54E-06 | 21.50 | |
| IL-6 | rs11732981 | 4 | A | C | 0.0722 | 0.5119 | 0.0156 | 3.69E-06 | 21.42 | |
| IL-6 | rs1333040 | 9 | T | C | 0.0747 | 0.4508 | 0.0157 | 1.96E-06 | 22.64 | |
| IL-6 | rs13412535 | 2 | A | G | -0.1186 | 0.189 | 0.0214 | 2.99E-08 | 30.71 | |
| IL-6 | rs4684700 | 3 | T | C | -0.0747 | 0.5142 | 0.0162 | 4.01E-06 | 21.26 | |
| IL-6 | rs73273528 | 20 | T | C | 0.268 | 0.0211 | 0.0553 | 1.26E-06 | 23.49 | |
| IL-6 | rs76856708 | 16 | T | C | 0.336 | 0.9856 | 0.0697 | 1.43E-06 | 23.24 | |
| IL-7 | rs115215018 | 4 | T | C | 0.5985 | 0.0101 | 0.1308 | 4.75E-06 | 20.94 | |
| IL-7 | rs11757972 | 6 | T | C | 0.121 | 0.4776 | 0.0257 | 2.50E-06 | 22.17 | |
| IL-7 | rs1374279 | 2 | A | T | 0.1625 | 0.143 | 0.0347 | 2.83E-06 | 21.93 | |
| IL-7 | rs142397827 | 5 | A | C | 0.4592 | 0.0178 | 0.0994 | 3.84E-06 | 21.34 | |
| IL-7 | rs17091524 | 14 | T | C | 0.5092 | 0.9849 | 0.1015 | 5.26E-07 | 25.17 | |
| IL-7 | rs218238 | 4 | A | T | 0.1319 | 0.7729 | 0.0284 | 3.41E-06 | 21.57 | |
| IL-7 | rs28793375 | 8 | T | C | 0.1644 | 0.1305 | 0.036 | 4.96E-06 | 20.85 | |
| IL-7 | rs62006410 | 14 | T | C | -0.1492 | 0.245 | 0.0302 | 7.80E-07 | 24.41 | |
| IL-7 | rs7155170 | 14 | A | T | -0.1236 | 0.2744 | 0.027 | 4.70E-06 | 20.96 | |
| IL-7 | rs77318030 | 19 | T | C | -0.2966 | 0.9574 | 0.0631 | 2.60E-06 | 22.09 | |
| IL-7 | rs7739450 | 6 | A | G | -0.2907 | 0.5167 | 0.0252 | 8.72E-31 | 133.07 | |
| IL-7 | rs77981494 | 16 | T | C | -0.5201 | 0.984 | 0.1055 | 8.23E-07 | 24.30 | |
| IL-7 | rs78346957 | 10 | A | G | 0.4632 | 0.0161 | 0.1008 | 4.32E-06 | 21.12 | |
| IL-8 | rs113487695 | 7 | A | C | -0.6129 | 0.9914 | 0.1292 | 2.10E-06 | 22.50 | |
| IL-8 | rs116726256 | 2 | T | C | -0.2247 | 0.9343 | 0.0489 | 4.33E-06 | 21.11 | |
| IL-8 | rs12075 | 1 | A | G | 0.1148 | 0.5353 | 0.0235 | 1.03E-06 | 23.86 | |
| IL-8 | rs12912642 | 15 | A | G | 0.1168 | 0.3302 | 0.0251 | 3.27E-06 | 21.65 | |
| IL-8 | rs183628733 | 1 | T | C | 0.6547 | 0.9917 | 0.1417 | 3.83E-06 | 21.35 | |
| IL-8 | rs2673604 | 8 | A | C | -0.118 | 0.6829 | 0.0254 | 3.39E-06 | 21.58 | |
| IL-8 | rs3786107 | 17 | A | G | 0.2463 | 0.9287 | 0.0517 | 1.90E-06 | 22.70 | |
| IL-8 | rs75840288 | 16 | A | C | 0.5125 | 0.9839 | 0.1121 | 4.84E-06 | 20.90 | |
| IL-9 | rs1259728 | 12 | A | G | -0.2381 | 0.0554 | 0.0507 | 2.65E-06 | 22.05 | |
| IL-9 | rs3736858 | 13 | C | G | -0.1351 | 0.7976 | 0.0291 | 3.44E-06 | 21.55 | |
| IL-9 | rs41294750 | 1 | T | C | 0.3442 | 0.0308 | 0.0736 | 2.92E-06 | 21.87 | |
| IL-9 | rs4880409 | 10 | T | C | -0.3552 | 0.9664 | 0.0716 | 7.02E-07 | 24.61 | |
| IL-9 | rs73443903 | 6 | A | C | 0.2162 | 0.0685 | 0.046 | 2.60E-06 | 22.09 | |
| IP-10 | rs12714300 | 2 | A | T | -0.1573 | 0.1378 | 0.0338 | 3.26E-06 | 21.66 | |
| IP-10 | rs143799975 | 4 | A | G | -0.7551 | 0.9949 | 0.1638 | 4.03E-06 | 21.25 | |
| IP-10 | rs34383175 | 8 | T | C | -0.3196 | 0.035 | 0.0653 | 9.86E-07 | 23.95 | |
| IP-10 | rs397816 | 22 | T | C | 0.1211 | 0.5891 | 0.0248 | 1.04E-06 | 23.84 | |
| IP-10 | rs4859940 | 4 | C | G | -0.1204 | 0.295 | 0.0258 | 3.06E-06 | 21.78 | |
| IP-10 | rs4862110 | 4 | T | C | -0.1453 | 0.771 | 0.0318 | 4.90E-06 | 20.88 | |
| IP-10 | rs7645625 | 3 | T | G | -0.1116 | 0.5724 | 0.0236 | 2.26E-06 | 22.36 | |
| IP-10 | rs79848609 | 15 | A | C | 0.2514 | 0.9449 | 0.0535 | 2.61E-06 | 22.08 | |
| IP-10 | rs8112618 | 19 | A | G | 0.1388 | 0.1841 | 0.0297 | 2.96E-06 | 21.84 | |
| M-CSF | rs116887628 | 8 | A | G | -0.2741 | 0.0611 | 0.0598 | 4.57E-06 | 21.01 | |
| M-CSF | rs117867915 | 18 | T | C | 0.5224 | 0.9793 | 0.1096 | 1.88E-06 | 22.72 | |
| M-CSF | rs11963606 | 6 | C | G | -0.5353 | 0.9826 | 0.117 | 4.76E-06 | 20.93 | |
| M-CSF | rs12962919 | 18 | T | C | 0.3025 | 0.0644 | 0.0659 | 4.43E-06 | 21.07 | |
| M-CSF | rs139457375 | 8 | A | C | -0.4047 | 0.9668 | 0.0854 | 2.15E-06 | 22.46 | |
| M-CSF | rs147378920 | 1 | A | G | -0.6064 | 0.9871 | 0.1318 | 4.21E-06 | 21.17 | |
| M-CSF | rs34089869 | 2 | T | C | 0.2194 | 0.1039 | 0.0462 | 2.05E-06 | 22.55 | |
| M-CSF | rs62294910 | 3 | A | G | 0.3472 | 0.0504 | 0.0687 | 4.33E-07 | 25.54 | |
| M-CSF | rs72723242 | 5 | T | G | -0.4969 | 0.0177 | 0.1083 | 4.47E-06 | 21.05 | |
| M-CSF | rs9387100 | 6 | T | C | -0.135 | 0.4291 | 0.029 | 3.24E-06 | 21.67 | |
| M-CSF | rs9626985 | 22 | T | C | 0.2277 | 0.0936 | 0.0496 | 4.42E-06 | 21.07 | |
| MCP-1-MCAF | rs111995966 | 2 | T | G | 0.1428 | 0.9297 | 0.0309 | 3.81E-06 | 21.36 | |
| MCP-1-MCAF | rs112313229 | 3 | A | G | -0.1652 | 0.0708 | 0.0312 | 1.19E-07 | 28.04 | |
| MCP-1-MCAF | rs11920996 | 3 | T | C | 0.1805 | 0.051 | 0.0376 | 1.58E-06 | 23.05 | |
| MCP-1-MCAF | rs12062235 | 1 | T | G | 0.1477 | 0.9377 | 0.032 | 3.92E-06 | 21.30 | |
| MCP-1-MCAF | rs12493953 | 3 | A | G | -0.0948 | 0.3224 | 0.0172 | 3.56E-08 | 30.38 | |
| MCP-1-MCAF | rs145155829 | 1 | T | C | -0.2125 | 0.037 | 0.0461 | 4.04E-06 | 21.25 | |
| MCP-1-MCAF | rs16837903 | 1 | A | G | -0.1104 | 0.1237 | 0.0238 | 3.51E-06 | 21.52 | |
| MCP-1-MCAF | rs2229593 | 3 | T | C | 0.2624 | 0.0385 | 0.0405 | 9.23E-11 | 41.98 | |
| MCP-1-MCAF | rs2820126 | 1 | T | G | 0.0907 | 0.7898 | 0.0193 | 2.61E-06 | 22.09 | |
| MCP-1-MCAF | rs3026968 | 1 | T | C | -0.0896 | 0.3195 | 0.0169 | 1.15E-07 | 28.11 | |
| MCP-1-MCAF | rs34190208 | 3 | T | C | 0.1052 | 0.1511 | 0.0219 | 1.56E-06 | 23.08 | |
| MCP-1-MCAF | rs56212190 | 1 | T | C | 0.1799 | 0.0519 | 0.0372 | 1.32E-06 | 23.39 | |
| MCP-1-MCAF | rs7197349 | 16 | A | G | 0.0971 | 0.8124 | 0.0206 | 2.43E-06 | 22.22 | |
| MCP-1-MCAF | rs72705803 | 9 | A | G | -0.2188 | 0.9694 | 0.047 | 3.23E-06 | 21.67 | |
| MCP-1-MCAF | rs77116118 | 3 | T | C | -0.4239 | 0.9892 | 0.0824 | 2.68E-07 | 26.47 | |
| MCP-1-MCAF | rs7978037 | 12 | A | T | 0.0746 | 0.3863 | 0.016 | 3.12E-06 | 21.74 | |
| MCP-1-MCAF | rs79939301 | 3 | A | G | 0.1449 | 0.1081 | 0.0255 | 1.33E-08 | 32.29 | |
| MCP-1-MCAF | rs80108502 | 3 | T | C | 0.2463 | 0.0391 | 0.0409 | 1.72E-09 | 36.26 | |
| MCP-1-MCAF | rs863002 | 1 | T | C | 0.1135 | 0.4077 | 0.0158 | 6.79E-13 | 51.60 | |
| MCP-1-MCAF | rs9317045 | 13 | A | C | 0.1157 | 0.8618 | 0.0235 | 8.51E-07 | 24.24 | |
| MCP-3 | rs10892381 | 11 | T | C | 0.2432 | 0.6629 | 0.0473 | 2.72E-07 | 26.44 | |
| MCP-3 | rs117286643 | 8 | A | G | 0.6934 | 0.0225 | 0.1474 | 2.55E-06 | 22.13 | |
| MCP-3 | rs28394764 | 4 | A | T | 0.597 | 0.9696 | 0.1282 | 3.21E-06 | 21.69 | |
| MCP-3 | rs3129806 | 9 | T | C | -0.1975 | 0.5729 | 0.0433 | 5.09E-06 | 20.80 | |
| MCP-3 | rs6993671 | 8 | T | C | 0.2041 | 0.5588 | 0.0443 | 4.08E-06 | 21.23 | |
| MCP-3 | rs7275485 | 21 | T | C | -0.2218 | 0.2711 | 0.0481 | 4.00E-06 | 21.26 | |
| MIF | rs1007888 | 22 | T | C | -0.1275 | 0.6128 | 0.0245 | 1.95E-07 | 27.08 | |
| MIF | rs11551183 | 16 | C | G | 0.3666 | 0.9756 | 0.0795 | 4.00E-06 | 21.26 | |
| MIF | rs12594190 | 15 | A | G | 0.1321 | 0.7008 | 0.0266 | 6.83E-07 | 24.66 | |
| MIF | rs141009259 | 2 | T | C | -0.6194 | 0.9886 | 0.1285 | 1.43E-06 | 23.23 | |
| MIF | rs35792361 | 4 | A | G | -0.2586 | 0.0591 | 0.0527 | 9.25E-07 | 24.08 | |
| MIF | rs35890933 | 19 | T | G | 0.1676 | 0.8474 | 0.0365 | 4.39E-06 | 21.08 | |
| MIF | rs3814097 | 7 | A | G | -0.1163 | 0.5533 | 0.0251 | 3.60E-06 | 21.47 | |
| MIF | rs78098071 | 5 | T | C | -0.4583 | 0.9809 | 0.0915 | 5.48E-07 | 25.09 | |
| MIG | rs10266753 | 7 | T | C | -0.2016 | 0.9109 | 0.0397 | 3.81E-07 | 25.79 | |
| MIG | rs111607343 | 19 | A | G | -0.5235 | 0.0134 | 0.1119 | 2.89E-06 | 21.89 | |
| MIG | rs11177248 | 12 | A | G | 0.3157 | 0.0351 | 0.0667 | 2.21E-06 | 22.40 | |
| MIG | rs113302091 | 14 | T | C | 0.2537 | 0.0468 | 0.0553 | 4.48E-06 | 21.05 | |
| MIG | rs139010077 | 3 | T | C | 0.4337 | 0.0169 | 0.0943 | 4.24E-06 | 21.15 | |
| MIG | rs192433162 | 10 | A | G | -0.8045 | 0.0061 | 0.1676 | 1.59E-06 | 23.04 | |
| MIG | rs3733233 | 4 | T | C | 0.1223 | 0.6588 | 0.025 | 9.98E-07 | 23.93 | |
| MIG | rs62562991 | 9 | A | G | 0.6239 | 0.0097 | 0.1259 | 7.21E-07 | 24.56 | |
| MIG | rs8127917 | 21 | T | G | 0.2382 | 0.0608 | 0.0492 | 1.29E-06 | 23.44 | |
| MIG | rs816960 | 13 | T | C | -0.1179 | 0.3692 | 0.0242 | 1.11E-06 | 23.74 | |
| MIG | rs9456663 | 6 | T | C | -0.1186 | 0.6702 | 0.0255 | 3.30E-06 | 21.63 | |
| MIP-1A | rs116615337 | 1 | A | G | 0.1286 | 0.4232 | 0.0278 | 3.73E-06 | 21.40 | |
| MIP-1A | rs117506943 | 11 | T | C | 0.3128 | 0.0323 | 0.0682 | 4.51E-06 | 21.04 | |
| MIP-1A | rs12159394 | 22 | A | G | -0.1708 | 0.1183 | 0.0366 | 3.06E-06 | 21.78 | |
| MIP-1A | rs57786342 | 14 | A | G | 0.139 | 0.2336 | 0.0283 | 9.03E-07 | 24.12 | |
| MIP-1A | rs6900267 | 6 | A | C | -0.2472 | 0.9308 | 0.0515 | 1.59E-06 | 23.04 | |
| MIP-1A | rs6956239 | 7 | T | C | 0.119 | 0.2927 | 0.026 | 4.72E-06 | 20.95 | |
| MIP-1B | rs114933663 | 3 | T | C | 0.3178 | 0.0694 | 0.0307 | 4.11E-25 | 107.16 | |
| MIP-1B | rs116237296 | 1 | A | G | 0.5284 | 0.0054 | 0.1115 | 2.15E-06 | 22.46 | |
| MIP-1B | rs117010890 | 17 | T | C | -0.2043 | 0.9347 | 0.0393 | 2.01E-07 | 27.02 | |
| MIP-1B | rs11716293 | 3 | C | G | 0.0986 | 0.7698 | 0.0189 | 1.82E-07 | 27.22 | |
| MIP-1B | rs117453826 | 17 | A | G | -0.5907 | 0.981 | 0.0591 | 1.60E-23 | 99.90 | |
| MIP-1B | rs12452320 | 17 | A | C | 0.2139 | 0.0716 | 0.0319 | 2.01E-11 | 44.96 | |
| MIP-1B | rs12951603 | 17 | A | G | -0.1132 | 0.1492 | 0.0225 | 4.88E-07 | 25.31 | |
| MIP-1B | rs141793738 | 17 | A | G | 0.1815 | 0.0432 | 0.0389 | 3.07E-06 | 21.77 | |
| MIP-1B | rs1437220 | 17 | T | C | 0.1437 | 0.9207 | 0.0315 | 5.07E-06 | 20.81 | |
| MIP-1B | rs145526037 | 3 | T | G | -0.1863 | 0.9604 | 0.0406 | 4.46E-06 | 21.06 | |
| MIP-1B | rs146565944 | 17 | T | C | 0.2863 | 0.0235 | 0.0558 | 2.88E-07 | 26.33 | |
| MIP-1B | rs148561432 | 17 | A | G | -0.2691 | 0.0454 | 0.0407 | 3.80E-11 | 43.72 | |
| MIP-1B | rs1543292 | 17 | A | G | 0.2627 | 0.0632 | 0.0338 | 7.71E-15 | 60.41 | |
| MIP-1B | rs159309 | 17 | T | C | 0.1254 | 0.1102 | 0.0258 | 1.17E-06 | 23.62 | |
| MIP-1B | rs17138331 | 7 | A | G | -0.1434 | 0.9092 | 0.0295 | 1.17E-06 | 23.63 | |
| MIP-1B | rs1867288 | 17 | C | G | 0.2034 | 0.2288 | 0.0215 | 3.07E-21 | 89.50 | |
| MIP-1B | rs191600590 | 3 | A | T | 0.1449 | 0.1326 | 0.0246 | 3.86E-09 | 34.69 | |
| MIP-1B | rs2131092 | 3 | A | G | -0.1278 | 0.1103 | 0.0248 | 2.56E-07 | 26.56 | |
| MIP-1B | rs2276857 | 3 | T | C | -0.1283 | 0.1356 | 0.0257 | 5.97E-07 | 24.92 | |
| MIP-1B | rs2314809 | 17 | T | C | -0.0735 | 0.5305 | 0.0157 | 2.85E-06 | 21.92 | |
| MIP-1B | rs2373048 | 3 | A | T | -0.116 | 0.1789 | 0.0215 | 6.84E-08 | 29.11 | |
| MIP-1B | rs2376263 | 17 | A | G | 0.1053 | 0.7795 | 0.0187 | 1.79E-08 | 31.71 | |
| MIP-1B | rs2411161 | 17 | T | C | 0.1719 | 0.9508 | 0.0365 | 2.48E-06 | 22.18 | |
| MIP-1B | rs2673059 | 3 | T | C | 0.0921 | 0.7787 | 0.0192 | 1.61E-06 | 23.01 | |
| MIP-1B | rs281728 | 8 | A | C | -0.079 | 0.7058 | 0.0171 | 3.84E-06 | 21.34 | |
| MIP-1B | rs28393318 | 4 | A | G | -0.1076 | 0.8758 | 0.0235 | 4.68E-06 | 20.96 | |
| MIP-1B | rs28856610 | 17 | T | C | -0.3074 | 0.031 | 0.0495 | 5.30E-10 | 38.57 | |
| MIP-1B | rs323877 | 3 | C | G | -0.0965 | 0.6443 | 0.0172 | 2.02E-08 | 31.48 | |
| MIP-1B | rs34437725 | 17 | T | C | -0.2571 | 0.9697 | 0.0481 | 9.04E-08 | 28.57 | |
| MIP-1B | rs35933743 | 17 | T | G | -0.1183 | 0.8438 | 0.0238 | 6.68E-07 | 24.71 | |
| MIP-1B | rs41290648 | 3 | A | G | 0.2231 | 0.1266 | 0.0239 | 1.01E-20 | 87.14 | |
| MIP-1B | rs41341749 | 17 | A | G | -0.1656 | 0.9203 | 0.0294 | 1.77E-08 | 31.73 | |
| MIP-1B | rs41502550 | 3 | T | C | 0.1266 | 0.8499 | 0.022 | 8.69E-09 | 33.11 | |
| MIP-1B | rs4795931 | 17 | A | G | -0.0865 | 0.3193 | 0.0173 | 5.73E-07 | 25.00 | |
| MIP-1B | rs4796110 | 17 | A | G | 0.1244 | 0.8879 | 0.0256 | 1.18E-06 | 23.61 | |
| MIP-1B | rs56083628 | 17 | T | C | -0.1297 | 0.1054 | 0.0257 | 4.50E-07 | 25.47 | |
| MIP-1B | rs62079535 | 17 | A | G | 0.2309 | 0.9572 | 0.0389 | 2.93E-09 | 35.23 | |
| MIP-1B | rs6908843 | 6 | A | G | 0.0997 | 0.1684 | 0.0209 | 1.84E-06 | 22.76 | |
| MIP-1B | rs71381491 | 17 | A | C | 0.3777 | 0.9746 | 0.0561 | 1.67E-11 | 45.33 | |
| MIP-1B | rs72791296 | 5 | T | C | 0.2364 | 0.0349 | 0.0466 | 3.92E-07 | 25.73 | |
| MIP-1B | rs72799710 | 5 | T | C | -0.1037 | 0.1514 | 0.0217 | 1.76E-06 | 22.84 | |
| MIP-1B | rs72820112 | 17 | T | C | 0.1059 | 0.7412 | 0.0183 | 7.17E-09 | 33.49 | |
| MIP-1B | rs72820246 | 17 | T | G | -0.0983 | 0.4163 | 0.0167 | 3.95E-09 | 34.65 | |
| MIP-1B | rs72825991 | 17 | A | G | -0.179 | 0.0709 | 0.0312 | 9.63E-09 | 32.92 | |
| MIP-1B | rs72829264 | 17 | A | G | -0.1517 | 0.8833 | 0.0277 | 4.34E-08 | 29.99 | |
| MIP-1B | rs74979864 | 7 | A | T | -0.3184 | 0.0169 | 0.0613 | 2.06E-07 | 26.98 | |
| MIP-1B | rs76582507 | 9 | A | G | 0.3259 | 0.0156 | 0.0676 | 1.43E-06 | 23.24 | |
| MIP-1B | rs76842834 | 17 | T | C | -0.4207 | 0.0371 | 0.0471 | 4.18E-19 | 79.78 | |
| MIP-1B | rs76863419 | 17 | T | G | -0.2833 | 0.073 | 0.0341 | 9.74E-17 | 69.02 | |
| MIP-1B | rs8081726 | 17 | T | C | -0.3499 | 0.0491 | 0.036 | 2.49E-22 | 94.47 | |
| MIP-1B | rs854222 | 3 | A | C | 0.0809 | 0.2696 | 0.0177 | 4.86E-06 | 20.89 | |
| MIP-1B | rs873944 | 17 | T | C | 0.2392 | 0.0726 | 0.0322 | 1.10E-13 | 55.18 | |
| MIP-1B | rs939408 | 3 | A | C | -0.1007 | 0.3938 | 0.016 | 3.10E-10 | 39.61 | |
| MIP-1B | rs951814 | 17 | A | G | 0.1614 | 0.07 | 0.0315 | 2.99E-07 | 26.25 | |
| MIP-1B | rs9793308 | 1 | A | G | 0.0842 | 0.6036 | 0.0177 | 1.96E-06 | 22.63 | |
| MIP-1B | rs9838883 | 3 | T | C | 0.079 | 0.3399 | 0.0166 | 1.95E-06 | 22.65 | |
| MIP-1B | rs9911839 | 17 | T | G | -0.1377 | 0.9011 | 0.027 | 3.40E-07 | 26.01 | |
| MIP-1B | rs9914803 | 17 | T | C | 0.098 | 0.5189 | 0.0156 | 3.34E-10 | 39.46 | |
| PDGF-BB | rs10512952 | 5 | T | C | -0.2816 | 0.9819 | 0.0587 | 1.61E-06 | 23.01 | |
| PDGF-BB | rs11766649 | 7 | A | G | 0.0902 | 0.8018 | 0.0196 | 4.18E-06 | 21.18 | |
| PDGF-BB | rs12289510 | 11 | A | G | -0.0772 | 0.4767 | 0.0158 | 1.03E-06 | 23.87 | |
| PDGF-BB | rs12615784 | 2 | T | C | -0.1003 | 0.7849 | 0.0193 | 2.03E-07 | 27.01 | |
| PDGF-BB | rs13037046 | 20 | A | T | -0.0948 | 0.1871 | 0.0206 | 4.19E-06 | 21.18 | |
| PDGF-BB | rs2643354 | 15 | A | G | 0.1251 | 0.8921 | 0.0261 | 1.64E-06 | 22.97 | |
| PDGF-BB | rs62191444 | 20 | T | G | -0.112 | 0.1492 | 0.0239 | 2.78E-06 | 21.96 | |
| PDGF-BB | rs6910518 | 6 | T | G | 0.0806 | 0.5924 | 0.0162 | 6.51E-07 | 24.75 | |
| PDGF-BB | rs7170232 | 15 | T | C | 0.1609 | 0.219 | 0.0189 | 1.69E-17 | 72.48 | |
| PDGF-BB | rs73162807 | 3 | A | C | -0.2313 | 0.0259 | 0.0499 | 3.56E-06 | 21.49 | |
| RANTES | rs10505135 | 8 | T | C | 0.1315 | 0.3704 | 0.0252 | 1.81E-07 | 27.23 | |
| RANTES | rs118096511 | 13 | T | C | -0.3374 | 0.9696 | 0.0709 | 1.95E-06 | 22.65 | |
| RANTES | rs11873385 | 18 | A | G | -0.2582 | 0.9466 | 0.0552 | 2.90E-06 | 21.88 | |
| RANTES | rs148526102 | 19 | T | C | -0.3798 | 0.0215 | 0.083 | 4.74E-06 | 20.94 | |
| RANTES | rs2731672 | 5 | T | C | -0.1242 | 0.267 | 0.0272 | 4.97E-06 | 20.85 | |
| RANTES | rs4795087 | 17 | C | G | 0.1494 | 0.8188 | 0.0312 | 1.68E-06 | 22.93 | |
| RANTES | rs62438851 | 6 | A | G | -0.1904 | 0.8961 | 0.0413 | 4.02E-06 | 21.25 | |
| RANTES | rs7170339 | 15 | C | G | -0.4283 | 0.0197 | 0.0904 | 2.16E-06 | 22.45 | |
| RANTES | rs72793342 | 16 | A | G | -0.1505 | 0.2003 | 0.0307 | 9.47E-07 | 24.03 | |
| RANTES | rs78050316 | 2 | A | C | 0.4202 | 0.0204 | 0.0859 | 1.00E-06 | 23.93 | |
| SCF | rs10800449 | 1 | A | C | 0.0851 | 0.2859 | 0.0179 | 1.99E-06 | 22.60 | |
| SCF | rs11244035 | 9 | T | C | -0.1296 | 0.0944 | 0.0279 | 3.40E-06 | 21.58 | |
| SCF | rs113127926 | 14 | A | C | 0.1974 | 0.0418 | 0.0418 | 2.33E-06 | 22.30 | |
| SCF | rs12345108 | 9 | T | C | -0.0772 | 0.3395 | 0.0167 | 3.79E-06 | 21.37 | |
| SCF | rs13412535 | 2 | A | G | -0.1065 | 0.1892 | 0.0213 | 5.73E-07 | 25.00 | |
| SCF | rs138538809 | 8 | T | C | -0.5788 | 0.0052 | 0.1139 | 3.74E-07 | 25.82 | |
| SCF | rs1536480 | 9 | T | C | 0.081 | 0.3204 | 0.0167 | 1.23E-06 | 23.53 | |
| SCF | rs72678285 | 14 | A | T | 0.1062 | 0.8512 | 0.0231 | 4.28E-06 | 21.14 | |
| SCF | rs72832071 | 16 | A | G | 0.2238 | 0.9732 | 0.0482 | 3.43E-06 | 21.56 | |
| SCF | rs78666213 | 4 | T | G | -0.2845 | 0.9797 | 0.0574 | 7.18E-07 | 24.57 | |
| SCF | rs8045376 | 16 | A | G | -0.3126 | 0.0144 | 0.068 | 4.28E-06 | 21.13 | |
| SCGF-B | rs112346514 | 19 | T | C | -0.3261 | 0.0302 | 0.0703 | 3.51E-06 | 21.52 | |
| SCGF-B | rs1149926 | 10 | T | C | -0.3458 | 0.0239 | 0.0749 | 3.90E-06 | 21.32 | |
| SCGF-B | rs118003677 | 12 | T | C | -0.3654 | 0.9775 | 0.0786 | 3.34E-06 | 21.61 | |
| SCGF-B | rs12118918 | 1 | A | G | -0.1631 | 0.8669 | 0.035 | 3.16E-06 | 21.72 | |
| SCGF-B | rs12480722 | 20 | T | C | 0.1654 | 0.8752 | 0.0353 | 2.79E-06 | 21.95 | |
| SCGF-B | rs13287050 | 9 | A | T | -0.121 | 0.7202 | 0.0263 | 4.21E-06 | 21.17 | |
| SCGF-B | rs13866 | 19 | T | C | -0.1647 | 0.2622 | 0.028 | 4.05E-09 | 34.60 | |
| SCGF-B | rs139413256 | 7 | A | G | -0.5174 | 0.0139 | 0.1076 | 1.52E-06 | 23.12 | |
| SCGF-B | rs143829871 | 3 | T | C | -0.1866 | 0.9047 | 0.0399 | 2.92E-06 | 21.87 | |
| SCGF-B | rs149009264 | 10 | A | G | 0.4551 | 0.9848 | 0.0985 | 3.83E-06 | 21.35 | |
| SCGF-B | rs150733161 | 13 | T | C | -0.5255 | 0.0142 | 0.112 | 2.71E-06 | 22.01 | |
| SCGF-B | rs151194174 | 7 | A | G | 0.4536 | 0.0187 | 0.0941 | 1.43E-06 | 23.24 | |
| SCGF-B | rs264157 | 18 | A | G | 0.1079 | 0.4743 | 0.0233 | 3.64E-06 | 21.45 | |
| SCGF-B | rs3817303 | 12 | T | G | 0.1362 | 0.194 | 0.0294 | 3.61E-06 | 21.46 | |
| SCGF-B | rs4737731 | 8 | T | C | 0.1146 | 0.3019 | 0.0251 | 4.98E-06 | 20.85 | |
| SCGF-B | rs4976691 | 5 | C | G | -0.1484 | 0.3257 | 0.0253 | 4.47E-09 | 34.41 | |
| SCGF-B | rs5742627 | 12 | T | C | 0.2625 | 0.045 | 0.0574 | 4.80E-06 | 20.91 | |
| SCGF-B | rs77247938 | 12 | A | G | 0.2862 | 0.0655 | 0.0475 | 1.69E-09 | 36.30 | |
| SCGF-B | rs77954165 | 9 | T | C | 0.2631 | 0.9528 | 0.0562 | 2.85E-06 | 21.92 | |
| SCGF-B | rs7802293 | 7 | T | C | -0.1145 | 0.3779 | 0.0247 | 3.56E-06 | 21.49 | |
| SCGF-B | rs78217154 | 8 | T | C | 0.3942 | 0.9796 | 0.0861 | 4.69E-06 | 20.96 | |
| SDF-1A | rs10474392 | 5 | A | G | 0.0934 | 0.334 | 0.0177 | 1.31E-07 | 27.84 | |
| SDF-1A | rs10516368 | 4 | A | C | -0.4268 | 0.0105 | 0.0883 | 1.34E-06 | 23.36 | |
| SDF-1A | rs12141941 | 1 | T | C | -0.0881 | 0.7493 | 0.0186 | 2.17E-06 | 22.43 | |
| SDF-1A | rs1600396 | 4 | A | G | -0.0933 | 0.8096 | 0.0204 | 4.80E-06 | 20.92 | |
| SDF-1A | rs62194946 | 2 | T | G | -0.0849 | 0.2628 | 0.0185 | 4.45E-06 | 21.06 | |
| SDF-1A | rs6586903 | 8 | T | C | -0.1264 | 0.1037 | 0.0268 | 2.40E-06 | 22.24 | |
| SDF-1A | rs76766406 | 4 | A | G | 0.4642 | 0.9935 | 0.1012 | 4.50E-06 | 21.04 | |
| TNF-A | rs10767536 | 11 | A | G | 0.118 | 0.3274 | 0.0253 | 3.10E-06 | 21.75 | |
| TNF-A | rs115018697 | 4 | C | G | -0.9542 | 0.9962 | 0.197 | 1.27E-06 | 23.46 | |
| TNF-A | rs116736594 | 5 | T | C | 0.3407 | 0.0312 | 0.0702 | 1.21E-06 | 23.55 | |
| TNF-A | rs79105320 | 8 | A | G | 0.5573 | 0.0112 | 0.1177 | 2.19E-06 | 22.42 | |
| TNF-B | rs10925040 | 1 | T | C | 0.1738 | 0.371 | 0.0372 | 2.98E-06 | 21.83 | |
| TNF-B | rs143259067 | 1 | T | C | -0.6923 | 0.9595 | 0.1003 | 5.12E-12 | 47.64 | |
| TNF-B | rs2420873 | 19 | T | G | 0.1673 | 0.3897 | 0.0365 | 4.57E-06 | 21.01 | |
| TNF-B | rs62284710 | 3 | A | G | 0.3702 | 0.9443 | 0.0782 | 2.20E-06 | 22.41 | |
| TNF-B | rs75240021 | 8 | C | G | 0.3713 | 0.071 | 0.0772 | 1.51E-06 | 23.13 | |
| TNF-B | rs76225863 | 1 | A | G | 0.7534 | 0.0293 | 0.1217 | 5.99E-10 | 38.32 | |
| TRAIL | rs10084050 | 18 | A | G | -0.1101 | 0.8647 | 0.023 | 1.69E-06 | 22.91 | |
| TRAIL | rs10164260 | 18 | A | G | 0.1003 | 0.1741 | 0.0211 | 2.00E-06 | 22.60 | |
| TRAIL | rs11081739 | 18 | A | G | 0.1395 | 0.2048 | 0.0202 | 4.99E-12 | 47.69 | |
| TRAIL | rs112821861 | 18 | T | G | -0.8566 | 0.9717 | 0.0494 | 2.34E-67 | 300.68 | |
| TRAIL | rs113057689 | 3 | A | G | -0.2625 | 0.033 | 0.0489 | 7.96E-08 | 28.82 | |
| TRAIL | rs11875481 | 18 | T | C | -0.0969 | 0.835 | 0.0211 | 4.38E-06 | 21.09 | |
| TRAIL | rs13115587 | 4 | A | C | 0.101 | 0.1558 | 0.0217 | 3.25E-06 | 21.66 | |
| TRAIL | rs13278062 | 8 | T | G | 0.08 | 0.5589 | 0.0157 | 3.48E-07 | 25.96 | |
| TRAIL | rs139958028 | 11 | A | G | 0.1803 | 0.0501 | 0.0395 | 5.01E-06 | 20.84 | |
| TRAIL | rs146827832 | 3 | T | C | 0.1341 | 0.9038 | 0.0291 | 4.06E-06 | 21.24 | |
| TRAIL | rs17535790 | 3 | A | G | -0.1125 | 0.1498 | 0.0218 | 2.46E-07 | 26.63 | |
| TRAIL | rs550057 | 9 | T | C | -0.0783 | 0.3049 | 0.0169 | 3.60E-06 | 21.47 | |
| TRAIL | rs558572 | 3 | T | C | 0.1351 | 0.0949 | 0.0265 | 3.43E-07 | 25.99 | |
| TRAIL | rs57396456 | 18 | T | C | -0.5641 | 0.9755 | 0.0516 | 8.09E-28 | 119.51 | |
| TRAIL | rs62093482 | 18 | T | C | 0.9827 | 0.0237 | 0.0529 | 4.97E-77 | 345.09 | |
| TRAIL | rs7233927 | 18 | A | G | 0.0905 | 0.6648 | 0.0164 | 3.42E-08 | 30.45 | |
| TRAIL | rs73039026 | 3 | A | C | -0.3098 | 0.9821 | 0.0634 | 1.03E-06 | 23.88 | |
| TRAIL | rs73408359 | 18 | T | C | 0.4153 | 0.049 | 0.0364 | 3.76E-30 | 130.17 | |
| TRAIL | rs74488044 | 18 | A | G | 0.3473 | 0.0583 | 0.0334 | 2.53E-25 | 108.12 | |
| TRAIL | rs747324 | 14 | T | C | -0.0826 | 0.2828 | 0.0178 | 3.48E-06 | 21.53 | |
| TRAIL | rs74778900 | 18 | T | C | 0.5791 | 0.0238 | 0.0531 | 1.08E-27 | 118.94 | |
| TRAIL | rs75473890 | 18 | T | C | -0.1349 | 0.9136 | 0.028 | 1.45E-06 | 23.21 | |
| TRAIL | rs75489499 | 3 | T | C | -0.2006 | 0.0677 | 0.0347 | 7.43E-09 | 33.42 | |
| TRAIL | rs75928541 | 4 | A | G | 0.2784 | 0.0188 | 0.0591 | 2.47E-06 | 22.19 | |
| TRAIL | rs7599203 | 2 | T | C | 0.0918 | 0.8034 | 0.02 | 4.43E-06 | 21.07 | |
| TRAIL | rs79085506 | 18 | A | G | 0.6961 | 0.0154 | 0.0737 | 3.55E-21 | 89.21 | |
| TRAIL | rs9946486 | 18 | A | G | 0.1856 | 0.1159 | 0.0245 | 3.58E-14 | 57.39 | |
| VEGF | rs10411345 | 19 | C | G | -0.1041 | 0.7823 | 0.0218 | 1.80E-06 | 22.80 | |
| VEGF | rs10822118 | 10 | T | C | -0.0797 | 0.4984 | 0.0168 | 2.09E-06 | 22.51 | |
| VEGF | rs10967183 | 9 | T | C | -0.0887 | 0.4262 | 0.0169 | 1.53E-07 | 27.55 | |
| VEGF | rs111950052 | 6 | A | G | -0.1763 | 0.0577 | 0.0383 | 4.16E-06 | 21.19 | |
| VEGF | rs12456390 | 18 | T | C | -0.0818 | 0.6772 | 0.0179 | 4.88E-06 | 20.88 | |
| VEGF | rs13190738 | 6 | T | C | 0.1111 | 0.3644 | 0.0231 | 1.51E-06 | 23.13 | |
| VEGF | rs3025020 | 6 | T | C | -0.124 | 0.2786 | 0.0253 | 9.53E-07 | 24.02 | |
| VEGF | rs4573079 | 6 | A | C | 0.1522 | 0.8439 | 0.0256 | 2.76E-09 | 35.35 | |
| VEGF | rs56071907 | 16 | T | C | 0.126 | 0.1319 | 0.027 | 3.06E-06 | 21.78 | |
| VEGF | rs58078557 | 9 | A | T | -0.1168 | 0.1473 | 0.024 | 1.13E-06 | 23.68 | |
| VEGF | rs60013354 | 10 | A | G | -0.2497 | 0.0266 | 0.0521 | 1.65E-06 | 22.97 | |
| VEGF | rs60987108 | 6 | A | G | 0.1812 | 0.9459 | 0.039 | 3.38E-06 | 21.59 | |
| VEGF | rs6496613 | 15 | A | C | -0.2359 | 0.9677 | 0.0515 | 4.64E-06 | 20.98 | |
| VEGF | rs73872715 | 3 | T | C | -0.6079 | 0.0045 | 0.1299 | 2.87E-06 | 21.90 | |
| VEGF | rs748227 | 6 | T | C | 0.2377 | 0.0654 | 0.0364 | 6.57E-11 | | 42.64 |
| VEGF | rs7739450 | 6 | A | G | -0.415 | 0.5059 | 0.018 | 1.29E-117 | | 531.56 |
| VEGF | rs7754905 | 6 | A | G | -0.1303 | 0.7682 | 0.0204 | 1.69E-10 | 40.80 | |
| VEGF | rs7757024 | 6 | T | C | 0.1443 | 0.0973 | 0.0291 | 7.09E-07 | 24.59 | |
| VEGF | rs77961527 | 3 | A | G | 0.2289 | 0.9606 | 0.0457 | 5.48E-07 | 25.09 | |
| VEGF | rs9369440 | 6 | T | C | -0.0872 | 0.2934 | 0.0186 | 2.76E-06 | 21.98 | |
| VEGF | rs9381249 | 6 | T | C | -0.2414 | 0.9461 | 0.0396 | 1.09E-09 | 37.16 | |
| VEGF | rs9472153 | 6 | A | G | 0.106 | 0.5187 | 0.0174 | 1.12E-09 | 37.11 | |

SNP: single nucleotide polymorphism; EA: effect allele; OA: non-effect allele; Chr: chromosome; EAF: effect allele frequency; Beta was obtained by allele-related effects; SE: standard error. Beta, SE, and P are SNP summary statistics; F: F-statistic.

**Supplementary Table 2. MR results.**

| **Exposure** | **Outcome** | **Method** | **Nsnp** | **OR (95% CI)** | **P** |
| --- | --- | --- | --- | --- | --- |
| B-NGF | OA | IVW | 4 | 0.93 (0.83 to 1.05) | 0.223 |
| CTACK | OA | IVW | 15 | 1.02 (0.95 to 1.09) | 0.539 |
| EOTAXIN | OA | IVW | 23 | 0.93 (0.87 to 0.99) | 0.031 |
| FGF-BASIC | OA | IVW | 7 | 1.12 (0.96 to 1.30) | 0.161 |
| G-CSF | OA | IVW | 8 | 0.97 (0.87 to 1.09) | 0.664 |
| GROA | OA | IVW | 14 | 0.98 (0.92 to 1.04) | 0.445 |
| HGF | OA | IVW | 10 | 1.07 (0.97 to 1.18) | 0.186 |
| IFN-G | OA | IVW | 12 | 1.10 (0.99 to 1.21) | 0.070 |
| IL-10 | OA | IVW | 21 | 0.95 (0.88 to 1.04) | 0.277 |
| IL-12-P70 | OA | IVW | 24 | 0.99 (0.91 to 1.07) | 0.759 |
| IL-13 | OA | IVW | 20 | 0.99 (0.95 to 1.03) | 0.578 |
| IL-16 | OA | IVW | 10 | 1.01 (0.96 to 1.07) | 0.724 |
| IL-17 | OA | IVW | 11 | 1.00 (0.92 to 1.10) | 0.918 |
| IL-18 | OA | IVW | 17 | 0.99 (0.93 to 1.05) | 0.672 |
| IL-1B | OA | IVW | 5 | 0.98 (0.89 to 1.08) | 0.638 |
| IL-1RA | OA | IVW | 9 | 0.98 (0.86 to 1.11) | 0.731 |
| IL-2 | OA | IVW | 8 | 1.01 (0.93 to 1.10) | 0.821 |
| IL-2RA | OA | IVW | 11 | 0.95 (0.89 to 1.02) | 0.133 |
| IL-4 | OA | IVW | 14 | 1.01 (0.92 to 1.11) | 0.861 |
| IL-5 | OA | IVW | 9 | 0.97 (0.90 to 1.05) | 0.471 |
| IL-6 | OA | IVW | 11 | 1.00 (0.88 to 1.12) | 0.945 |
| IL-7 | OA | IVW | 13 | 1.00 (0.95 to 1.07) | 0.883 |
| IL-8 | OA | IVW | 8 | 1.02 (0.96 to 1.09) | 0.495 |
| IL-9 | OA | IVW | 6 | 1.00 (0.92 to 1.09) | 0.967 |
| IP-10 | OA | IVW | 11 | 1.09 (1.01 to 1.17) | 0.024 |
| M-CSF | OA | IVW | 12 | 1.01 (0.94 to 1.08) | 0.850 |
| MCP-1-MCAF | OA | IVW | 22 | 0.97 (0.90 to 1.04) | 0.365 |
| MCP-3 | OA | IVW | 6 | 0.98 (0.91 to 1.04) | 0.478 |
| MIF | OA | IVW | 10 | 0.94 (0.87 to 1.02) | 0.127 |
| MIG | OA | IVW | 14 | 1.02 (0.96 to 1.07) | 0.568 |
| MIP-1A | OA | IVW | 6 | 1.00 (0.90 to 1.11) | 0.998 |
| MIP-1B | OA | IVW | 62 | 1.03 (0.99 to 1.07) | 0.181 |
| PDGF-BB | OA | IVW | 19 | 1.01 (0.93 to 1.09) | 0.819 |
| RANTES | OA | IVW | 10 | 1.05 (0.96 to 1.14) | 0.277 |
| SCF | OA | IVW | 11 | 0.96 (0.87 to 1.06) | 0.442 |
| SCGF-B | OA | IVW | 23 | 0.98 (0.94 to 1.03) | 0.432 |
| SDF-1A | OA | IVW | 10 | 0.99 (0.90 to 1.09) | 0.857 |
| TNF-A | OA | IVW | 4 | 0.97 (0.88 to 1.06) | 0.480 |
| TNF-B | OA | IVW | 6 | 1.03 (0.96 to 1.11) | 0.383 |
| TRAIL | OA | IVW | 29 | 0.96 (0.92 to 1.00) | 0.069 |
| VEGF | OA | IVW | 27 | 0.99 (0.94 to 1.03) | 0.503 |
| B-NGF | KOA | IVW | 4 | 1.03 (0.94 to 1.12) | 0.517 |
| CTACK | KOA | IVW | 16 | 1.00 (0.96 to 1.04) | 0.980 |
| EOTAXIN | KOA | IVW | 22 | 1.00 (0.94 to 1.07) | 0.893 |
| FGF-BASIC | KOA | IVW | 7 | 0.97 (0.89 to 1.05) | 0.436 |
| G-CSF | KOA | IVW | 9 | 0.91 (0.86 to 0.96) | 0.001 |
| GROA | KOA | IVW | 15 | 0.98 (0.94 to 1.01) | 0.179 |
| HGF | KOA | IVW | 10 | 0.99 (0.93 to 1.06) | 0.836 |
| IFN-G | KOA | IVW | 12 | 0.97 (0.91 to 1.04) | 0.430 |
| IL-10 | KOA | IVW | 21 | 1.00 (0.94 to 1.07) | 0.940 |
| IL-12-P70 | KOA | IVW | 23 | 0.98 (0.92 to 1.05) | 0.584 |
| IL-13 | KOA | IVW | 21 | 1.02 (0.99 to 1.05) | 0.281 |
| IL-16 | KOA | IVW | 10 | 1.03 (1.00 to 1.07) | 0.079 |
| IL-17 | KOA | IVW | 11 | 0.97 (0.91 to 1.03) | 0.274 |
| IL-18 | KOA | IVW | 18 | 1.03 (0.99 to 1.07) | 0.140 |
| IL-1B | KOA | IVW | 4 | 0.96 (0.88 to 1.04) | 0.291 |
| IL-1RA | KOA | IVW | 11 | 0.98 (0.91 to 1.05) | 0.583 |
| IL-2 | KOA | IVW | 8 | 0.97 (0.91 to 1.04) | 0.411 |
| IL-2RA | KOA | IVW | 11 | 0.98 (0.93 to 1.03) | 0.495 |
| IL-4 | KOA | IVW | 14 | 0.98 (0.92 to 1.03) | 0.397 |
| IL-5 | KOA | IVW | 9 | 1.02 (0.97 to 1.07) | 0.475 |
| IL-6 | KOA | IVW | 11 | 1.03 (0.96 to 1.10) | 0.400 |
| IL-7 | KOA | IVW | 14 | 1.00 (0.96 to 1.04) | 0.905 |
| IL-8 | KOA | IVW | 8 | 0.99 (0.93 to 1.04) | 0.628 |
| IL-9 | KOA | IVW | 6 | 1.04 (0.98 to 1.10) | 0.201 |
| IP-10 | KOA | IVW | 12 | 1.00 (0.96 to 1.05) | 0.965 |
| M-CSF | KOA | IVW | 12 | 1.01 (0.98 to 1.05) | 0.533 |
| MCP-1-MCAF | KOA | IVW | 22 | 1.03 (0.97 to 1.09) | 0.384 |
| MCP-3 | KOA | IVW | 6 | 1.00 (0.96 to 1.04) | 0.832 |
| MIF | KOA | IVW | 10 | 1.01 (0.96 to 1.06) | 0.689 |
| MIG | KOA | IVW | 14 | 1.00 (0.96 to 1.03) | 0.906 |
| MIP-1A | KOA | IVW | 6 | 1.05 (0.98 to 1.12) | 0.175 |
| MIP-1B | KOA | IVW | 67 | 1.00 (0.98 to 1.02) | 0.929 |
| PDGF-BB | KOA | IVW | 19 | 0.96 (0.91 to 1.01) | 0.079 |
| RANTES | KOA | IVW | 10 | 1.03 (0.98 to 1.09) | 0.188 |
| SCF | KOA | IVW | 12 | 0.99 (0.93 to 1.06) | 0.841 |
| SCGF-B | KOA | IVW | 23 | 1.00 (0.97 to 1.03) | 0.861 |
| SDF-1A | KOA | IVW | 10 | 0.99 (0.93 to 1.05) | 0.735 |
| TNF-A | KOA | IVW | 4 | 0.95 (0.90 to 1.01) | 0.102 |
| TNF-B | KOA | IVW | 6 | 1.00 (0.94 to 1.05) | 0.876 |
| TRAIL | KOA | IVW | 30 | 1.00 (0.97 to 1.04) | 0.816 |
| VEGF | KOA | IVW | 27 | 1.02 (0.99 to 1.05) | 0.251 |
| B-NGF | HOA | IVW | 4 | 0.98 (0.89 to 1.08) | 0.636 |
| CTACK | HOA | IVW | 16 | 0.98 (0.92 to 1.04) | 0.485 |
| EOTAXIN | HOA | IVW | 23 | 0.98 (0.93 to 1.04) | 0.474 |
| FGF-BASIC | HOA | IVW | 7 | 1.01 (0.90 to 1.13) | 0.877 |
| G-CSF | HOA | IVW | 9 | 0.98 (0.91 to 1.05) | 0.520 |
| GROA | HOA | IVW | 15 | 0.99 (0.94 to 1.03) | 0.505 |
| HGF | HOA | IVW | 10 | 1.05 (0.97 to 1.14) | 0.199 |
| IFN-G | HOA | IVW | 12 | 1.02 (0.93 to 1.12) | 0.665 |
| IL-10 | HOA | IVW | 21 | 1.00 (0.93 to 1.07) | 0.950 |
| IL-12-P70 | HOA | IVW | 24 | 0.99 (0.93 to 1.05) | 0.762 |
| IL-13 | HOA | IVW | 21 | 1.01 (0.97 to 1.05) | 0.580 |
| IL-16 | HOA | IVW | 10 | 1.01 (0.95 to 1.07) | 0.863 |
| IL-17 | HOA | IVW | 11 | 1.00 (0.89 to 1.11) | 0.950 |
| IL-18 | HOA | IVW | 18 | 1.03 (0.99 to 1.07) | 0.202 |
| IL-1B | HOA | IVW | 4 | 0.95 (0.86 to 1.04) | 0.274 |
| IL-1RA | HOA | IVW | 11 | 1.00 (0.91 to 1.09) | 0.934 |
| IL-2 | HOA | IVW | 8 | 1.01 (0.92 to 1.11) | 0.869 |
| IL-2RA | HOA | IVW | 11 | 1.01 (0.94 to 1.09) | 0.701 |
| IL-4 | HOA | IVW | 13 | 1.01 (0.92 to 1.10) | 0.860 |
| IL-5 | HOA | IVW | 9 | 1.01 (0.93 to 1.09) | 0.845 |
| IL-6 | HOA | IVW | 11 | 0.94 (0.85 to 1.03) | 0.189 |
| IL-7 | HOA | IVW | 14 | 1.03 (0.99 to 1.08) | 0.164 |
| IL-8 | HOA | IVW | 8 | 0.98 (0.93 to 1.04) | 0.472 |
| IL-9 | HOA | IVW | 6 | 0.98 (0.91 to 1.05) | 0.534 |
| IP-10 | HOA | IVW | 12 | 0.97 (0.92 to 1.03) | 0.344 |
| M-CSF | HOA | IVW | 12 | 0.99 (0.94 to 1.04) | 0.612 |
| MCP-1-MCAF | HOA | IVW | 22 | 1.01 (0.95 to 1.08) | 0.775 |
| MCP-3 | HOA | IVW | 6 | 1.00 (0.96 to 1.05) | 0.864 |
| MIF | HOA | IVW | 10 | 1.00 (0.94 to 1.06) | 0.938 |
| MIG | HOA | IVW | 14 | 0.99 (0.95 to 1.04) | 0.732 |
| MIP-1A | HOA | IVW | 6 | 1.06 (0.97 to 1.15) | 0.214 |
| MIP-1B | HOA | IVW | 67 | 1.01 (0.98 to 1.04) | 0.482 |
| PDGF-BB | HOA | IVW | 19 | 0.98 (0.92 to 1.04) | 0.539 |
| RANTES | HOA | IVW | 10 | 1.00 (0.94 to 1.07) | 0.895 |
| SCF | HOA | IVW | 12 | 0.90 (0.83 to 0.97) | 0.004 |
| SCGF-B | HOA | IVW | 23 | 1.01 (0.97 to 1.05) | 0.735 |
| SDF-1A | HOA | IVW | 10 | 0.97 (0.90 to 1.05) | 0.497 |
| TNF-A | HOA | IVW | 4 | 0.98 (0.91 to 1.06) | 0.629 |
| TNF-B | HOA | IVW | 6 | 1.01 (0.96 to 1.06) | 0.704 |
| TRAIL | HOA | IVW | 30 | 1.00 (0.96 to 1.04) | 0.895 |
| VEGF | HOA | IVW | 27 | 1.01 (0.98 to 1.05) | 0.429 |
| B-NGF | OP | IVW | 4 | 1.00 (0.99 to 1.00) | 0.416 |
| CTACK | OP | IVW | 6 | 1.00 (0.99 to 1.00) | 0.417 |
| EOTAXIN | OP | IVW | 21 | 1.00 (1.00 to 1.00) | 0.659 |
| FGF-BASIC | OP | IVW | 5 | 1.00 (1.00 to 1.00) | 0.823 |
| G-CSF | OP | IVW | 5 | 1.00 (1.00 to 1.00) | 0.721 |
| GROA | OP | IVW | 5 | 1.00 (1.00 to 1.00) | 0.014 |
| HGF | OP | IVW | 8 | 1.00 (1.00 to 1.00) | 0.699 |
| IFN-G | OP | IVW | 7 | 1.00 (1.00 to 1.00) | 0.280 |
| IL-10 | OP | IVW | 19 | 1.00 (1.00 to 1.00) | 0.690 |
| IL-12-P70 | OP | IVW | 19 | 1.00 (1.00 to 1.00) | 0.805 |
| IL-13 | OP | IVW | 11 | 1.00 (1.00 to 1.00) | 0.947 |
| IL-16 | OP | IVW | 4 | 1.00 (1.00 to 1.00) | 0.794 |
| IL-17 | OP | IVW | 7 | 1.00 (1.00 to 1.00) | 0.598 |
| IL-18 | OP | IVW | 10 | 1.00 (1.00 to 1.00) | 0.221 |
| IL-1B | OP | IVW | 3 | 1.00 (1.00 to 1.00) | 0.966 |
| IL-1RA | OP | IVW | 6 | 1.00 (1.00 to 1.00) | 0.868 |
| IL-2 | OP | IVW | 6 | 1.00 (1.00 to 1.00) | 0.282 |
| IL-2RA | OP | IVW | 6 | 1.00 (1.00 to 1.00) | 0.679 |
| IL-4 | OP | IVW | 9 | 1.00 (1.00 to 1.00) | 0.956 |
| IL-5 | OP | IVW | 6 | 1.00 (1.00 to 1.00) | 0.731 |
| IL-6 | OP | IVW | 8 | 1.00 (1.00 to 1.00) | 0.093 |
| IL-7 | OP | IVW | 8 | 1.00 (1.00 to 1.00) | 0.596 |
| IL-8 | OP | IVW | 4 | 1.00 (1.00 to 1.00) | 0.316 |
| IL-9 | OP | IVW | 3 | 1.00 (1.00 to 1.00) | 0.067 |
| IP-10 | OP | IVW | 8 | 1.00 (1.00 to 1.00) | 0.812 |
| M-CSF | OP | IVW | 7 | 1.00 (1.00 to 1.00) | 0.497 |
| MCP-1-MCAF | OP | IVW | 11 | 1.00 (1.00 to 1.00) | 0.632 |
| MCP-3 | OP | IVW | 4 | 1.00 (1.00 to 1.00) | 0.778 |
| MIF | OP | IVW | 6 | 1.00 (1.00 to 1.00) | 0.333 |
| MIG | OP | IVW | 8 | 1.00 (1.00 to 1.00) | 0.850 |
| MIP-1A | OP | IVW | 4 | 1.00 (1.00 to 1.01) | 0.157 |
| MIP-1B | OP | IVW | 42 | 1.00 (1.00 to 1.00) | 0.032 |
| PDGF-BB | OP | IVW | 10 | 1.00 (1.00 to 1.00) | 0.553 |
| RANTES | OP | IVW | 5 | 1.00 (1.00 to 1.00) | 0.419 |
| SCF | OP | IVW | 8 | 1.00 (1.00 to 1.00) | 0.440 |
| SCGF-B | OP | IVW | 11 | 1.00 (1.00 to 1.00) | 0.758 |
| SDF-1A | OP | IVW | 4 | 1.00 (1.00 to 1.00) | 0.997 |
| TNF-A | OP | IVW | 3 | 1.00 (0.99 to 1.00) | 0.094 |
| TNF-B | OP | IVW | 4 | 1.00 (1.00 to 1.00) | 0.450 |
| TRAIL | OP | IVW | 17 | 1.00 (1.00 to 1.00) | 0.295 |
| VEGF | OP | IVW | 19 | 1.00 (1.00 to 1.00) | 0.672 |
| B-NGF | RA | IVW | 4 | 1.12 (0.89 to 1.42) | 0.330 |
| CTACK | RA | IVW | 15 | 1.01 (0.94 to 1.08) | 0.778 |
| EOTAXIN | RA | IVW | 21 | 0.92 (0.86 to 0.99) | 0.035 |
| FGF-BASIC | RA | IVW | 6 | 0.93 (0.85 to 1.02) | 0.116 |
| G-CSF | RA | IVW | 9 | 0.95 (0.88 to 1.03) | 0.215 |
| GROA | RA | IVW | 13 | 0.98 (0.94 to 1.03) | 0.497 |
| HGF | RA | IVW | 10 | 1.08 (0.96 to 1.22) | 0.183 |
| IFN-G | RA | IVW | 10 | 1.08 (0.96 to 1.22) | 0.180 |
| IL-10 | RA | IVW | 18 | 1.07 (0.99 to 1.16) | 0.110 |
| IL-12-P70 | RA | IVW | 21 | 1.03 (0.95 to 1.12) | 0.439 |
| IL-13 | RA | IVW | 17 | 0.96 (0.92 to 1.01) | 0.133 |
| IL-16 | RA | IVW | 9 | 1.00 (0.95 to 1.04) | 0.862 |
| IL-17 | RA | IVW | 9 | 0.97 (0.87 to 1.10) | 0.671 |
| IL-18 | RA | IVW | 14 | 1.04 (0.97 to 1.11) | 0.316 |
| IL-1B | RA | IVW | 4 | 0.97 (0.89 to 1.05) | 0.404 |
| IL-1RA | RA | IVW | 10 | 0.97 (0.87 to 1.08) | 0.527 |
| IL-2 | RA | IVW | 8 | 1.00 (0.92 to 1.09) | 0.989 |
| IL-2RA | RA | IVW | 10 | 0.97 (0.85 to 1.10) | 0.596 |
| IL-4 | RA | IVW | 12 | 1.00 (0.89 to 1.12) | 0.997 |
| IL-5 | RA | IVW | 8 | 0.91 (0.83 to 0.98) | 0.019 |
| IL-6 | RA | IVW | 9 | 0.92 (0.82 to 1.04) | 0.182 |
| IL-7 | RA | IVW | 13 | 1.01 (0.95 to 1.08) | 0.648 |
| IL-8 | RA | IVW | 8 | 1.01 (0.92 to 1.10) | 0.902 |
| IL-9 | RA | IVW | 5 | 0.98 (0.85 to 1.13) | 0.797 |
| IP-10 | RA | IVW | 9 | 0.91 (0.81 to 1.02) | 0.089 |
| M-CSF | RA | IVW | 11 | 0.99 (0.92 to 1.07) | 0.817 |
| MCP-1-MCAF | RA | IVW | 20 | 1.01 (0.92 to 1.10) | 0.893 |
| MCP-3 | RA | IVW | 6 | 0.95 (0.89 to 1.02) | 0.138 |
| MIF | RA | IVW | 8 | 1.00 (0.91 to 1.11) | 0.968 |
| MIG | RA | IVW | 11 | 0.97 (0.87 to 1.07) | 0.492 |
| MIP-1A | RA | IVW | 6 | 0.91 (0.79 to 1.06) | 0.242 |
| MIP-1B | RA | IVW | 58 | 1.02 (0.97 to 1.07) | 0.431 |
| PDGF-BB | RA | IVW | 10 | 0.89 (0.80 to 0.98) | 0.022 |
| RANTES | RA | IVW | 10 | 0.98 (0.90 to 1.06) | 0.616 |
| SCF | RA | IVW | 11 | 1.06 (0.99 to 1.12) | 0.082 |
| SCGF-B | RA | IVW | 21 | 1.03 (0.97 to 1.09) | 0.372 |
| SDF-1A | RA | IVW | 7 | 0.93 (0.86 to 1.02) | 0.121 |
| TNF-A | RA | IVW | 4 | 0.96 (0.85 to 1.09) | 0.543 |
| TNF-B | RA | IVW | 6 | 1.03 (0.98 to 1.09) | 0.226 |
| TRAIL | RA | IVW | 27 | 0.98 (0.94 to 1.02) | 0.369 |
| VEGF | RA | IVW | 22 | 1.00 (0.95 to 1.04) | 0.839 |

Results of MR analysis between circulating inflammatory factors and OA/KOA/HOA/OP/RA. IVW: inverse variance weighting method; OA: Osteoarthritis; KOA: Knee osteoarthritis; HOA: Hip osteoarthritis; OP: Osteoporosis; RA: Rheumatoid arthritis.

**Supplementary Table 3. Sensitivity Analysis.**

| Exposure | Outcome | Heterogeneity test (MR-Egger) | | Heterogeneity test (IVW) | | Horizontal pleiotropy test (MR-Egger) | |
| --- | --- | --- | --- | --- | --- | --- | --- |
|  |  | Cochran's Q | P | Cochran's Q | P | Intercept | P |
| B-NGF | OA | 1.94 | 0.38 | 2.26 | 0.52 | 0.03 | 0.63 |
| CTACK | OA | 18.55 | 0.14 | 21.69 | 0.09 | 0.02 | 0.16 |
| EOTAXIN | OA | 15.16 | 0.82 | 16.40 | 0.80 | -0.01 | 0.28 |
| FGF-BASIC | OA | 6.82 | 0.23 | 8.25 | 0.22 | -0.02 | 0.35 |
| G-CSF | OA | 11.59 | 0.07 | 11.75 | 0.11 | 0.00 | 0.78 |
| GROA | OA | 16.74 | 0.16 | 17.19 | 0.19 | -0.01 | 0.58 |
| HGF | OA | 7.36 | 0.50 | 7.86 | 0.55 | 0.01 | 0.50 |
| IFN-G | OA | 10.24 | 0.42 | 10.24 | 0.51 | 0.00 | 1.00 |
| IL-10 | OA | 23.05 | 0.24 | 23.41 | 0.27 | 0.01 | 0.59 |
| IL-12-P70 | OA | 25.29 | 0.28 | 25.47 | 0.33 | 0.01 | 0.69 |
| IL-13 | OA | 15.60 | 0.62 | 16.46 | 0.63 | 0.01 | 0.37 |
| IL-16 | OA | 7.70 | 0.46 | 7.81 | 0.55 | 0.00 | 0.74 |
| IL-17 | OA | 5.45 | 0.79 | 5.79 | 0.83 | 0.01 | 0.57 |
| IL-18 | OA | 19.88 | 0.18 | 22.97 | 0.11 | 0.02 | 0.15 |
| IL-1B | OA | 1.30 | 0.73 | 1.56 | 0.82 | 0.01 | 0.64 |
| IL-1RA | OA | 17.31 | 0.02 | 17.44 | 0.03 | -0.01 | 0.82 |
| IL-2 | OA | 8.15 | 0.23 | 9.48 | 0.22 | 0.01 | 0.36 |
| IL-2RA | OA | 7.10 | 0.63 | 7.12 | 0.71 | 0.00 | 0.87 |
| IL-4 | OA | 17.77 | 0.12 | 18.09 | 0.15 | 0.01 | 0.65 |
| IL-5 | OA | 3.25 | 0.86 | 3.27 | 0.92 | 0.00 | 0.89 |
| IL-6 | OA | 14.18 | 0.12 | 15.39 | 0.12 | -0.01 | 0.40 |
| IL-7 | OA | 15.48 | 0.16 | 16.45 | 0.17 | 0.01 | 0.42 |
| IL-8 | OA | 5.30 | 0.51 | 5.97 | 0.54 | -0.01 | 0.44 |
| IL-9 | OA | 1.74 | 0.78 | 1.77 | 0.88 | 0.00 | 0.88 |
| IP-10 | OA | 10.37 | 0.32 | 10.60 | 0.39 | 0.01 | 0.66 |
| M-CSF | OA | 19.73 | 0.03 | 21.64 | 0.03 | 0.02 | 0.35 |
| MCP-1-MCAF | OA | 12.80 | 0.89 | 12.80 | 0.92 | 0.00 | 0.98 |
| MCP-3 | OA | 4.78 | 0.31 | 7.69 | 0.17 | -0.03 | 0.19 |
| MIF | OA | 8.46 | 0.39 | 8.62 | 0.47 | -0.01 | 0.70 |
| MIG | OA | 10.01 | 0.61 | 12.99 | 0.45 | 0.02 | 0.11 |
| MIP-1A | OA | 3.87 | 0.42 | 3.87 | 0.57 | 0.00 | 0.95 |
| MIP-1B | OA | 64.61 | 0.32 | 65.08 | 0.34 | 0.00 | 0.51 |
| PDGF-BB | OA | 14.79 | 0.61 | 14.82 | 0.67 | 0.00 | 0.86 |
| RANTES | OA | 11.22 | 0.19 | 11.23 | 0.26 | 0.00 | 0.92 |
| SCF | OA | 10.36 | 0.32 | 11.07 | 0.35 | 0.01 | 0.45 |
| SCGF-B | OA | 17.27 | 0.69 | 17.66 | 0.73 | -0.01 | 0.54 |
| SDF-1A | OA | 6.24 | 0.62 | 7.69 | 0.57 | -0.01 | 0.26 |
| TNF-A | OA | 1.03 | 0.60 | 1.25 | 0.74 | 0.01 | 0.68 |
| TNF-B | OA | 5.02 | 0.29 | 7.37 | 0.19 | -0.02 | 0.24 |
| TRAIL | OA | 20.95 | 0.79 | 21.17 | 0.82 | 0.00 | 0.64 |
| VEGF | OA | 16.97 | 0.88 | 17.22 | 0.90 | 0.00 | 0.62 |
| B-NGF | KOA | 1.87 | 0.39 | 3.82 | 0.28 | -0.04 | 0.30 |
| CTACK | KOA | 15.09 | 0.37 | 15.90 | 0.39 | 0.01 | 0.40 |
| EOTAXIN | KOA | 39.77 | 0.01 | 43.19 | 0.00 | -0.01 | 0.20 |
| FGF-BASIC | KOA | 4.57 | 0.47 | 4.60 | 0.60 | 0.00 | 0.89 |
| G-CSF | KOA | 1.89 | 0.97 | 3.68 | 0.88 | -0.01 | 0.22 |
| GROA | KOA | 3.89 | 0.99 | 11.62 | 0.64 | -0.02 | 0.02 |
| HGF | KOA | 8.15 | 0.42 | 8.39 | 0.50 | 0.00 | 0.64 |
| IFN-G | KOA | 9.85 | 0.45 | 11.78 | 0.38 | 0.01 | 0.20 |
| IL-10 | KOA | 30.79 | 0.04 | 31.84 | 0.04 | -0.01 | 0.43 |
| IL-12-P70 | KOA | 31.11 | 0.07 | 33.20 | 0.06 | 0.01 | 0.25 |
| IL-13 | KOA | 26.54 | 0.12 | 27.72 | 0.12 | 0.01 | 0.37 |
| IL-16 | KOA | 7.44 | 0.49 | 9.64 | 0.38 | 0.01 | 0.18 |
| IL-17 | KOA | 6.65 | 0.67 | 7.81 | 0.65 | -0.01 | 0.31 |
| IL-18 | KOA | 24.02 | 0.09 | 24.88 | 0.10 | 0.01 | 0.46 |
| IL-1B | KOA | 2.95 | 0.23 | 3.56 | 0.31 | 0.01 | 0.59 |
| IL-1RA | KOA | 19.01 | 0.03 | 20.28 | 0.03 | 0.01 | 0.46 |
| IL-2 | KOA | 10.28 | 0.11 | 12.94 | 0.07 | 0.01 | 0.26 |
| IL-2RA | KOA | 13.64 | 0.14 | 14.10 | 0.17 | -0.01 | 0.59 |
| IL-4 | KOA | 13.76 | 0.32 | 14.62 | 0.33 | 0.01 | 0.40 |
| IL-5 | KOA | 3.05 | 0.88 | 3.07 | 0.93 | 0.00 | 0.90 |
| IL-6 | KOA | 11.07 | 0.27 | 11.51 | 0.32 | -0.01 | 0.57 |
| IL-7 | KOA | 17.39 | 0.14 | 17.56 | 0.18 | 0.00 | 0.74 |
| IL-8 | KOA | 9.01 | 0.17 | 11.73 | 0.11 | 0.01 | 0.23 |
| IL-9 | KOA | 2.32 | 0.68 | 2.33 | 0.80 | 0.00 | 0.91 |
| IP-10 | KOA | 5.81 | 0.83 | 6.87 | 0.81 | -0.01 | 0.33 |
| M-CSF | KOA | 8.39 | 0.59 | 8.77 | 0.64 | 0.01 | 0.55 |
| MCP-1-MCAF | KOA | 29.82 | 0.07 | 29.85 | 0.09 | 0.00 | 0.88 |
| MCP-3 | KOA | 4.94 | 0.29 | 5.82 | 0.32 | -0.01 | 0.45 |
| MIF | KOA | 6.30 | 0.61 | 6.67 | 0.67 | -0.01 | 0.56 |
| MIG | KOA | 6.25 | 0.90 | 9.66 | 0.72 | 0.01 | 0.09 |
| MIP-1A | KOA | 1.55 | 0.82 | 1.57 | 0.90 | 0.00 | 0.88 |
| MIP-1B | KOA | 56.35 | 0.77 | 56.86 | 0.78 | 0.00 | 0.48 |
| PDGF-BB | KOA | 14.63 | 0.62 | 14.67 | 0.68 | 0.00 | 0.85 |
| RANTES | KOA | 6.02 | 0.65 | 6.36 | 0.70 | -0.01 | 0.57 |
| SCF | KOA | 11.10 | 0.35 | 12.59 | 0.32 | 0.01 | 0.27 |
| SCGF-B | KOA | 22.67 | 0.36 | 23.18 | 0.39 | 0.00 | 0.50 |
| SDF-1A | KOA | 1.71 | 0.99 | 1.81 | 0.99 | 0.00 | 0.76 |
| TNF-A | KOA | 0.09 | 0.95 | 0.14 | 0.99 | 0.00 | 0.85 |
| TNF-B | KOA | 7.17 | 0.13 | 9.85 | 0.08 | -0.02 | 0.29 |
| TRAIL | KOA | 32.40 | 0.26 | 38.00 | 0.12 | -0.01 | 0.04 |
| VEGF | KOA | 35.22 | 0.08 | 36.11 | 0.09 | 0.00 | 0.43 |
| B-NGF | HOA | 1.78 | 0.41 | 2.52 | 0.47 | -0.03 | 0.48 |
| CTACK | HOA | 19.23 | 0.16 | 25.06 | 0.05 | 0.02 | 0.06 |
| EOTAXIN | HOA | 15.96 | 0.77 | 16.03 | 0.81 | 0.00 | 0.79 |
| FGF-BASIC | HOA | 3.70 | 0.59 | 6.76 | 0.34 | 0.03 | 0.14 |
| G-CSF | HOA | 7.34 | 0.39 | 7.37 | 0.50 | 0.00 | 0.88 |
| GROA | HOA | 12.74 | 0.47 | 12.99 | 0.53 | -0.01 | 0.63 |
| HGF | HOA | 4.00 | 0.86 | 4.56 | 0.87 | -0.01 | 0.48 |
| IFN-G | HOA | 9.83 | 0.46 | 15.18 | 0.17 | 0.02 | 0.04 |
| IL-10 | HOA | 23.41 | 0.22 | 23.89 | 0.25 | -0.01 | 0.54 |
| IL-12-P70 | HOA | 12.23 | 0.95 | 12.49 | 0.96 | -0.01 | 0.62 |
| IL-13 | HOA | 22.83 | 0.25 | 22.84 | 0.30 | 0.00 | 0.91 |
| IL-16 | HOA | 15.43 | 0.05 | 16.94 | 0.05 | 0.01 | 0.40 |
| IL-17 | HOA | 18.99 | 0.03 | 19.80 | 0.03 | 0.01 | 0.55 |
| IL-18 | HOA | 10.31 | 0.85 | 14.63 | 0.62 | 0.02 | 0.05 |
| IL-1B | HOA | 3.29 | 0.19 | 3.30 | 0.35 | 0.00 | 0.96 |
| IL-1RA | HOA | 21.12 | 0.01 | 21.88 | 0.02 | 0.01 | 0.58 |
| IL-2 | HOA | 7.21 | 0.30 | 17.31 | 0.02 | 0.03 | 0.03 |
| IL-2RA | HOA | 18.73 | 0.03 | 18.95 | 0.04 | 0.00 | 0.75 |
| IL-4 | HOA | 20.01 | 0.05 | 22.25 | 0.03 | 0.01 | 0.29 |
| IL-5 | HOA | 12.26 | 0.09 | 12.26 | 0.14 | 0.00 | 0.97 |
| IL-6 | HOA | 9.69 | 0.38 | 13.70 | 0.19 | 0.02 | 0.09 |
| IL-7 | HOA | 15.81 | 0.20 | 15.92 | 0.25 | 0.00 | 0.79 |
| IL-8 | HOA | 5.76 | 0.45 | 6.54 | 0.48 | 0.01 | 0.41 |
| IL-9 | HOA | 2.42 | 0.66 | 2.46 | 0.78 | 0.00 | 0.85 |
| IP-10 | HOA | 3.57 | 0.96 | 5.63 | 0.90 | 0.02 | 0.18 |
| M-CSF | HOA | 11.40 | 0.33 | 13.99 | 0.23 | 0.02 | 0.16 |
| MCP-1-MCAF | HOA | 22.45 | 0.32 | 22.82 | 0.35 | 0.01 | 0.57 |
| MCP-3 | HOA | 2.65 | 0.62 | 3.37 | 0.64 | -0.01 | 0.44 |
| MIF | HOA | 7.69 | 0.46 | 7.73 | 0.56 | 0.00 | 0.84 |
| MIG | HOA | 8.80 | 0.72 | 11.41 | 0.58 | -0.02 | 0.13 |
| MIP-1A | HOA | 4.70 | 0.32 | 5.06 | 0.41 | -0.01 | 0.61 |
| MIP-1B | HOA | 77.42 | 0.14 | 77.72 | 0.15 | 0.00 | 0.62 |
| PDGF-BB | HOA | 13.48 | 0.70 | 14.08 | 0.72 | -0.01 | 0.45 |
| RANTES | HOA | 7.07 | 0.53 | 9.48 | 0.39 | 0.02 | 0.16 |
| SCF | HOA | 8.87 | 0.54 | 9.92 | 0.54 | 0.01 | 0.33 |
| SCGF-B | HOA | 16.72 | 0.73 | 18.50 | 0.68 | -0.01 | 0.20 |
| SDF-1A | HOA | 8.06 | 0.43 | 8.89 | 0.45 | -0.01 | 0.39 |
| TNF-A | HOA | 0.51 | 0.77 | 2.55 | 0.47 | -0.02 | 0.29 |
| TNF-B | HOA | 3.66 | 0.45 | 3.69 | 0.59 | 0.00 | 0.86 |
| TRAIL | HOA | 32.88 | 0.24 | 34.81 | 0.21 | 0.01 | 0.21 |
| VEGF | HOA | 24.43 | 0.49 | 24.44 | 0.55 | 0.00 | 0.96 |
| B-NGF | OP | 8.14 | 0.02 | 8.24 | 0.04 | 0.00 | 0.89 |
| CTACK | OP | 18.51 | 0.00 | 19.06 | 0.00 | 0.00 | 0.75 |
| EOTAXIN | OP | 15.68 | 0.68 | 26.11 | 0.16 | 0.00 | 0.00 |
| FGF-BASIC | OP | 1.82 | 0.61 | 1.85 | 0.76 | 0.00 | 0.86 |
| G-CSF | OP | 5.01 | 0.17 | 6.02 | 0.20 | 0.00 | 0.49 |
| GROA | OP | 0.59 | 0.90 | 0.78 | 0.94 | 0.00 | 0.69 |
| HGF | OP | 6.60 | 0.36 | 6.77 | 0.45 | 0.00 | 0.71 |
| IFN-G | OP | 6.32 | 0.28 | 8.64 | 0.19 | 0.00 | 0.23 |
| IL-10 | OP | 12.31 | 0.78 | 12.32 | 0.83 | 0.00 | 0.90 |
| IL-12-P70 | OP | 15.01 | 0.59 | 20.47 | 0.31 | 0.00 | 0.03 |
| IL-13 | OP | 8.31 | 0.50 | 10.94 | 0.36 | 0.00 | 0.14 |
| IL-16 | OP | 2.91 | 0.23 | 3.33 | 0.34 | 0.00 | 0.65 |
| IL-17 | OP | 6.48 | 0.26 | 7.78 | 0.25 | 0.00 | 0.36 |
| IL-18 | OP | 9.19 | 0.33 | 9.90 | 0.36 | 0.00 | 0.45 |
| IL-1B | OP | 2.01 | 0.16 | 2.12 | 0.35 | 0.00 | 0.86 |
| IL-1RA | OP | 2.39 | 0.66 | 2.81 | 0.73 | 0.00 | 0.55 |
| IL-2 | OP | 2.99 | 0.56 | 3.33 | 0.65 | 0.00 | 0.59 |
| IL-2RA | OP | 4.36 | 0.36 | 5.08 | 0.41 | 0.00 | 0.46 |
| IL-4 | OP | 5.87 | 0.55 | 5.89 | 0.66 | 0.00 | 0.91 |
| IL-5 | OP | 2.72 | 0.61 | 2.73 | 0.74 | 0.00 | 0.93 |
| IL-6 | OP | 5.57 | 0.47 | 5.63 | 0.58 | 0.00 | 0.82 |
| IL-7 | OP | 7.34 | 0.29 | 8.16 | 0.32 | 0.00 | 0.45 |
| IL-8 | OP | 0.34 | 0.85 | 0.97 | 0.81 | 0.00 | 0.51 |
| IL-9 | OP | 0.17 | 0.68 | 0.19 | 0.91 | 0.00 | 0.90 |
| IP-10 | OP | 5.73 | 0.45 | 5.73 | 0.57 | 0.00 | 0.97 |
| M-CSF | OP | 7.75 | 0.17 | 9.96 | 0.13 | 0.00 | 0.29 |
| MCP-1-MCAF | OP | 10.86 | 0.29 | 12.17 | 0.27 | 0.00 | 0.33 |
| MCP-3 | OP | 3.91 | 0.14 | 4.38 | 0.22 | 0.00 | 0.67 |
| MIF | OP | 4.18 | 0.38 | 5.63 | 0.34 | 0.00 | 0.30 |
| MIG | OP | 9.07 | 0.17 | 9.11 | 0.24 | 0.00 | 0.88 |
| MIP-1A | OP | 5.33 | 0.07 | 6.71 | 0.08 | 0.00 | 0.55 |
| MIP-1B | OP | 47.26 | 0.20 | 47.83 | 0.22 | 0.00 | 0.49 |
| PDGF-BB | OP | 13.19 | 0.11 | 13.43 | 0.14 | 0.00 | 0.72 |
| RANTES | OP | 3.74 | 0.29 | 8.78 | 0.07 | 0.00 | 0.14 |
| SCF | OP | 7.21 | 0.30 | 7.21 | 0.41 | 0.00 | 0.96 |
| SCGF-B | OP | 13.60 | 0.14 | 13.63 | 0.19 | 0.00 | 0.90 |
| SDF-1A | OP | 0.21 | 0.90 | 0.23 | 0.97 | 0.00 | 0.93 |
| TNF-A | OP | 1.33 | 0.25 | 3.16 | 0.21 | 0.00 | 0.45 |
| TNF-B | OP | 3.23 | 0.20 | 3.25 | 0.35 | 0.00 | 0.91 |
| TRAIL | OP | 18.66 | 0.23 | 20.71 | 0.19 | 0.00 | 0.22 |
| VEGF | OP | 12.37 | 0.78 | 12.56 | 0.82 | 0.00 | 0.66 |
| B-NGF | RA | 10.42 | 0.01 | 10.97 | 0.01 | 0.04 | 0.78 |
| CTACK | RA | 9.09 | 0.77 | 11.75 | 0.63 | -0.02 | 0.13 |
| EOTAXIN | RA | 17.17 | 0.58 | 17.97 | 0.59 | -0.01 | 0.38 |
| FGF-BASIC | RA | 0.79 | 0.94 | 0.84 | 0.97 | 0.00 | 0.83 |
| G-CSF | RA | 4.44 | 0.73 | 4.44 | 0.81 | 0.00 | 0.95 |
| GROA | RA | 10.39 | 0.50 | 11.07 | 0.52 | -0.01 | 0.43 |
| HGF | RA | 3.74 | 0.88 | 4.61 | 0.87 | -0.01 | 0.38 |
| IFN-G | RA | 6.01 | 0.65 | 6.71 | 0.67 | 0.02 | 0.43 |
| IL-10 | RA | 13.58 | 0.63 | 13.76 | 0.68 | 0.01 | 0.68 |
| IL-12-P70 | RA | 16.85 | 0.60 | 18.52 | 0.55 | 0.02 | 0.21 |
| IL-13 | RA | 13.15 | 0.59 | 14.69 | 0.55 | -0.01 | 0.23 |
| IL-16 | RA | 7.05 | 0.42 | 9.10 | 0.33 | 0.02 | 0.20 |
| IL-17 | RA | 5.72 | 0.57 | 6.86 | 0.55 | -0.03 | 0.32 |
| IL-18 | RA | 9.57 | 0.65 | 11.33 | 0.58 | -0.02 | 0.21 |
| IL-1B | RA | 2.27 | 0.32 | 2.28 | 0.52 | 0.00 | 0.96 |
| IL-1RA | RA | 14.20 | 0.08 | 14.21 | 0.11 | 0.00 | 0.92 |
| IL-2 | RA | 4.43 | 0.62 | 4.44 | 0.73 | 0.00 | 0.94 |
| IL-2RA | RA | 21.73 | 0.01 | 23.43 | 0.01 | -0.02 | 0.45 |
| IL-4 | RA | 11.32 | 0.33 | 13.15 | 0.28 | 0.02 | 0.23 |
| IL-5 | RA | 8.79 | 0.19 | 8.83 | 0.26 | 0.00 | 0.87 |
| IL-6 | RA | 6.19 | 0.52 | 9.70 | 0.29 | -0.02 | 0.10 |
| IL-7 | RA | 10.91 | 0.45 | 12.79 | 0.38 | 0.02 | 0.20 |
| IL-8 | RA | 6.27 | 0.39 | 6.27 | 0.51 | 0.00 | 0.95 |
| IL-9 | RA | 2.73 | 0.44 | 2.85 | 0.58 | -0.01 | 0.75 |
| IP-10 | RA | 10.55 | 0.16 | 10.56 | 0.23 | 0.00 | 0.93 |
| M-CSF | RA | 16.70 | 0.05 | 16.76 | 0.08 | 0.01 | 0.86 |
| MCP-1-MCAF | RA | 23.31 | 0.18 | 23.45 | 0.22 | -0.01 | 0.74 |
| MCP-3 | RA | 2.74 | 0.60 | 7.38 | 0.19 | 0.05 | 0.10 |
| MIF | RA | 2.72 | 0.84 | 3.42 | 0.84 | 0.02 | 0.44 |
| MIG | RA | 14.22 | 0.11 | 15.04 | 0.13 | -0.01 | 0.49 |
| MIP-1A | RA | 8.24 | 0.08 | 9.03 | 0.11 | -0.03 | 0.57 |
| MIP-1B | RA | 74.02 | 0.05 | 74.20 | 0.06 | 0.00 | 0.71 |
| PDGF-BB | RA | 7.58 | 0.48 | 7.59 | 0.58 | 0.00 | 0.92 |
| RANTES | RA | 14.21 | 0.08 | 16.24 | 0.06 | -0.02 | 0.32 |
| SCF | RA | 7.24 | 0.61 | 7.24 | 0.70 | 0.00 | 0.99 |
| SCGF-B | RA | 19.11 | 0.45 | 19.33 | 0.50 | -0.01 | 0.65 |
| SDF-1A | RA | 2.80 | 0.73 | 3.31 | 0.77 | 0.01 | 0.51 |
| TNF-A | RA | 3.45 | 0.18 | 3.72 | 0.29 | -0.01 | 0.73 |
| TNF-B | RA | 2.96 | 0.57 | 2.98 | 0.70 | 0.00 | 0.89 |
| TRAIL | RA | 28.09 | 0.30 | 28.10 | 0.35 | 0.00 | 0.94 |
| VEGF | RA | 16.85 | 0.66 | 17.06 | 0.71 | 0.00 | 0.65 |

Sensitivity analysis of MR, including pleiotropy analysis and heterogeneity analysis.
